# Interpretable AI-guided discovery of degradable ionizable lipids with enhanced hepatic mRNA delivery in vivo

**DOI:** 10.1038/s41467-026-78635-1

**Published:** 2026-10-12

**Authors:** Zhen Zhang, Yongbo Ren, Deqiang Kong, Jindong Feng, Kaiyi Hu, Xinyun Xu, Fukang Deng, Peixia Li, Jiuao Wang, Wenhui Huang, Guodan Wei, Yang Su, Ying Tan, Shuixing Zhang, Xiaodan Sun, Feiyu Kang, Hao Yan

**Affiliations:** 1 Tsinghua Shenzhen International Graduate School, Tsinghua University, Shenzhen, 518055 China; 2 Research Institute of Tsinghua, Pearl River Delta, Guangzhou, 510530 China; 3 Department of Biomedical Engineering, Boston University, Boston, MA 02215 USA; 4 College of Engineering, Georgia Institute of Technology, Atlanta, GA 30332 USA; 5 State Key Laboratory of Advanced Welding and Joining of Materials and Structures, School of Materials Science and Engineering, Harbin Institute of Technology (Shenzhen), Shenzhen, 518055 China; 6 School of Light Industry and Food Engineering, Guangxi University, Nanning, 530004 China; 7 Medical Imaging Center, The First Affiliated Hospital of Jinan University, Guangzhou, 510630 China; 8 Key Laboratory of New Ceramics and Fine Processing, Key Laboratory of Advanced Materials of Ministry of Education of China, School of Materials Science and Engineering, Tsinghua University, Beijing, 100084 China

**Keywords:** Drug delivery, Biomedical materials, Genetic vectors

## Abstract

Lipid nanoparticles (LNPs) enable RNA medicines, yet ionizable-lipid discovery remains largely empirical, particularly when in vitro activity must be translated into functional hepatic delivery in vivo. Here we establish an interpretable discovery framework that combines modular degradable lipid chemistry, high-throughput phenotyping, machine learning, virtual library expansion, and independent in vivo validation to define a learnable ionizable-lipid design space. Starting from a 520-member library, we train an XGBoost model on firefly luciferase (Fluc) expression in HeLa cells and use physicochemical descriptors and molecular fingerprints to prospectively prioritize candidates with higher predicted in vitro transfection activity across a 10,920-member virtual space. In vivo phenotyping subsequently identifies PA97MD101 as a lead ionizable lipid. In mice, PA97MD101 enhances hepatic mRNA expression and Cre-dependent recombination and supports Cas9/sgTtr-mediated TTR suppression. Its performance is associated with pH-dependent membrane activity and reduced lysosomal colocalization. These findings support further evaluation of PA97MD101 for functional hepatic RNA delivery.

## Abstract (web summary)

Ionizable-lipid discovery remains largely empirical. Here, the authors integrate interpretable machine learning with modular degradable lipid chemistry to prioritize candidates for experimental testing and identify a lead lipid for hepatic mRNA delivery.

## Introduction

Lipid nanoparticles (LNPs) are the leading nonviral platform for nucleic acid delivery, underpinning clinically validated small interfering RNA (siRNA) therapeutics, messenger RNA (mRNA) vaccines, and first-in-human in vivo genome editing^1–5^. By packaging nucleic acids into modular nanostructures, LNPs connect molecular design to in vivo pharmacology and convert sequence information into controlled protein expression or gene perturbation^6^. As nucleic acid therapeutics expand, however, the central bottleneck has shifted from payload design to delivery design: how to encode organ distribution, cell-type selectivity, and intracellular trafficking into LNP chemistry without compromising manufacturability or safety^7–13^.

Recent advances have established LNPs as programmable biomaterials rather than passive carriers^2,14^. Rationally engineered ionizable lipids, peptide–ionizable lipids, targeting ligands, and stimulus-responsive materials have extended mRNA and genome-engineering delivery across diverse tissues and disease settings^4,7,15–24^. In parallel, high-throughput in vivo screening, barcode-enabled functional reporters, and data-driven modeling have begun to shift LNP discovery from empirical optimization toward a more systematic exploration of chemical and formulation space^1,3,25–30^. Together, these developments suggest that LNP performance can, in principle, be learned, predicted, and prospectively engineered.

A central problem nevertheless remains unresolved: the relationship between ionizable-lipid structure and biological fate is still difficult to predict. In practice, structure–activity landscapes are frequently confounded by helper-lipid composition, formulation variables, manufacturing conditions, and protein corona effects, making it difficult to isolate the intrinsic contribution of the ionizable lipid itself^24,27,28,31–34^. At the same time, highly expressive deep-learning architectures generally require large labeled datasets, which are rarely available for newly synthesized chemical spaces^32,35–37^. This limitation is particularly consequential for liver-directed delivery. Although extrahepatic targeting has attracted broad interest, the liver remains the dominant therapeutic destination for protein replacement and gene correction. Yet successful liver delivery depends not simply on bulk hepatic accumulation, but on productive delivery to parenchymal hepatocytes, efficient endosomal escape, and functional intracellular release^38–40^. A key challenge is therefore to establish a data-efficient workflow that prioritizes ionizable lipids using in vitro transfection data and subsequently evaluates their hepatic functional delivery in vivo.

Here, we develop a two-stage machine-learning-guided framework for the discovery of degradable ionizable lipids. We integrate a 520-member amine modular degradable (AMD) lipid library, standardized high-throughput phenotyping, and interpretable machine learning to prioritize candidates from a virtual space of more than 10,000 lipids. Independent in vivo evaluation subsequently identifies PA97MD101 as a lead lipid with enhanced hepatic functional output across the tested mRNA applications. This study establishes a data-efficient workflow linking modular lipid chemistry and interpretable modeling with independent in vivo functional validation.

## Results

### A workflow links in vitro lipid prioritization with independent in vivo phenotyping

**Fig. 1:**
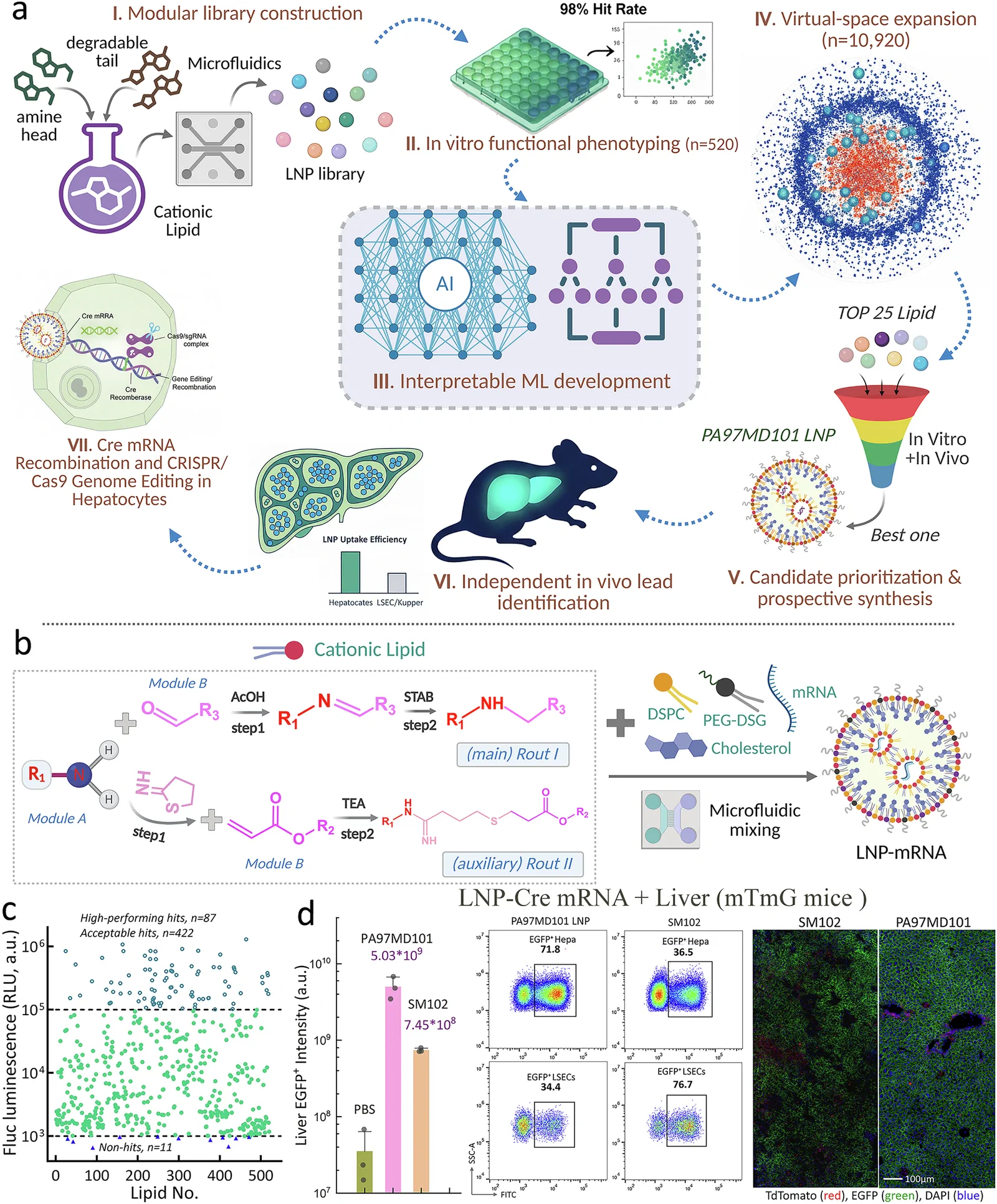
Two-stage AI-guided discovery and independent in vivo validation of degradable ionizable lipids. **a** Discovery workflow. The 520-member AMD library was screened using Fluc mRNA expression in HeLa cells, and the resulting in vitro labels were used for machine-learning-based prioritization within the 10,920-member PAMD virtual space. Selected candidates were subsequently synthesized and evaluated independently in vivo for organ-level and cell-population-resolved functional expression. **b** Synthetic and formulation strategy for AMD lipids and LNP preparation by microfluidic mixing. **c** In vitro Fluc mRNA transfection activity of the 520-member AMD library in HeLa cells. Lipids were descriptively categorized as non-hits (<10³ RLU; *n* = 11), acceptable hits (10³–10⁵ RLU; *n* = 422), or high-performing hits (>10⁵ RLU; *n* = 87). These categories were used for visualization only; model training used binary labels defined at 2000 RLU. **d** Functional validation and hepatocyte-enriched output of the lead lipid PA97MD101 in mTmG reporter mice. Left, ex vivo liver fluorescence and quantification of EGFP expression. Middle, flow cytometry of EGFP-positive cells within the CD31^−^CD45^−^ hepatocyte-enriched population, liver sinusoidal endothelial cells (LSECs), and Kupffer cells, showing hepatocyte-enriched functional expression for PA97MD101. Right, liver cryosections confirming widespread genetic recombination. For the quantitative analyses in (**d**), data are presented as mean ± SD (*n* = 3 biologically independent mice per group). Each data point represents one mouse. Panels a and b were created with BioRender. Yan, B. (2026) https://BioRender.com/1k7d6cg.

To determine whether degradable ionizable lipids can be prioritized prospectively rather than empirically, we established a two-stage workflow that integrates modular chemistry, high-throughput phenotyping, machine-learning-based prioritization, virtual library expansion, and independent in vivo validation (Fig. 1a). Rather than relying on empirical trial-and-error, the workflow is structured to (I–III) generate dense in vitro experimental labels in a chemically diverse but tractable AMD space and then (IV, V) use those labels to prospectively prioritize designs in a substantially larger virtual space for animal testing. We first constructed a 520-member AMD ionizable lipid library and generated quantitative activity labels via standardized in vitro screening. These experimentally anchored data were then used to train predictive models that guided in silico exploration of a 10,920-member predictive amine modular degradable (PAMD) virtual space, from which selected candidates were synthesized and advanced for independent in vivo evaluation of organ distribution, functional expression, and intrahepatic cell-type delivery (Fig. 1a).

AMD lipids were assembled using a mild one-pot route that couples Schiff-base formation/reduction with thiol-addition reactions, enabling rapid diversification across a broad chemical space while preserving a shared synthetic logic for downstream comparison (Fig. 1b). Unlike recently reported amidine-incorporated degradable lipids that require multi-step prefunctionalization and explore a narrower head–tail space^40^, our Route 1 directly ligates readily accessible amine heads and acrylate-derived degradable tails in a single convergent step, scaling to hundreds of ionizable lipids without route-specific optimization. By keeping lipid synthesis orthogonal to nucleic acid barcoding strategies for high-throughput nanoparticle pooling^25^, the chemistry retains the simplicity of small molecules while remaining compatible with pooled in vivo screening formats. LNPs were formulated by microfluidic mixing to reproducibly encapsulate mRNA and minimize batch-to-batch variability in functional readouts (Fig. 1b). Screening in HeLa cells revealed delivery activities spanning orders of magnitude, thereby defining a phenotypically rich landscape that could be partitioned into non-hits, intermediate performers, and high-performing formulations (Fig. 1c). This dense performance landscape provides an information-rich foundation for learning structure–function relationships and for guiding virtual expansion beyond the enumerated set.

We next asked whether this discovery framework could nominate candidates for independent in vivo testing. In membrane-targeted tdTomato/membrane-targeted enhanced green fluorescent protein (mTmG) reporter mice, PA97MD101 produced robust hepatic recombination and substantially greater integrated liver EGFP signal than SM102 at a matched dose (Fig. 1d, left). Flow cytometry quantified EGFP activation in the CD31^−^CD45^−^ hepatocyte-enriched population (Fig. 1d, middle), CD31⁺CD45^−^ endothelial/LSEC-enriched population, and CD45⁺ immune-cell compartment, while liver cryosections provided complementary spatial evidence (Fig. 1d, right). Thus, PA97MD101 showed enhanced hepatic functional output and a hepatocyte-enriched, rather than hepatocyte-exclusive, expression profile established by independent in vivo analysis.

### The AMD library defines a broad yet learnable delivery space

**Fig. 2:**
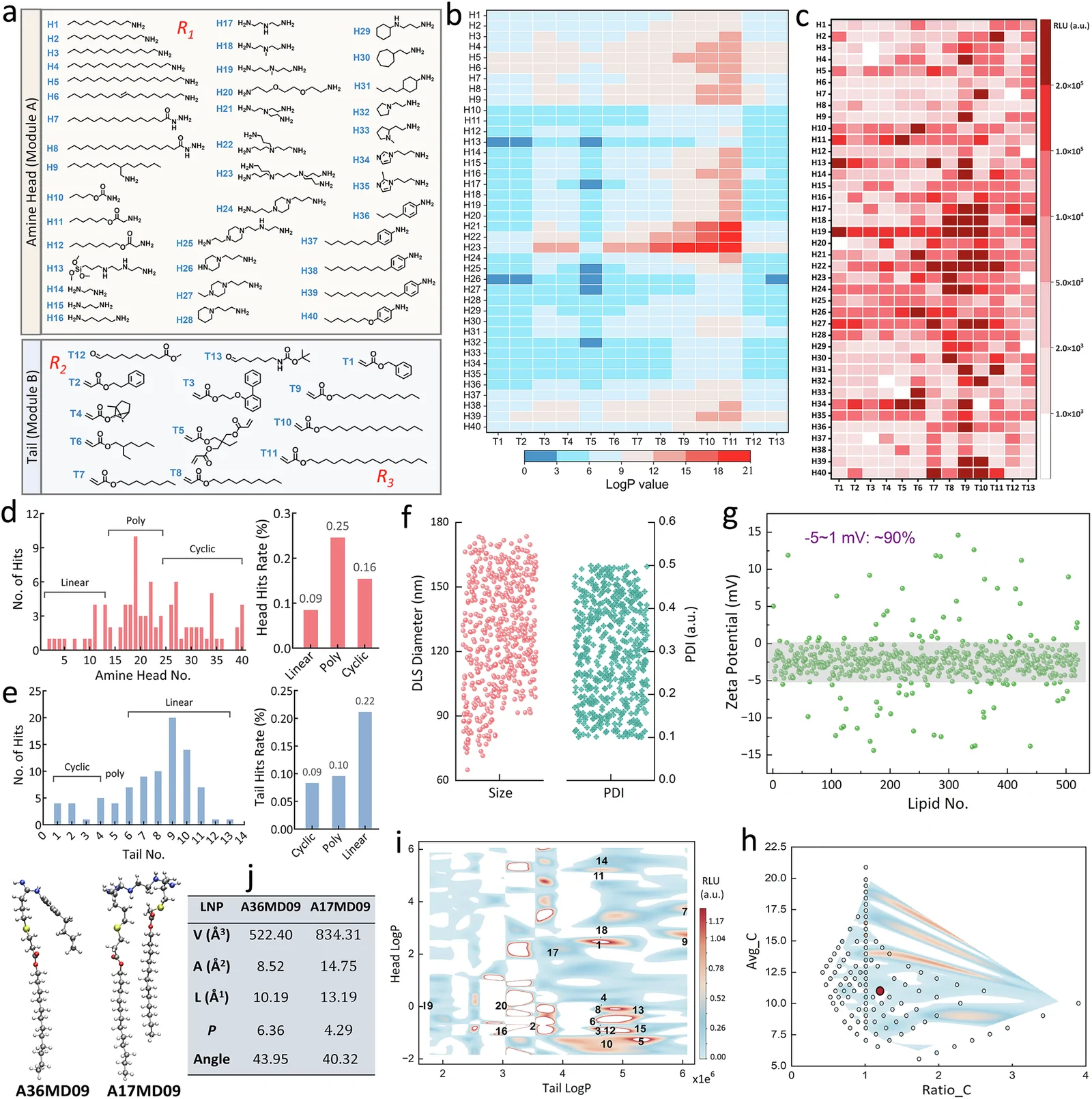
Modular synthesis and multidimensional profiling of the AMD lipid library. **a** Combinatorial design of the 520-member AMD library generated from 40 amine heads (H1–H40) and 13 degradable tails (T1–T13). **b** LogP distribution across the library, showing broad coverage of lipophilic chemical space. **c** Heatmap of in vitro mRNA delivery activity in HeLa cells, measured by luciferase expression (RLU), revealing distinct activity clusters. **d**, **e** Frequency of observed high-performing hits in the experimental AMD library, stratified by amine head (**d**) and tail (**e**), identifying privileged structural motifs. **f**, **g** Global physicochemical properties of the library, including hydrodynamic diameter, polydispersity index (PDI) (**f**), and zeta potential (**g**), with most formulations showing near-neutral surface charge. **h**, **i** Contour plots correlating transfection efficiency with tail-to-head carbon ratio and average carbon count (**h**), and with head and tail LogP values (**i**), defining an optimal hydrophobicity window. **j** Representative 3D conformations and calculated geometric parameters of lead lipids, highlighting the relationship between molecular shape and delivery performance.

To systematically connect chemical diversity to delivery performance, we constructed the AMD library as a 40 × 13 modular matrix comprising 40 amine heads (H1–H40) and 13 degradable tails (T1–T13) (Fig. 2a). This design expanded the accessible ionizable-lipid space while preserving a common synthetic framework compatible with machine-learning analysis. Mapping the library onto a lipophilicity landscape revealed broad chemical coverage, with distinct LogP distributions across head–tail combinations that support structured comparisons across related chemotypes (Fig. 2b). A library-wide luciferase heatmap revealed pronounced activity heterogeneity and discrete high-performance neighborhoods, indicating that the AMD matrix encodes a structured rather than random delivery landscape (Fig. 2c). Consistent with this view, hit enrichment was nonuniform across both head and tail modules, with only a subset of motifs disproportionately represented among active formulations (Fig. 2d, e).

To determine whether functional variation primarily reflected chemistry rather than gross formulation failure, we profiled key physicochemical properties across the library. Most AMD LNPs formed nanoparticles within a narrow nanoscale size range and with low dispersity (Fig. 2f; Supplementary Data 2), while zeta-potential measurements showed that the majority of formulations remained near-neutral under physiological conditions (Fig. 2g; Supplementary Data 1). These data indicate that screening was conducted within a broadly formulatable space, such that major differences in biological output are unlikely to arise from gross assembly failure alone. The results suggest that delivery performance is encoded by chemistry-dependent features that can be systematically learned.

Descriptor-based analyses supported this interpretation. Delivery efficiency was enriched within constrained compositional windows defined by tail-to-head carbon ratio and average carbon count (Fig. 2h), and by a head-versus-tail LogP balance that was permissive for high activity (Fig. 2i). Notably, the library-wide lipophilicity–activity relationship was clearly nonmonotonic (Supplementary Fig. 1), arguing against a simple more hydrophobic is better model. Finally, conformational modeling of representative candidates yielded geometric parameters, including packing parameter and cone angle, that linked modular structures to assembly-relevant molecular envelopes (Fig. 2j; Supplementary Fig. 2). Together, these measurements characterize the chemical and physicochemical diversity of the library and provide a basis for subsequent predictive modeling.

### Interpretable machine learning prospectively expanded the searchable lipid space and enriched for potent candidates

**Fig. 3:**
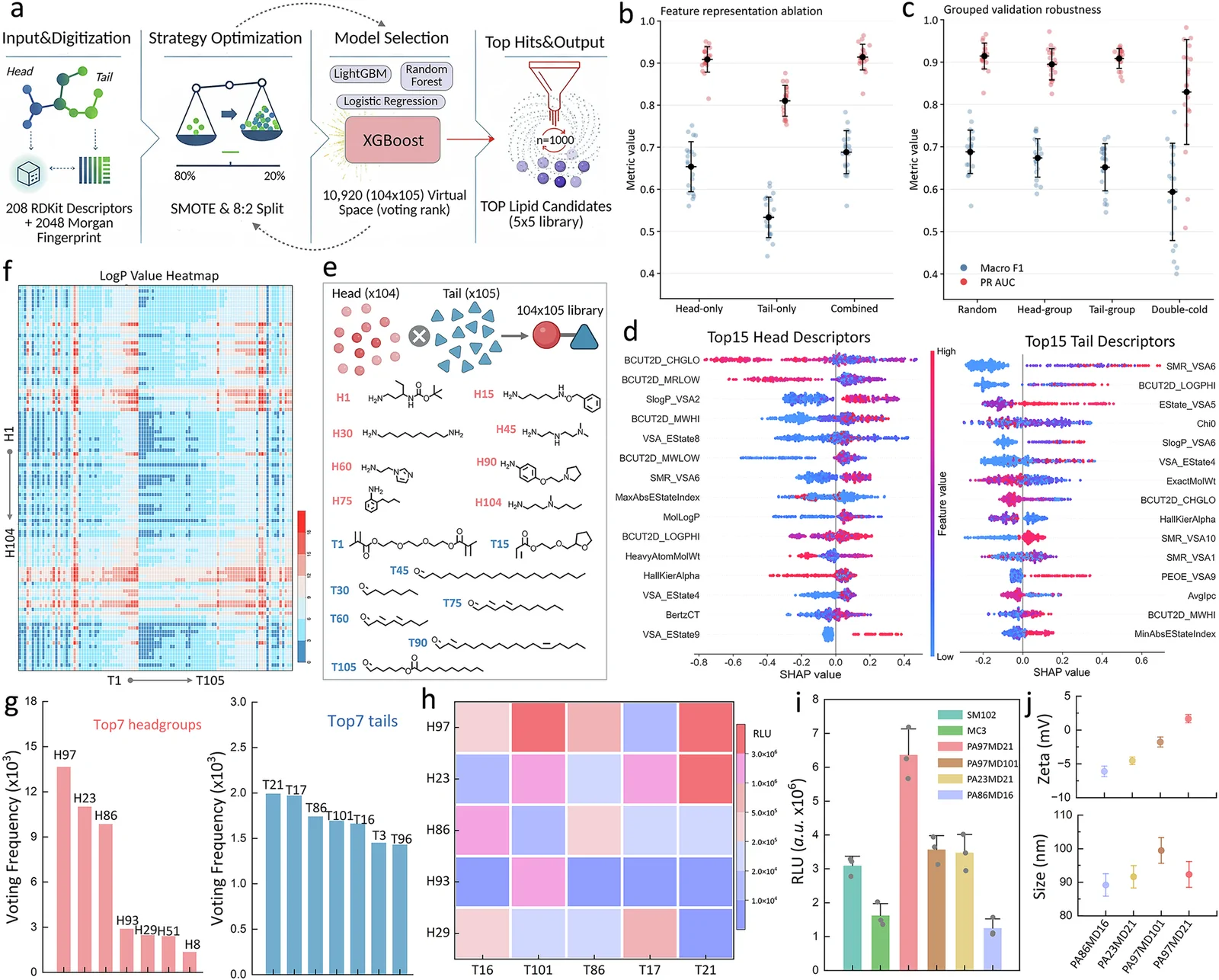
Machine learning-guided virtual screening and validation of high-performance ionizable lipids. **a** Computational workflow for lipid discovery, including molecular featurization, SMOTE-like oversampling data balancing, and supervised model training. **b** Feature ablation of head-only, tail-only, and combined molecular representations under the unified ML workflow. Model performance was evaluated across 20 repeated train-test splits (*n* = 20 splits). The same splits were used for the head-only, tail-only, and combined representations. Data are presented as mean ± SD across splits. **c** Model robustness evaluated under random, head-grouped, tail-grouped, and double-cold validation strategies. Blue and red dots indicate Macro F1 and PR AUC, respectively; black dots and error bars show mean ± s.d. across 20 splits. **d** SHAP analysis of molecular descriptors associated with the HeLa-cell transfection phenotype, shown separately for the Head and Tail modules. **e**, **f** Schematic of the 10,920-member PAMD virtual library (**e**) and its LogP landscape (**f**), illustrating broad chemical diversity. **g** Frequency distribution of privileged amine heads and tails among top-ranked candidates identified by ensemble voting. **h**, **i** Experimental validation of predicted PAMD lipids by in vitro luciferase screening in HeLa cells (**h**) and quantitative comparison with MC3 and SM102 (**i**). **j** Physicochemical characterization of lead candidates, showing particle sizes and zeta potentials consistent with stable LNP formation. For **i**, data are presented as mean ± SD from three independent experiments (*n* = 3 independent experiments). Within each experiment, three technical replicate wells were averaged to obtain one value per formulation. Each plotted point represents the mean of one independent experiment. For **j**, particle size and zeta potential are presented as mean ± SD from three independently prepared LNP samples per formulation (*n* = 3 independent preparations). Panel **a** was created with BioRender. Yan, B. (2026) https://BioRender.com/kckn10l. Panel **e** was created with BioRender. Yan, B. (2026) https://BioRender.com/kqnrm0q.

To move beyond empirical screening, we trained supervised models on the experimentally labeled AMD set and used them to prospectively prioritize candidates in an expanded chemical space (Fig. 3a). Lipids were encoded using RDKit physicochemical descriptors and Morgan fingerprints, enabling the model to capture both interpretable physicochemical information and substructure-level patterns. To improve calibration under cross-validated training and reduce inflation of rare positives, class imbalance was addressed during model development (Fig. 3a). Random Forest, XGBoost, LightGBM, and Logistic Regression were compared using identical feature matrices, matched splits, and training-data-only preprocessing, with macro-F1 and PR-AUC emphasized under class imbalance. XGBoost showed the best overall balance across the revised metrics and was selected for virtual-library prioritization (Supplementary Table 1). Feature ablation showed that the combined Head–Tail representation performed best, with substantial predictive information retained in Head features and a more limited contribution from Tail features (Fig. 3b). Under structure-constrained validation, XGBoost retained candidate-enrichment capability in Head- and Tail-grouped splits but showed reduced performance under strict double-cold validation, indicating uncertainty when both modules were unseen (Fig. 3c and Supplementary Fig. 3). Additional baseline and class-imbalance sensitivity analyses supported this evaluation framework (Supplementary Figs. 4 and 5).

SHapley Additive exPlanations (SHAP) analysis identified descriptors associated with the HeLa-cell transfection phenotype. Head and Tail descriptors were calculated separately and assigned to their respective modules using feature prefixes (Fig. 3d). Complementary interpretability analyses are provided in Supplementary Figs. 6–10. Module-resolved rankings highlighted descriptors related to charge distribution, lipophilicity, and topological connectivity (Supplementary Fig. 11), while correlation-network analysis revealed related physicochemical clusters and partial redundancy among model inputs (Supplementary Fig. 12). Together, these results suggest that in vitro activity reflects coordinated physicochemical properties rather than a single dominant feature. These associations are structural correlates of the in vitro phenotype; organ distribution and hepatocyte-enriched functional expression were evaluated independently in vivo. Guided by this validated model, we combinatorially paired an extended set of 104 amine heads (H1–H104; Supplementary Fig. 13) and 105 degradable tails (T1–T105; Supplementary Fig. 14) to construct a 10,920-member virtual PAMD space (Fig. 3e). Chemical-space mapping revealed broad lipophilicity coverage across this extrapolated library (Fig. 3f; Supplementary Fig. 15). To identify privileged structural motifs for in vitro activity-based prioritization, we quantified the recurrence of building blocks among top-ranked candidates and found that only a limited subset of heads and tails repeatedly appeared across high-scoring designs (Fig. 3g; Supplementary Figs. 16–18), thereby nominating practical modules for focused synthesis.

We then synthesized and experimentally validated ML-prioritized PAMD candidates. In vitro screening showed that multiple predicted lipids drove strong luciferase expression in HeLa cells, with several outperforming widely used benchmark ionizable lipids, including MC3 and SM102 (Fig. 3h, i). These lead candidates also retained physicochemical properties consistent with robust nanoparticle formation, including near-neutral surface charge, narrow size distributions, and low PDIs (Fig. 3j; Supplementary Fig. 19). Representative lead structures were confirmed by NMR and HRMS (Supplementary Figs. 20–22). Together, these results establish an enrichment workflow in which machine learning prioritizes experimentally testable lipids with higher predicted in vitro activity, followed by direct synthesis, formulation, and biological evaluation.

### Enhanced endosomal escape drives lead lipid performance

**Fig. 4:**
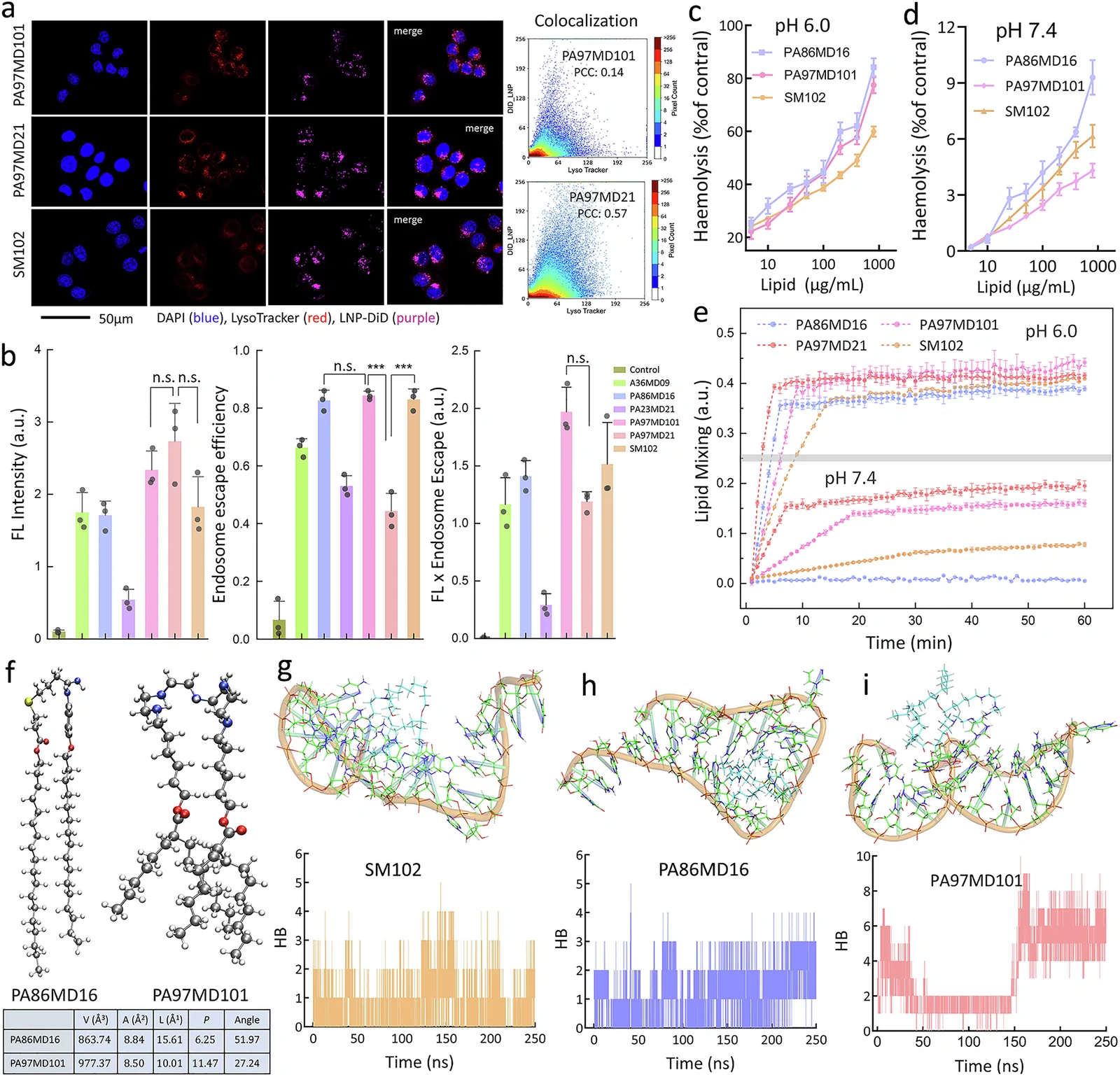
Mechanistic basis of enhanced mRNA delivery by lead ionizable lipids. **a**, **b** Confocal imaging (**a**) and quantification (**b**) of DiD-labeled LNP uptake and LysoTracker colocalization in HeLa cells show that lead candidates achieve superior endosomal escape despite variable uptake. For **b**, *n* = 3 independent cell-culture experiments per treatment. Measurements from three fields of view within each experiment were averaged to obtain one value per experiment. Data are presented as mean ± SD. **c**, **d** Dose-dependent hemolysis at pH 6.0 (**c**) and pH 7.4 (**d**) demonstrates strong acid-triggered membrane-disruptive activity with minimal lysis at physiological pH. For **c**, **d**, *n* = 3 biologically independent erythrocyte preparations obtained from three different mice. Technical replicate measurements were averaged within each preparation. Data are presented as mean ± SD. **e** FRET-based lipid mixing assays reveal accelerated fusion of lead LNPs with anionic membranes under acidic conditions. For **e**, data are presented as mean ± SD from three independent lipid-mixing experiments using independently prepared LNP and model-liposome samples (*n* = 3 experiments). **f** Energy-minimized 3D conformations and calculated geometric parameters indicate cone-shaped architectures favorable for membrane destabilization. **g**–**i** Molecular dynamics simulations of SM102 (**g**), PA86MD16 (**h**), and PA97MD101 (**i**) show time-dependent hydrogen bonding and stable interactions between ionizable heads and the RNA backbone. Together, these data support enhanced cytosolic release as a major determinant of lead lipid performance. Statistical significance was assessed using one-way ANOVA followed by Tukey’s multiple-comparisons test. All pairwise comparisons were included in the analysis, but only selected comparisons are annotated in the figure for clarity. Reported *P* values are adjusted for multiple comparisons. *P < 0.05, **P < 0.01, ***P < 0.001 and ****P < 0.0001; n. s., *P* ≥ 0.05.

Having identified high-performing, ML-nominated lipids, we next asked which step in the delivery cascade differentiates lead candidates from benchmark ionizable lipids. Confocal imaging of DiD-labeled LNPs together with LysoTracker staining revealed formulation-dependent trafficking patterns in HeLa cells (Fig. 4a). Quantitative analysis showed that, although cellular uptake varied across formulations, PA97MD101 and PA86MD16 consistently displayed lower overlap with acidic endo-lysosomal compartments than SM102 and poorly performing analogs (Fig. 4b; Supplementary Fig. 23). Thus, lead performance was not explained by uptake alone, but instead by a larger fraction of material escaping degradative compartments and gaining productive cytosolic access.

This interpretation was further supported by pH-dependent membrane activity. Lead formulations showed pronounced, dose-dependent hemolysis under endosome-relevant acidic conditions (Fig. 4c), but much weaker activity at physiological pH (Fig. 4d), consistent with a pH-gated membrane-disruptive mechanism. A Förster resonance energy transfer (FRET)-based lipid-mixing assay similarly showed accelerated fusion with anionic model membranes under acidic conditions, whereas fusion was minimal at neutral pH (Fig. 4e; Supplementary Fig. 24). Apparent pKa values fell within an endosome-relevant ionization window, and all lead candidates retained high encapsulation efficiencies (Supplementary Fig. 25), indicating that performance differences were more tightly linked to membrane remodeling and intracellular release than to cargo loading or gross assembly.

To connect these phenotypes back to molecular design, computational conformational analyses highlighted cone-shaped architectures in lead lipids, consistent with curvature stress and membrane destabilization (Fig. 4f; Supplementary Figs. 26 and 27). Molecular dynamics simulations further suggested strengthened and sustained lipid–RNA interactions relative to SM102, supported by time-resolved hydrogen-bond analyses and representative docking snapshots (Fig. 4g–i). Collectively, these orthogonal measurements support an escape-dominant mechanism in which pH-activated membrane activity, favorable molecular geometry, and reinforced lipid–RNA interactions cooperate to enhance productive cytosolic release.

### PA97MD101 enhances hepatic mRNA delivery in vivo

**Fig. 5:**
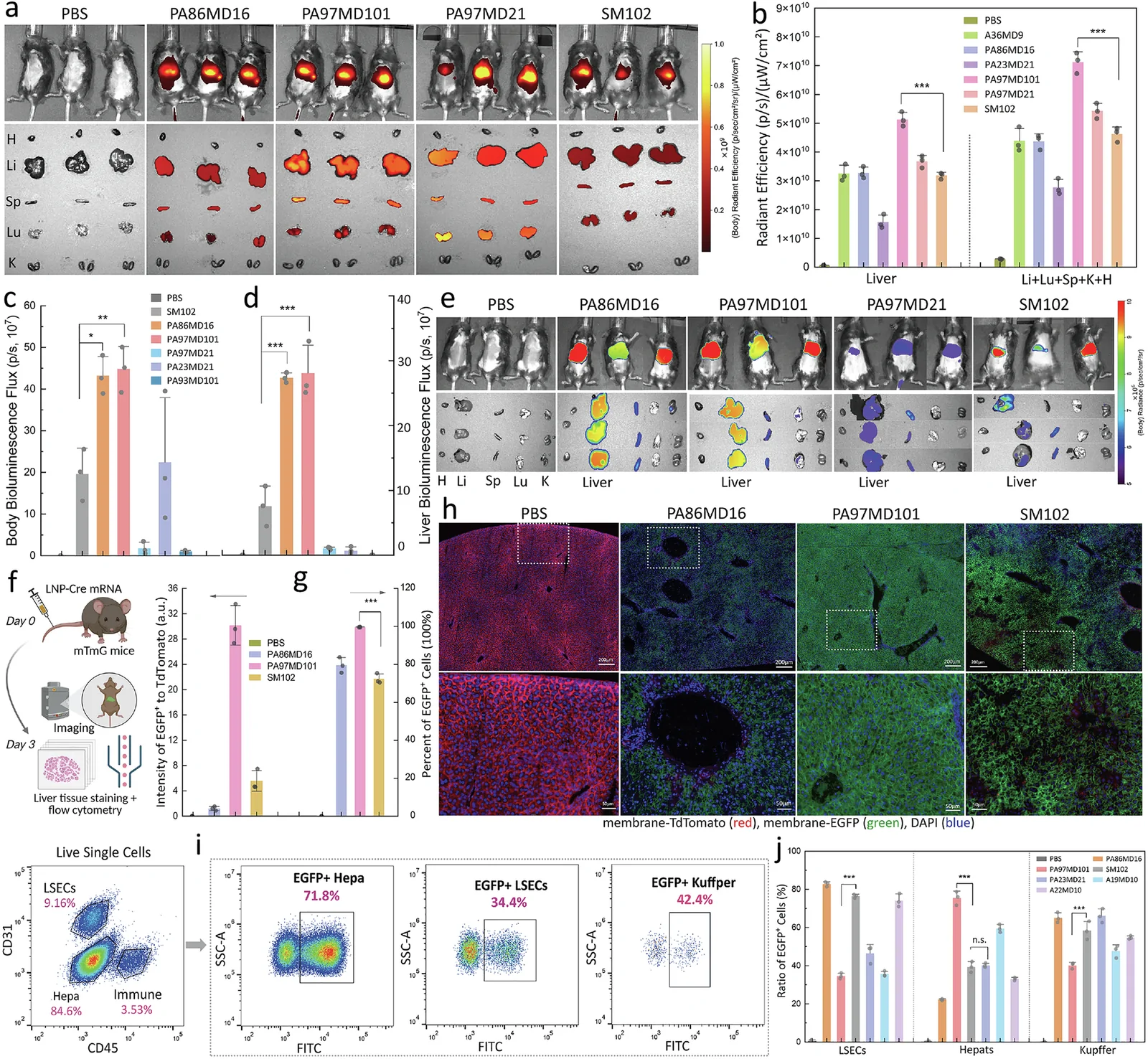
In vivo organ distribution and cell-type-resolved functional mRNA delivery of prioritized LNPs. **a**, **b** Whole-body and ex vivo fluorescence imaging (**a**) and ROI quantification (**b**) of DiR-labeled LNPs in C57BL/6 mice show predominant hepatic accumulation of PA86MD16 and PA97MD101, comparable to SM102. **c**–**e** Bioluminescence imaging and quantification of Fluc mRNA delivery 6 h after administration demonstrate enhanced hepatic protein expression by ML-prioritized LNPs relative to benchmark formulations. H heart, Li liver, Sp spleen, Lu lungs, K kidneys, PBS phosphate-buffered saline, ROI region of interest, Fluc firefly luciferase, EGFP enhanced green fluorescent protein, LSECs liver sinusoidal endothelial cells. **f**–**h** In mTmG reporter mice, Cre mRNA delivery induces robust tdTomato-to-EGFP conversion, as shown by the experimental scheme (**f**), liver fluorescence quantification (arb. units, arbitrary units) (**g**), and representative confocal images (**h**). In **h**, membrane-targeted tdTomato is shown in red, membrane-targeted EGFP in green, and DAPI-stained nuclei in blue. **i**, **j** Flow cytometric analysis of liver cell subsets identifies EGFP-positive fractions within the CD31^−^CD45^−^ hepatocyte-enriched population, liver sinusoidal endothelial cells (LSECs), and Kupffer cells, revealing that PA97MD101 shows hepatocyte-enriched functional expression relative to non-parenchymal populations. For (**b**–**d**, **g**, **j**), *n* = 3 biologically independent mice per treatment group for each experiment. Each data point represents one mouse. Data in (**b**–**d**, **g**, **j**) are presented as mean ± SD. Statistical significance was assessed using one-way ANOVA followed by Tukey’s multiple-comparisons test. All pairwise comparisons were included in the analysis, but only selected comparisons are annotated in the figure for clarity. Reported *P* values are adjusted for multiple comparisons. *P < 0.05, **P < 0.01, ***P < 0.001 and ****P < 0.0001; n. s., *P* ≥ 0.05. Panel **f** was created with BioRender. Yan, B. (2026) https://BioRender.com/wfxc4j8.

We next asked whether ML-prioritized lipids translate into bona fide in vivo performance. DiR-labeled LNPs in C57BL/6 mice showed predominant hepatic enrichment for both lead candidates in whole-body and ex vivo imaging, with ROI quantification indicating liver accumulation broadly comparable to SM102 (Fig. 5a, b; Supplementary Fig. 28). Expanded profiling across major organs confirmed liver-dominant signals, with hepatic fluorescence intensities far exceeding extrahepatic signals on a logarithmic scale (Supplementary Fig. 29). These data show that the lead PAMD scaffolds are not only potent in vitro, but also support liver-dominant biodistribution after systemic administration under the tested conditions.

We then quantified functional translation using Fluc mRNA. At a matched dose, PA97MD101 produced approximately fourfold higher hepatic Fluc output than SM102 despite broadly comparable liver accumulation (Fig. 5c–e; Supplementary Figs. 30–32). Expanded analysis of A19MD10, A22MD10, A36MD09, PA23MD21, and PA93MD101 revealed distinct whole-body, hepatic, and extrahepatic functional-expression profiles. Thus, favorable HeLa-cell activity did not translate uniformly into either the magnitude or anatomical distribution of functional mRNA expression in vivo, supporting the use of the model for candidate prioritization rather than the prediction of systemic performance.

To apply a more stringent genome-level readout, we used the Cre/LoxP-based mTmG reporter system, in which successful Cre mRNA delivery and expression induce permanent tdTomato-to-EGFP conversion (Fig. 5f; Supplementary Fig. 33). PA97MD101 produced stronger liver-level recombination than SM102, supported by fluorescence imaging, confocal microscopy, and LoxP-specific qPCR (Fig. 5g, h; Supplementary Figs. 34–38). Flow cytometry showed distinct EGFP-activation profiles across the CD31⁻CD45⁻ hepatocyte-enriched, CD31⁺CD45⁻ endothelial/LSEC-enriched and CD45⁺ immune-cell compartments (Fig. 5i, j; Supplementary Figs. 39–45). PA97MD101 showed greater functional expression in the hepatocyte-enriched population and lower expression in non-parenchymal populations than SM102. EGFP activation integrates delivery, translation, and recombination and should not be interpreted as a direct measurement of nanoparticle uptake or receptor-specific targeting.

### PA97MD101 generalizes across reporter, therapeutic, and CRISPR payloads while preserving acute tolerability

**Fig. 6:**
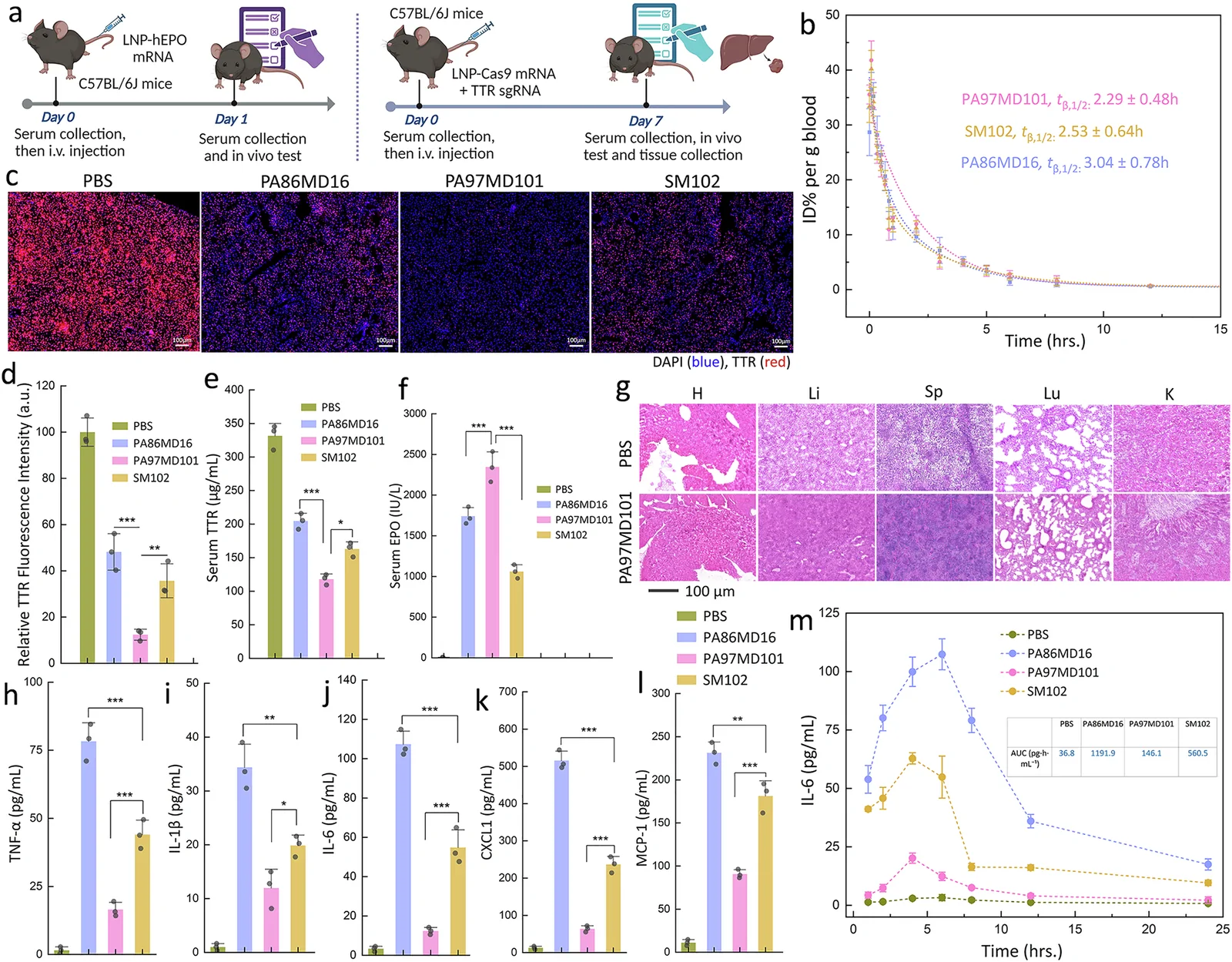
Functional CRISPR delivery, pharmacokinetics, and safety of lead ML-prioritized LNPs. **a** Experimental timeline for systemic delivery of hEPO mRNA and CRISPR–Cas9/sgRNA cargos for functional evaluation and safety analysis. **b** Blood pharmacokinetic profiles of selected LNPs over 24 h, showing distinct circulation and clearance behaviors of lead candidates. For **b**, *n* = 3 biologically independent mice per formulation. Data are presented as mean ± SD. **c**–**e** In vivo CRISPR/Cas9-mediated TTR silencing in the liver, assessed by representative FISH images of Ttr mRNA (**c**), In **c**, Ttr mRNA signals are shown in red and DAPI-stained nuclei in blue. FISH signal quantification (arb. units, arbitrary units) (**d**), and serum TTR concentrations measured by ELISA 7 days after treatment (**e**). For **d**, *n* = 3 biologically independent mice per group. FISH measurements from three fields of view per mouse were averaged to obtain one value per mouse. Each plotted point represents one mouse. **f** Serum human erythropoietin (hEPO) levels 24 h after administration of hEPO mRNA-loaded LNPs, demonstrating efficient translation across cargo types. **g** H&E staining of major organs showing no detectable acute tissue injury. **h**–**l** Serum cytokine profiling of TNF-α, IL-1β, IL-6, CXCL1, and MCP-1 at 6 h post-injection, indicating low acute immunogenicity of PA97MD101. For **e**, **f**, **h**–**l**, *n* = 3 biologically independent mice per group for each experiment. Each data point represents the serum measurement from one mouse. Data are presented as mean ± SD. **m** Time-course of serum IL-6, with inset AUC analysis, highlighting the favorable safety window of ML-prioritized lipids. These payload studies indicate that the lead lipid generalizes across reporters, secreted proteins, and functional CRISPR cargos while maintaining favorable acute tolerability. For **m**, *n* = 3 biologically independent mice per group, with repeated measurements collected from the same mice over time. Data are presented as mean ± SD. Statistical significance was assessed using one-way ANOVA followed by Tukey’s multiple-comparisons test. All pairwise comparisons were included in the analysis, but only selected comparisons are annotated in the figure for clarity. Reported *P* values are adjusted for multiple comparisons. *P < 0.05, **P < 0.01, ***P < 0.001 and ****P < 0.0001; n.s., *P* ≥ 0.05. Panel **a** was created with BioRender. Yan, B. (2026) https://BioRender.com/wfxc4j8.

Having characterized the hepatic functional expression of PA97MD101, we next evaluated its performance with additional payloads and assessed its acute tolerability. Pharmacokinetic profiling over 24 h revealed distinct circulation and clearance patterns across formulations and provided an exposure framework for downstream efficacy and tolerability comparisons (Fig. 6a, b).

To evaluate functional CRISPR cargo delivery in vivo, we delivered Cas9/sgTtr and quantified hepatic on-target consequences. FISH imaging showed substantially greater loss of Ttr mRNA with PA97MD101 than with SM102, corresponding to a roughly two to threefold deeper reduction in hepatic signal (Fig. 6c, d; Supplementary Fig. 46) and consistent with lower serum TTR by ELISA (Fig. 6e). Beyond Cas9-based disruption, PA97MD101 also efficiently delivered hEPO and mCherry mRNAs, yielding detectable serum hEPO at 24 h and broad intracellular fluorescence, respectively (Fig. 6f; Supplementary Fig. 47). These data indicate that the lead lipid generalizes across reporter, secreted-protein, and CRISPR payloads, consistent with intracellular release as a dominant determinant of functional output.

We finally assessed acute tolerability using a prespecified in vivo design (Supplementary Fig. 48) spanning histology and systemic cytokine responses. H&E staining of major organs showed no overt tissue injury or inflammatory infiltration after administration of lead formulations (Fig. 6g; Supplementary Fig. 49). Serum cytokine profiling at 6 h post-injection indicated minimal inflammatory perturbation for PA97MD101, with low levels of TNF-α, IL-1β, IL-6, CXCL1, and MCP-1 (Fig. 6h–l). IL-6 time-course analysis and area-under-the-curve comparisons further supported a favorable acute inflammatory window for the ML-prioritized LNP (Fig. 6m). Collectively, these results support a discovery workflow combining modular synthesis, in vitro screening, and machine-learning-based prioritization with independent in vivo evaluation, and identify PA97MD101 as a lead lipid for hepatic mRNA delivery with favorable acute tolerability under the tested conditions.

## Discussion

LNPs have evolved from empirical carriers into programmable biomaterials that encode organ tropism, intracellular trafficking, and immune activity. Here, we establish an interpretable workflow for the prospective prioritization of degradable ionizable lipids using in vitro transfection data, followed by independent evaluation of hepatic mRNA delivery in vivo. A modular AMD library, built by a mild one-pot chemistry that directly ligates amine heads to degradable tails, was coupled to high-throughput phenotyping, interpretable machine learning, virtual expansion, and in vivo validation. Head-to-head comparisons with the clinical benchmark SM102 showed that the lead lipid PA97MD101 produced approximately fourfold higher hepatic Fluc output, ~6.75-fold higher liver-level Cre-dependent recombination, and approximately two to threefold deeper Cas9/sgTtr-mediated transthyretin perturbation at matched doses in vivo. Using data from a few hundred structurally diverse lipids, this framework identified cone-shaped, pH-responsive chemotypes with privileged endosomal escape and prospectively enriched lipids with strong in vitro functional activity, among which independent in vivo phenotyping identified PA97MD101 with hepatocyte-enriched functional output under the tested conditions. These findings highlight intracellular delivery processes as important considerations when evaluating hepatic functional expression, alongside organ-level accumulation.

Methodologically, our study contributes on two fronts. First, the AMD synthetic route generalizes recent degradable lipid chemistries by expanding the accessible head–tail space while retaining a unified, one-pot logic compatible with library-scale formulation and screening. In contrast to multi-step amidine-incorporated lipids that require scaffold-specific route optimization^40,41^, this head–tail ligation strategy scales to hundreds of ionizable lipids without re-engineering the reaction sequence, thereby providing a chemically coherent backbone for structure–activity learning. Second, rather than using machine learning purely as a black-box predictor—as is common in models requiring large training datasets^35,36^—we trained an interpretable model using module-level RDKit physicochemical descriptors to prioritize candidates with higher predicted in vitro functional transfection activity. This combination of modular chemistry and interpretable modeling enables both prospective lead discovery and extraction of experimentally testable design rules.

In the broader context of LNP-enabled gene medicine, our results complement several ongoing trends while sharpening one central design principle. Recent studies have shown that rationally engineered ionizable lipids and formulations can retarget LNPs to the retina^15^, lung stem cells^42^, in situ CAR-T generation^43^, and transient hepatic reconstitution^44^ and liver fibrosis^45^. Parallel work has shown that LNPs can be tuned along an immunological axis—from strongly adjuvant mRNA vaccines to tolerogenic formulations^23^, and placenta-tropic VEGF mRNA LNP^46^. Our study contributes to this landscape by combining interpretable modeling of in vitro transfection activity with independent evaluation of hepatic mRNA delivery. The model identifies molecular features associated with the HeLa-cell transfection phenotype, whereas the hepatic performance and intrahepatic expression profile of PA97MD101 are established through subsequent in vivo experiments.

Future studies could adapt this workflow to other tissues or cell populations by incorporating training data that directly measure the relevant functional delivery phenotype. Such applications would require prospective validation in the intended biological systems. The same two-stage workflow—modular synthesis, high-throughput phenotyping, interpretable ML, virtual expansion, and in vivo validation—can in principle be reparameterized for extrahepatic targets by changing the training phenotype (for example, to lung epithelium^7^, or tissue-resident immune cells) and incorporating route- and ligand-dependent variables. Our observation that efficient in vivo function is governed more by endosomal escape and molecular architecture than by bulk uptake suggests that future AI-guided LNP design should explicitly integrate intracellular processing alongside biodistribution. Because the synthetic strategy is orthogonal to nucleic-acid barcoding and pooled in vivo readouts, it is naturally compatible with emerging high-throughput nanoparticle barcoding platforms^25,26^, opening the door to combined AI–barcoding maps of tissue and cell-type selectivity.

Several limitations should be considered. The model was trained on HeLa-cell Fluc expression and did not include organ distribution or intrahepatic cell-type preference as training labels. It should therefore be viewed as a scalable enrichment tool for cell-intrinsic functional delivery, rather than a stand-alone predictor of systemic pharmacology. Accordingly, SHAP and descriptor analyses identify features associated with the in vitro phenotype, not causal determinants of hepatocyte-enriched delivery. Performance also decreased under strict double-cold validation, highlighting uncertainty for unseen head–tail combinations. Direct prediction of hepatocyte preference will require matched datasets integrating lipid structure, formulation properties, circulation, organ exposure, and cell-type-resolved functional labels.

Taken together, this study establishes a data-efficient workflow that integrates modular degradable lipid synthesis, in vitro phenotyping, interpretable machine learning, and independent in vivo evaluation. The model supports candidate prioritization based on HeLa-cell transfection activity, while subsequent experiments identify PA97MD101 as a lead lipid for hepatic mRNA delivery. Its intrahepatic expression profile represents an experimentally observed property under the tested conditions and does not establish a general ability to design hepatocyte-selective lipids. Extending this approach to predict tissue or cell-type preference will require training datasets containing the corresponding in vivo functional measurements.

## Methods

### One-pot high-throughput synthesis of AMD lipids

#### Route I: acrylate-based degradable lipids

The AMD library was constructed using two one-pot synthetic strategies classified by degradable tail linkage. Acrylate-type lipids (Route I) were prepared via a one-pot ring-opening/Michael-addition sequence using Traut’s reagent. Amine headgroups (0.06 mmol, 1.0 equiv.), 2-iminothiolane hydrochloride (Traut’s reagent; 0.075 mmol, 1.25 equiv.), acrylate tails (0.09 mmol, 1.5 equiv.), and TEA (0.075 mmol, 1.25 equiv.) were dissolved in anhydrous ethanol (0.5 mL) in 1.5 mL microcentrifuge tubes. Reaction mixtures were sonicated in an ultrasonic bath (40 kHz) at room temperature for 40–60 min.

For polyamines containing multiple reactive centers, the equivalents of Traut’s reagent and acrylate were scaled proportionally to the number of primary amines. Due to the high concentration of crude mixtures, reactions were diluted tenfold with ethanol before high-throughput screening.

For characterization, representative reactions were purified. For example, 4-butylaniline (8.95 mg, 0.06 mmol), dodecyl acrylate (21.63 mg, 0.09 mmol), Traut’s reagent (10.32 mg, 0.075 mmol), and TEA (7.58 μL, 0.075 mmol) were combined in 0.5 mL of ethanol and sonicated for 30 min. After complete consumption of Traut’s reagent (monitored visually), the crude mixture was purified by column chromatography to afford AMD lipids (21.33 mg, 91.2% yield). For library screening, crude products were used without purification. Representative purified multi-amine-derived products and lead lipids were characterized by ¹H NMR, ¹³C NMR, and HRMS.

### Route II: aldehyde-based degradable lipids

Aldehyde-type lipids (Route II) were synthesized via a one-pot, two-step reductive amination. Amine headgroups (0.1 mmol, 1.0 equiv.), degradable aldehyde tails (0.12 mmol, 1.2 equiv.), and acetic acid (0.48 mmol, 4.8 equiv.) were dissolved in DCM (1 mL) in sealed vials equipped with magnetic stir bars. Mixtures were heated at 80 °C with stirring for 3 h. After cooling to room temperature, STAB (0.5 mmol, 5.0 equiv.) was added, and the reaction was stirred at room temperature for 24 h.

Reactions were quenched with saturated aqueous NaHCO₃, extracted with DCM, washed with brine, dried over anhydrous Na_2_SO₄, filtered (0.22 μm), and concentrated under reduced pressure. A representative procedure used tetraethylenepentamine (18.93 mg, 0.1 mmol), 6-oxohexyl 2-hexyldecanoate (85.09 mg, 0.24 mmol), acetic acid (28.82 mg, 0.48 mmol), and STAB (106 mg, 0.5 mmol) in 1 mL DCM to provide a PAMD lipid (51.39 mg, 62.4% yield) after column chromatography. For library generation and in vitro screening, crude products were used without chromatographic purification. Structural characterization of representative ionizable lipids by ¹H/¹³C NMR and HRMS is provided in Supplementary Information.

### In vitro Fluc mRNA transfection screening

HeLa cells were seeded in black, clear-bottom 96-well plates (LABSELECT) at 1 × 10⁴ cells per well in 100 μL DMEM supplemented with 10% FBS and 1% penicillin–streptomycin. After 24 h incubation at 37 °C and 5% CO₂, the medium was replaced with fresh complete DMEM, and Fluc mRNA–LNPs were added at a final total lipid concentration of 50 μg mL^−1^ (lipid:mRNA mass ratio 40:1). Transfections were performed in triplicate (*n* = 3 technical replicates) for each lipid candidate. After 24 h, luciferase activity was quantified using a Luciferase Reporter Gene Assay Kit (Cat. No. 11401ES80, Yeasen Biotechnology) according to the manufacturer’s instructions.

### In vivo bioluminescence imaging and biodistribution

C57BL/6 mice (6–8 weeks old) were randomly assigned to treatment groups (*n* = 3 per group). Mice were anesthetized with isoflurane, and chest and abdominal fur was removed using depilatory cream (Veet). Fluc mRNA–LNPs were injected via the tail vein (0.1 mg kg^−1^ mRNA in 100 μL). Six hours post-injection, mice received intraperitoneal D-luciferin potassium salt (150 mg kg^−^¹, 150 μL of 20 mg mL^−1^ solution). After 10 min, whole-body bioluminescence imaging was performed using an IVIS Spectrum system (PerkinElmer) with a 1 min exposure. Major organs (heart, liver, spleen, lung, kidney) were then harvested for ex vivo imaging under identical settings. Data were analyzed using Living Image software.

### Quantification of hEPO expression

For systemic protein replacement, C57BL/6 mice received hEPO mRNA–LNPs via tail vein injection (0.25 mg kg^−1^ mRNA). At 24 h, 0.1–0.25 mL blood was collected from the retro-orbital sinus into microcentrifuge tubes. Samples were allowed to clot at room temperature for 2–4 h and centrifuged at 3000*g* for 4 min at 4 °C. Serum hEPO levels in the supernatant were measured using a human EPO ELISA kit (Jiangsu Meimian Industrial Co., Ltd.) according to the manufacturer’s instructions.

### Cre-mediated recombination in mTmG reporter mice

mTmG mice were injected intravenously with Cre mRNA-LNPs (0.25 mg kg^−1^ mRNA) via the tail vein. Three days later, mice were anesthetized and euthanized by isoflurane overdose. Major organs were harvested and imaged using the IVIS system in GFP fluorescence mode. Tissues were then flash-frozen in liquid nitrogen. Frozen sectioning and DAPI staining were performed by Wuhan Servicebio Technology Co., Ltd., and slide scanning was conducted by Shenzhen Shengqiang Technology Co., Ltd.

### In vivo therapeutic *Ttr* gene knockdown via LNP-mediated CRISPR–Cas9 delivery in C57BL/6 mice

To evaluate the therapeutic potential of the ML-prioritized LNPs, Cas9 mRNA and sgRNA targeting the *Ttr* gene (sequence: mUmUm*ACAGCCACGUCUACAGCAGUUUUAGAGCUAGAAAUAGCAAGUUAAAAUAA GGCUAGUCCGUUAUCAACUUGAAAAAGUGGCACCGAGUCGGUGCU*mUmUmU) were co-encapsulated at a 5:1 mass ratio. C57BL/6 mice were intravenously administered via the tail vein using a 29 G insulin syringe with a dose of 0.25 mg/kg Cas9 mRNA (plus the corresponding weight of sgRNA), while the control group received LNPs encapsulating Cas9 mRNA only. The administration followed a 2-day consecutive injection schedule. On day 7 post-initial injection, mice were anesthetized with isoflurane and euthanized via CO_2_ inhalation, followed by the rapid harvest and flash-freezing of liver tissues in liquid nitrogen. Transcriptional disruption was quantified via qPCR to determine relative Ttr mRNA levels using the following primers: *Ttr-S* (5′-CTGCTGTAGACGTGGCTGTA-3′), *Ttr-A* (5′-ATGGAACGGGGAAATGCCAA-3′), and Gapdh as the internal reference (S: 5′-CCTCGTCCCGTAGACAAAATG-3′, A: 5′-TGAGGTCAATGAAGGGG TCGT-3′). Systemic protein knockdown was assessed using a Mouse TTR ELISA Kit (Cat. No. LV30603, Animalunion Biotechnology Co., Ltd.). For histological visualization of mRNA knockdown, liver cryosections were subjected to fluorescence in situ hybridization (FISH). To maximize the detection sensitivity and targeting efficiency of Ttr transcripts, a dual-probe hybridization strategy was employed using a mixture of two specific sequences: 5′-TTCTAGCTCTAAAACTGCTGTAGACGTG-3′ and 5′-TTTCAAGTTGATAACGGACTAGCCTTA-3′.

### Chemicals and reagents

All starting materials for lipid synthesis, including amines, acrylates, degradable aldehydes, 2-iminothiolane hydrochloride (Traut’s reagent), triethylamine (TEA), acetic acid, ethanol, dichloromethane (DCM), sodium triacetoxyborohydride (STAB), sodium bicarbonate (NaHCO₃), and sodium chloride (NaCl), were purchased from commercial suppliers (Aladdin, J&K Scientific, Macklin, Ichemco, Shanghai Yuanye Bio-Technology, Bide Pharmatech, Adamas-beta) and used as received. Saturated aqueous NaHCO₃ and NaCl solutions were prepared in-house.

Messenger RNAs (Fluc, Cre, mCherry, hEPO, Cas9) and Ttr sgRNA were synthesized by Nanjing GenScript Biotechnology Co., Ltd. Helper lipids 1,2-distearoyl-sn-glycero-3-phosphocholine (DSPC), cholesterol, 1,2-dimyristoyl-rac-glycero-3-methoxypolyethylene glycol-2000 (PEG-DMG), DSPE-PEG-maleimide, and NBD-PE were obtained from MedChemExpress (MCE). 1,2-Dioleoyl-sn-glycero-3-phosphoethanolamine-N-(lissamine rhodamine B sulfonyl) (18:1 Liss Rhod PE) and fetal bovine serum (FBS, Cat. No. F8318) were purchased from Sigma-Aldrich. The lipophilic dye DiD and Triton X-100 were purchased from Beyotime Biotechnology. LysoTracker Red was obtained from Thermo Fisher Scientific.

For flow cytometry, Brilliant Violet 605™ anti-mouse CD31 (Cat. No. 102427), PE/Cyanine7 anti-mouse CD45 (Cat. No. 103113), Pacific Blue™ anti-mouse F4/80 (Cat. No. 123123), and the viability dye DRAQ7™ (Cat. No. 424001) were purchased from BioLegend. ELISA kits for mouse IL-1β (Cat. No. 88-7013A-88), TNF-α (Cat. No. 88-7324-88), IL-6 (Cat. No. 88-7064-88), and CCL2/MCP-1 (Cat. No. 88-7391-88) were purchased from Thermo Fisher Scientific. The Mouse CXCL1/KC ELISA kit (Cat. No. EK296/2-96) was obtained from MultiSciences (Lianke) Biotech Co., Ltd.

### Preparation of mRNA-loaded lipid nanoparticles

LNPs encapsulating Fluc, hEPO, Cre, or Cas9/sgTtr mRNA were formulated by microfluidic mixing. The organic phase contained the ionizable lipid, DSPC, cholesterol, and PEG-DMG at a molar ratio of 50:10:38.5:1.5 dissolved in ethanol. The aqueous phase contained mRNA in 10 mM sodium citrate buffer (pH 4.5).

Organic and aqueous streams were combined using a microfluidic mixer at flow rates of 3 mL min⁻¹ and 9 mL min⁻¹, respectively (ethanol:aqueous volume ratio 1:3). LNPs were concentrated and diafiltered using Amicon® Ultra centrifugal filters (4 mL, 30 kDa MWCO; Millipore Sigma) with at least three wash cycles to remove ethanol and exchange into the final buffer. Suspensions were sterile-filtered (0.22 μm), adjusted to a total lipid concentration of 1 mg mL⁻¹, and stored at 4 °C until use.

### Quantitative PCR for recombination efficiency

Genomic DNA was extracted from liver tissue and analyzed by qPCR. Total copy number was determined using primers targeting the mG fragment (mG-Forward: 5′-GTCTATATCATGGCCGACAAGCA-3′; mG-Reverse: 5′-ATGTGATCGCGCTTCTCGTT-3′). Recombined alleles were quantified using primers spanning the two loxP sites (loxP-Forward: 5′-ATTGATTTGATACCGCGGGC-3′; loxP-Reverse: 5′-TTCACGTGGCCATTCTCCTGT-3′), which amplify only after Cre-mediated excision. Actin served as the internal reference (Actin-Forward: 5′-TGAGGCTGGTGATAAGTGGC-3′; Actin-Reverse: 5′-AAAGCCACAAGAAACACTCAGGG-3′). Recombination rates were normalized to actin.

### Physicochemical and structural characterization of LNPs

Hydrodynamic diameter, polydispersity index (PDI), and zeta potential were measured with a Zetasizer Nano ZS90 (Malvern Instruments). Chemical structures of selected lipids were confirmed by ¹H and ¹³C NMR (Bruker 400 MHz) and high-resolution quadrupole time-of-flight mass spectrometry (G2-S Q-TOF, Waters). LNP morphology was visualized by cryogenic TEM (Talos L120C, Thermo Fisher Scientific) and conventional TEM.

mRNA encapsulation efficiency (EE%) was determined using Quant-iT™ RiboGreen™ RNA reagent (Beijing Solarbio Science & Technology Co., Ltd.) following the manufacturer’s protocol. Apparent pKa values of LNPs were measured using a 6-(p-toluidinyl)naphthalene-2-sulfonic acid (TNS) fluorescence assay.

### Hemolysis assay

Red blood cells (RBCs) were isolated from fresh C57BL/6 mouse blood by centrifugation at 3000*g* for 3 min and washed three times with PBS (pH 7.4). RBC pellets were resuspended in PBS (pH 7.4) or sodium citrate buffer (pH 6.0).

LNPs were incubated with RBC suspensions at various lipid concentrations in 96-well plates at 37 °C and 5% CO₂ for 1 h. Plates were centrifuged, and 60 μL supernatant was transferred to new plates. Hemoglobin release was quantified at 540 nm using a microplate reader. RBCs in PBS served as negative controls; RBCs treated with 1 wt% Triton X-100 served as positive controls.

### Lipid fusion assay via FRET

Endosome-mimicking anionic liposomes were composed of 1,2-dioleoyl-sn-glycero-3-phospho-L-serine (DOPS): 1,2-dioleoyl-sn-glycero-3-phosphocholine (DOPC): 1,2-dioleoyl-sn-glycero-3-phosphoethanolamine (DOPE): N-(7-nitrobenz-2-oxa-1,3-diazol-4-yl)-phosphoethanolamine (NBD-PE): rhodamine-phosphoethanolamine (Rho-PE) at a molar ratio of 25:25:48:1:1. Lipids were dissolved in DCM, evaporated to form a thin film, and vacuum-dried for 2 h. Films were hydrated with PBS (pH 7.4) and sonicated for 30 min. Liposomes were concentrated to ~1 mg mL⁻¹ using 30 kDa centrifugal filters.

For fusion assays, 90 μL buffer (sodium citrate, pH 6.0, or PBS, pH 7.4) was added to black 96-well plates, followed by 5 μL anionic liposomes and 5 μL candidate LNPs (1 mg mL⁻¹). NBD fluorescence de-quenching, reflecting NBD/Rho separation upon fusion, was recorded every 1 min for 60 min at 37 °C using excitation/emission of 465/520 nm. Liposomes with PBS and 1 wt% Triton X-100 served as negative and positive controls, respectively.

### Molecular dynamics simulations

MD simulations were performed using GROMACS 2025.02. Lipids were parameterized with the AMBER19SB–Lipid21 force field; RNA used AMBER19SB–OL3, and OPC3 water was employed. RESP charges for lipids were obtained using ORCA 6.1.0 at the B3LYP-D3(BJ)/Def2TZVP level.

Lipid bilayer simulations were run in the NPT ensemble (298 K, 1 bar) for 250 ns. Conformations from the final 50 ns were clustered (RMSD threshold 1 Å), and the dominant cluster was used to calculate the packing parameter (P).

For lipid–RNA complexes, a 20-mer RNA (sequence: GACUGACUGACUGACUGACU) was constructed in Avogadro, assembled with lipids using Packmol, and simulated under NPT conditions (298 K, 1 bar) for 250 ns.

### Blood collection and ELISA

For ELISA assays, blood was collected from the retro-orbital sinus using EDTA-treated capillary tubes (for cytokines) or into plain tubes (for hEPO, TTR). Samples were transferred to 1.5 mL tubes and incubated at 4 °C for 4–6 h, then centrifuged at 3000*g* for 4 min at 4 °C. Serum was collected, aliquoted, and stored at −80 °C until analysis. Cytokines (TNF-α, IL-1β, IL-6, CXCL1, MCP-1) and serum TTR were quantified using the indicated ELISA kits according to the manufacturers’ protocols.

### Machine-learning model training and optimization

The machine-learning task was formulated as binary classification using in vitro Fluc mRNA expression measured in HeLa cells. The three activity categories were used to visualize the screening distribution, whereas model training used an operational cutoff of 2000 RLU for candidate prioritization. Lipids with signals <2000 RLU were assigned to the low-activity class (class 0), and those with signals ≥2000 RLU to the active class (class 1). The dataset comprised 520 AMD Head–Tail combinations, including 127 class-0 and 393 class-1 samples.

Random Forest, XGBoost, LightGBM, and Logistic Regression were evaluated using identical molecular representations and preprocessing. For each predefined Head and Tail module, a 2048-bit Morgan fingerprint (radius 2) and 208 physicochemical descriptors were calculated using RDKit. Fixed-order concatenation of the fingerprint and descriptor vectors generated a 4512-dimensional representation for each Head–Tail combination. Zero-variance features were removed using VarianceThreshold (threshold = 0.0), followed by Z-score standardization with StandardScaler. All preprocessing, including variance filtering, scaling, and class-imbalance handling, was fitted exclusively on training data and applied unchanged to held-out data to prevent information leakage.

Model performance was assessed using accuracy, positive-class F1, macro-F1, weighted F1, ROC-AUC, and PR-AUC. Because the objective was candidate enrichment under class imbalance, macro-F1 and PR-AUC were emphasized during model comparison. Class imbalance was addressed within training folds using SMOTE-like oversampling, with class weighting evaluated as an alternative strategy. Generalization beyond random interpolation within the combinatorial AMD matrix was assessed using Head-grouped, Tail-grouped, and strict double-cold validation. Identical Head modules, Tail modules, or both modules, respectively, were mutually exclusive between training and held-out sets. Based on its overall performance across the revised metrics under the unified preprocessing and validation workflow, XGBoost was selected for virtual-library prediction and candidate prioritization.

### Flow cytometric analysis of hepatic cell populations

To evaluate cell-type-resolved functional Cre mRNA delivery in the liver of Cre mRNA-LNPs, mTmG mice received intravenous injections of Cre mRNA–LNPs (0.25 mg kg^−1^). Three days later, mice were anesthetized, and livers were perfused via the portal vein with PBS containing 0.1% BSA and 0.2% EDTA, followed by perfusion with digestion buffer (DMEM with 0.5 mg mL^−1^ collagenase IV and 50 μg mL⁻¹ DNase I). Mice were then euthanized by isoflurane overdose. Livers were minced and incubated in serum-free DMEM with collagenase IV at 37 °C for 3–6 min with gentle agitation and pipetting. Cell suspensions were filtered through 40 μm strainers and centrifuged at 800*g* for 4 min. Pellets were resuspended in PBS, and RBCs were lysed with ACK buffer (1 mL; Thermo Fisher Scientific) for 6 min, followed by centrifugation at 800*g* for 4 min.

Single-cell suspensions were resuspended in PBS at 8 × 10⁵–1 × 10⁶ cells mL⁻¹. For staining, 200 μL of suspension was incubated with anti-CD31, anti-CD45, and anti-F4/80 antibodies (diluted 1:100, 2 μL per antibody) for 20–30 min at 4 °C in the dark. After washing with PBS, cells were resuspended in PBS containing 0.1% DRAQ7™ to exclude dead cells. Data were acquired on a CytoFLEX flow cytometer (Beckman Coulter) and analyzed in FlowJo (v10.8.1). Samples were sequentially gated to identify cells, singlets, and viable DRAQ7-negative cells. Among viable cells, hepatocyte-enriched cells were defined as CD31⁻CD45⁻, liver sinusoidal endothelial cells (LSECs) as CD31⁺CD45⁻, immune cells as CD45⁺, and Kupffer cells as F4/80⁺ cells within the CD45⁺ compartment. EGFP-positive fractions were subsequently quantified to assess Cre-dependent functional mRNA delivery.

### Cell culture

HeLa (ATCC CCL-2) and HepG2 (ATCC CRL-10741) cells were cultured in DMEM, and HEK293T cells (ATCC CRL-3216) were cultured in MEM (Gibco), each supplemented with 10% FBS and 1% penicillin–streptomycin. Cells were maintained at 37 °C in a humidified 5% CO₂ incubator.

### Ethical statement

All animal procedures were approved by the Institutional Animal Care and Use Committee of Tsinghua Shenzhen International Graduate School (SIGS), Tsinghua University (#2025F147), and conducted in accordance with institutional guidelines. mTmG mice were kindly provided by Dr. Deqiang Kong. C57BL/6 mice (6–8 weeks old) were purchased from Zhuhai BesTest Bio-Tech Co., Ltd. (distributed by Guangdong Jinzhihe Biotechnology Co., Ltd.). Animals were housed in SPF facilities (22 ± 2 °C; 40–70% humidity; 12 h light/12 h dark cycle) with ad libitum access to chow and water. Animal health was monitored routinely by veterinary staff. Both male and female mice were included. Sex was recorded for each animal; however, the study was not designed or powered for sex-stratified analysis, and sex was not used as an experimental factor.

### Statistics and reproducibility

No statistical method was used to predetermine sample size. Sample sizes were selected based on established experimental practice and previous studies using comparable assays. No data were excluded from the analyses. Animals were randomly assigned to the treatment groups. Animal experiments were conducted using a double-blind design, in which both the investigators conducting the experiments and those assessing the outcomes were blinded to group allocation until completion of data collection and analysis. Quantitative data are presented as mean ± SD. Statistical significance was assessed using one-way ANOVA followed by Tukey’s multiple-comparisons test, as specified in the corresponding figure legends. All pairwise comparisons were included in the analysis, although only selected comparisons are annotated in the figures for clarity. Reported *P* values were adjusted for multiple comparisons. The exact sample sizes and the biological and technical replicates used in the animal experiments are specified in the corresponding figure legends.

### Reporting summary

Further information on research design is available in the Nature Portfolio Reporting Summary linked to this article.

## Supplementary information

Supplementary Information

Description of Additional Supplementary Files

Supplementary Data 1

Supplementary Data 2

Reporting Summary

Transparent Peer Review file

## Source data

Source Data

## Data Availability

Source data underlying the main and Supplementary Figs. are provided with this paper. The Source Data file includes the numerical data for the corresponding figures and the dataset used for the machine-learning analyses, organized in separate worksheets. Source data are provided with this paper.

## Code availability

The code used for molecular featurization, preprocessing, model training, validation, SHAP analysis, and virtual-library prioritization is available at https://github.com/thuZHANGZhen/AMD-PAMD-lipid-ML-code.

## References

[1] Xu, Y. et al. Chemistry, manufacturing and controls strategies for using novel excipients in lipid nanoparticles. *Nat. Nanotechnol.* **20**, 331–344 (2025). 10.1038/s41565-024-01833-9 39821140

[2] Sharma, P., Breier, D. & Peer, D. Immunogenic amines on lipid nanoparticles. *Nat. Biomed. Eng.* **8**, 1332–1333 (2024). 10.1038/s41551-024-01265-9 39443742

[3] Melamed, J. R. et al. Ionizable lipid nanoparticles deliver mRNA to pancreatic β cells via macrophage-mediated gene transfer. *Sci. Adv.* **9**, eade1444 (2023). 10.1126/sciadv.ade1444 PMC9882987 36706177

[4] Kasiewicz, L. N. et al. GalNAc-lipid nanoparticles enable non-LDLR dependent hepatic delivery of a CRISPR base editing therapy. *Nat. Commun.* **14**, 2776 (2023). 10.1038/s41467-023-37465-1 PMC10185539 37188660

[5] Zhao, S. et al. Acid-degradable lipid nanoparticles enhance the delivery of mRNA. *Nat. Nanotechnol.* **19**, 1702–1711 (2024). 10.1038/s41565-024-01765-4 PMC12479011 39179796

[6] Padilla, M. S. et al. Elucidating lipid nanoparticle properties and structure through biophysical analyses. *Nat. Biotechnol*. 10.1038/s41587-025-02855-x (2025). 41131152

[7] Lin, Y. et al. Tissue-specific mRNA delivery and prime editing with peptide–ionizable lipid nanoparticles. *Nat. Mater.* **25**, 133–145 (2026). 10.1038/s41563-025-02320-9 40890498

[8] Vaidya, A. et al. Expanding RNAi to kidneys, lungs, and spleen via selective organ targeting (SORT) siRNA lipid nanoparticles. *Adv. Mater.* **36**, 2313791 (2024). 10.1002/adma.202313791 PMC11823468 38973655

[9] Bitounis, D. & Amiji, M. M. Lipid nanoparticles target haematopoietic stem cells. *Nat. Nanotechnol.* **19**, 1249–1250 (2024). 10.1038/s41565-024-01682-6 38849545

[10] Fei, Y. et al. Simplified lipid nanoparticles for tissue- and cell-targeted mRNA delivery facilitate precision tumor therapy in a lung metastasis mouse model. *Adv. Mater.* **36**, 2409812 (2024). 10.1002/adma.202409812 39390844

[11] Xue, L. et al. Combinatorial design of siloxane-incorporated lipid nanoparticles augments intracellular processing for tissue-specific mRNA therapeutic delivery. *Nat. Nanotechnol.* **20**, 132–143 (2025). 10.1038/s41565-024-01747-6 PMC12207990 39354147

[12] Liu, Z. et al. Structure-guided design of endosomolytic chloroquine-like lipid nanoparticles for mRNA delivery and genome editing. *Nat. Commun.* **16**, 4241 (2025). 10.1038/s41467-025-59501-y PMC12058976 40335474

[13] Omo-Lamai, S. et al. Physicochemical targeting of lipid nanoparticles to the lungs induces clotting: mechanisms and solutions. *Adv. Mater.* **36**, 2312026 (2024). 10.1002/adma.202312026 PMC11209818 38394670

[14] Kang, D. D. et al. Targeting and tracking mRNA lipid nanoparticles at the particle, transcript and protein level. *Nat. Biomed. Eng.* **9**, 1591–1609 (2025). 10.1038/s41551-025-01511-8 PMC12673502 41102346

[15] Gautam, M. et al. Lipid nanoparticles with PEG-variant surface modifications mediate genome editing in the mouse retina. *Nat. Commun.* **14**, 6468 (2023). 10.1038/s41467-023-42189-3 PMC10575971 37833442

[16] Han, X. et al. Ligand-tethered lipid nanoparticles for targeted RNA delivery to treat liver fibrosis. *Nat. Commun.* **14**, 75 (2023). 10.1038/s41467-022-35637-z PMC9845313 36650129

[17] Cao, D. et al. Dynamically covalent lipid nanoparticles mediate CRISPR–Cas9 genome editing against choroidal neovascularization in mice. *Sci. Adv.* **11**, eadj0006 (2025). 10.1126/sciadv.adj0006 PMC12248289 40644543

[18] Gao, Q. et al. Reinforced plant-derived lipid nanoparticles for oral precise epigenome editing in colonic diseases. *Sci. Adv.* **11**, eadw9275 (2025). 10.1126/sciadv.adw9275 PMC12466914 41004579

[19] Feng, Y., Yu, G. & Chen, X. Albumin-coated lipid nanoparticles are effective delivery systems for mRNA vaccines. *Nat. Mater.* **24**, 1692–1693 (2025). 10.1038/s41563-025-02285-9 40813915

[20] Qi, S. et al. Supramolecular lipid nanoparticles based on host–guest recognition: a new generation delivery system of mRNA vaccines for cancer immunotherapy. *Adv. Mater.* **36**, 2311574 (2024). 10.1002/adma.202311574 38433564

[21] Xu, C. et al. Slimming and reinvigorating tumor-associated dendritic cells with hierarchical lipid rewiring nanoparticles. *Adv. Mater.* **35**, 2211415 (2023). 10.1002/adma.202211415 37096955

[22] Fu, C. et al. Macrophage-targeted Mms6 mRNA-lipid nanoparticles promote locomotor functional recovery after traumatic spinal cord injury in mice. *Sci. Adv.* **11**, eads2295 (2025). 10.1126/sciadv.ads2295 PMC11939073 40138430

[23] Liu, Y. et al. Generation of tolerogenic antigen-presenting cells in vivo via the delivery of mRNA encoding PDL1 within lipid nanoparticles. *Nat. Biomed. Eng.* **9**, 1320–1334 (2025). 10.1038/s41551-025-01373-0 40155762

[24] Xue, L. et al. High-throughput barcoding of nanoparticles identifies cationic, degradable lipid-like materials for mRNA delivery to the lungs in female preclinical models. *Nat. Commun.* **15**, 1884 (2024). 10.1038/s41467-024-45422-9 PMC10904786 38424061

[25] Rhym, L. H. et al. Peptide-encoding mRNA barcodes for the high-throughput in vivo screening of libraries of lipid nanoparticles for mRNA delivery. *Nat. Biomed. Eng.* **7**, 901–910 (2023). 10.1038/s41551-023-01030-4 37127709

[26] Jin, H. et al. Premixing enables loading of long RNA in cubic phase lipid nanoparticles. *Nat. Commun.* **16**, 5054 (2025). 10.1038/s41467-025-60380-6 PMC12125232 40447600

[27] Son, G. et al. Fusogenic lipid nanoparticles for rapid delivery of large therapeutic molecules to exosomes. *Nat. Commun.* **16**, 4799 (2025). 10.1038/s41467-025-59489-5 PMC12102247 40410169

[28] Pires, I. S. et al. Charge-stabilized nanodiscs as a new class of lipid nanoparticles. *Adv. Mater.* **36**, 2408307 (2024). 10.1002/adma.202408307 PMC11681300 39543433

[29] Papadopoulou, P. et al. Phase-separated lipid-based nanoparticles: selective behavior at the nano-bio interface. *Adv. Mater.* **36**, 2310872 (2024). 10.1002/adma.202310872 37988682

[30] Renzi, S. et al. Structuring lipid nanoparticles, DNA, and protein corona into stealth bionanoarchitectures for in vivo gene delivery. *Nat. Commun.* **15**, 9119 (2024). 10.1038/s41467-024-53569-8 PMC11496629 39438484

[31] Voke, E. et al. Protein corona formed on lipid nanoparticles compromises delivery efficiency of mRNA cargo. *Nat. Commun.* **16**, 8699 (2025). 10.1038/s41467-025-63726-2 PMC12485112 41027853

[32] Chan, A. et al. Designing lipid nanoparticles using a transformer-based neural network. *Nat. Nanotechnol.* **20**, 1491–1501 (2025). 10.1038/s41565-025-01975-4 PMC12534189 40817189

[33] Simonsen, J. B. Technical challenges of studying the impact of plasma components on the efficacy of lipid nanoparticles for vaccine and therapeutic applications. *Nat. Commun.* **15**, 3852 (2024). 10.1038/s41467-024-47724-4 PMC11082148 38724528

[34] Degtev, D. et al. Engineered PsCas9 enables therapeutic genome editing in mouse liver with lipid nanoparticles. *Nat. Commun.* **15**, 9173 (2024). 10.1038/s41467-024-53418-8 PMC11544209 39511150

[35] Witten, J. et al. Artificial intelligence-guided design of lipid nanoparticles for pulmonary gene therapy. *Nat. Biotechnol.* **43**, 1790–1799 (2025). 10.1038/s41587-024-02490-y PMC12149338 39658727

[36] Estabrook, D. A. et al. Buffer optimization of siRNA-lipid nanoparticles mitigates lipid oxidation and RNA-lipid adduct formation. *Nat. Commun.* **16**, 8380 (2025). 10.1038/s41467-025-63651-4 PMC12462517 40998813

[37] Su, K. et al. Reformulating lipid nanoparticles for organ-targeted mRNA accumulation and translation. *Nat. Commun.* **15**, 5659 (2024). 10.1038/s41467-024-50093-7 PMC11226454 38969646

[38] Sarode, A. et al. Potent liver-tropic mRNA lipid nanoparticles: ApoE-mediated delivery through a low-density lipoprotein receptor-independent uptake mechanism. *Adv. Mater.* **38**, e17893 (2026). 10.1002/adma.202517893 PMC13088227 41316820

[39] Smith, A. R. et al. Transient growth factor expression via mRNA in lipid nanoparticles promotes hepatocyte cell therapy in mice. *Nat. Commun.* **15**, 5010 (2024). 10.1038/s41467-024-49332-8 PMC11169405 38866762

[40] Han, X. et al. Fast and facile synthesis of amidine-incorporated degradable lipids for versatile mRNA delivery in vivo. *Nat. Chem.* **16**, 1687–1697 (2024). 10.1038/s41557-024-01557-2 PMC11446653 38982196

[41] Breda, L. et al. In vivo hematopoietic stem cell modification by mRNA delivery. *Science* **381**, 436–443 (2023). 10.1126/science.ade6967 PMC10567133 37499029

[42] Sun, Y. et al. In vivo editing of lung stem cells for durable gene correction in mice. *Science* **384**, 1196–1202 (2024). 10.1126/science.adk9428 PMC12208706 38870301

[43] Hunter, T. L. et al. In vivo CAR T cell generation to treat cancer and autoimmune disease. *Science* **388**, 1311–1317 (2025). 10.1126/science.ads8473 40536974

[44] Friedrich, M. J. et al. Transient hepatic reconstitution of trophic factors enhances aged immunity. *Nature* **650**, 481–489 (2026). 10.1038/s41586-025-09873-4 PMC12893904 41407851

[45] Zhang, L.-F. et al. Vicious cycle-breaking lipid nanoparticles remodeling multicellular crosstalk to reverse liver fibrosis. *Adv. Mater.* **36**, 2311474 (2024). 10.1002/adma.202311474 38194906

[46] Swingle, K. L. et al. Placenta-tropic VEGF mRNA lipid nanoparticles ameliorate murine pre-eclampsia. *Nature* **637**, 412–421 (2025). 10.1038/s41586-024-08291-2 PMC12205896 39663452

