# Supplementary material for: Interpretable AI-guided discovery of degradable ionizable lipids with enhanced hepatic mRNA delivery in vivo
Source: Nat Commun. 2026 Oct 12;17:11561. doi: 10.1038/s41467-026-78635-1 (PMC13748640; doi:10.1038/s41467-026-78635-1)
Supplement: Supplementary file 1 [file 41467_2026_78635_MOESM1_ESM.pdf]

Supplementary Information

Interpretable AI-guided discovery of degradable ionizable lipids with enhanced hepatic mRNA delivery in vivo

Zhen Zhang[1, #], Yongbo Ren[1, #], Deqiang Kong[1, 2], Jindong Feng[3], Kaiyi Hu[4], Xinyun Xu[5], Fukang Deng[6], Peixia Li[1], Jiuao Wang[1], Wenhui Huang[7], Guodan Wei[1], Yang Su[1], Ying Tan[1], Shuixing Zhang[7], Xiaodan Sun[8], Feiyu Kang[1, 8, *], Hao Yan[1, *]

[1] *Tsinghua Shenzhen International Graduate School, Tsinghua University, Shenzhen, 518055, China*

[2] *Research Institute of Tsinghua, Pearl River Delta, Guangzhou, 510530, China*

[3] *Department of Biomedical Engineering, Boston University, Boston, MA 02215, USA*

[4] *College of Engineering, Georgia Institute of Technology, Atlanta, GA 30332, USA*

[5] *State Key Laboratory of Advanced Welding and Joining of Materials and Structures, School of Materials Science and Engineering, Harbin Institute of Technology (Shenzhen), Shenzhen, 518055, China*

[6] *School of Light Industry and Food Engineering, Guangxi University, Nanning, 530004, China*

[7] *Medical Imaging Center, The First Affiliated Hospital of Jinan University, Guangzhou, 510630, China*

[8] *Key Laboratory of New Ceramics and Fine Processing, Key Laboratory of Advanced Materials of Ministry of Education of China, School of Materials Science and Engineering, Tsinghua University, Beijing, 100084, China*

# Zhen Zhang and Yongbo Ren contributed equally to this work.

* Corresponding Author Emails: fykang@sz.tsinghua.edu.cn, hyan23@sz.tsinghua.edu.cn

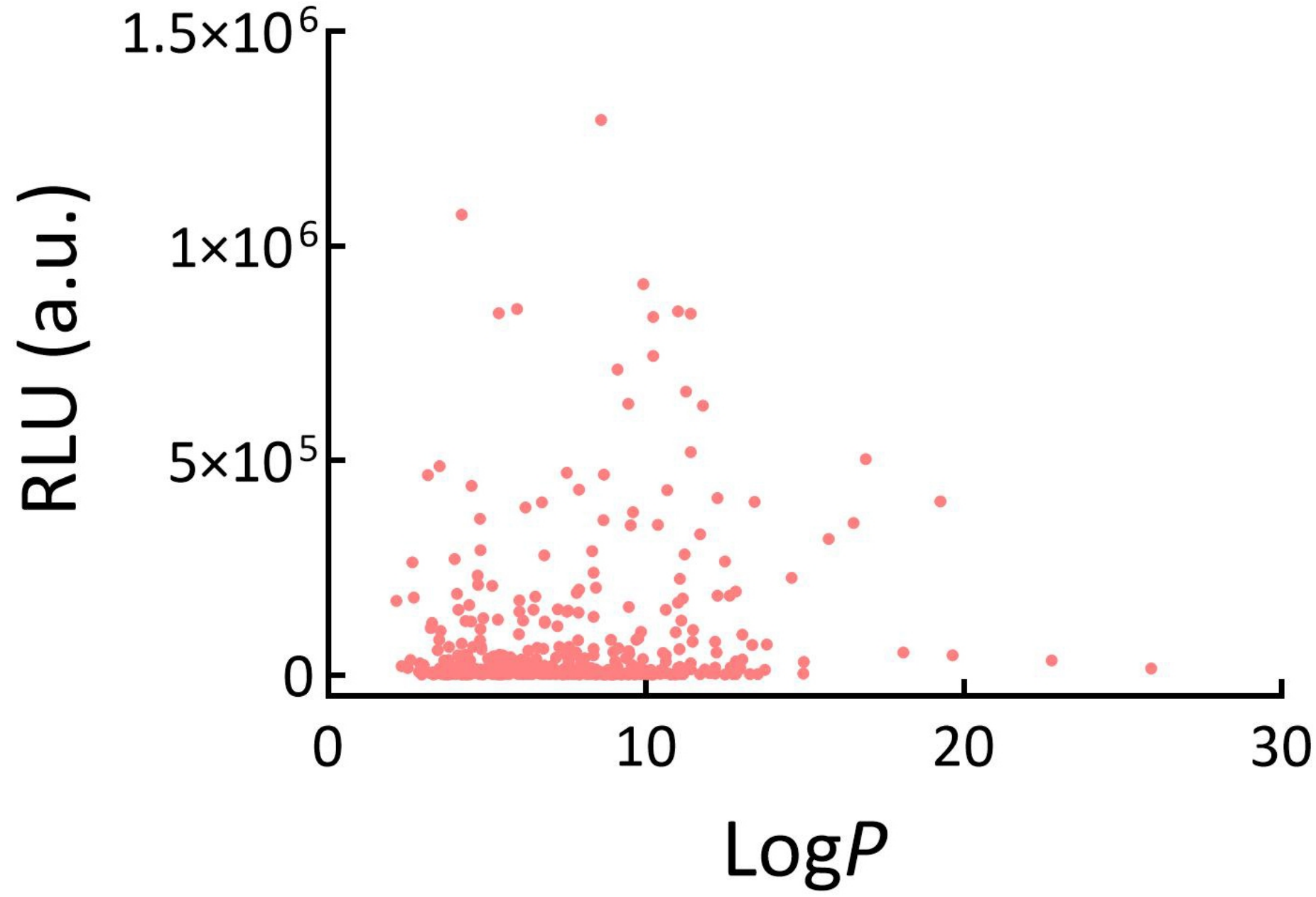

Supplementary Fig. 1. Scatter plot showing the correlation between the total lipid LogP and transfection efficiency.

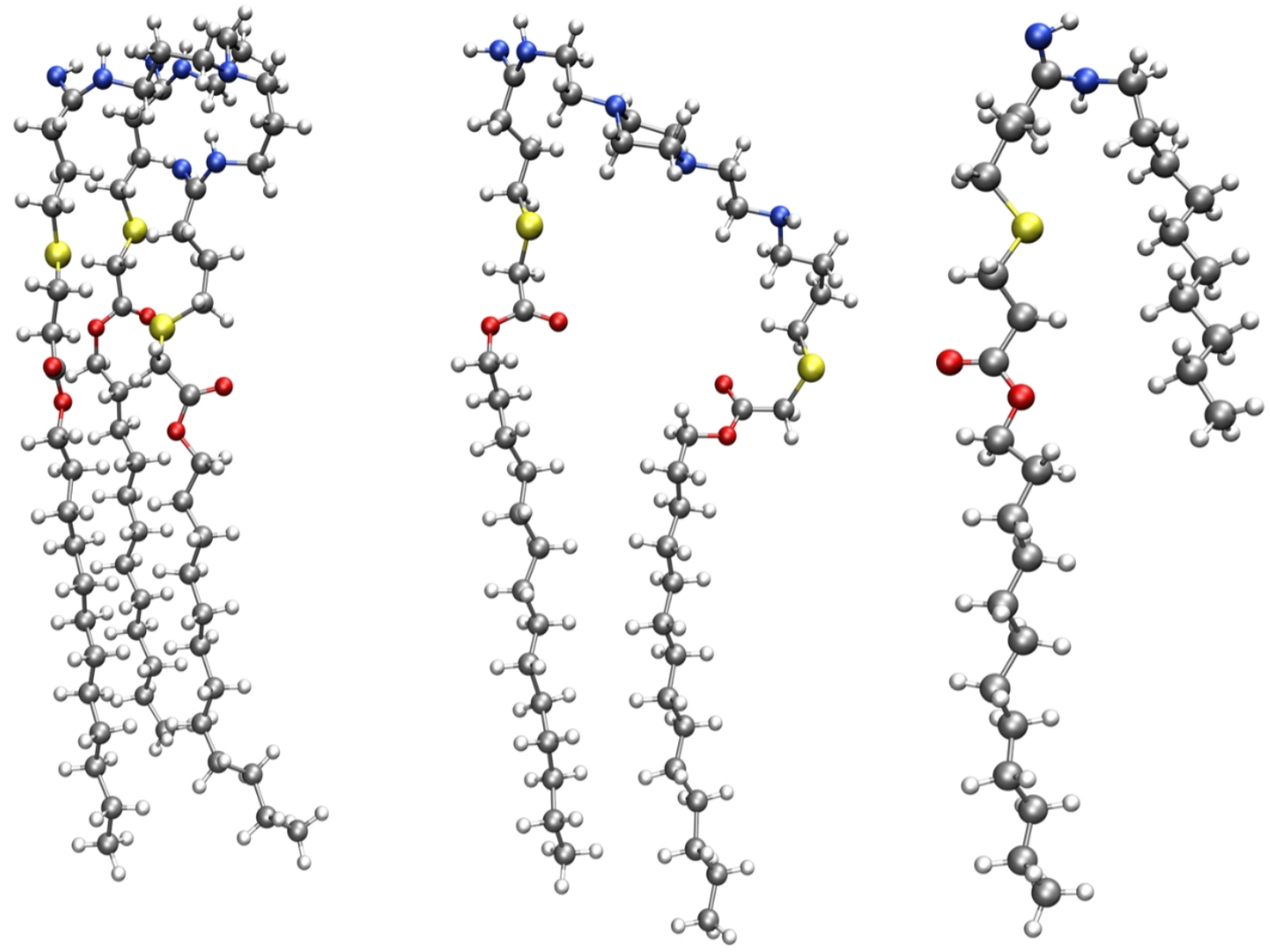

A22MD09 **A24MD10** **A36MD01**

LNP	A22MD09	A24MD10	A36MD01
**V (Å³)**	**1595.40**	**934.76**	**562.95**
**A (Å²)**	**13.85**	**15.45**	**8.74**
**L (Å¹)**	**36.86**	**14.61**	**9.96**
P	**3.12**	**4.13**	**6.47**
**Angle**	**27.27**	**31.39**	**31.37**

Supplementary Fig. 2. **Computational conformational analysis and geometric characterization of representative AMD lipids.** (**Upper**) Energy-optimized 3D molecular conformations of three selected lipid candidates (A22MD09, A24MD10 and A36MD01), visualized as ball-and-stick models. The spatial arrangements, colored by element (C, gray; H, white; N, blue; O, red; S, yellow), illustrate the diverse molecular envelopes generated by distinct combinations of amine heads and biodegradable tails. (**Lower**) Quantitative geometric descriptors derived from the energy-minimized states. The table summarizes key parameters: molecular volume (*V*), optimal headgroup area (*A*), critical lipid length (*L*), critical packing parameter (*P*, defined as $P = V / AL$), and the molecular cone angle. These parameters enable comparison of molecular geometry across representative AMD lipids.

Supplementary Table 1. Model performance under the unified preprocessing workflow.

Random Forest, XGBoost, LightGBM and Logistic Regression were evaluated using identical molecular representations, matched splits and training-data-only preprocessing. Preprocessing included RDKit descriptors, Morgan fingerprints, zero-variance feature filtering, Z-score standardization and training-fold-only SMOTE-like class balancing. Metrics include accuracy, positive-class F1, macro-F1, weighted F1, ROC-AUC and PR-AUC.

Model	Accuracy	Positive-class F1	Macro-F1	Weighted F1	ROC-AUC	PR-AUC
Random Forest	0.7788	0.8639	0.6371	0.7330	0.8009	0.9160
XGBoost	0.8654	0.9167	0.7833	0.8500	0.8590	0.9385
LightGBM	0.8077	0.8780	0.7118	0.7821	0.7932	0.9047
Logistic Regression	0.7981	0.8662	0.7272	0.7860	0.8045	0.9093

Supplementary Note 1. Scope, validation and interpretive limits of the machine-learning model.

The machine-learning model in this study was designed as a candidate-enrichment model for in vitro functional mRNA transfection activity. Although the screening landscape in Fig. 1c is displayed using three descriptive activity categories, model training was formulated as a binary classification task using HeLa-cell Fluc expression, with Fluc signals <2,000 assigned to class 0 and signals >=2,000 assigned to class 1. The model was not trained on organ distribution, liver accumulation, intrahepatic cell-type preference or hepatocyte-specific functional output. Therefore, SHAP and descriptor analyses should be interpreted as identifying molecular features associated with the HeLa-cell in vitro transfection phenotype. To reduce preprocessing-related confounding, all algorithms were compared using the same feature representation and preprocessing workflow, including RDKit descriptors, Morgan fingerprints, zero-variance feature filtering, feature standardization and training-fold-only class-imbalance handling. Head-grouped, tail-grouped and strict double-cold validation were used to evaluate generalization beyond random interpolation within the modular AMD matrix. These analyses support the use of XGBoost for candidate prioritization, but they do not establish direct prediction of hepatocyte-biased delivery. *In vivo* organ distribution and intrahepatic cell-type preference were therefore evaluated independently by bioluminescence imaging, Cre/LoxP reporter assays and flow cytometry.

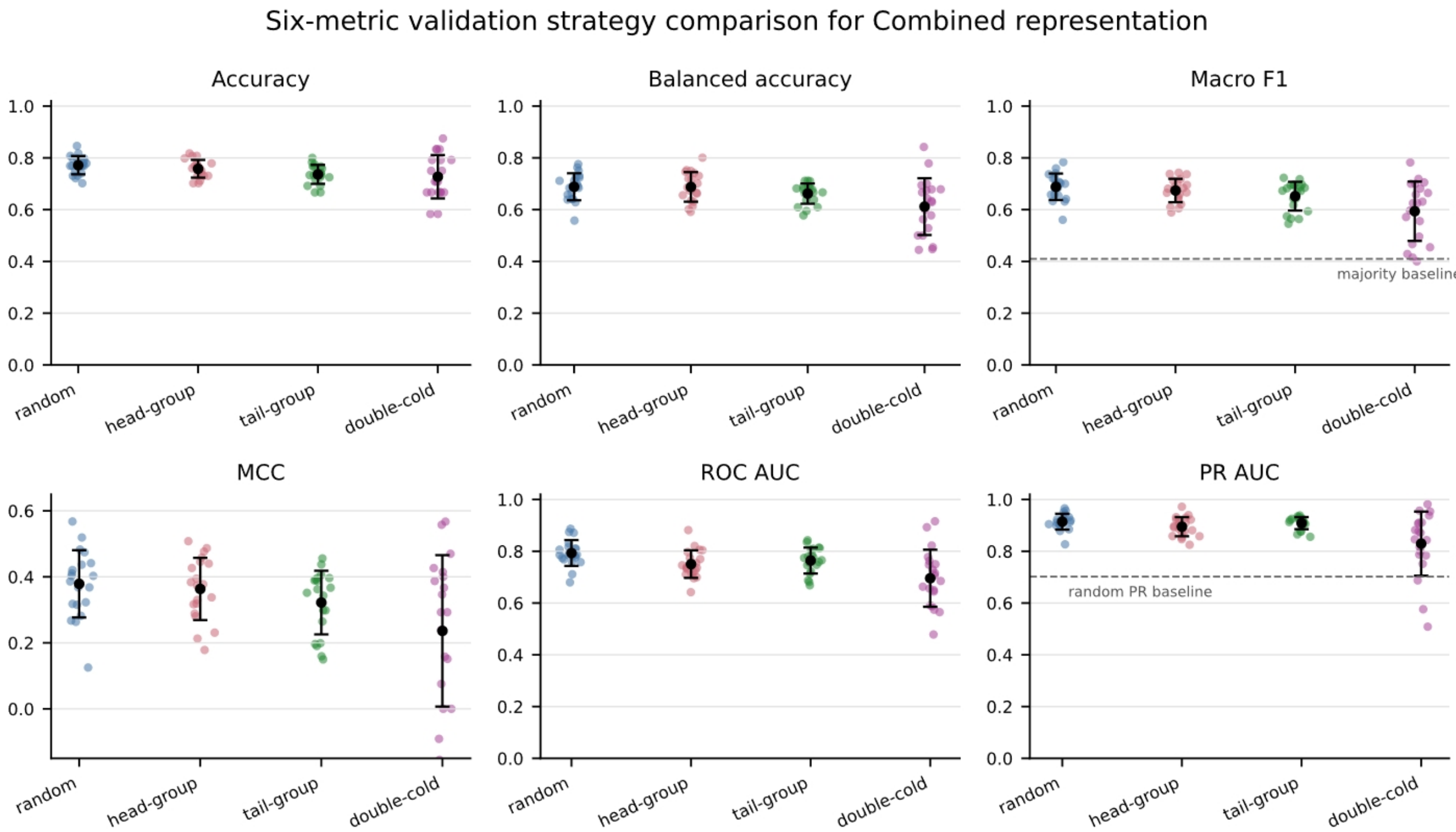

Supplementary Fig. 3. Validation-strategy comparison for the combined head–tail XGBoost model. The combined head–tail XGBoost model was evaluated under random, head-group, tail-group, and strict double-cold validation using 20 repeated splits. Six metrics are shown: accuracy, balanced accuracy, macro F1, Matthews correlation coefficient (MCC), ROC AUC, and PR AUC. In head-group and tail-group validation, identical head or tail modules, respectively, were excluded from simultaneous occurrence in the training and test sets. In strict double-cold validation, both head and tail modules in the test set were absent from the corresponding training set. Colored points represent individual validation splits. Black points and error bars indicate mean ± s.d. Dashed horizontal lines indicate the majority-class baseline for macro F1 and the prevalence-based random-ranking baseline for PR AUC.

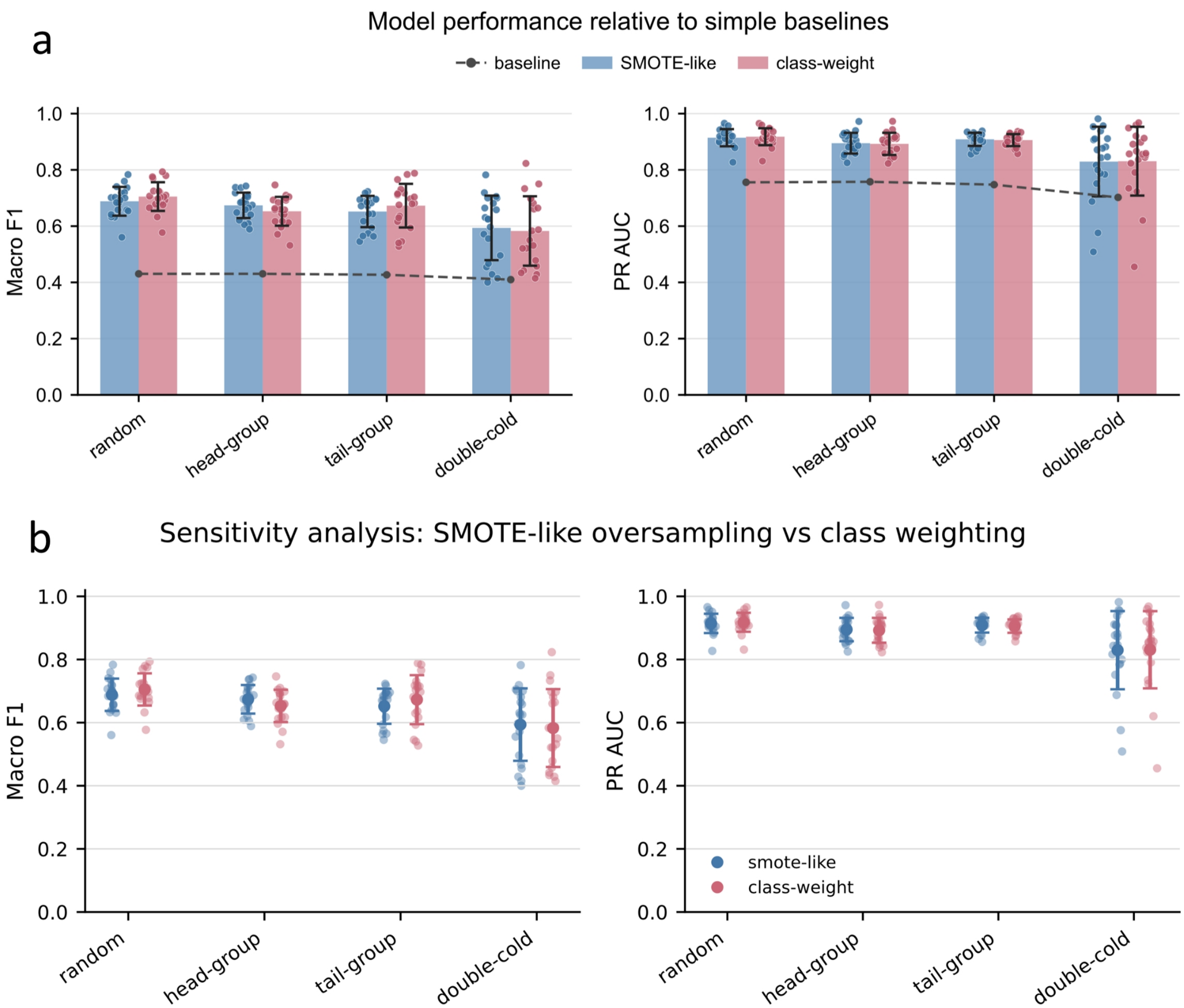

Supplementary Fig. 4. Baseline comparison and class-imbalance sensitivity analysis for the combined head–tail XGBoost model. (a) Macro F1 and PR AUC of the combined head–tail XGBoost model under random, head-group, tail-group, and strict double-cold validation using SMOTE-like oversampling or class weighting. Dashed lines indicate the corresponding simple baselines: the majority-class baseline for macro F1 and the prevalence-based random-ranking baseline for PR AUC. Bars and error bars indicate mean ± s.d. across 20 repeated splits. (b) Split-level macro F1 and PR AUC values obtained using SMOTE-like oversampling or class weighting under the four validation strategies. Each point represents one validation split.

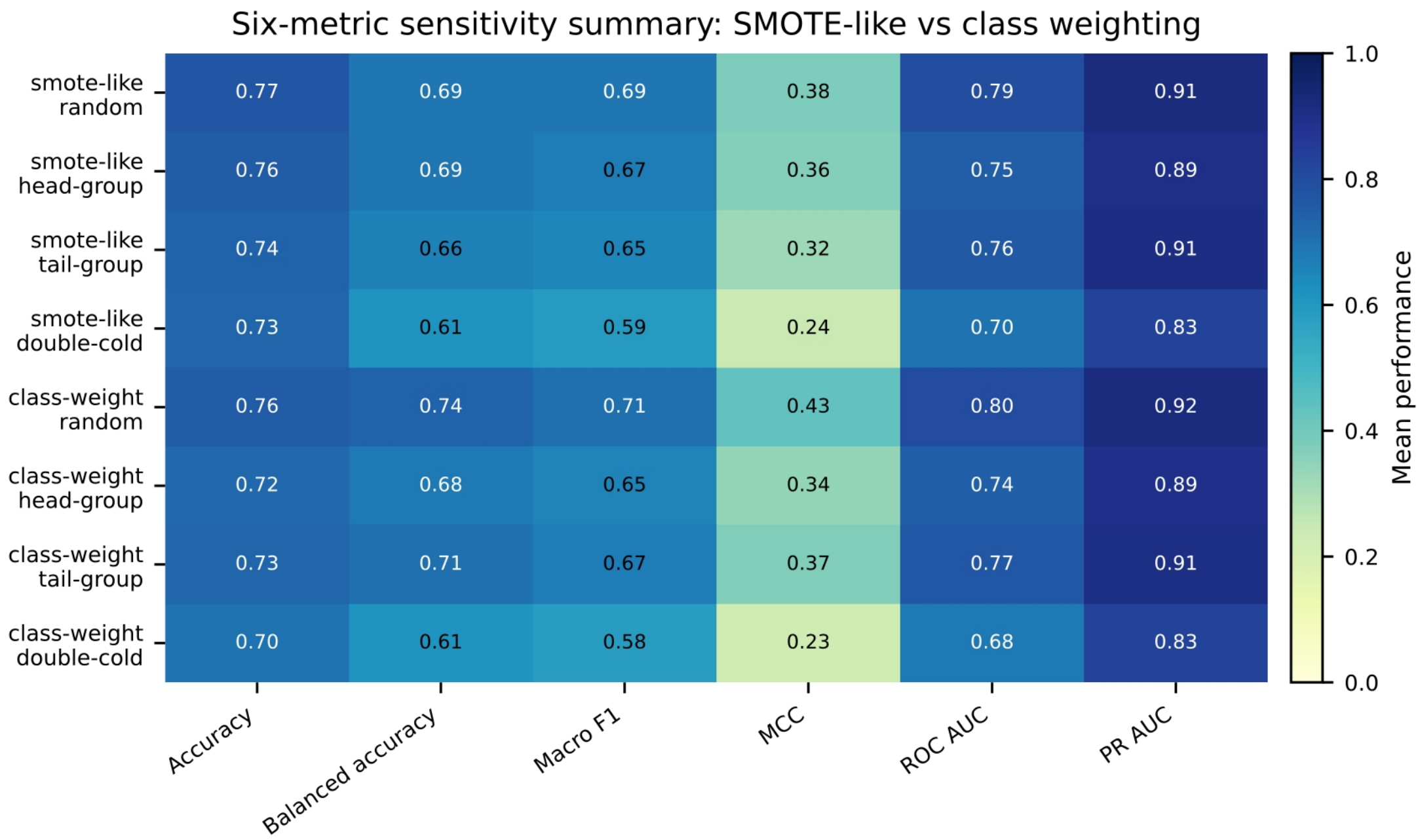

Supplementary Fig. 5. Sensitivity analysis of class-imbalance handling for the combined head–tail XGBoost model. Mean performance of the combined head–tail XGBoost model under random, head-grouped, tail-grouped, and strict double-cold validation using either training-fold-only SMOTE-like oversampling or class weighting. Columns show accuracy, balanced accuracy, macro F1, Matthews correlation coefficient (MCC), ROC AUC, and PR AUC. Rows indicate the combination of class-imbalance handling strategy and validation strategy. Cell colors and overlaid values indicate the mean metric values across 20 validation splits.

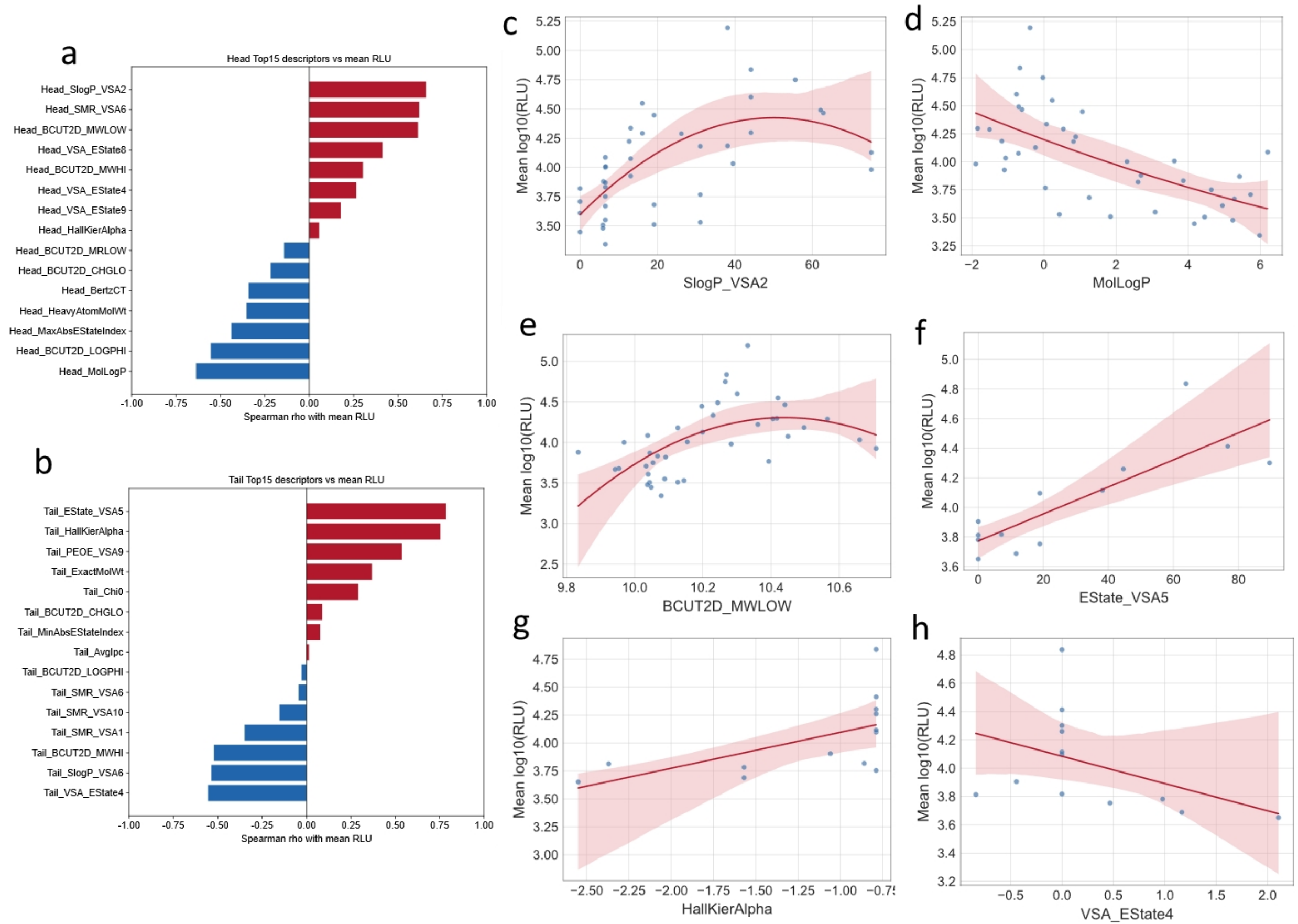

Supplementary Fig. 6. Module-level associations between molecular descriptors and in vitro mRNA transfection activity. (a, b) Spearman correlations between the top-ranked head-module descriptors (a; n = 40 heads) or tail-module descriptors (b; n = 13 tails) and module-averaged log10-transformed relative luminescence units (RLU). For each head module, the response was averaged across its 13 tail combinations; for each tail module, the response was averaged across its 40 head combinations. Red and blue bars denote positive and negative correlations, respectively. (c–e) Representative relationships for the head descriptors SlogP_VSA2 (c), MolLogP (d), and BCUT2D_MWLOW (e). (f–h) Representative relationships for the tail descriptors EState_VSA5 (f), HallKierAlpha (g), and VSA_EState4 (h). Each point represents one independent head or tail module. Red curves indicate fitted trends, and shaded bands denote 95% confidence intervals. These associations describe the HeLa-cell transfection phenotype and should not be interpreted as determinants of *in vivo* hepatocyte-enriched functional expression.

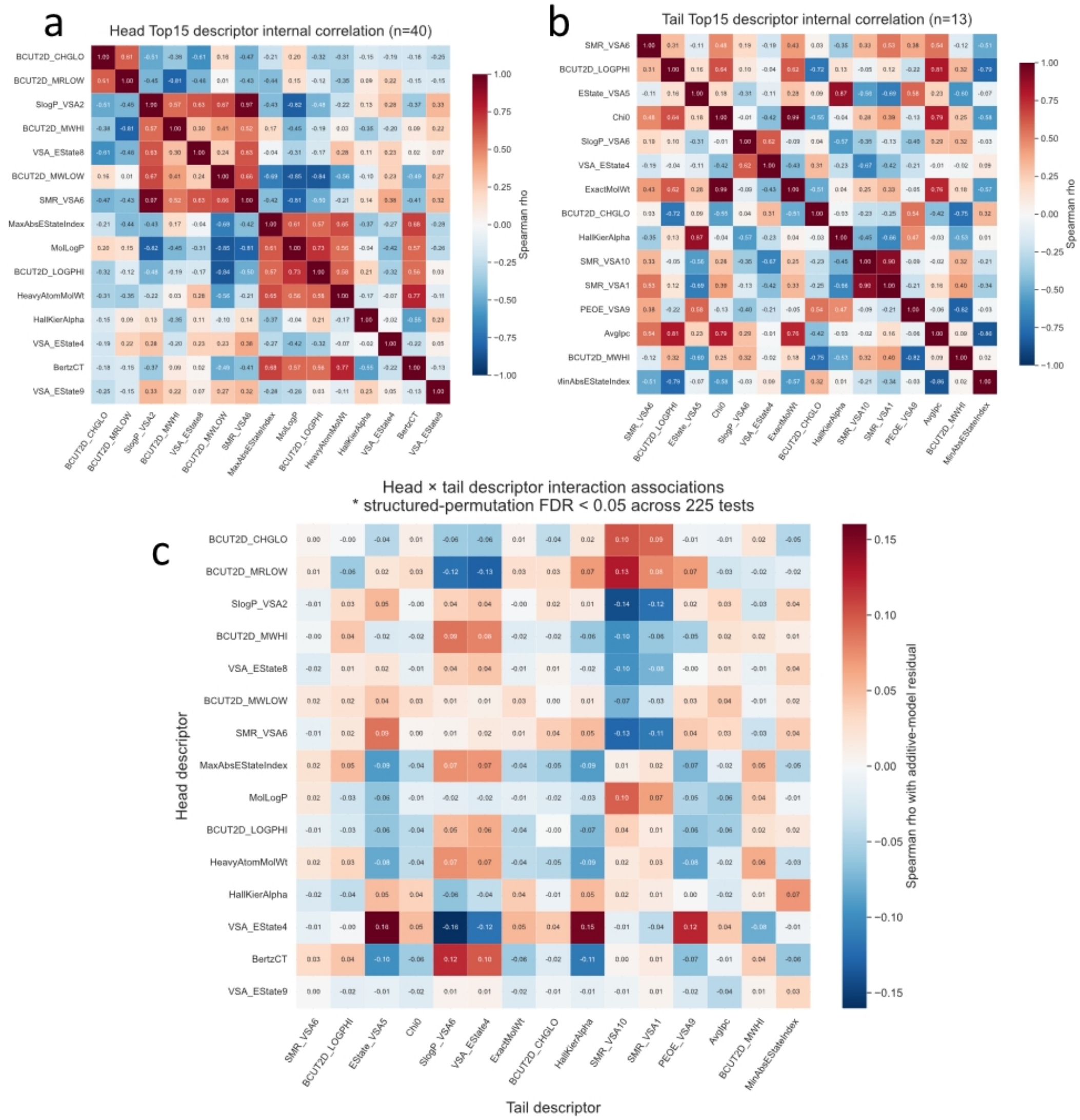

Supplementary Fig. 7. Descriptor collinearity and exploratory head–tail interaction analysis. (a, b) Spearman correlation matrices among the top-ranked head descriptors across 40 head modules (a) and tail descriptors across 13 tail modules (b). Cell values and colors indicate Spearman's ρ. (c) Spearman correlations between pairwise head–tail descriptor interaction terms and residuals from an additive head-plus-tail model of log10-transformed RLU. The matrix contains 225 descriptor-pair tests. Asterisks, if present, indicate interactions meeting the structured-permutation false-discovery-rate threshold of FDR < 0.05. This analysis evaluates whether individual descriptor pairs explain activity beyond additive module effects and should be interpreted as exploratory rather than causal.

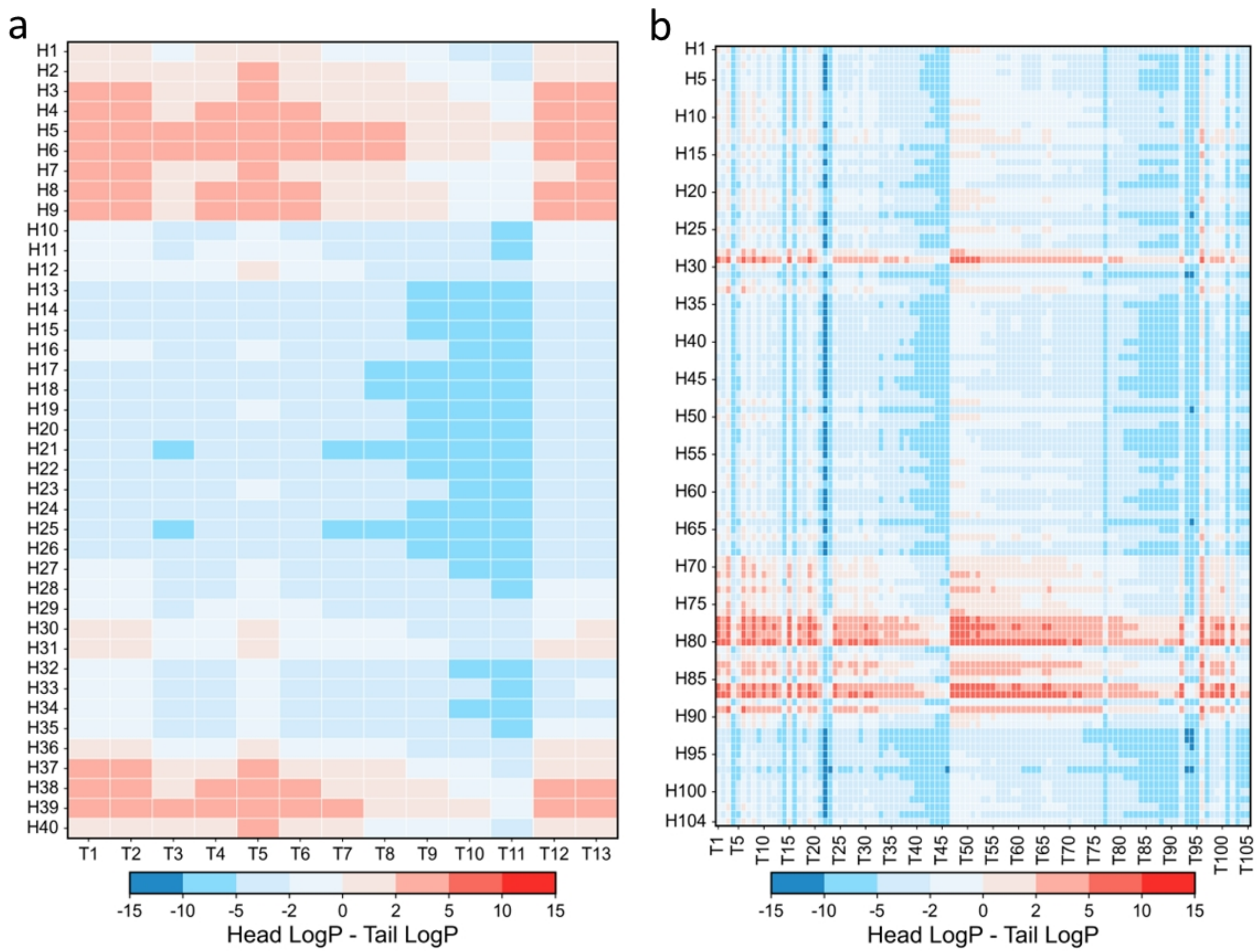

Supplementary Fig. 8. Distribution of head–tail LogP differences in the experimental and virtual chemical spaces. Heat maps of calculated Head LogP − Tail LogP values for the 40 × 13 experimental AMD combinations (a) and the 104 × 105 virtual PAMD combinations (b). Rows correspond to head modules and columns correspond to tail modules. Red indicates combinations in which the calculated Head LogP exceeds the Tail LogP, blue indicates combinations with a more hydrophobic tail, and white indicates similar Head and Tail LogP values. These maps visualize the hydrophobicity-balance space sampled by the experimental library and the expanded virtual library.

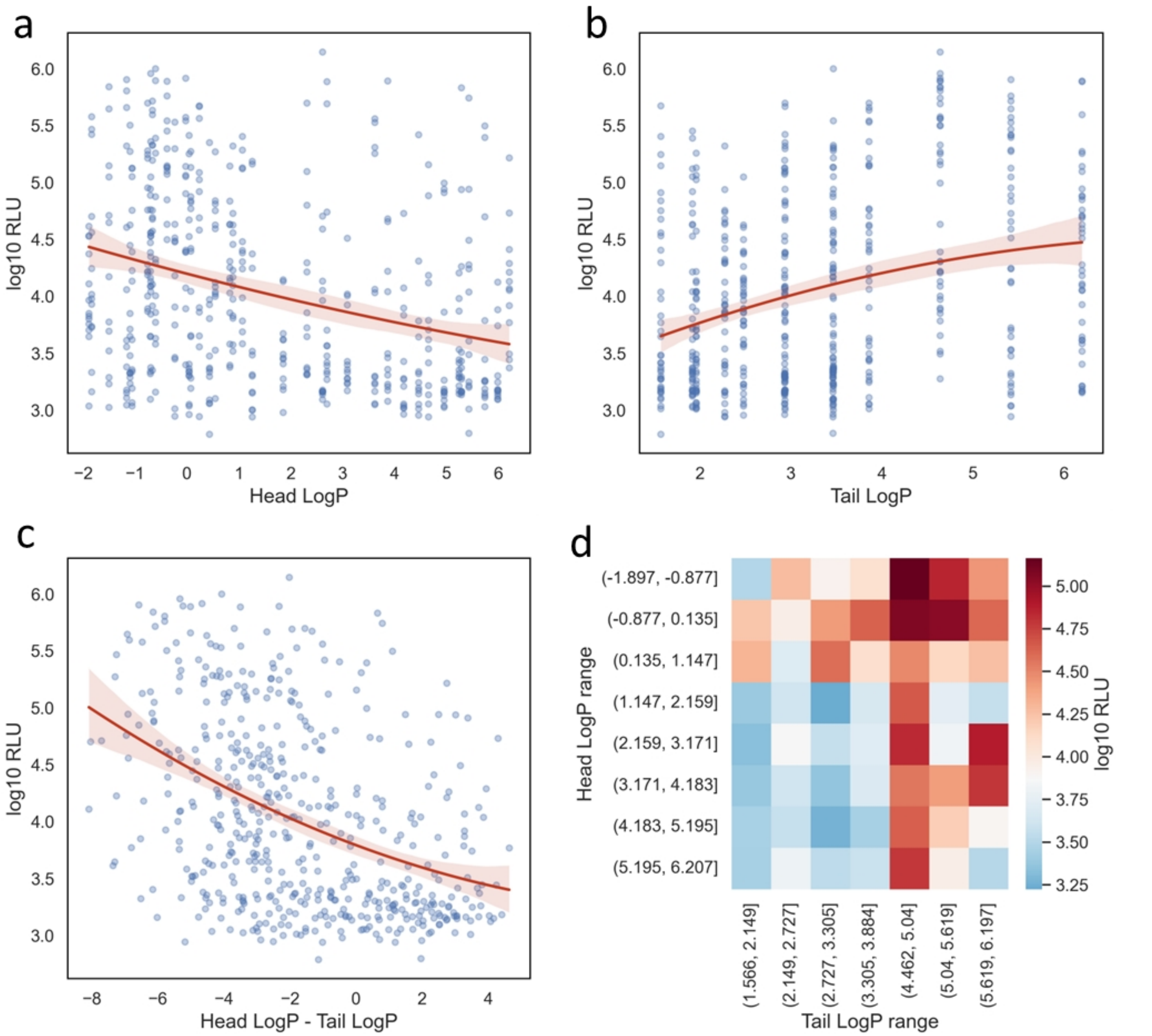

Supplementary Fig. 9. Relationships between head–tail hydrophobicity and in vitro mRNA transfection activity. Relationships between calculated module LogP values and log10-transformed RLU across the 520 experimental AMD head–tail combinations. (a–c) Associations of Head LogP (a), Tail LogP (b), and Head LogP − Tail LogP (c) with log10(RLU). Each blue point represents one head–tail combination; red curves indicate quadratic polynomial fits, and shaded bands denote 95% bootstrap confidence intervals. (d) Two-dimensional binned heat map of Head LogP and Tail LogP. Cell colors indicate the mean log10(RLU) for combinations within each bin; blank cells indicate bins without observations. These analyses identify associations between head–tail hydrophobicity balance and the in vitro transfection phenotype, rather than causal determinants of in vivo delivery.

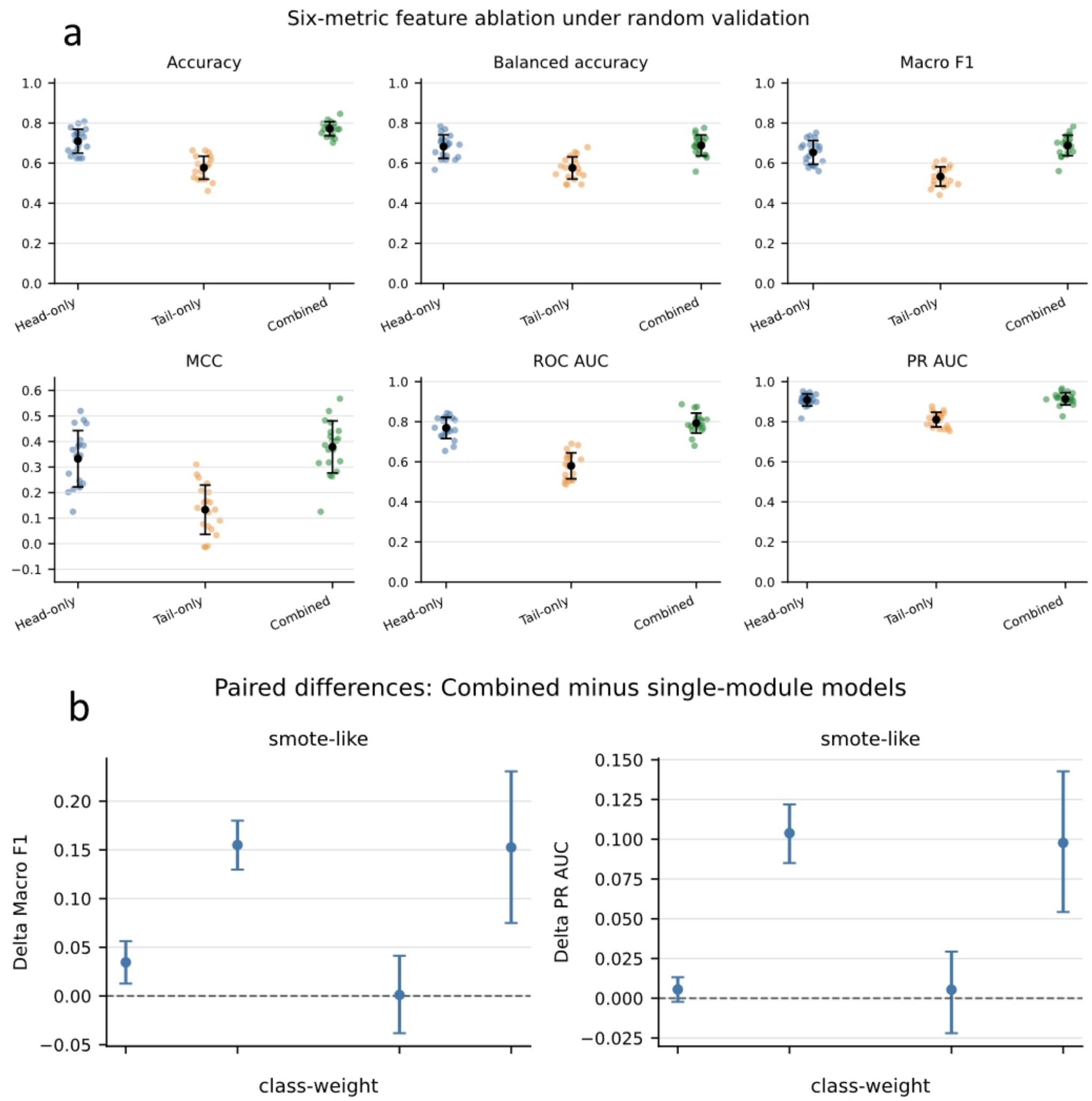

Supplementary Fig. 10. Ablation analysis of head-only, tail-only, and combined molecular representations. (a) Performance of Head-only, Tail-only and combined Head–Tail XGBoost models evaluated using matched repeated splits under random and strict double-cold validation. Accuracy, balanced accuracy, macro-F1, Matthews correlation coefficient (MCC), ROC-AUC, and PR-AUC are shown. zero-variance feature filtering, feature scaling, and SMOTE-like class balancing were performed within each training fold only. Colored points represent individual test splits; black points and error bars denote mean ± s.d. (n = 20 splits). (b) Paired split-level differences in macro-F1 and PR-AUC between the combined model and the corresponding single-module models using the same SMOTE-like workflow. The four comparisons correspond to Combined versus Head-only and Combined versus Tail-only under random validation and strict double-cold validation. Points denote mean paired differences, error bars denote bootstrap 95% confidence intervals, and dashed lines indicate no difference. Positive values favor the combined representation. Together, the results indicate that head-derived features contain most of the predictive information, whereas tail-derived features provide more limited complementary information.

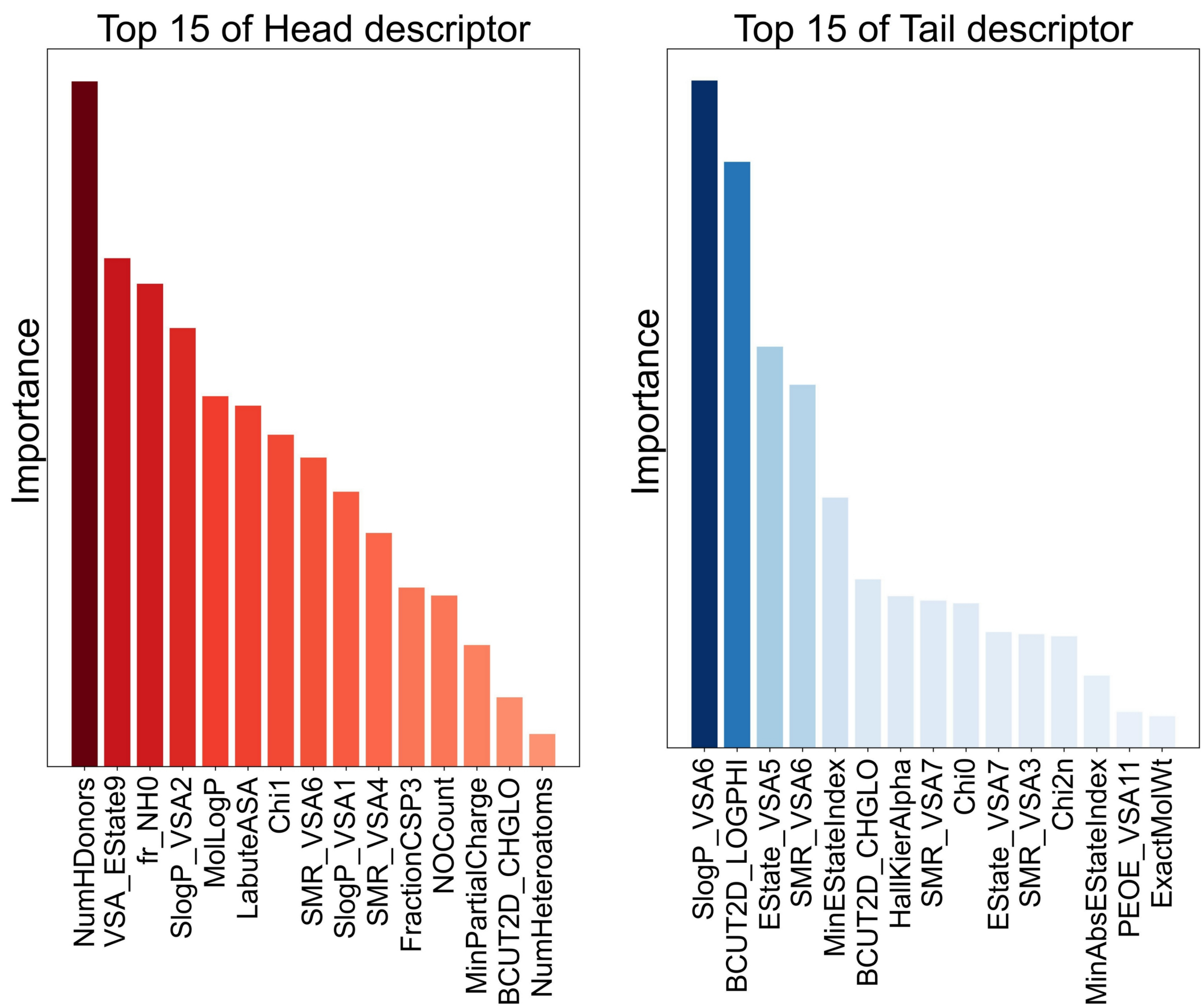

Supplementary Fig. 11. **Module-resolved descriptor importance in the XGBoost model.**

Gain-based rankings of the top 15 descriptors calculated for the Head (left) and Tail (right) modules. The leading descriptors capture charge distribution, lipophilicity and molecular connectivity associated with the HeLa-cell transfection phenotype.

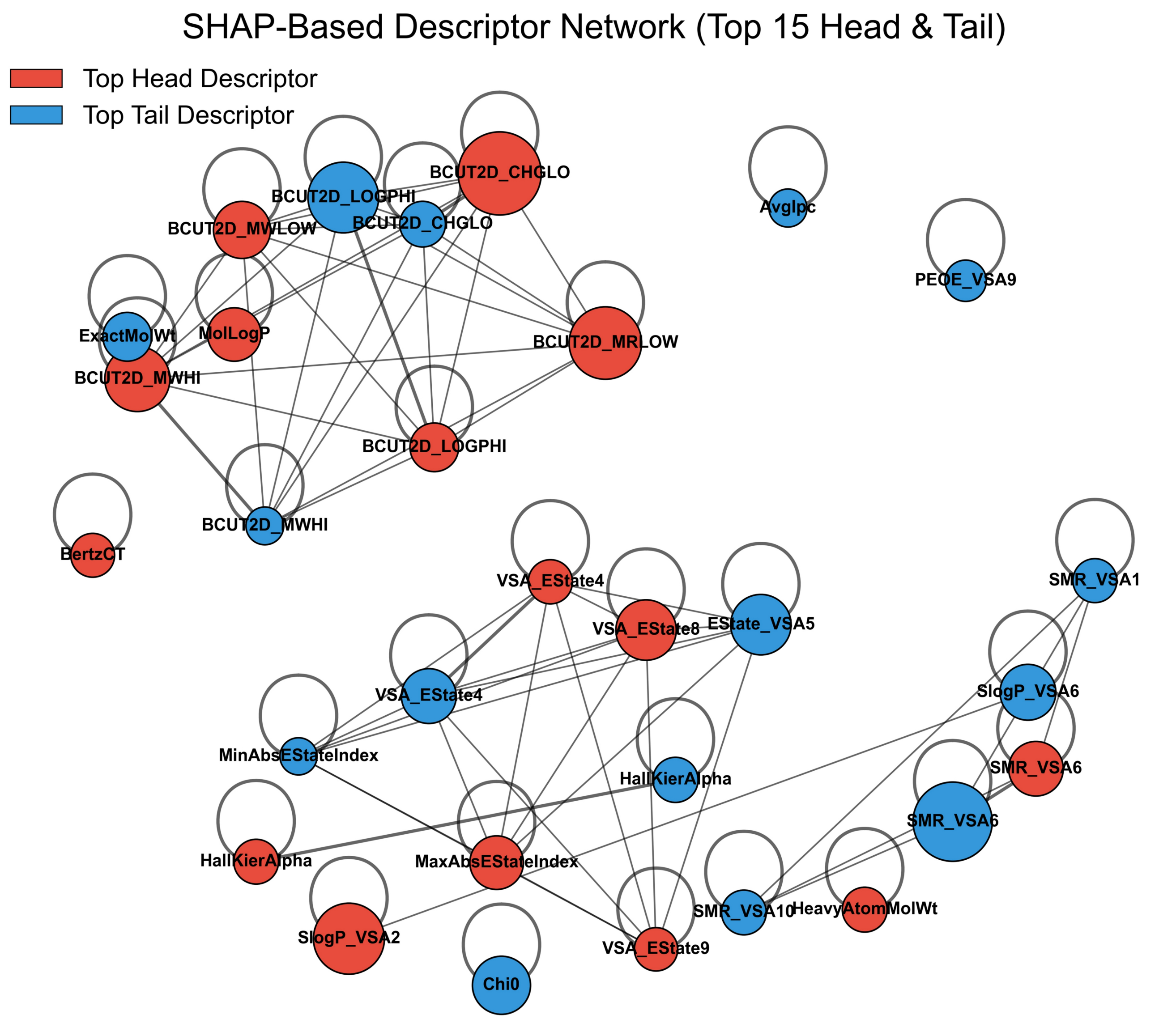

Supplementary Fig. 12. Correlation network of key molecular descriptors associated with the HeLa-cell in vitro transfection phenotype. Network visualization of pairwise correlations among the top-ranked descriptors for amine headgroups (red nodes) and degradable tail modules (blue nodes). Edges represent pairwise correlations, and the network structure highlights clusters of related physicochemical features and potential redundancy among model inputs. Nodes are scaled according to their relative gain-based feature importance in the XGBoost model.

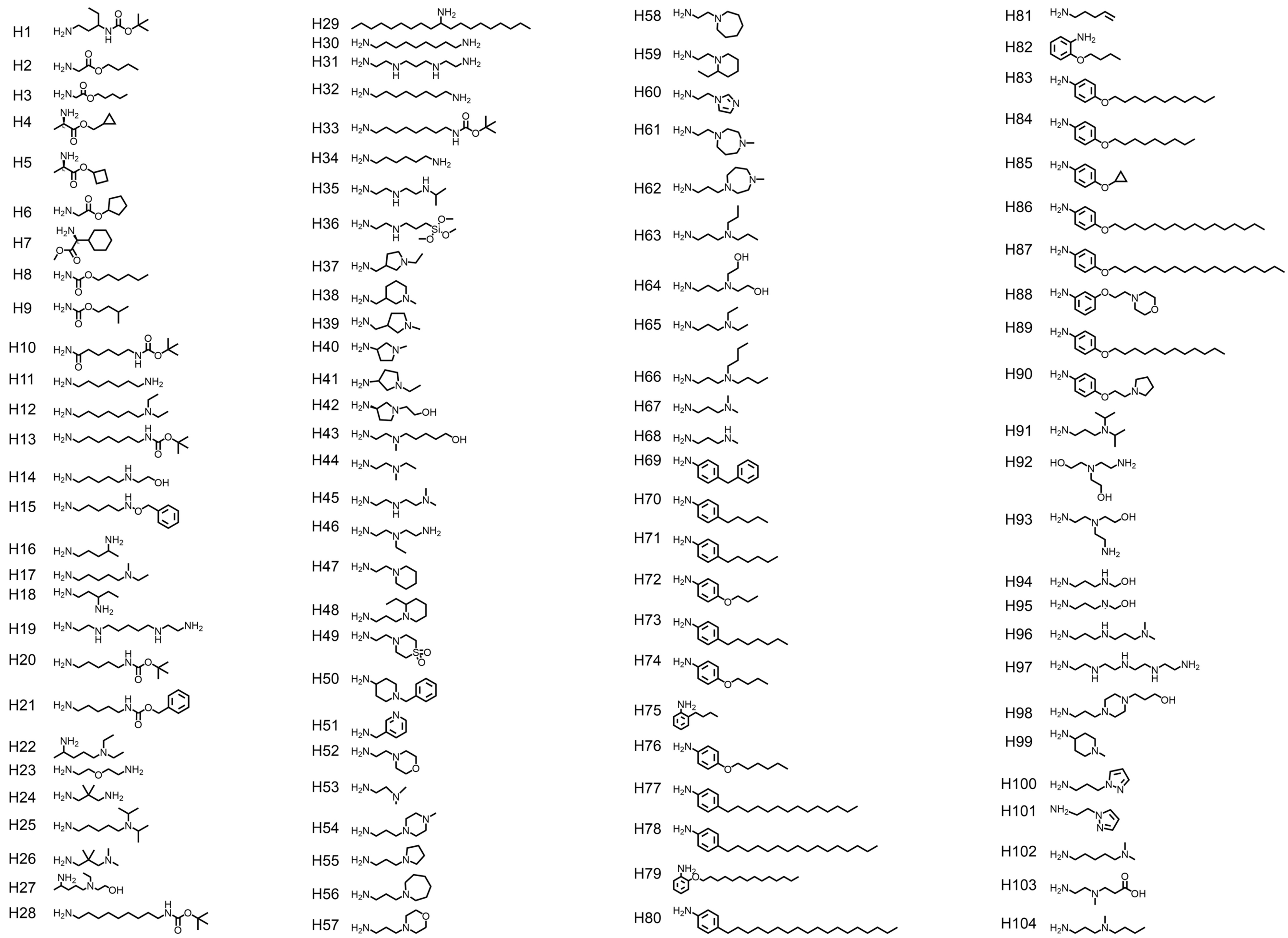

Supplementary Fig. 13. Structural diversity of the extended amine headgroup library for virtual screening. Detailed chemical structures of the 104 amine heads (H1–H104) employed in the machine learning (ML)-driven virtual expansion phase. These building blocks represent a diverse chemical space, including linear, branched, and cyclic motifs with varying degrees of nitrogen substitution and polar functionalities. The trained XGBoost model was utilized to navigate the resulting 10,920-member combinatorial space, enabling the prioritization of candidates with high predicted in vitro transfection activity beyond the initial experimental set.

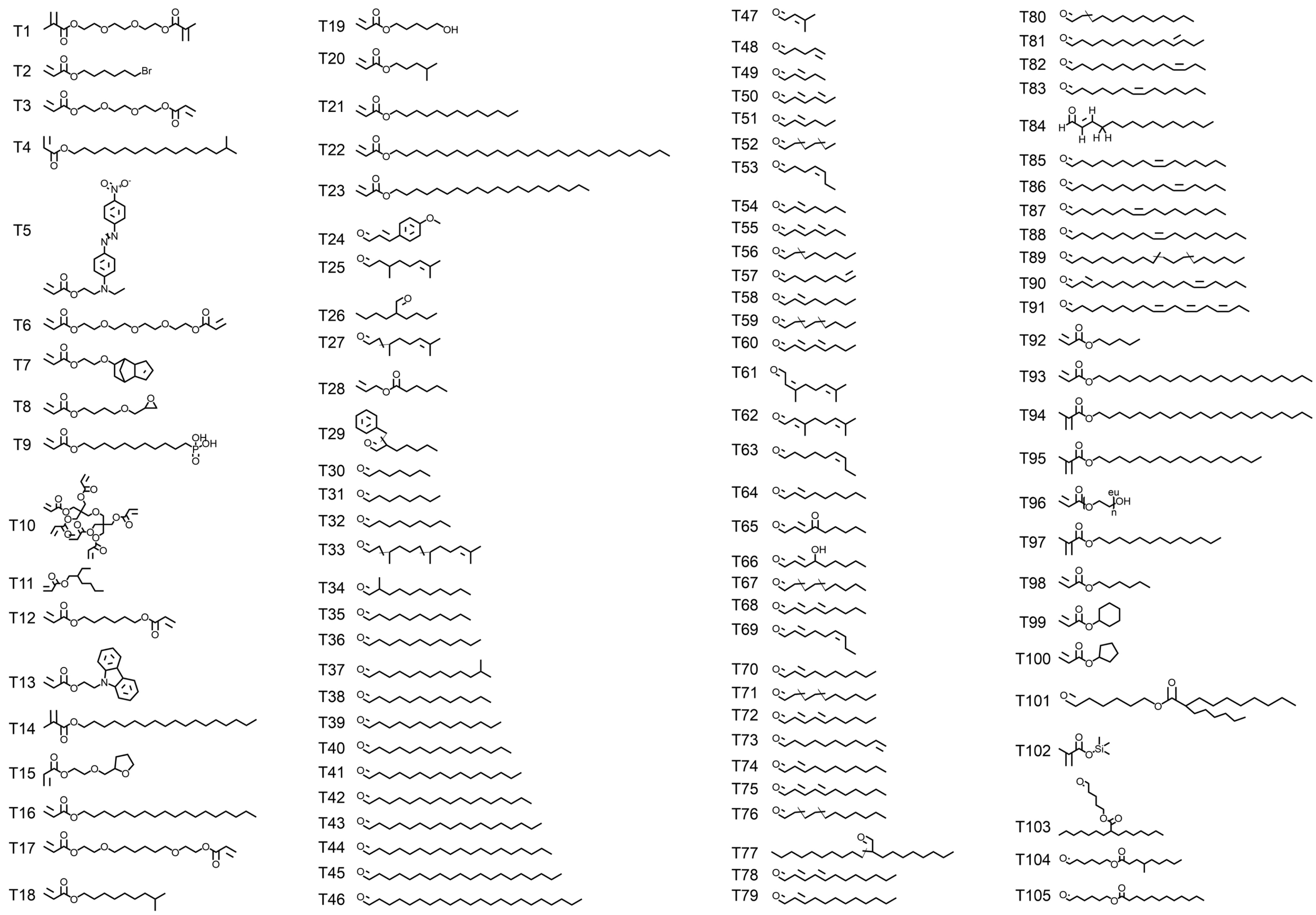

Supplementary Fig. 14. **Structural diversity of the extended degradable tail library for virtual screening.** Chemical structures of the 105 hydrophobic tail modules (T1–T105) used to construct the expanded PAMD virtual library. This structural pool encompasses a broad range of carbon chain lengths, degrees of unsaturation, and bioreducible ester linkages. By integrating these tail architectures with the amine headgroups (Fig. S13), the ML platform facilitated the systematic exploration of lipophilicity and geometric parameters, leading to the selection of candidate lipids with high predicted in vitro transfection activity for subsequent experimental evaluation.

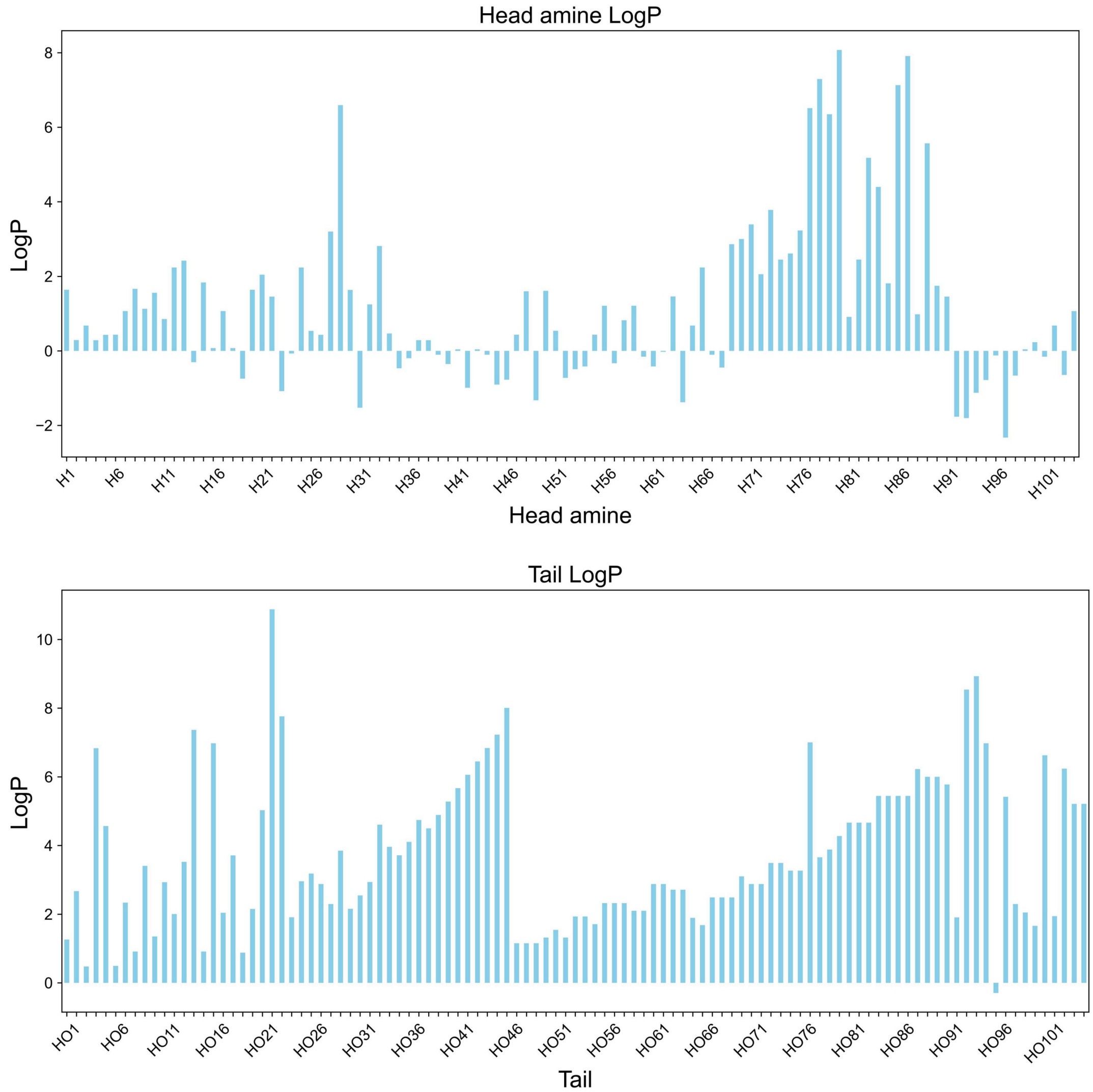

Supplementary Fig. 15. **Structural landscape and lipophilicity (LogP) distribution of the combinatorial building blocks for virtual library expansion**. Detailed chemical architectures and calculated LogP values are provided for the building blocks used to construct the 10,920-member PAMD virtual library. The upper panel displays the 104 amine headgroups (H1–H104), encompassing diverse motifs such as linear alkyl amines, branched architectures, and nitrogen-substituted heterocycles, illustrating a broad spectrum of hydrophilicity. The lower panel presents the 105 hydrophobic tail modules (T1–T105), featuring systematic variations in chain length, degrees of unsaturation, and bioreducible ester linkages, which define the lipophilic boundaries of the library. By combinatorially pairing these modules, the trained XGBoost model was able to navigate a vast, previously uncharacterized chemical space defined by these LogP landscapes. This in silico strategy facilitated the selection of candidate ionizable lipids with descriptor profiles associated with higher predicted in vitro transfection activity, extending far beyond the lipophilic diversity of the initial experimental training set.

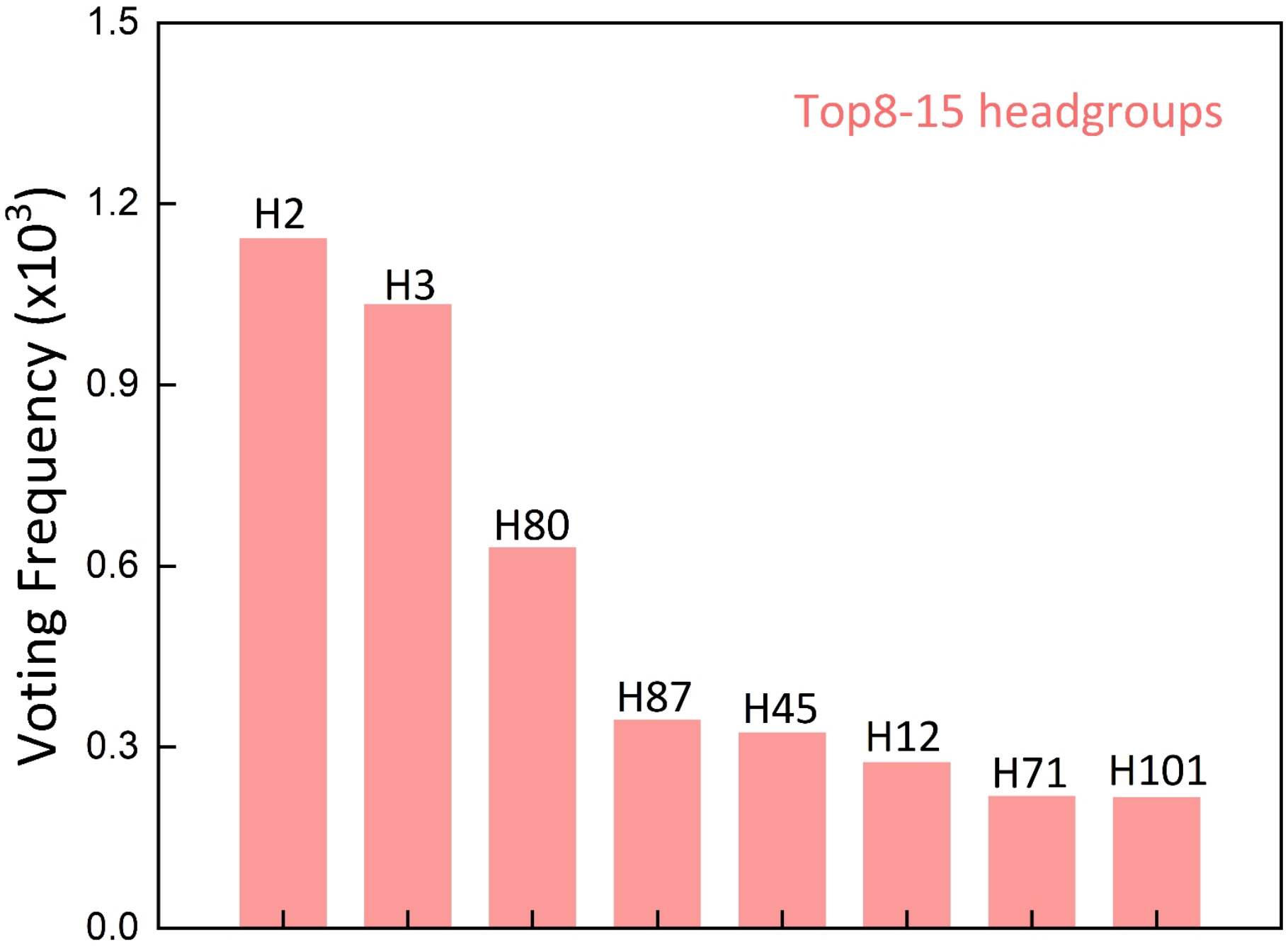

Supplementary Fig. 16. Frequency distribution of privileged amine heads (ranking from 8 to 15) identified through ensemble voting from the top-ranked candidates.

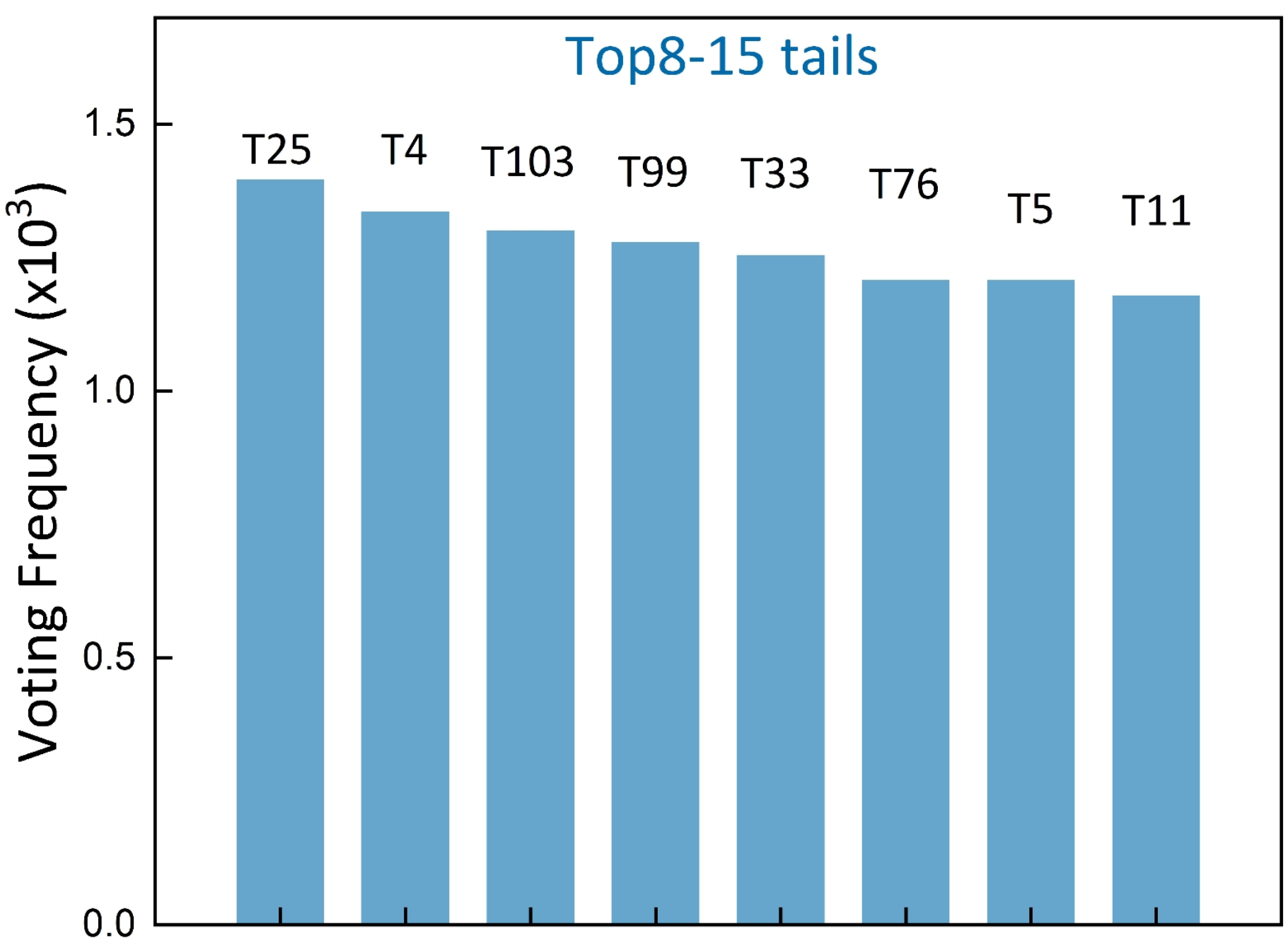

Supplementary Fig. 17. Frequency distribution of privileged degradable tails (ranking from 8 to 15) identified through ensemble voting from the top-ranked candidates.

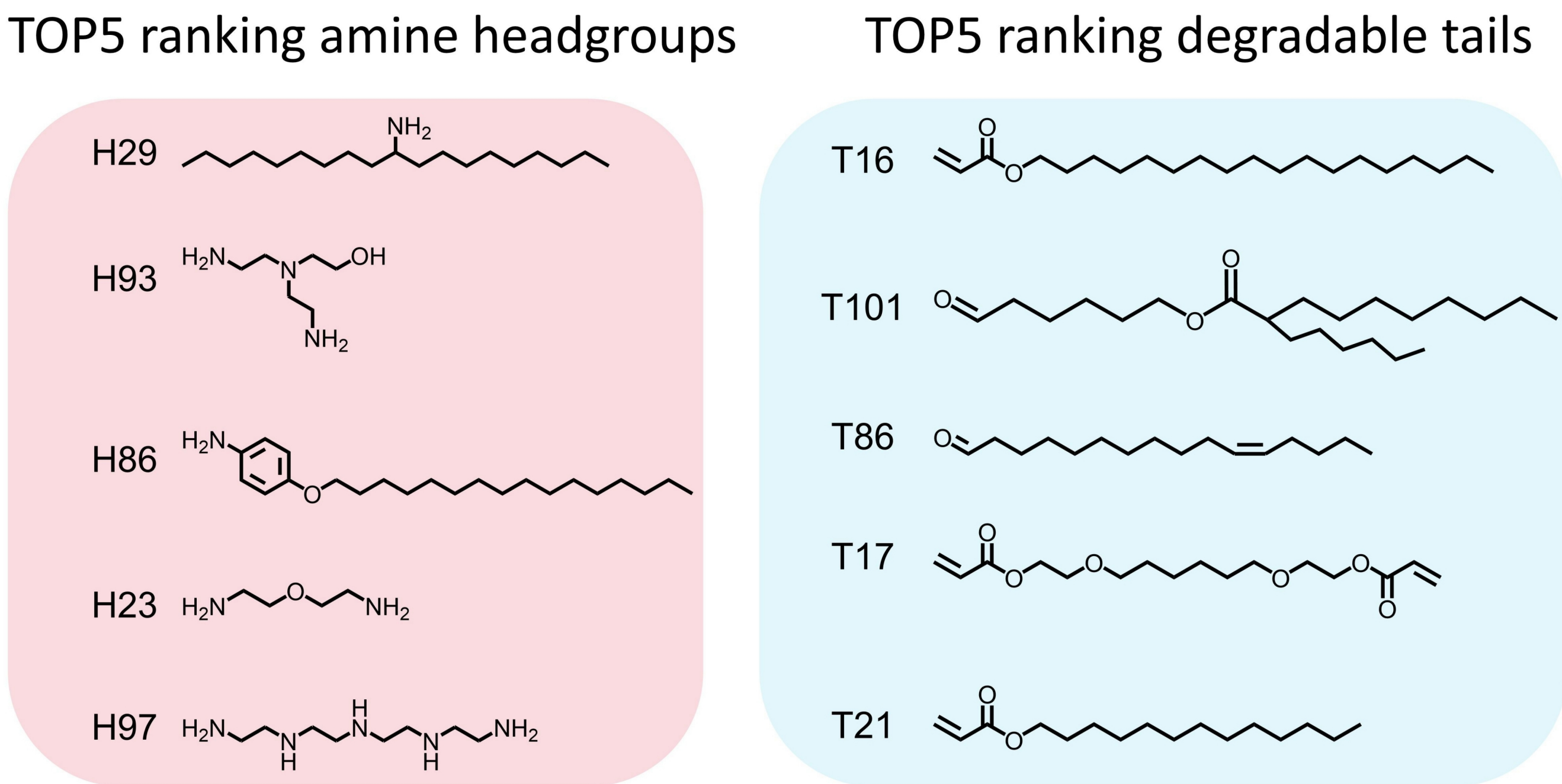

Supplementary Fig. 18. Chemical structures of the top-ranking head and tail modules selected for library construction.

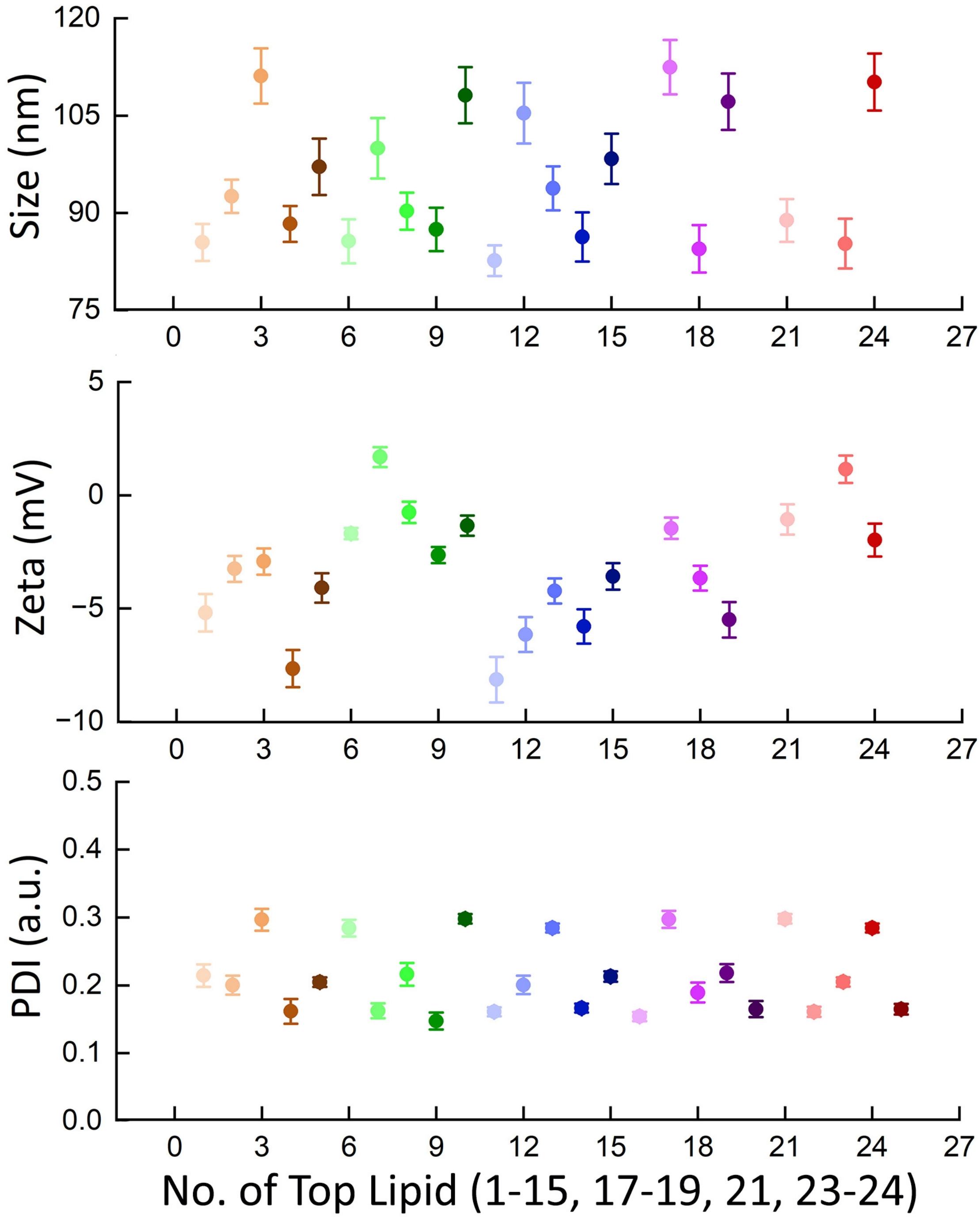

Supplementary Fig. 19. Physicochemical robustness and colloidal characterization of the ML-prioritized validation library. Experimental validation of the physical properties for a focused library (n = 25), synthesized through the combinatorial assembly of the top 5 amine headgroups and top 5 biodegradable tails identified via ML-driven virtual screening. (**Middle**) Zeta potential (mV) measurements illustrating the surface charge distribution of the resulting LNPs, with the majority of formulations maintaining a near-neutral profile conducive to favorable *in vivo* circulation. (**Upper**)

Dynamic light scattering (DLS) analysis of hydrodynamic diameters (nm), demonstrating consistent and predictable nanoparticle formation. (**Bottom**) Polydispersity index (PDI) as an indicator of particle homogeneity, with values generally below 0.25, confirming a monodisperse population. These data characterize the colloidal properties of the machine-learning-prioritized validation library and confirm that the selected formulations formed nanoparticles suitable for subsequent mRNA-delivery evaluation. Data are expressed as mean ± SD from three biologically independent experiments.

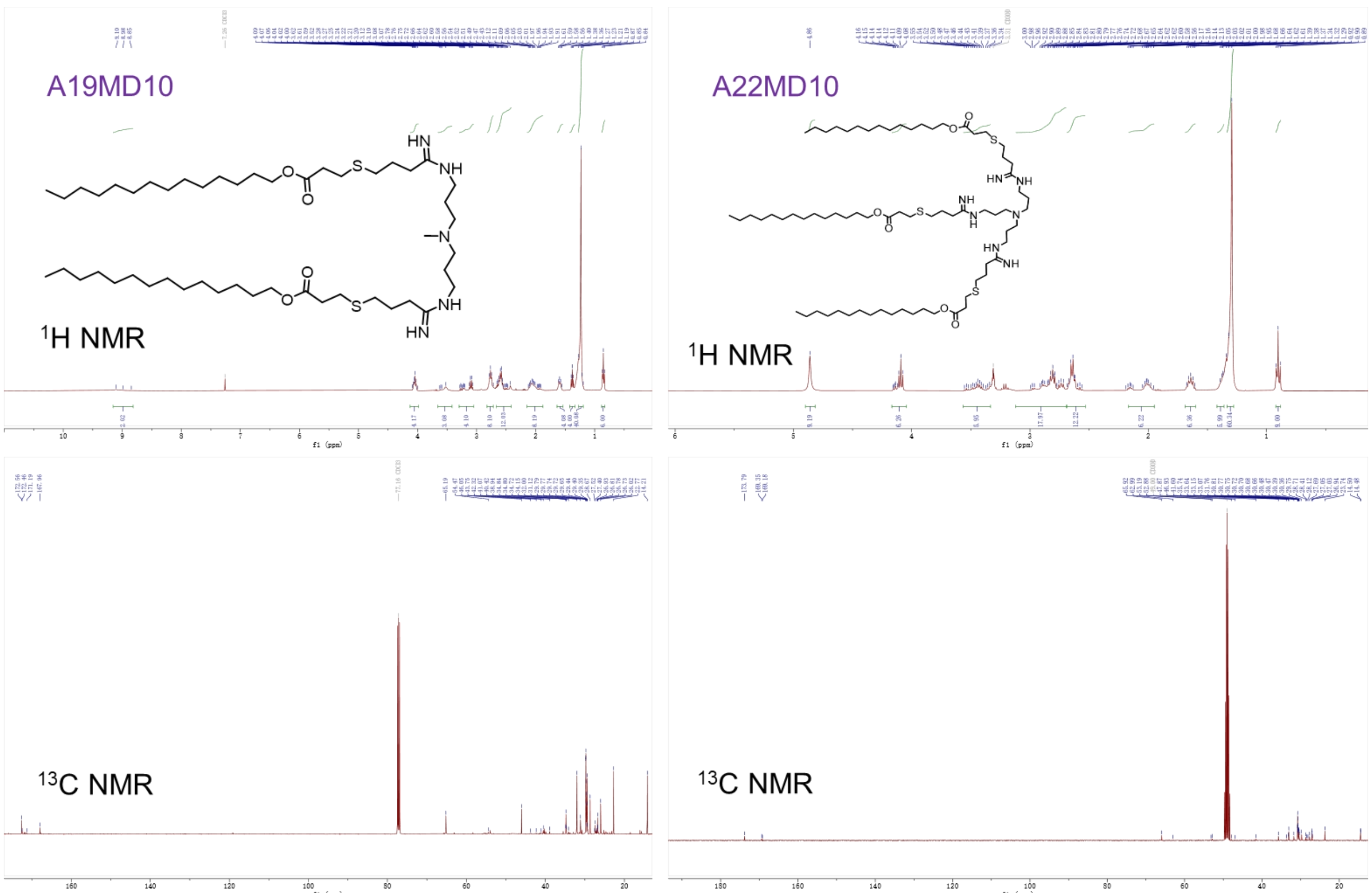

Supplementary Fig. 20. Representative ^{1}H NMR, ^{13}C NMR, and HRMS characterization of the A19MD10 (left) and A22MD10 (right) lipid synthesized. A19MD10: ^{1}H NMR (400 MHz, $CDCl_3$) δ 8.98 (s, 2H), 4.05 (q, *J* = 5.7 Hz, 4H), 3.66 – 3.41 (m, 3H), 3.30 – 3.05 (m, 4H), 2.76 (t, *J* = 7.2 Hz, 8H), 2.67 – 2.41 (m, 12H), 2.15 – 1.88 (m, 8H), 1.59 (q, *J* = 7.1 Hz, 4H), 1.38 (t, *J* = 7.3 Hz, 4H), 1.23 (m, 40H), 0.88 – 0.83 (m, 6H). ^{13}C NMR (101 MHz, $CDCl_3$) δ 172.56, 172.46, 171.19, 167.96, 65.19, 54.47, 46.05, 43.75, 42.32, 41.07, 40.42, 38.94, 34.84, 34.80, 34.72, 34.15, 32.00, 31.12, 29.79, 29.77, 29.74, 29.72, 29.65, 29.44, 29.40, 29.35, 28.67, 27.52, 27.40, 26.81, 26.78, 26.73, 26.02, 22.77, 14.21. Mass calculated m/z 883.6982, found [M + H] $^+$ (HRMS Direct Infusion) m/z 884.7059. A22MD10: ^{1}H NMR (400 MHz, MeOD) δ 4.86 (s, 9H), 4.17 – 4.04 (m, 6H), 3.56 – 3.33 (m, 6H), 3.12 – 2.69 (m, 18H), 2.69 – 2.53 (m, 12H), 2.17 – 1.95 (m, 6H), 1.64 (p, *J* = 6.7 Hz, 6H), 1.42 – 1.36 (m, 6H), 1.29 (m, 60H), 0.92 – 0.88 (m, 9H). ^{13}C NMR (101 MHz, MeOD) δ 173.79, 169.35, 169.18, 65.92, 62.99, 53.19, 52.88, 47.87, 46.93, 41.60, 35.74, 33.64, 33.15, 33.07, 31.76, 30.81, 30.77,

30.75, 30.72, 30.70, 30.68, 30.66, 30.48, 30.47, 30.39, 30.36, 29.75, 28.71, 28.41, 28.12, 27.69, 27.05, 27.03, 26.94, 23.74, 14.50, 14.48. Mass calculated m/z 1296.01055, found $[M + Na]^{+}$ (HRMS Direct Infusion) m/z 1318.9995.

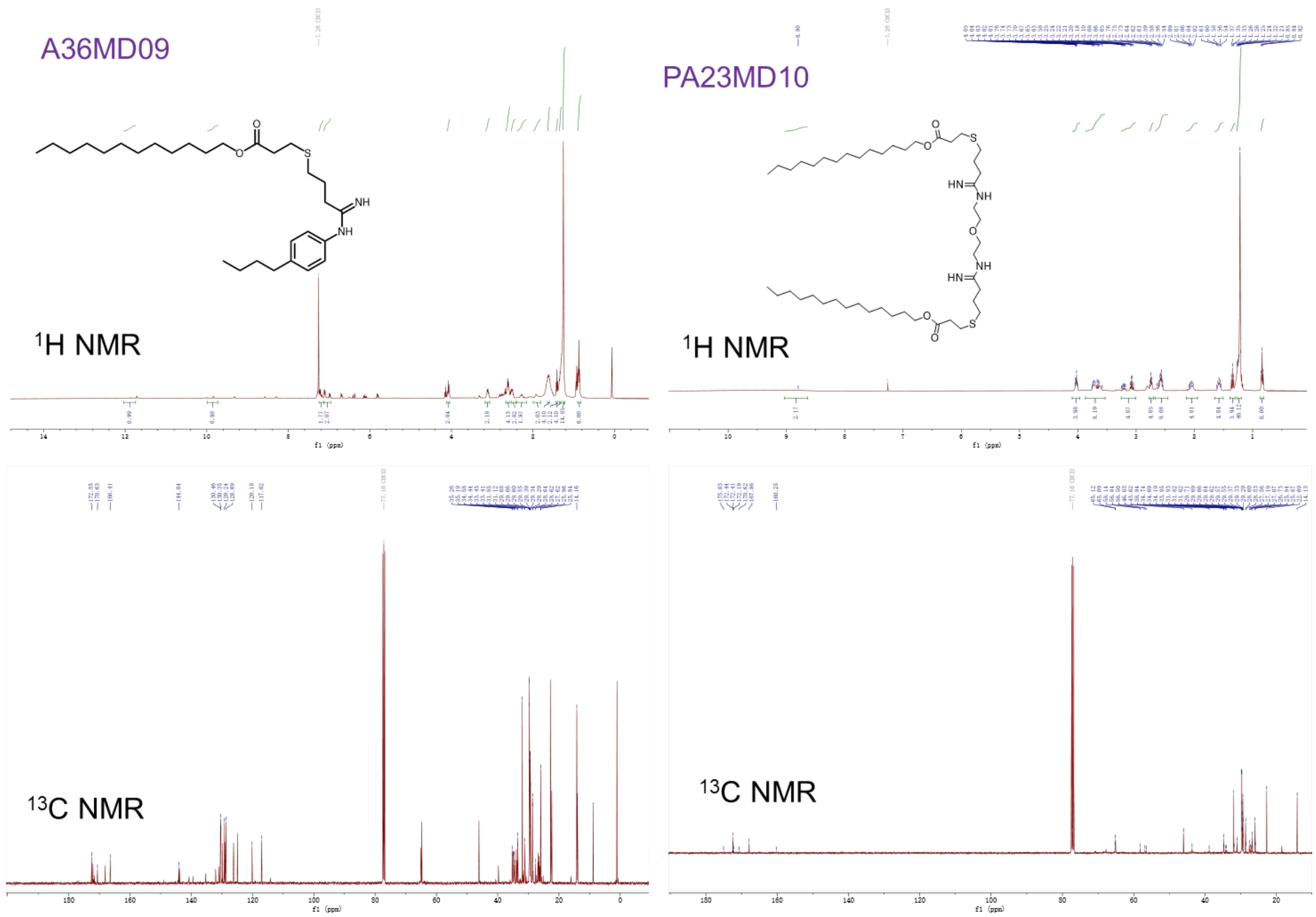

Supplementary Fig. 21. Representative ^{1}H NMR, ^{13}C NMR, and HRMS characterization of the A36MD09 (left) and PA23MD10 (right) lipid synthesized. A36MD09: ^{1}H NMR (400 MHz, $CDCl_3$) δ 11.90 (d, *J* = 30.9 Hz, 1H), 9.84 (s, 1H), 7.20 (dd, *J* = 14.3, 8.0 Hz, 2H), 7.05 (dd, *J* = 50.3, 8.1 Hz, 2H), 4.07 (q, *J* = 6.8 Hz, 2H), 3.11 (qd, *J* = 7.4, 4.6 Hz, 2H), 2.67 – 2.57 (m, 4H), 2.53 – 2.42 (m, 2H), 2.27 (dt, *J* = 13.4, 6.9 Hz, 2H), 1.93 (dt, *J* = 12.6, 5.6 Hz, 2H), 1.64 – 1.60 (m, 4H), 1.41 (t, *J* = 7.3 Hz, 2H), 1.35 (d, *J* = 7.2 Hz, 4H), 1.26 (m, 14H), 0.88 (t, *J* = 6.8 Hz, 6H). ^{13}C NMR (101 MHz, $CDCl_3$) δ 172.55, 170.63, 166.41, 144.04, 130.46, 130.35, 129.24, 128.69, 120.18, 117.02, 35.26, 35.19, 34.58, 34.44, 33.43, 33.41, 31.95, 31.12, 29.68, 29.66, 29.60, 29.55, 29.39, 29.34, 29.29, 28.64, 28.62, 27.62, 25.96, 25.94, 14.16. Mass calculated m/z 490.359, found $[M + H]^{+}$ (HRMS Direct Infusion) m/z 491.367. PA23MD10: ^{1}H NMR (400 MHz, $CDCl_3$) δ 8.80 (s, 2H), 4.03 (q, *J* = 3.7 Hz, 4H), 3.87 – 3.53 (m, 8H), 3.26 – 3.01 (m, 4H), 2.75 (t, *J* = 7.2 Hz, 4H), 2.68 – 2.45 (m, 8H), 2.06 (dt, *J* = 14.1, 7.0 Hz, 4H), 1.57 (q, *J* = 6.9 Hz, 4H), 1.35 (t, *J* = 7.3 Hz, 4H), 1.22 (m, 40H), 0.87 – 0.81 (m, 6H). ^{13}C NMR (101 MHz, $CDCl_3$) δ 172.44, 172.41, 172.19, 170.62, 167.86, 160.25, 76.94, 65.12, 65.09, 58.14, 56.84, 56.50, 46.03, 43.62, 38.84, 34.74, 34.69, 34.19, 33.95, 31.93, 31.82, 31.02, 29.71,

29.69, 29.66, 29.64, 29.62, 29.57, 29.55, 29.37, 29.33, 29.29, 28.60, 28.53, 27.56, 27.19, 27.07, 26.73, 25.94, 25.87, 22.69, 14.13. Mass calculated m/z 842.63526, found $[M + H]^+$ (HRMS Direct Infusion) m/z 843.6432.

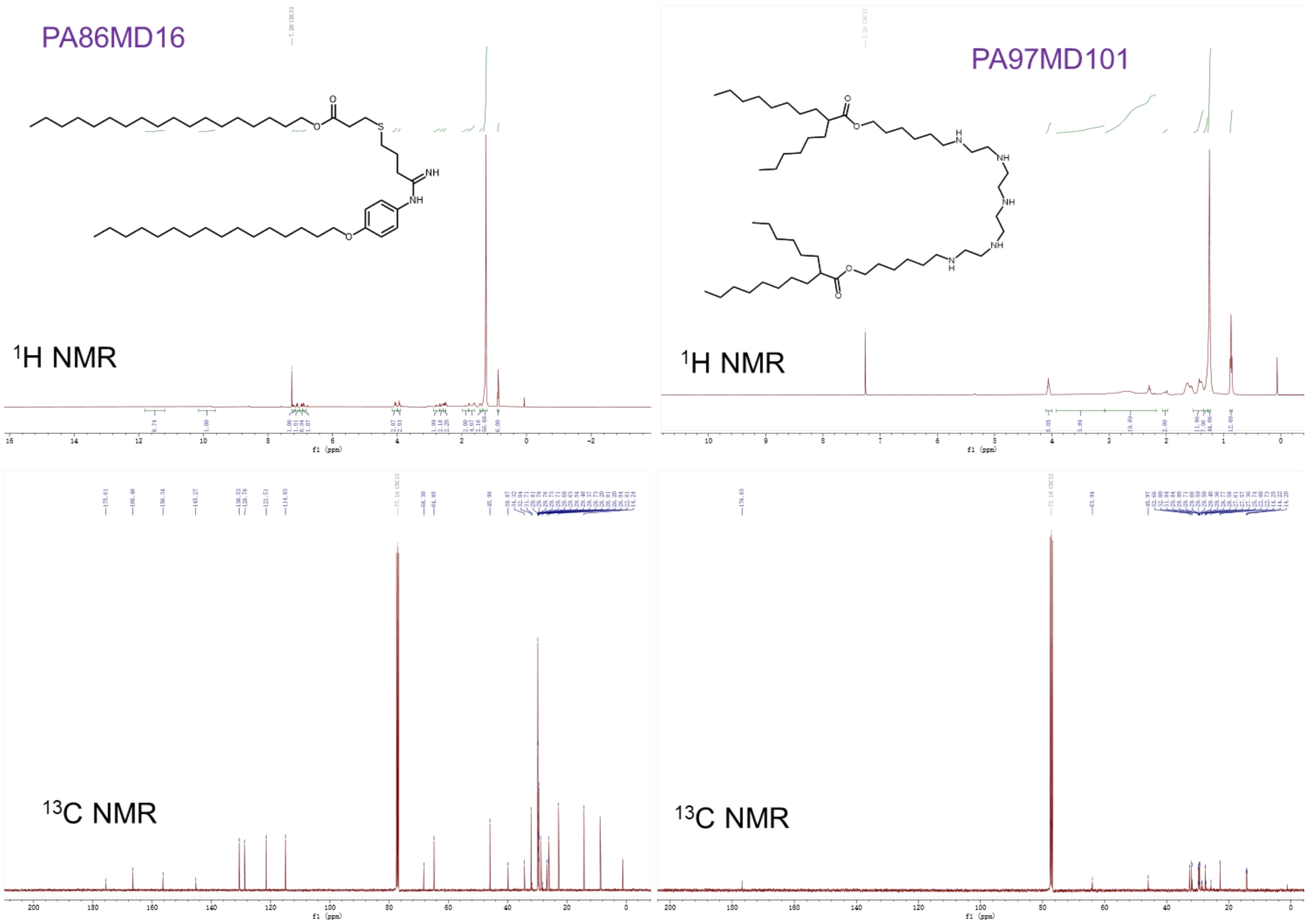

Supplementary Fig. 22. Representative ^{1}H NMR, ^{13}C NMR, and HRMS characterization of the PA86MD16 (left) and PA97MD101 (right) lipid synthesized. PA86MD16: ^{1}H NMR (400 MHz, $CDCl_3$) δ 11.45 (s, 1H), 10.15 – 9.63 (m, 1H), 7.20 (dd, *J* = 18.5, 8.8 Hz, 1H), 7.15 – 7.03 (m, 1H), 7.02 – 6.92 (m, 1H), 6.91 – 6.82 (m, 1H), 4.06 (dt, *J* = 9.2, 6.8 Hz, 2H), 3.95 (q, *J* = 5.3 Hz, 2H), 2.88 – 2.68 (m, 2H), 2.68 – 2.58 (m, 2H), 2.57 – 2.49 (m, 2H), 1.99 – 1.79 (m, 2H), 1.62 (s, 4H), 1.45 – 1.36 (m, 2H), 1.25 (m, 56H), 0.87 (t, *J* = 6.7 Hz, 6H). ^{13}C NMR (101 MHz, $CDCl_3$) δ 175.61, 166.48, 156.34, 145.27, 130.53, 128.78, 121.51, 114.93, 68.30, 64.85, 45.98, 39.87, 34.32, 32.04, 31.71, 29.81, 29.78, 29.76, 29.73, 29.71, 29.68, 29.63, 29.54, 29.48, 29.37, 28.73, 28.20, 26.81, 26.20, 26.04, 22.81, 14.24. Mass calculated m/z 758.63592, found $[M + H]^+$ (HRMS Direct Infusion) m/z 759.6439. PA97MD101: ^{1}H NMR (400 MHz, $CDCl_3$) δ 4.06 (m, 5H), 3.92 – 3.08 (m, 4H), 3.07 – 2.18 (m, 20H), 2.06 – 1.98 (m, 2H), 1.41 (ddt, *J* = 17.5, 7.6, 3.4 Hz, 12H), 1.35 – 1.28 (m, 8H), 1.25 (m, 44H), 0.89 – 0.85 (m, 12H). ^{13}C NMR (101 MHz, $CDCl_3$) δ 176.83, 63.94, 45.97, 32.66, 32.00, 31.84, 29.84, 29.80, 29.71, 29.66, 29.59, 29.50, 29.40, 29.36, 28.77, 28.58, 27.61, 27.57, 27.36, 25.74, 22.80, 22.73, 14.25, 14.22, 14.20. Mass calculated m/z 865.83231, found $[M + Na]^+$ (HRMS Direct Infusion) m/z

888.8228. The spectra support the assigned predominant structures but do not exclude trace regioisomeric or secondary-amine-derived species.

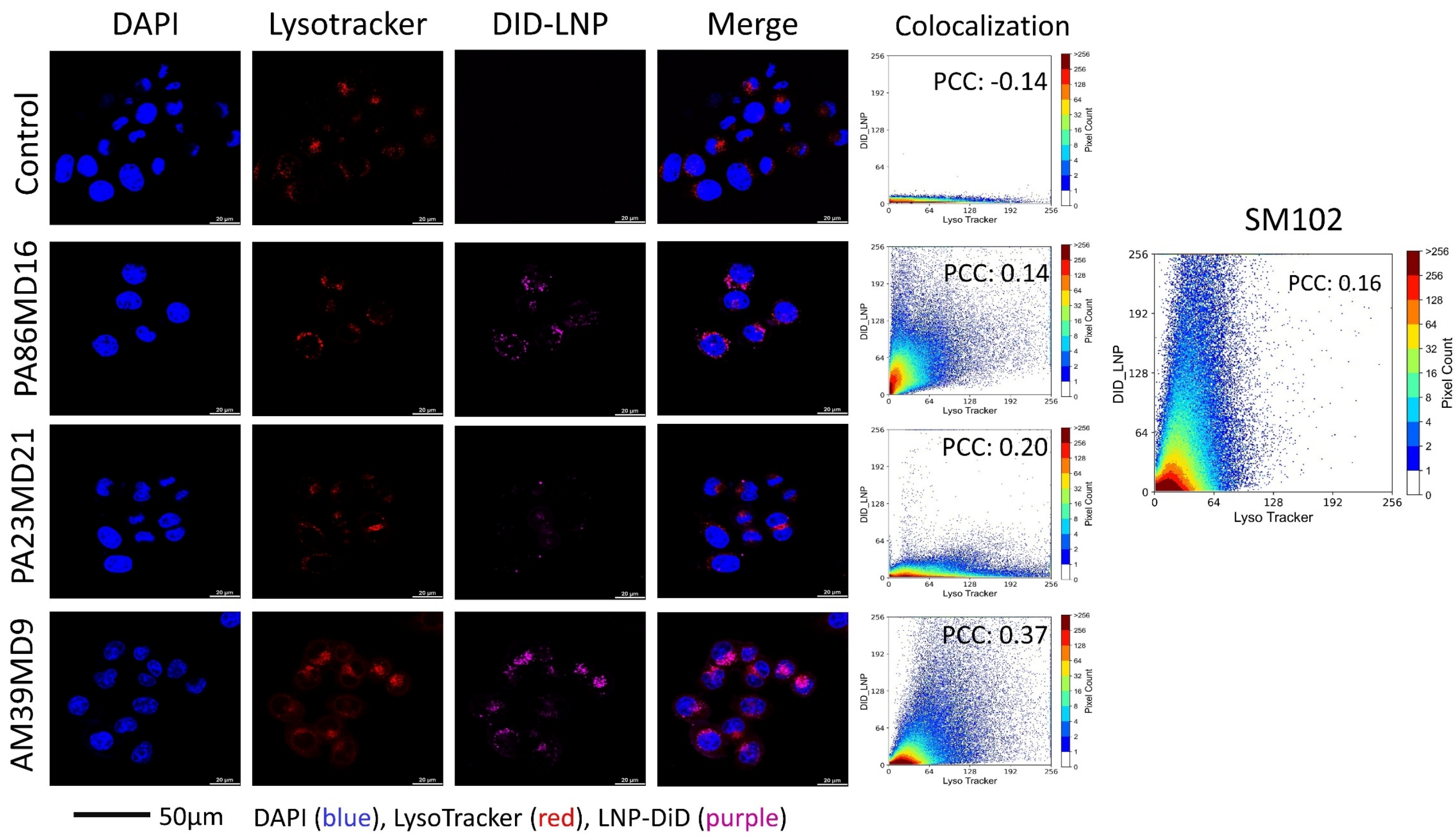

Supplementary Fig. 23. Visualization and quantification of endosomal escape via confocal colocalization analysis. Representative confocal laser scanning microscopy (CLSM) images of HeLa cells treated with DiD-labeled LNPs (purple) for 2 h. Acidic endo-lysosomal compartments were stained with LysoTracker Red (red), and nuclei were counterstained with Hoechst 33342 (blue). (**a–d**) Multichannel panels displaying individual fluorescence channels and merged overlays for the negative control (NC), ML-prioritized candidates (e.g., PA97MD101, PA86MD16), the experimental hit (A36MD09), and the benchmark SM102. (**e**) Corresponding colocalization scatter plots with calculated Pearson's Correlation Coefficients (PCC). A lower PCC indicates reduced spatial colocalization between LNP-associated fluorescence and acidic endo-lysosomal compartments, consistent with reduced endo-lysosomal retention. These colocalization patterns are consistent with the membrane-interaction phenotypes observed in the complementary hemolysis and lipid-mixing assays. Scale bars: 20 μm.

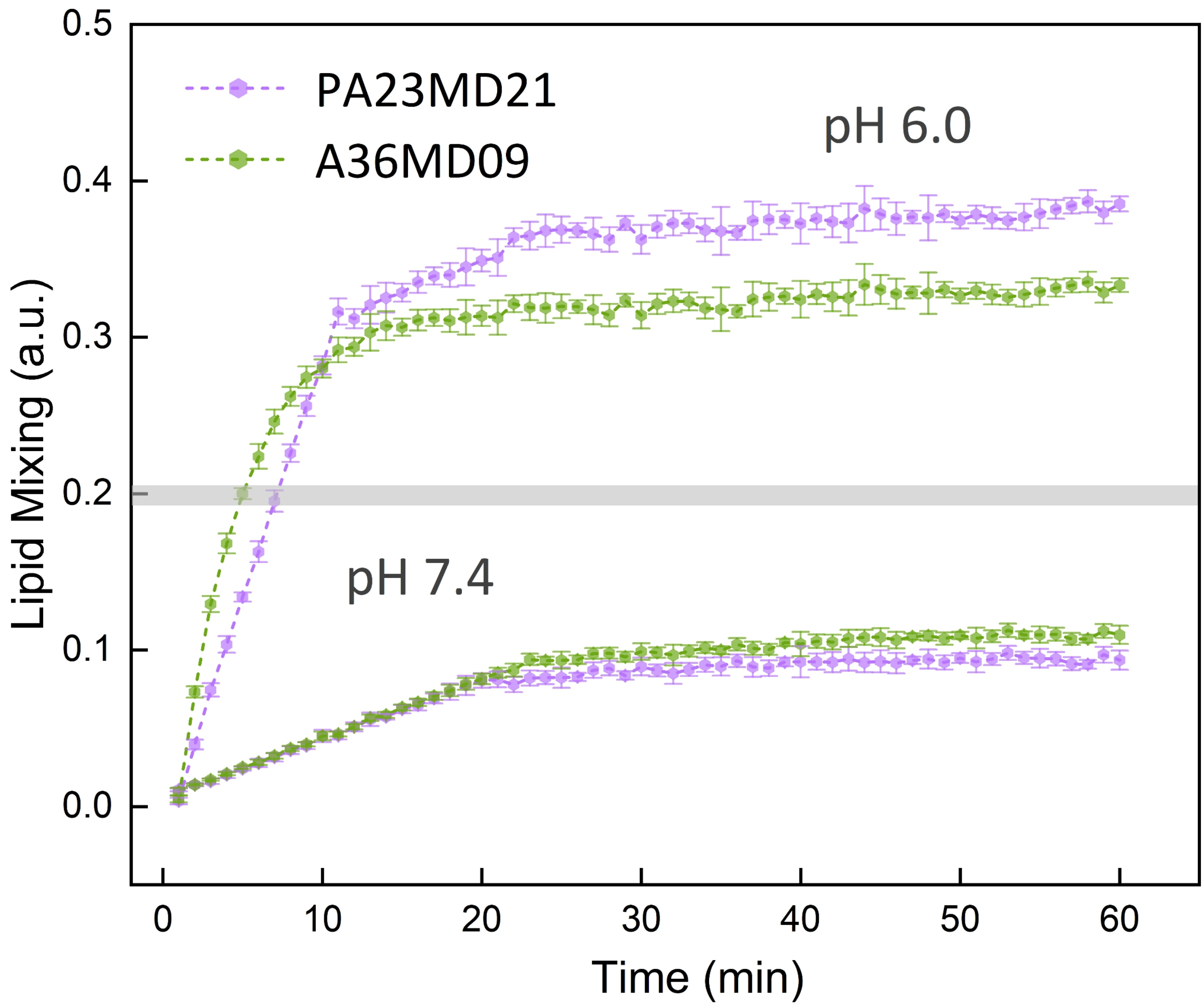

Supplementary Fig. 24. Kinetics of pH-dependent lipid mixing assayed by FRET. The membrane fusion capability of LNPs with model anionic liposomes was monitored over 60 min. The graph compares the percentage of lipid mixing at pH 6.0 (solid lines, circles), simulating the acidic endosomal environment, versus pH 7.4 (dashed lines, squares), representing physiological conditions. Data are presented as mean ± SD (n=3).

a

Sample	pKa
SM102	6.72
PA86MD16	7.19
PA23MD21	7.59
PA97MD101	6.85
PA97MD21	7.14
A36MD09	7.29

b

Sample	Encapsulation Efficiency (EE%)	Standard Deviation
A36MD09	88.250232	0.487695
PA23MD21	88.701338	0.508115
PA86MD16	89.582719	0.211792
PA97MD101	91.191365	0.584366
PA97MD21	89.485920	0.488928
SM102	91.427720	0.658613

Supplementary Fig. 25. Assessment of apparent pKa values and mRNA encapsulation efficiencies of lead LNP formulations. (**Upper**) Apparent pKa values determined via the TNS (6-(p-toluidinyl) naphthalene-2-sulfonate) fluorescence titration assay. The table provides a comparative analysis of the experimental hit (A36MD09), the commercial benchmark (SM102), and the top-ranked ML-prioritized candidates. The observed pKa values for most candidates fall within the optimal range of 6.5–7.6, which is thermodynamically favorable for triggering membrane destabilization within acidic endosomes while ensuring nanoparticle stability during systemic circulation. (**Lower**) Quantification of mRNA encapsulation efficiency (EE%) for selected LNP formulations. The EE% was evaluated using the RiboGreen fluorescence assay for the lead experimental lipid, the top four predicted candidates, and SM102. All formulations achieved high cargo loading (>85%), demonstrating the robust RNA-binding affinity and effective condensation capabilities of the ionizable AMD lipid scaffolds. Data represent mean ± SD (*n* = 3 biologically independent samples).

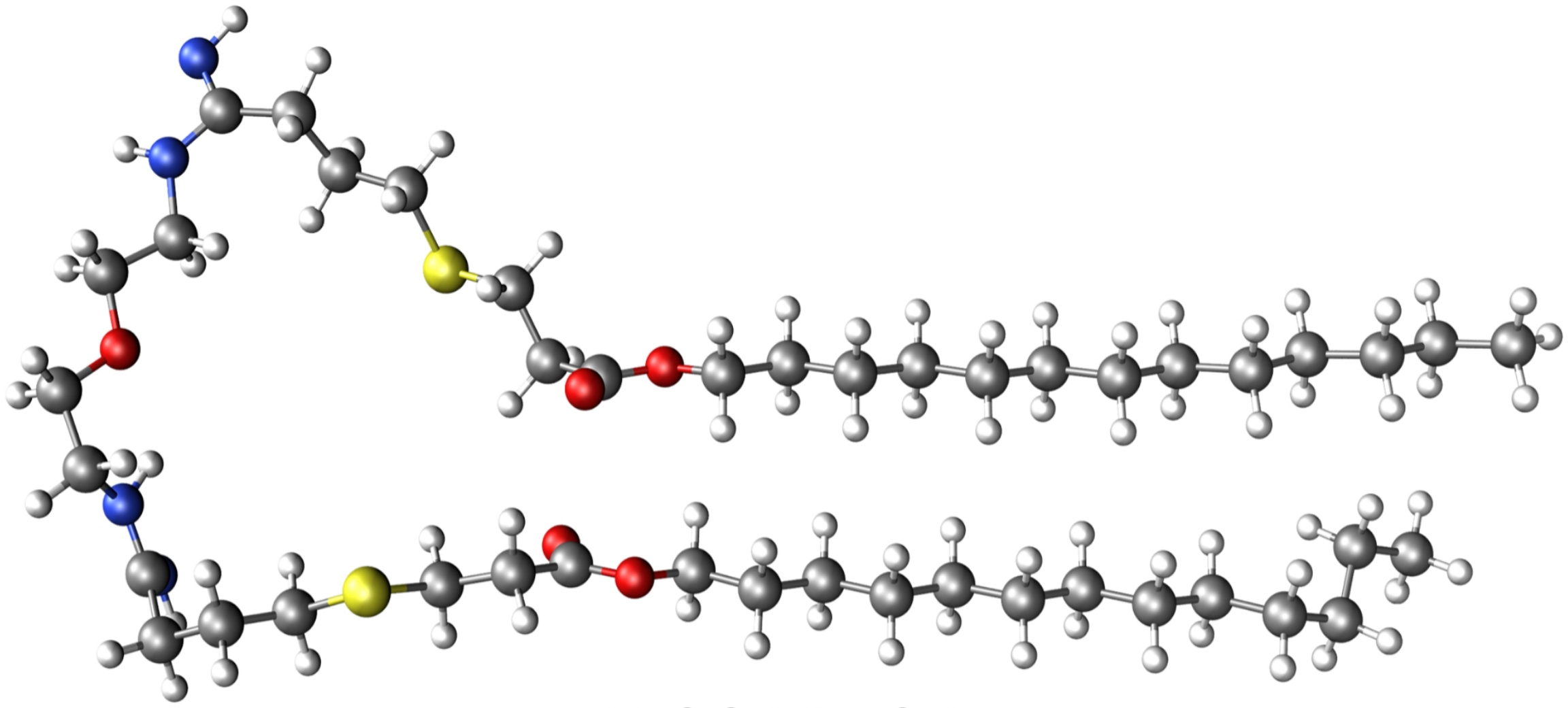

PA23MD21

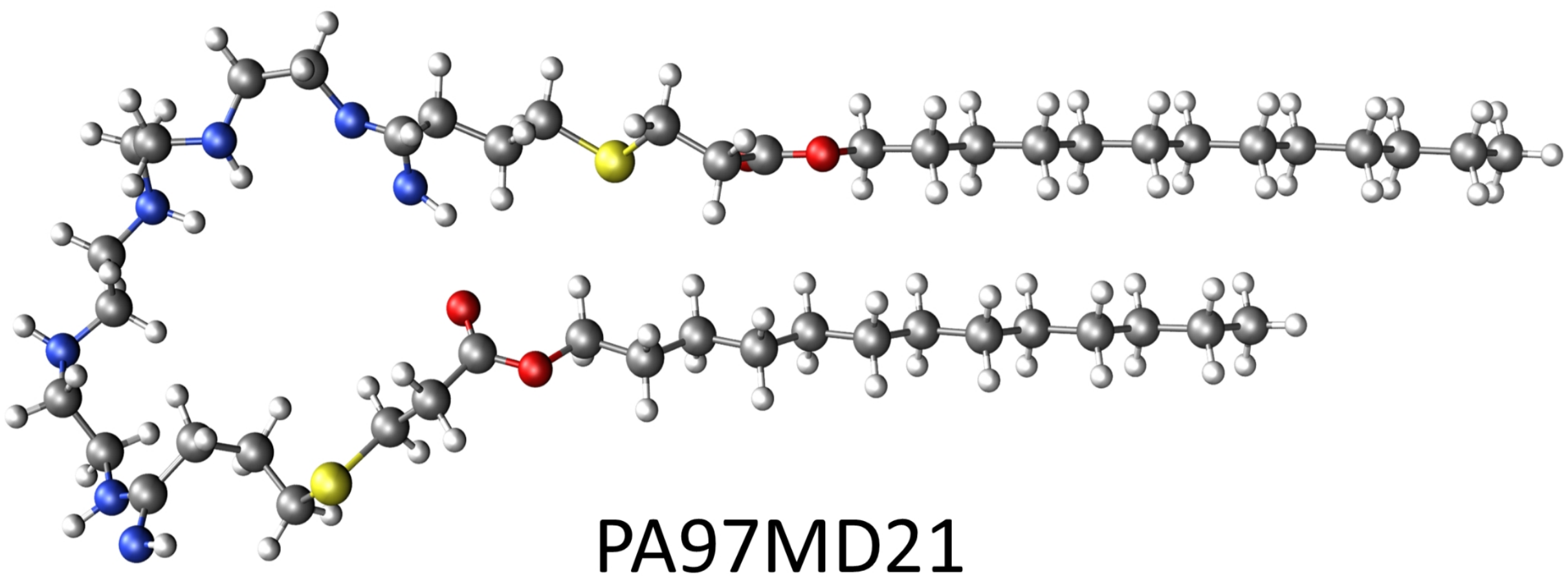

PA97MD21

Supplementary Fig. 26. Optimized molecular geometries of selected PAMD lipids. The energy-minimized 3D conformations of the other top-ranked lipid candidates (No. PA23MD21, PA97MD21) were obtained via computational modeling. The ball-and-stick models illustrate the spatial arrangement of the ionizable headgroups, linkers, and hydrophobic tails. Atoms are color-coded: carbon (gray), oxygen (red), nitrogen (blue), sulfur (yellow), and hydrogen (white).

LNP	PA23MD21	PA97MD21
V (Å^3)	884.87	991.93
A (Å^2)	15.09	22.30
L (Å^1)	15.52	10.73
P	3.78	4.15
Angle	19.24	21.20

Supplementary Fig. 27. Calculated molecular geometric parameters of the top-ranked PAMD lipids. The structural descriptors were derived from the energy-minimized conformations shown in Figure S26. V (Å^3): Molecular volume; A (Å^2): Molecular surface area or headgroup area; L (Å): Molecular length; P: Packing parameter/Shape factor, indicating the spatial geometry of the lipid; Angle: The cone angle of the lipid molecule. These parameters quantitatively characterize the steric properties that influence LNP assembly and endosomal escape efficiency.

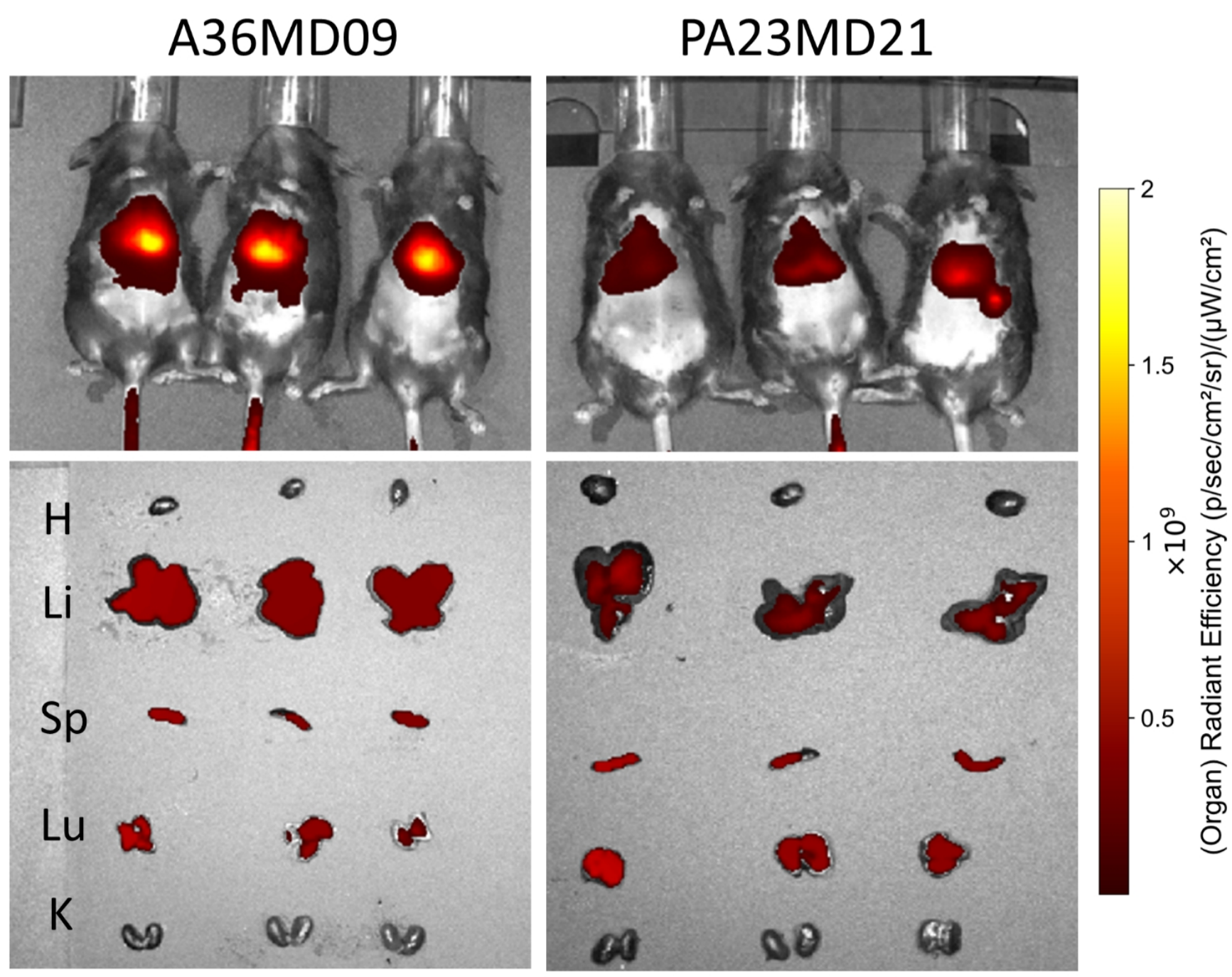

Supplementary Fig. 28. Representative *ex vivo* fluorescence images of major organs 6h post-administration of DIR-LNPs for mice.

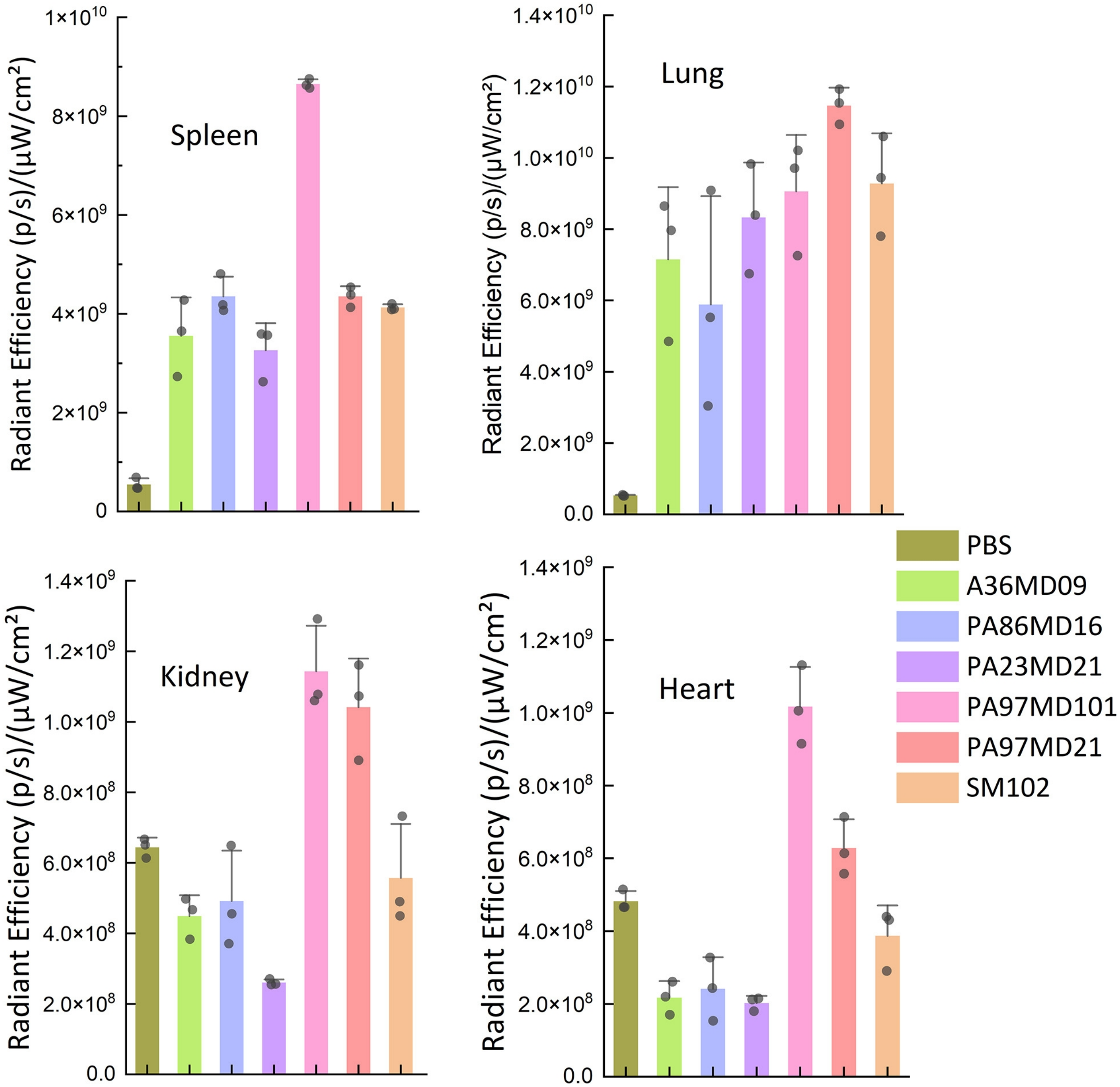

Supplementary Fig. 29. Quantitative organ-level biodistribution. Region-of-interest analysis was performed on ex vivo fluorescence images to quantify the accumulation of DiR-labelled LNPs in the heart, liver, spleen, lungs and kidneys. The lead formulations showed liver-dominant fluorescence under the tested conditions. These measurements report organ-level nanoparticle-associated fluorescence and do not establish hepatocyte-specific delivery or receptor-mediated targeting. Data are presented as mean $\pm$ SD (n = 3 biologically independent mice).

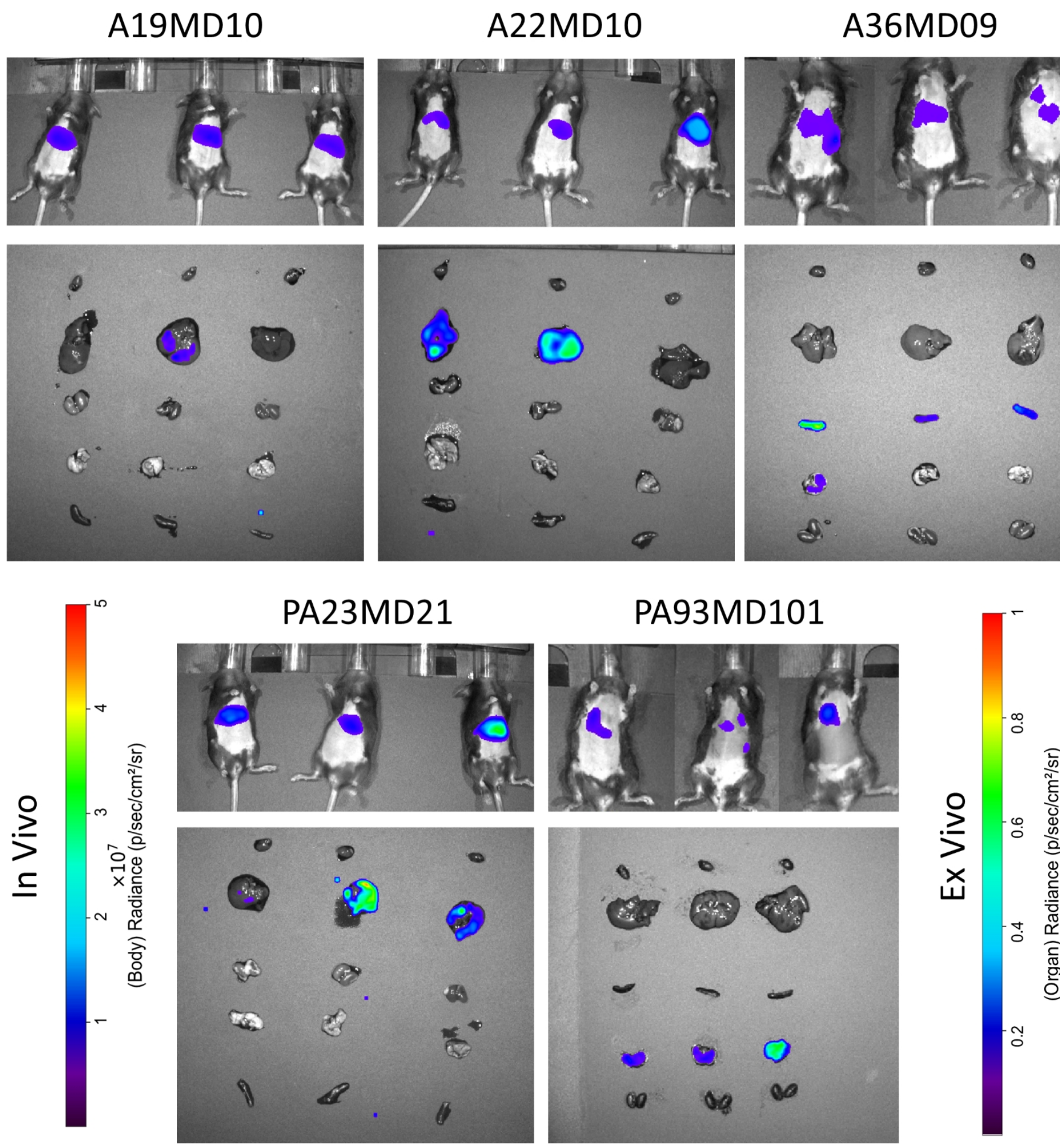

Supplementary Fig. 30. In vivo functional-expression profiles of additional candidate mRNA–LNPs. Representative whole-body and ex vivo organ bioluminescence images acquired 6 h after intravenous administration of Fluc mRNA formulated in A19MD10, A22MD10, A36MD09, PA23MD21 or PA93MD101 LNPs (0.1 mg kg^{-1} mRNA). Three biologically independent mice are shown for each formulation. Bioluminescence reports productive Fluc mRNA delivery and translation and should not be interpreted as a direct measurement of nanoparticle mass distribution.

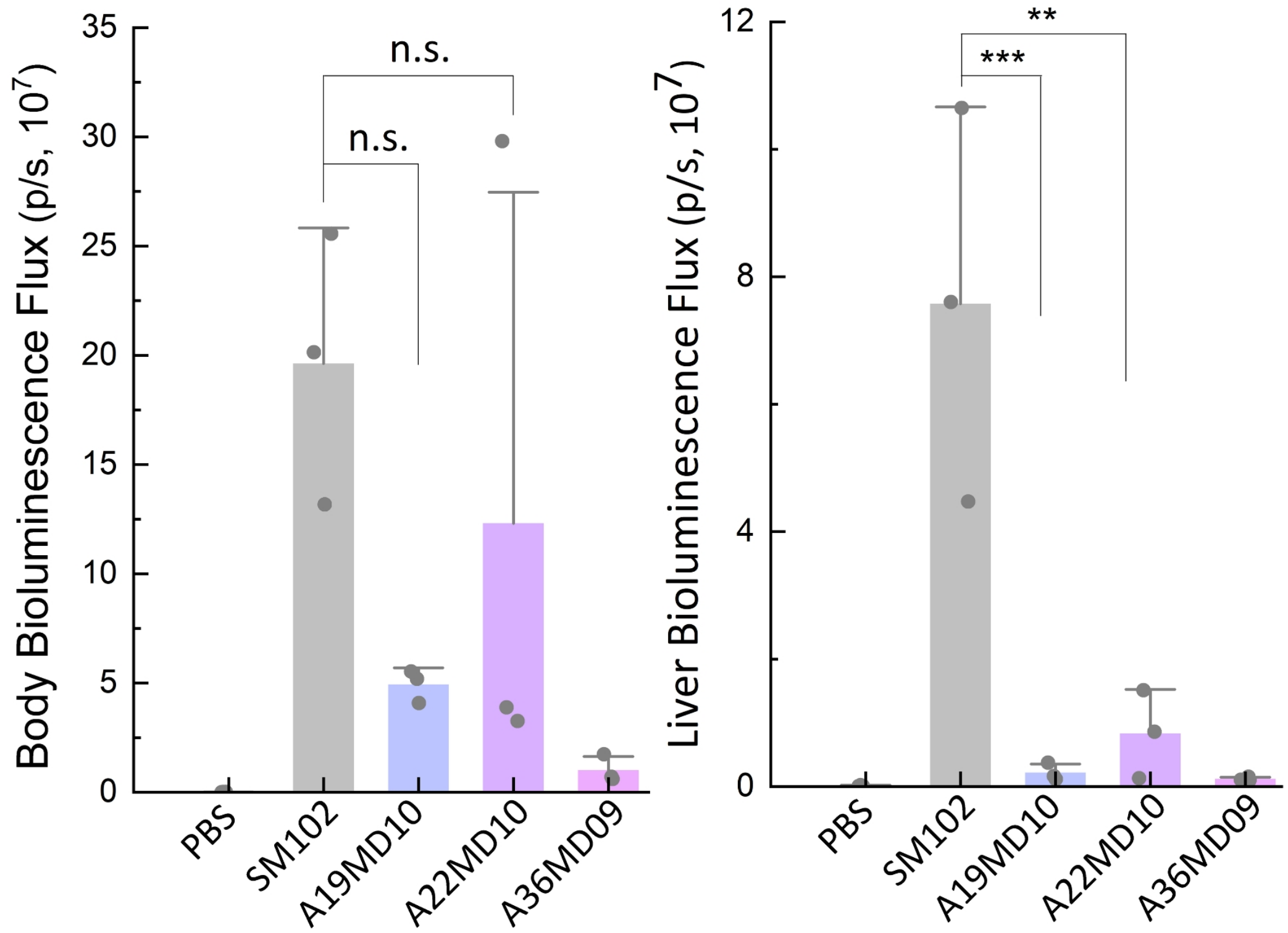

Supplementary Fig. 31. Quantification of whole-body and hepatic Fluc expression mediated by representative AMD LNPs. Whole-body total bioluminescence flux (left) and *ex vivo* liver flux (right) measured 6 h after intravenous administration of Fluc mRNA formulated in A19MD10, A22MD10 or A36MD09 LNPs, with PBS and SM102 included as controls. Data are mean ± s.d.; individual points represent biologically independent mice (n = 3 per group). Statistical comparisons were performed as described in the Methods; n.s., not significant; ***$P<0.001$.

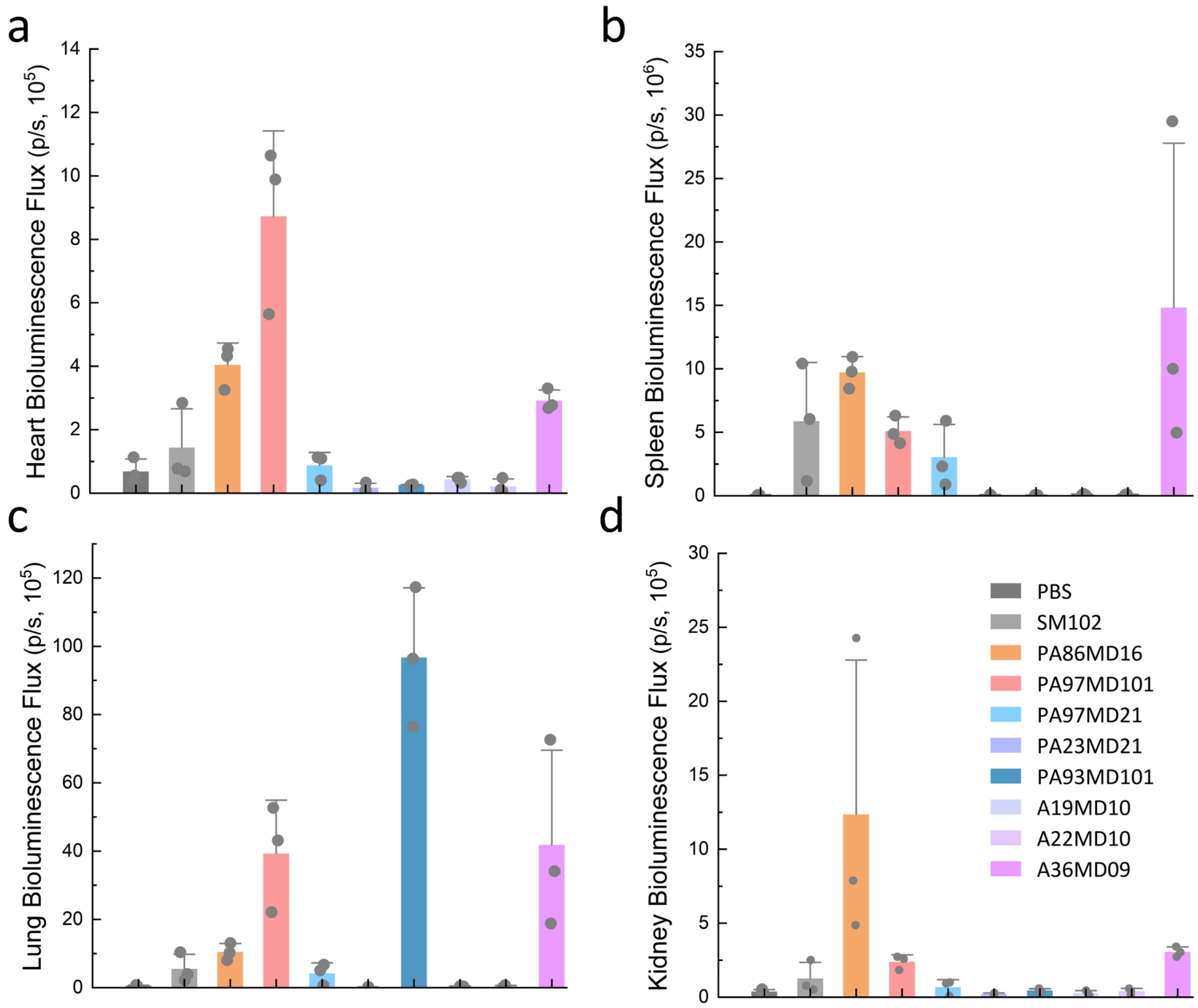

Supplementary Fig.32. Organ-resolved functional Fluc expression after systemic administration of candidate LNPs. Ex vivo Fluc bioluminescence flux in the heart (a), spleen (b), lungs (c) and kidneys (d) 6 h after intravenous administration of the indicated Fluc mRNA–LNPs. Data are mean ± s.d.; individual points represent biologically independent mice (*n* = 3 per group). Organ-specific y axes are independently scaled. These measurements report productive organ-level mRNA expression rather than nanoparticle mass biodistribution.

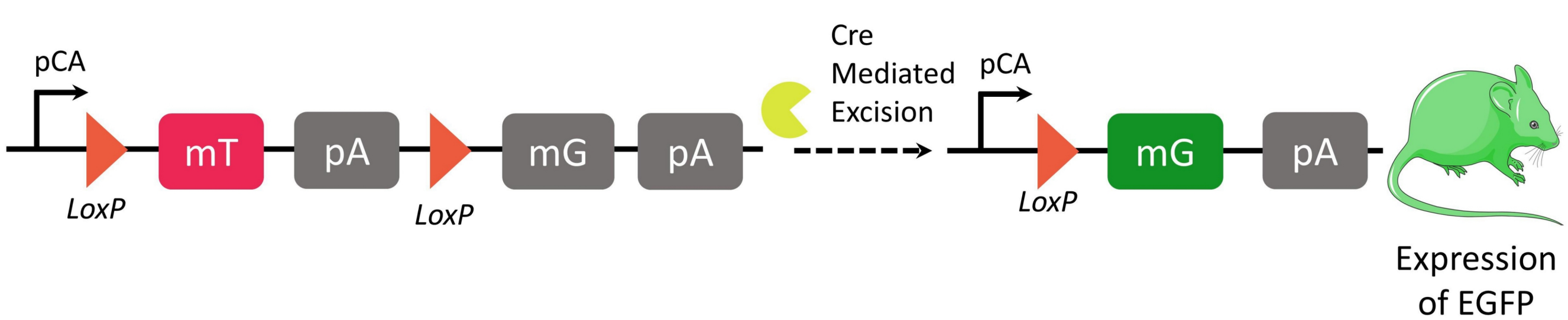

Supplementary Fig. 33. Schematic of the Cre/LoxP-mediated fluorescence switch (membrane-targeted tdTomato to membrane-targeted EGFP).

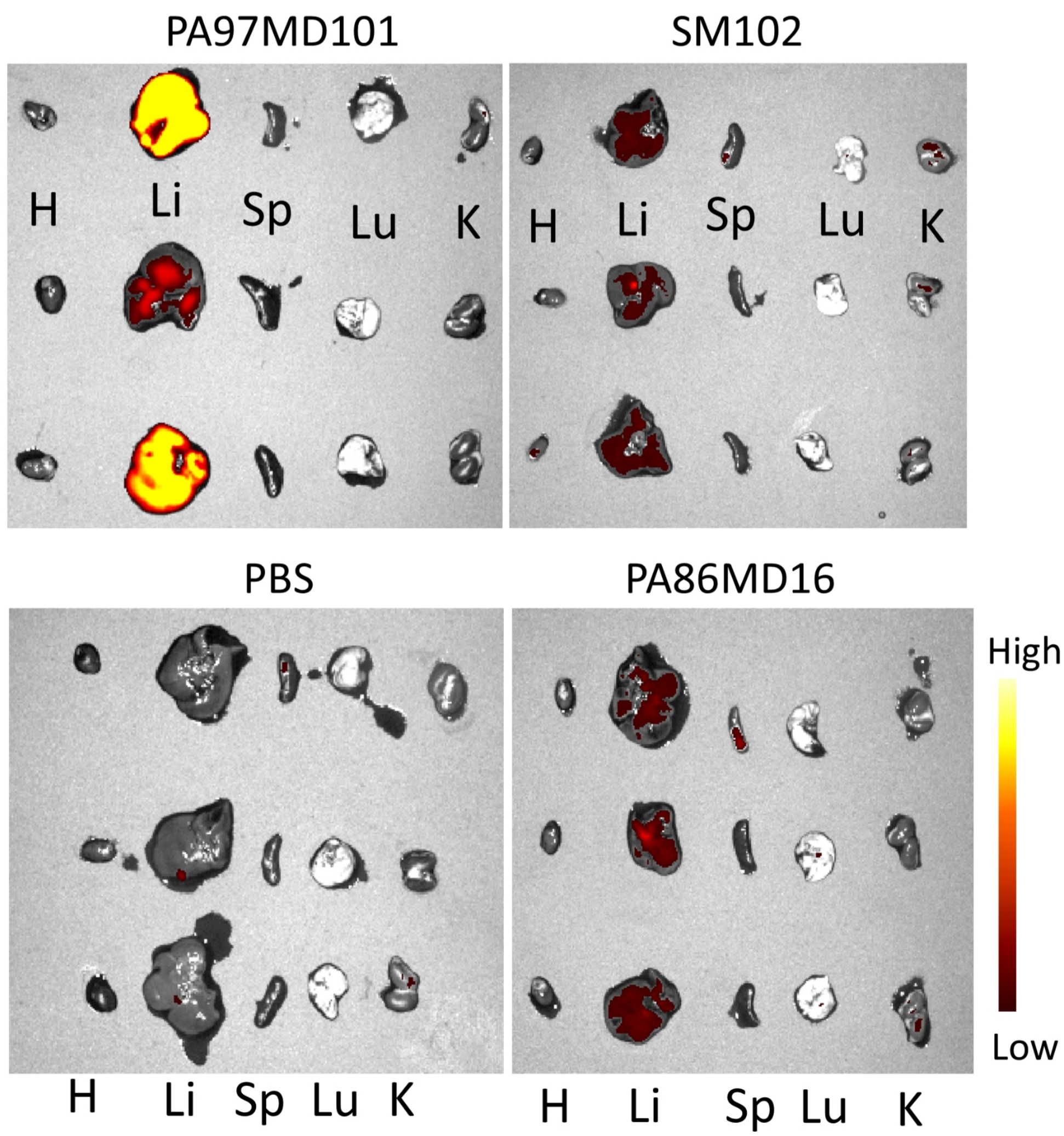

Supplementary Fig. 34. Cre-dependent tdTomato-to-EGFP recombination in mTmG reporter mice. Representative whole-body and ex vivo organ fluorescence images acquired 3 days after intravenous administration of Cre mRNA–LNPs (0.25 mg kg^{-1}). Following cytosolic translation, Cre recombinase enters the nucleus and induces permanent tdTomato-to-EGFP conversion. EGFP signals therefore report successful Cre expression and genomic recombination rather than nanoparticle uptake alone.

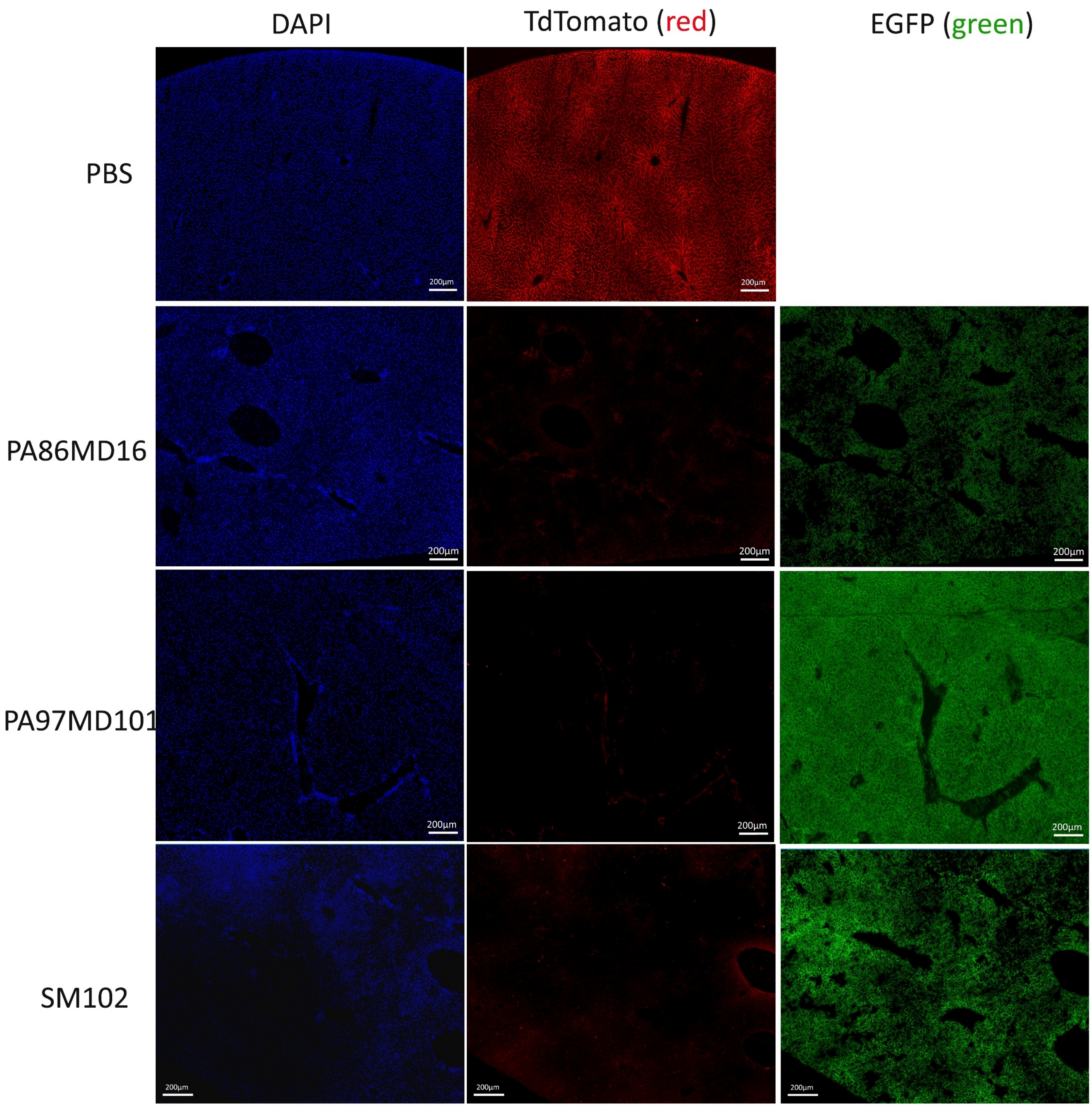

Supplementary Fig. 35. Representative confocal images of frozen liver sections showing EGFP (green) and tdTomato (red) expression. Nuclei were stained with DAPI (blue). Scale bars: 500 μm (top) and 100 μm (bottom).

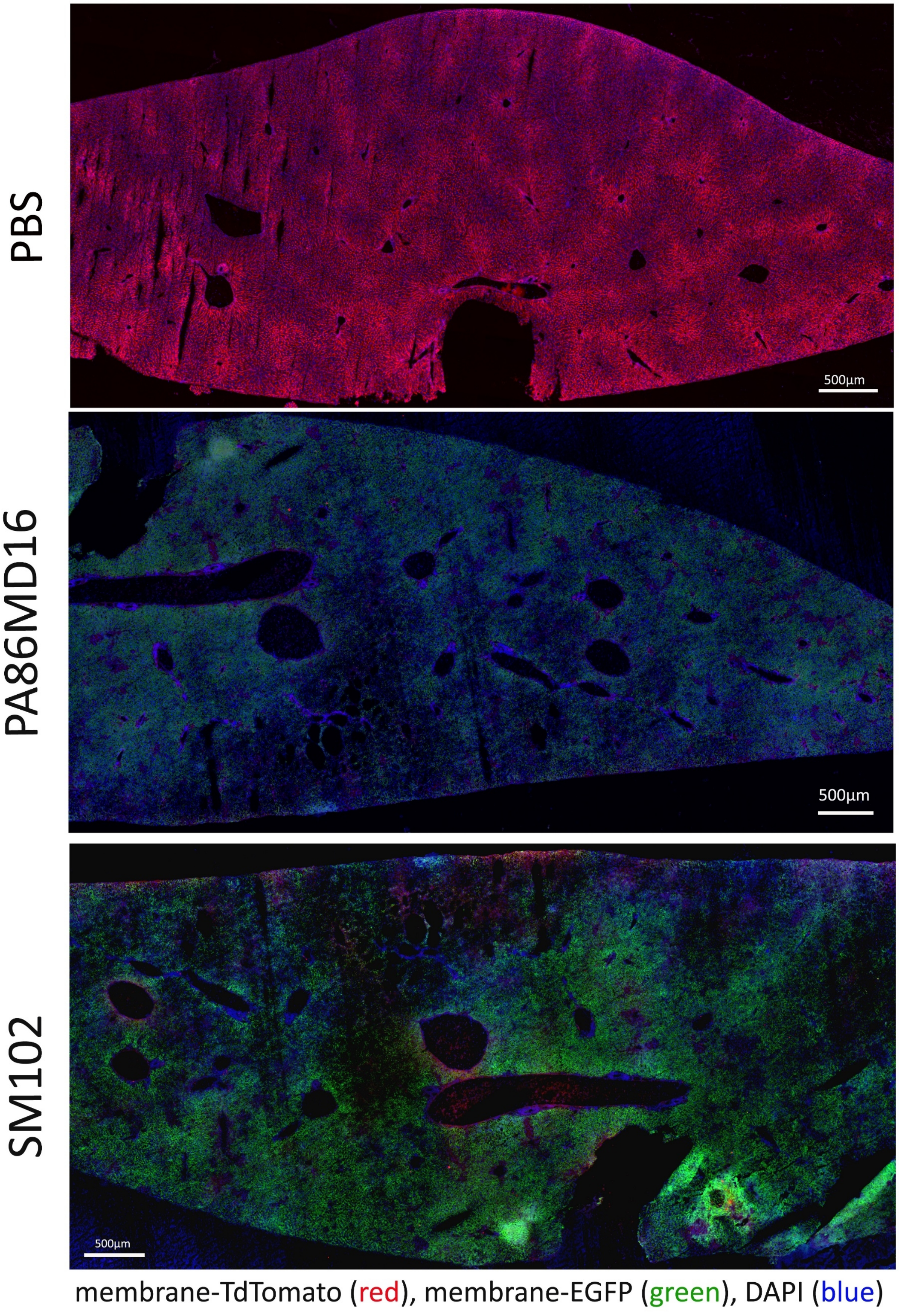

Supplementary Fig. 36. Representative confocal images of frozen liver sections showing EGFP (green) and tdTomato (red) expression. Nuclei were stained with DAPI (blue). Scale bars: 500 μm (top) and 100 μm (bottom).

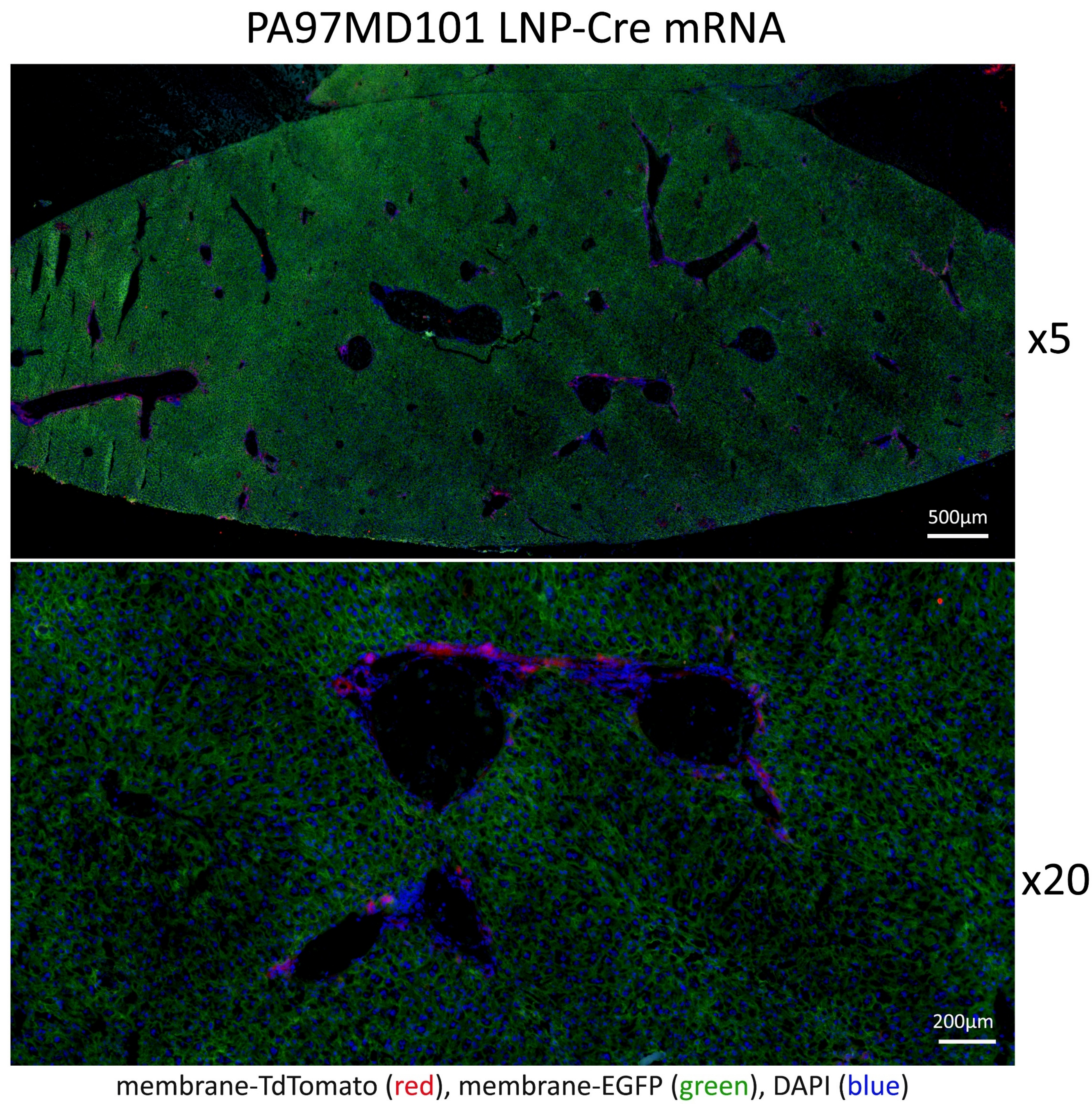

Supplementary Fig. 37. Representative confocal images of frozen liver sections showing EGFP (green) and tdTomato (red) expression for PA97MD101-Cre mRNA treated mice. Nuclei were stained with DAPI (blue). Scale bars: 500 µm (top) and 100 µm (bottom).

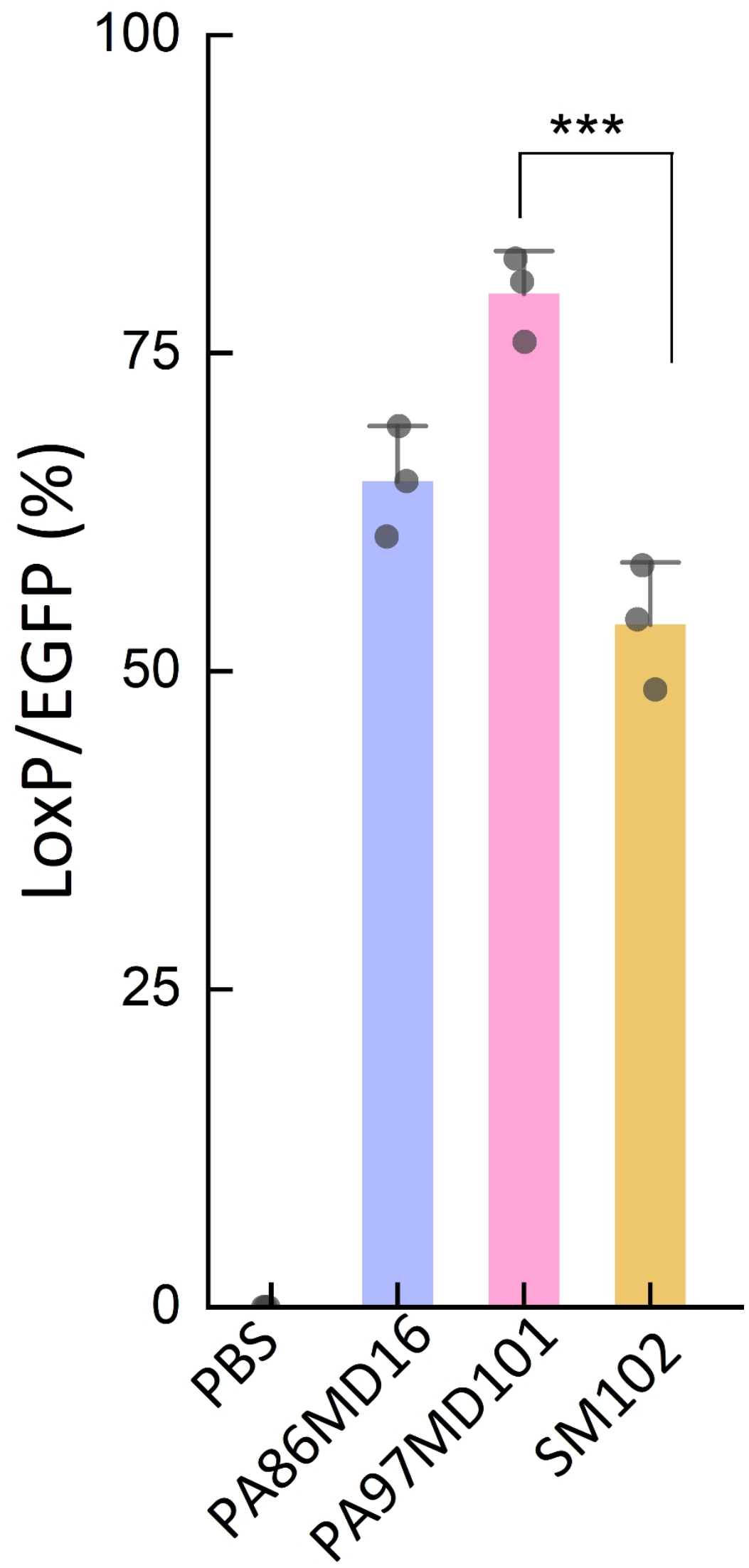

Supplementary Fig. 38. Genomic recombination efficiency quantified via qPCR using LoxP-specific primers.

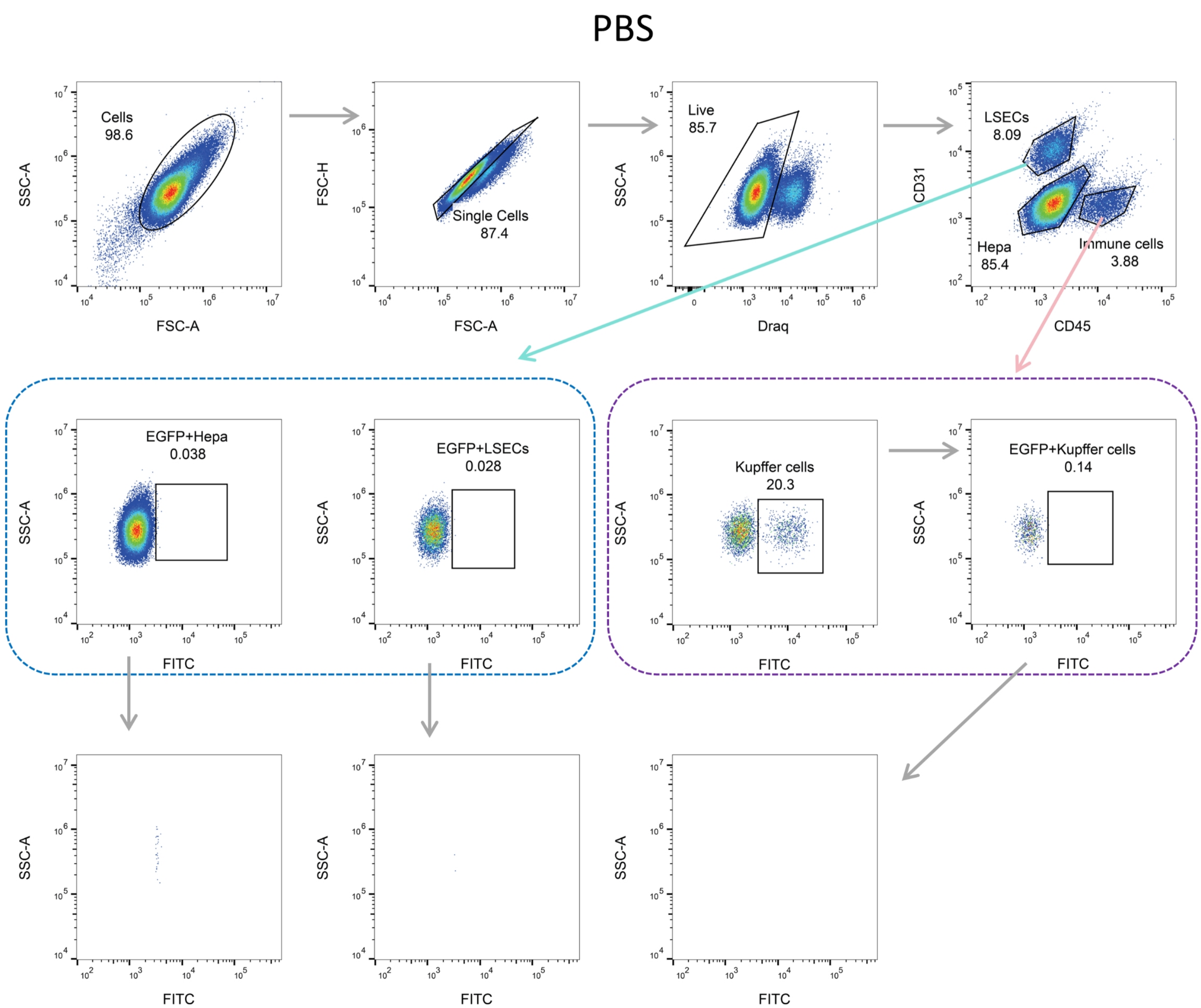

Supplementary Fig. 39. Cell-type-resolved Cre-dependent functional delivery by PBS in the liver. Representative flow-cytometric gating and pseudocolour plots from mTmG reporter mice 3 days after intravenous administration of PBS. Liver single-cell suspensions were sequentially gated for cells, singlets and viable cells. Viable cells were separated into a CD31$^-$CD45$^-$ hepatocyte-enriched population, CD31$^+$CD45$^-$ liver sinusoidal endothelial cells (LSECs) and CD45$^+$ immune cells; Kupffer cells were defined as F4/80$^+$ cells within the CD45$^+$ compartment. EGFP-positive fractions indicate Cre-dependent reporter recombination and functional Cre mRNA delivery. Numbers adjacent to gates denote percentages of the corresponding parent population. The plots are representative of n = 3 biologically independent mice.

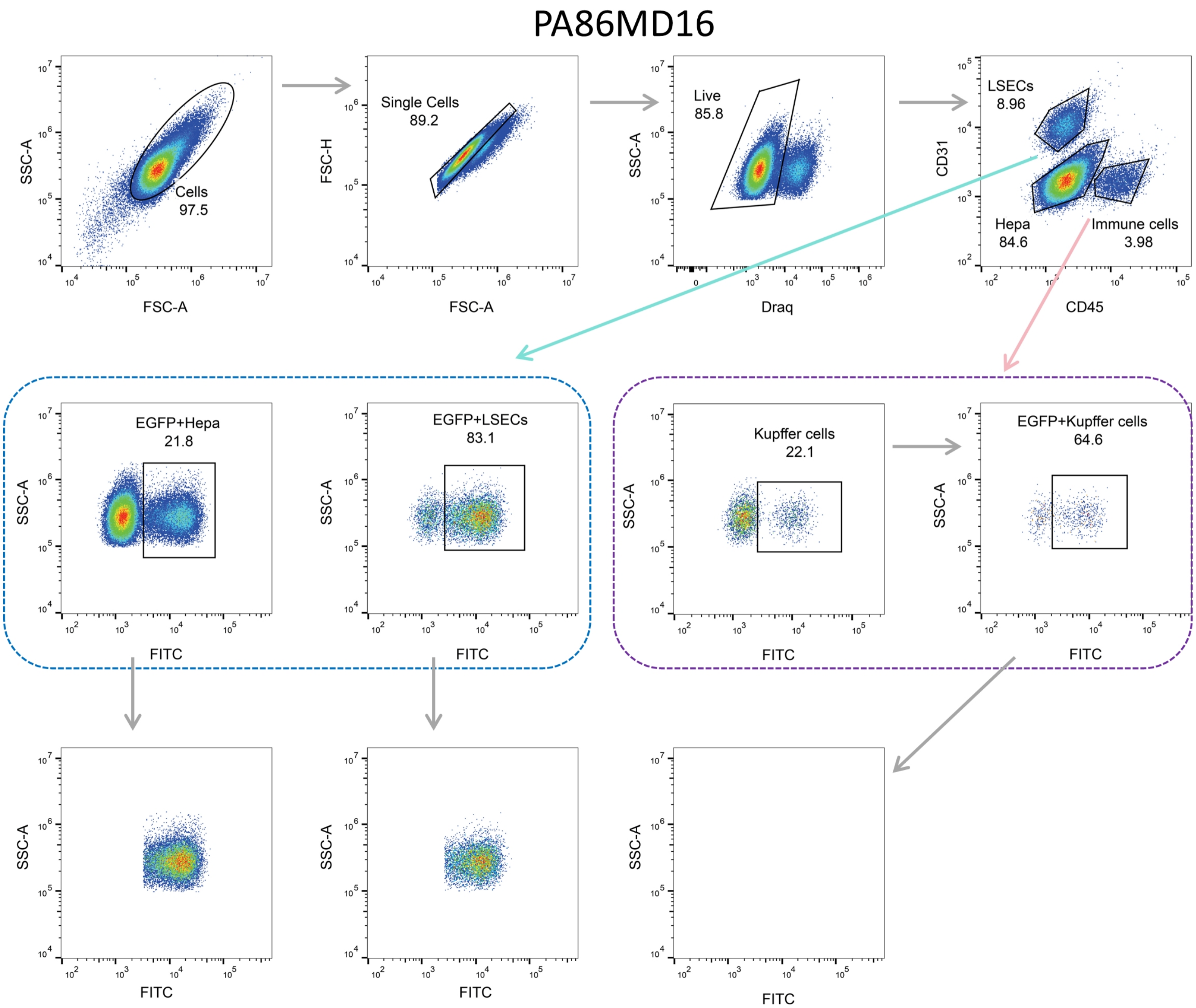

Supplementary Fig. 40. Cell-type-resolved Cre-dependent functional delivery by PA86MD16 in the liver. Representative flow-cytometric gating and pseudocolour plots from mTmG reporter mice 3 days after intravenous administration of Cre mRNA-loaded PA86MD16 LNPs. Liver single-cell suspensions were sequentially gated for cells, singlets and viable cells. Viable cells were separated into a $CD31^{-}CD45^{-}$ hepatocyte-enriched population, $CD31^{+}CD45^{-}$ liver sinusoidal endothelial cells (LSECs) and $CD45^{+}$ immune cells; Kupffer cells were defined as F4/80^{+} cells within the $CD45^{+}$ compartment. EGFP-positive fractions indicate Cre-dependent reporter recombination and functional Cre mRNA delivery. Numbers adjacent to gates denote percentages of the corresponding parent population. The plots are representative of n = 3 biologically independent mice.

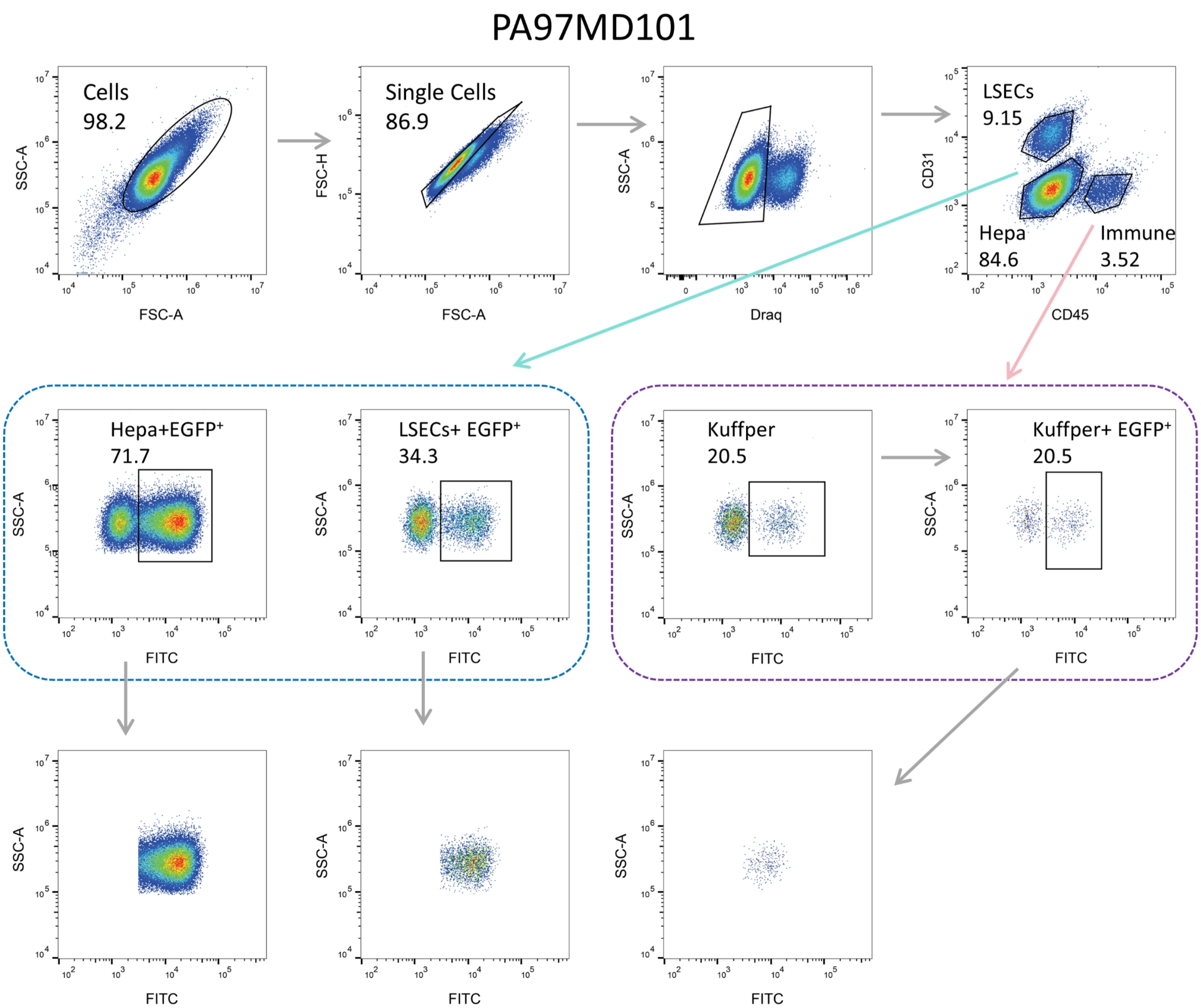

Supplementary Fig. 41. Cell-type-resolved Cre-dependent functional delivery by PA97MD101 in the liver. Representative flow-cytometric gating and pseudocolour plots from mTmG reporter mice 3 days after intravenous administration of Cre mRNA-loaded PA97MD101 LNPs. Liver single-cell suspensions were sequentially gated for cells, singlets and viable cells. Viable cells were separated into a $CD31^{-}CD45^{-}$ hepatocyte-enriched population, $CD31^{+}CD45^{-}$ liver sinusoidal endothelial cells (LSECs) and $CD45^{+}$ immune cells; Kupffer cells were defined as $F4/80^{+}$ cells within the $CD45^{+}$ compartment. EGFP-positive fractions indicate Cre-dependent reporter recombination and functional Cre mRNA delivery. Numbers adjacent to gates denote percentages of the corresponding parent population. The plots are representative of n = 3 biologically independent mice.

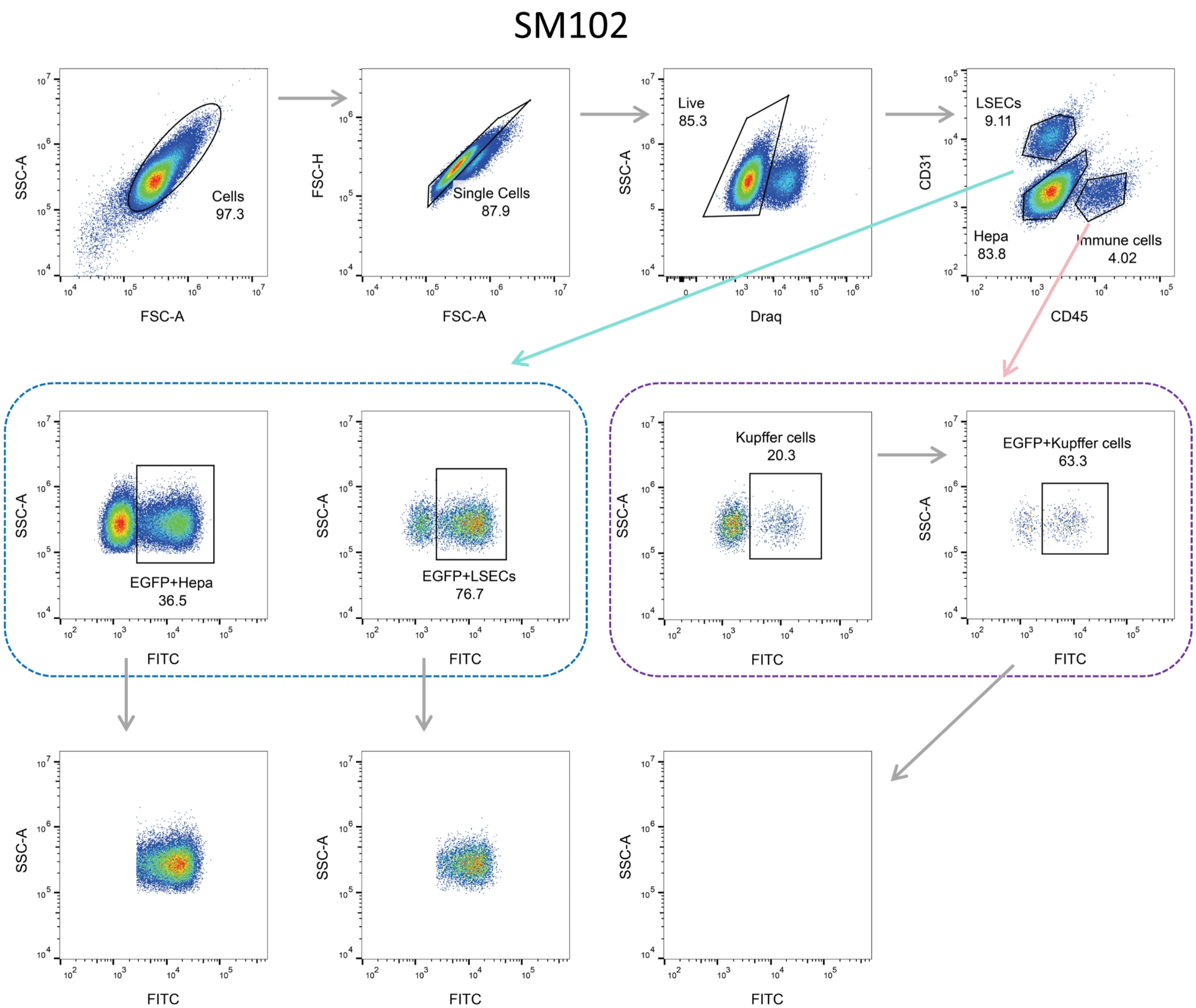

Supplementary Fig. 42. Cell-type-resolved Cre-dependent functional delivery by SM102 in the liver. Representative flow-cytometric gating and pseudocolour plots from mTmG reporter mice 3 days after intravenous administration of Cre mRNA-loaded SM102 LNPs. Liver single-cell suspensions were sequentially gated for cells, singlets and viable cells. Viable cells were separated into a $CD31^{-}CD45^{-}$ hepatocyte-enriched population, $CD31^{+}CD45^{-}$ liver sinusoidal endothelial cells (LSECs) and $CD45^{+}$ immune cells; Kupffer cells were defined as $F4/80^{+}$ cells within the $CD45^{+}$ compartment. EGFP-positive fractions indicate Cre-dependent reporter recombination and functional Cre mRNA delivery. Numbers adjacent to gates denote percentages of the corresponding parent population. The plots are representative of n = 3 biologically independent mice.

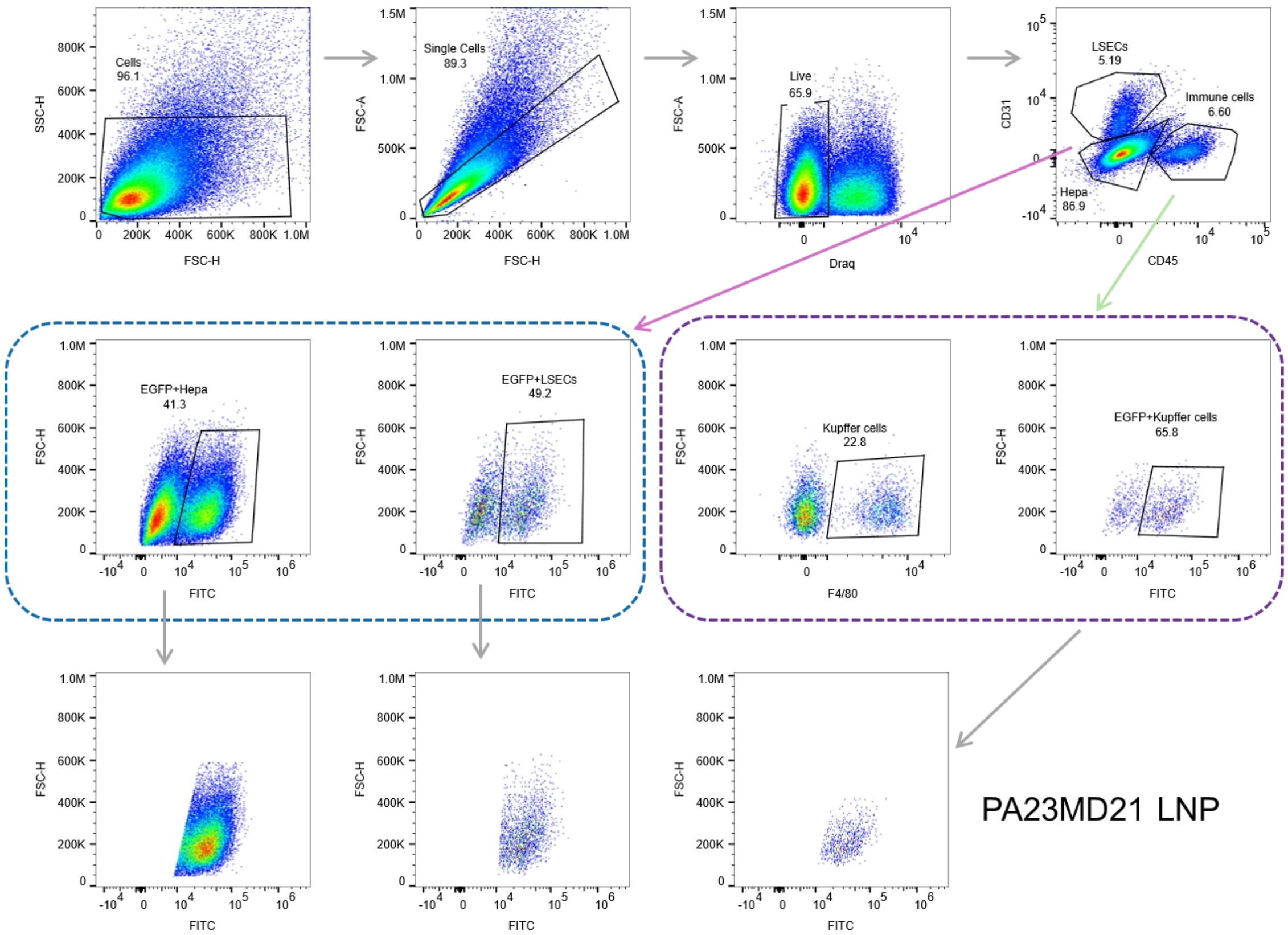

Supplementary Fig. 43. Cell-type-resolved Cre-dependent functional delivery by PA23MD21 in the liver. Representative flow-cytometric gating and pseudocolour plots from mTmG reporter mice 3 days after intravenous administration of Cre mRNA-loaded PA23MD21 LNPs. Liver single-cell suspensions were sequentially gated for cells, singlets and viable cells. Viable cells were separated into a $CD31^{-}CD45^{-}$ hepatocyte-enriched population, $CD31^{+}CD45^{-}$ liver sinusoidal endothelial cells (LSECs) and $CD45^{+}$ immune cells; Kupffer cells were defined as $F4/80^{+}$ cells within the $CD45^{+}$ compartment. EGFP-positive fractions indicate Cre-dependent reporter recombination and functional Cre mRNA delivery. Numbers adjacent to gates denote percentages of the corresponding parent population. The plots are representative of n = 3 biologically independent mice.

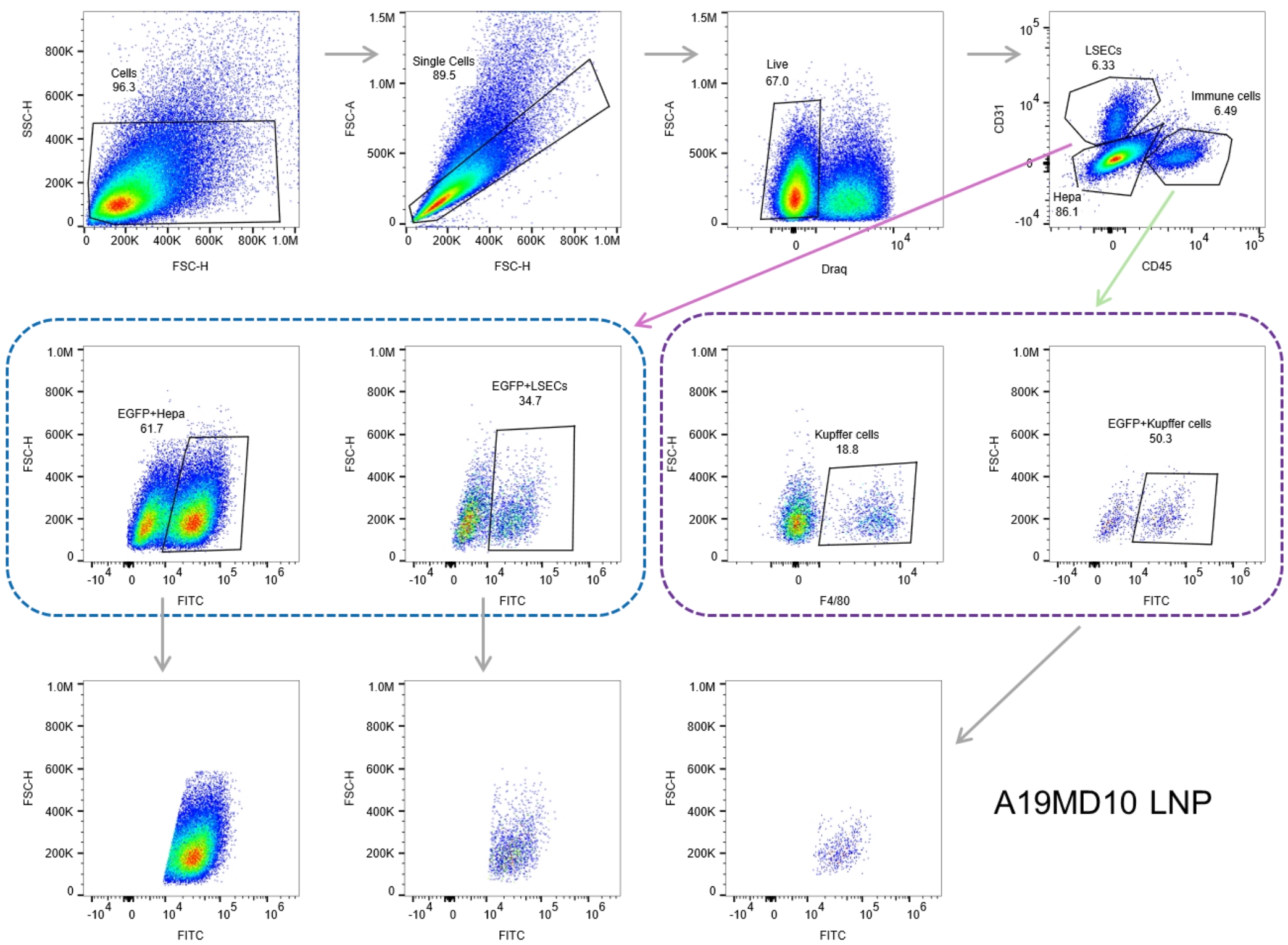

Supplementary Fig. 44. Cell-type-resolved Cre-dependent functional delivery by A19MD10 in the liver. Representative flow-cytometric gating and pseudocolour plots from mTmG reporter mice 3 days after intravenous administration of Cre mRNA-loaded A19MD10 LNPs. Liver single-cell suspensions were sequentially gated for cells, singlets and viable cells. Viable cells were separated into a $CD31^{-}CD45^{-}$ hepatocyte-enriched population, $CD31^{+}CD45^{-}$ LSECs and $CD45^{+}$ immune cells; Kupffer cells were defined as $F4/80^{+}$ cells within the $CD45^{+}$ compartment. EGFP-positive fractions indicate Cre-dependent reporter recombination and functional Cre mRNA delivery. Numbers adjacent to gates denote percentages of the corresponding parent population. The plots are representative of *n* = 3 biologically independent mice.

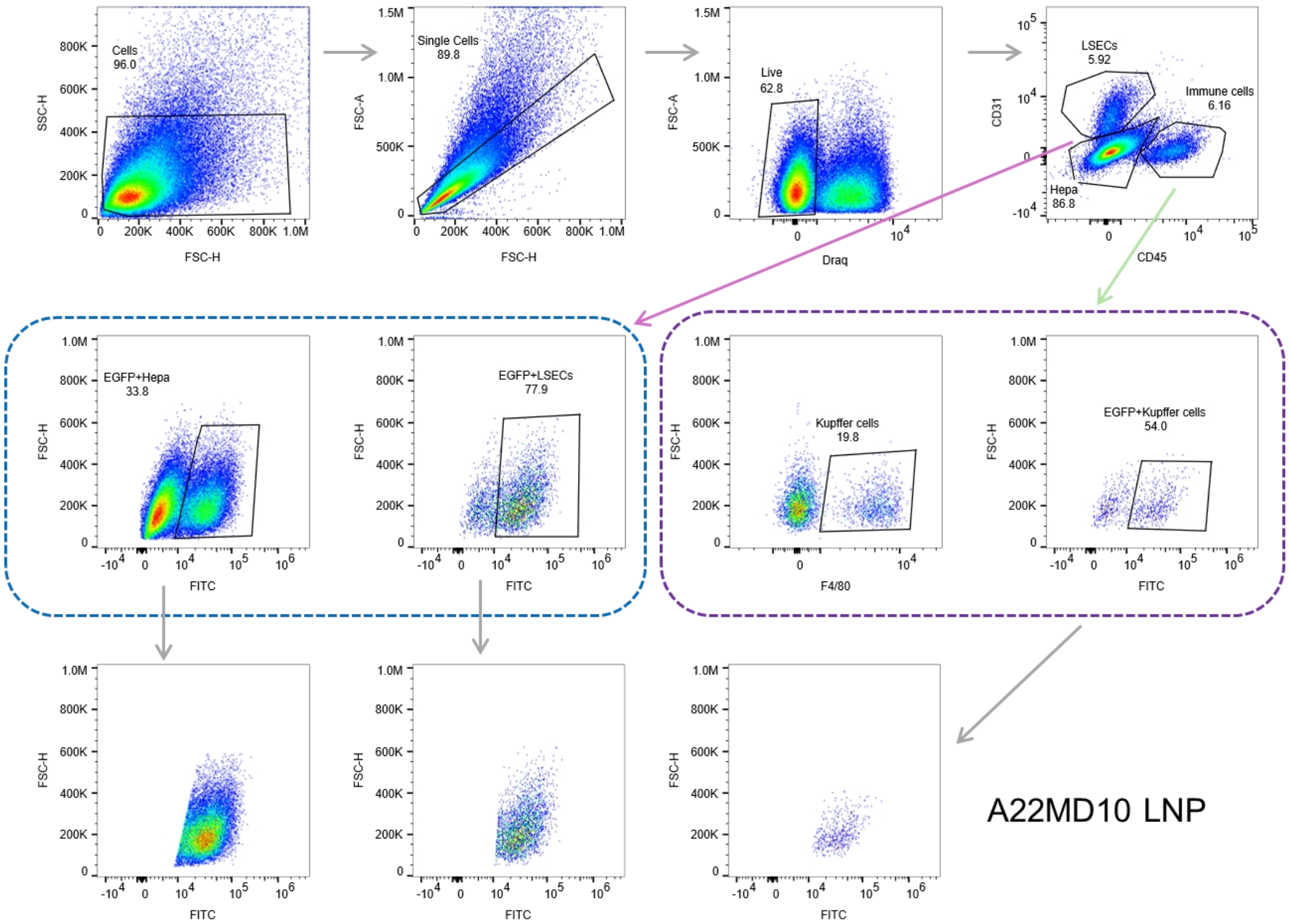

Supplementary Fig. 45. Cell-type-resolved Cre-dependent functional delivery by A22MD10 in the liver. Representative flow-cytometric gating and pseudocolour plots from mTmG reporter mice 3 days after intravenous administration of Cre mRNA-loaded A22MD10 LNPs. Liver single-cell suspensions were sequentially gated for cells, singlets and viable cells. Viable cells were separated into a $CD31^{-}CD45^{-}$ hepatocyte-enriched population, $CD31^{+}CD45^{-}$ LSECs and $CD45^{+}$ immune cells; Kupffer cells were defined as $F4/80^{+}$ cells within the $CD45^{+}$ compartment. EGFP-positive fractions indicate Cre-dependent reporter recombination and functional Cre mRNA delivery. Numbers adjacent to gates denote percentages of the corresponding parent population. The plots are representative of $n = 3$ biologically independent mice.

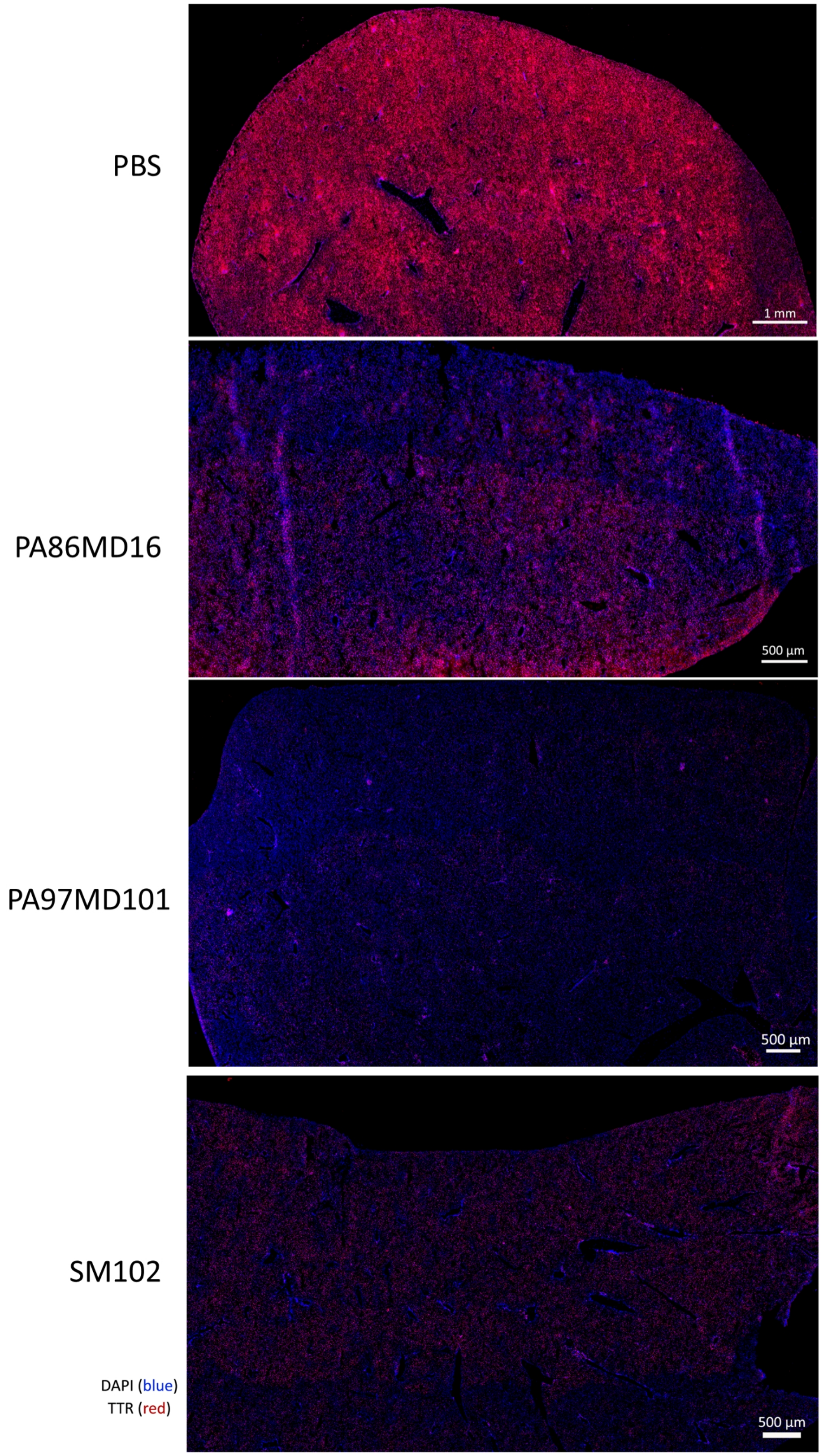

Supplementary Fig. 46. Representative fluorescence *in situ* hybridization (FISH) images showing

reduction of *Ttr* mRNA (red) in liver sections. Nuclei stained with DAPI (blue).

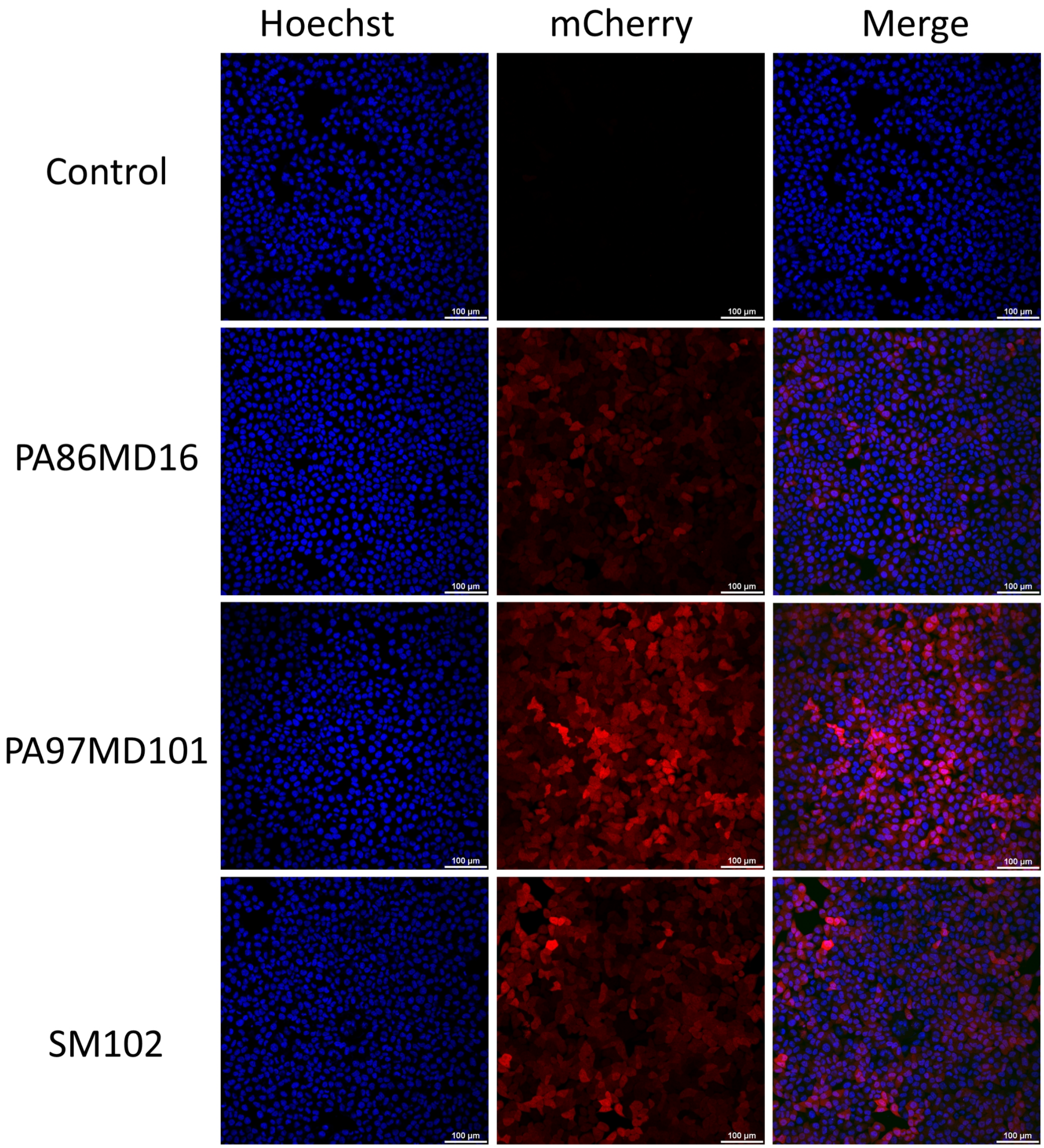

Supplementary Fig. 47. Verification of delivery versatility using mCherry mRNA reporter.

Fluorescence microscopy analysis of cells following incubation with mCherry-encoding LNPs. (Top row) Negative control (NC) showing no red fluorescence. (Middle & Bottom rows) Cells treated with Lipid PA86MD16, Lipid PA97MD101, and SM102 exhibit widespread and intense mCherry expression (red signals). Nuclei were counterstained with Hoechst 33342 (blue). Scale bar: 100 µm.

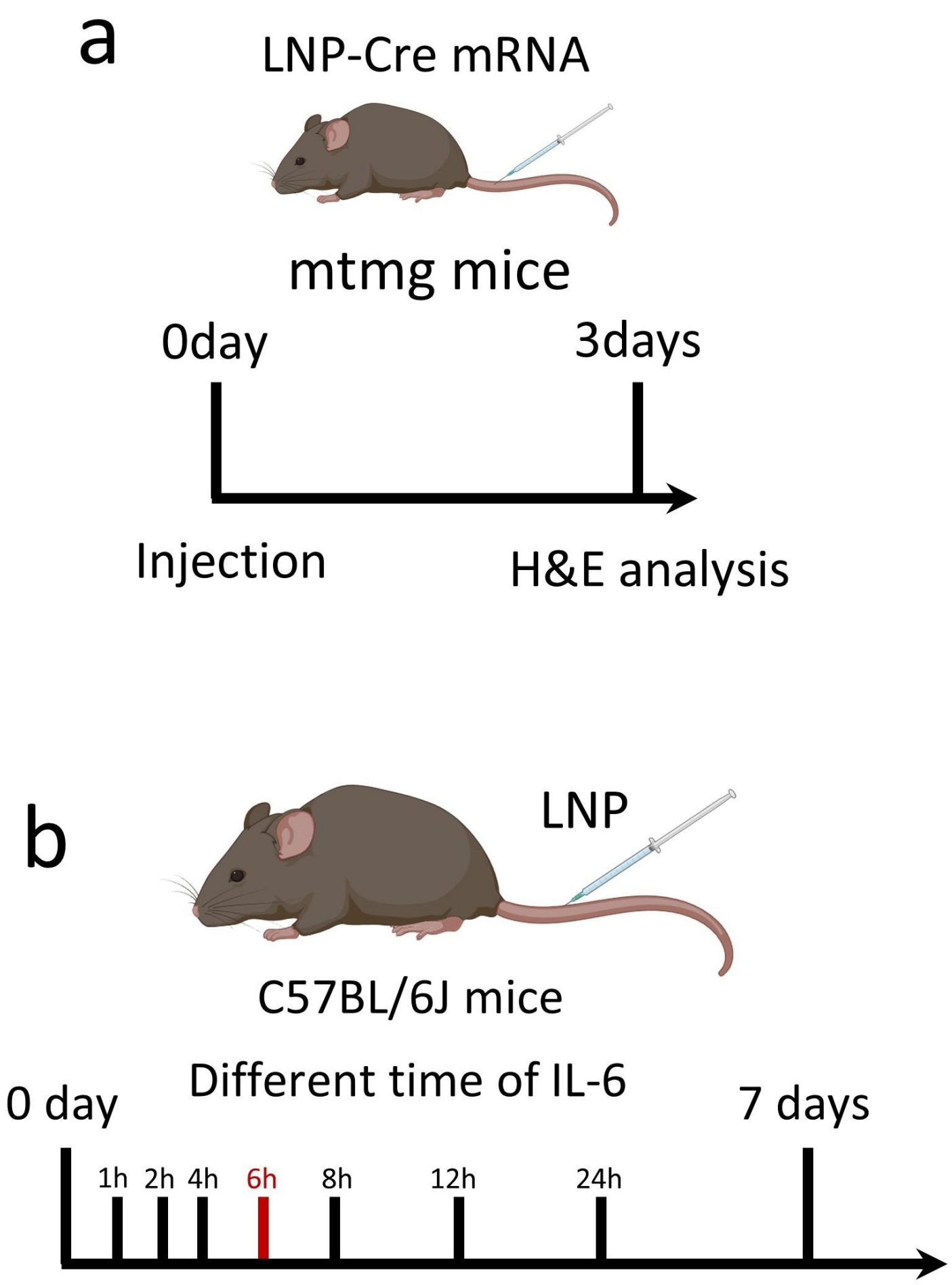

Supplementary Fig. 48. Schematic of the in vivo biosafety study design. Created in BioRender. Zhang, Z. (2026) https://BioRender.com/0gx5o01.

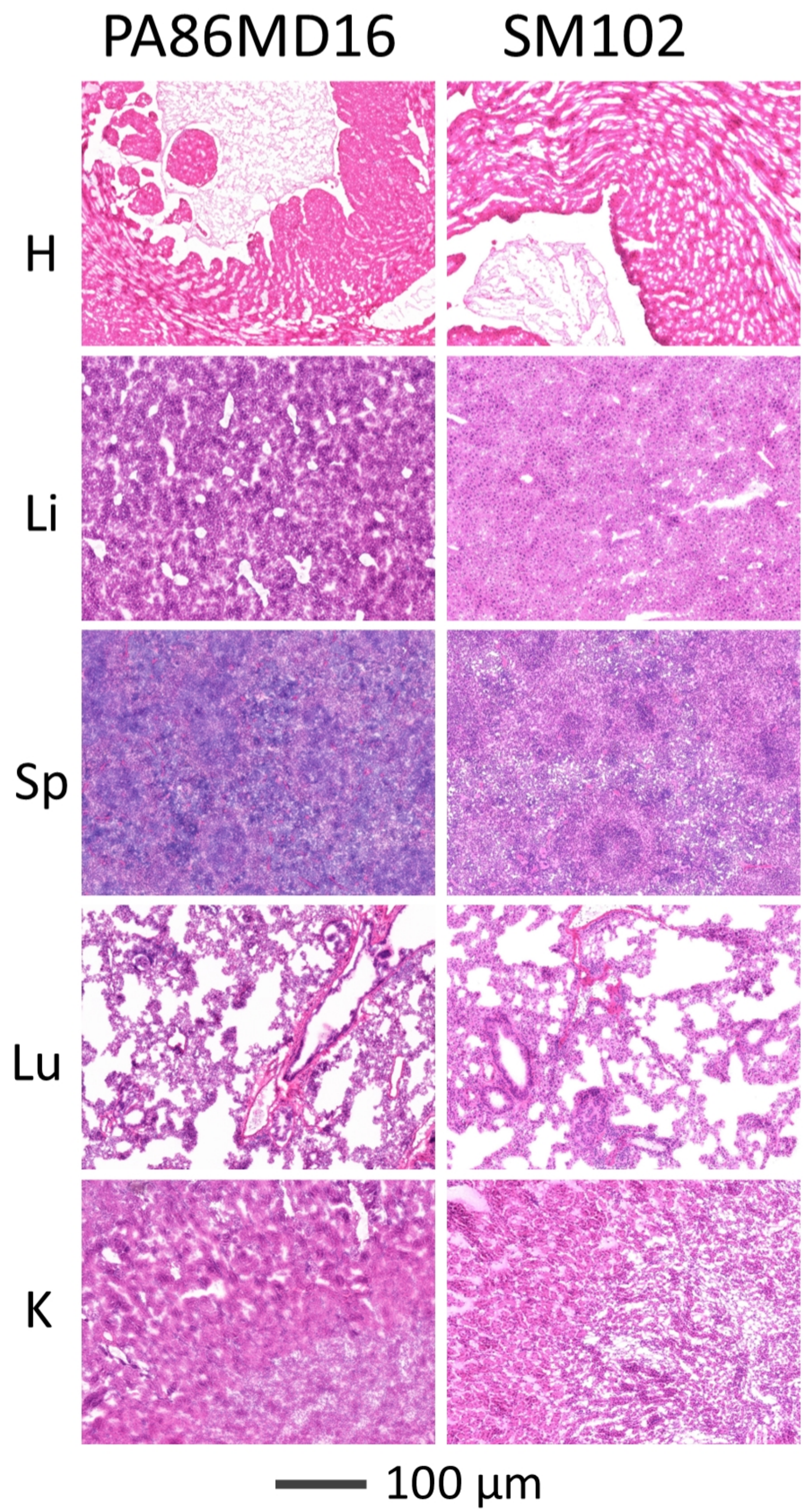

Supplementary Fig. 49. Representative H&E-stained sections of liver, spleen, and lung tissues assessing histological toxicity. Scale bar: 100 μm.
