# Supplementary material for: Interpretable AI-guided discovery of degradable ionizable lipids with enhanced hepatic mRNA delivery in vivo
Source: Nat Commun. 2026 Oct 12;17:11561. doi: 10.1038/s41467-026-78635-1 (PMC13748640; doi:10.1038/s41467-026-78635-1)
Supplement: Supplementary file 2 [file 41467_2026_78635_MOESM2_ESM.pdf]

**Title**: Supplementary Data 1

**Description**: Zeta-potential measurements for the 520-member AMD lipid library.

**Title**: Supplementary Data 2

**Description**: Hydrodynamic diameter and polydispersity-index measurements for the 520-member AMD lipid library.
