# Supplementary material for: Interpretable AI-guided discovery of degradable ionizable lipids with enhanced hepatic mRNA delivery in vivo
Source: Nat Commun. 2026 Oct 12;17:11561. doi: 10.1038/s41467-026-78635-1 (PMC13748640; doi:10.1038/s41467-026-78635-1)
Supplement: Supplementary file 3 [file 41467_2026_78635_MOESM5_ESM.pdf]

nature portfolio

Corresponding author(s): Feiyu Kang, Hao Yan

Last updated by author(s): Sep 14, 2026

Reporting Summary

Nature Portfolio wishes to improve the reproducibility of the work that we publish. This form provides structure for consistency and transparency in reporting. For further information on Nature Portfolio policies, see our Editorial Policies and the Editorial Policy Checklist.

Statistics

For all statistical analyses, confirm that the following items are present in the figure legend, table legend, main text, or Methods section.

n/a	Confirmed	
☐	☒	The exact sample size (*n*) for each experimental group/condition, given as a discrete number and unit of measurement
☐	☒	A statement on whether measurements were taken from distinct samples or whether the same sample was measured repeatedly
☐	☒	The statistical test(s) used AND whether they are one- or two-sided<br>*Only common tests should be described solely by name; describe more complex techniques in the Methods section.*
☒	☐	A description of all covariates tested
☐	☒	A description of any assumptions or corrections, such as tests of normality and adjustment for multiple comparisons
☐	☒	A full description of the statistical parameters including central tendency (e.g. means) or other basic estimates (e.g. regression coefficient) AND variation (e.g. standard deviation) or associated estimates of uncertainty (e.g. confidence intervals)
☐	☒	For null hypothesis testing, the test statistic (e.g. *F*, *t*, *r*) with confidence intervals, effect sizes, degrees of freedom and *P* value noted<br>*Give P values as exact values whenever suitable.*
☒	☐	For Bayesian analysis, information on the choice of priors and Markov chain Monte Carlo settings
☒	☐	For hierarchical and complex designs, identification of the appropriate level for tests and full reporting of outcomes
☐	☒	Estimates of effect sizes (e.g. Cohen's *d*, Pearson's *r*), indicating how they were calculated

Our web collection on statistics for biologists contains articles on many of the points above.

Software and code

Policy information about availability of computer code

Data collection	Particle size (hydrodynamic diameter), polydispersity index (PDI), and zeta potential were measured using a Zetasizer Nano ZS90 (Malvern Instruments). The chemical structures of synthesized lipids were confirmed via 1H NMR and 13C NMR spectroscopy on a Bruker 400 MHz spectrometer. Exact mass and molecular structure were verified using high-resolution quadrupole time-of-flight mass spectrometry (G2-S Q-TOF, Waters). LNP morphology was visualized using cryogenic transmission electron microscopy (Cryo-TEM; Talos L120C, Thermo Fisher Scientific) and standard transmission electron microscopy (TEM). mRNA encapsulation efficiency (EE%) was quantified using the Quant-iT™ RiboGreen™ RNA Reagent (Beijing Solarbio Science & Technology Co., Ltd., China) according to the manufacturer's protocol. The apparent acid dissociation constant (pKa) of the LNPs was determined using the 6-(p-toluidinyl)naphthalene-2-sulfonic acid (TNS) assay.<br>Molecular dynamics (MD) simulations: MD simulations of lipid bilayers were performed using GROMACS 2025.02. The AMBER19SB-Lipid21 and AMBER19SB-OL3 force fields were employed for lipids and RNA, respectively, with the OPC3 water model. For lipid parameterization, Restrained Electrostatic Potential (RESP) charges were calculated using ORCA 6.1.0 at the B3LYP-D3(BJ)/Def2TZVP level of theory. Bilayer simulations were conducted in the NPT ensemble at 298 K and 1 bar for 250 ns. Conformations from the final 50 ns were clustered using an RMSD threshold of 1 Å, and the most prevalent cluster was selected to calculate the packing parameter (P). For lipid–RNA interaction simulations, a 20-mer RNA strand (sequence: GACUGACUGACUGACUGACU) was constructed using Avogadro. The initial lipid–RNA complex was assembled using Packmol and simulated under NPT conditions (298 K, 1 bar) for 250 ns.
Data analysis	Statistical analyses and data visualization were conducted using Origin 2021 and GraphPad Prism (version 10.1.2). The artificial intelligence-guided lipid discovery, including machine learning model training and related computational analyses, was implemented using custom Python scripts (version 3.8.20) developed in Visual Studio Code.

For manuscripts utilizing custom algorithms or software that are central to the research but not yet described in published literature, software must be made available to editors and reviewers. We strongly encourage code deposition in a community repository (e.g. GitHub). See the Nature Portfolio guidelines for submitting code & software for further information.

Data

Policy information about availability of data

All manuscripts must include a data availability statement. This statement should provide the following information, where applicable:

- Accession codes, unique identifiers, or web links for publicly available datasets
- A description of any restrictions on data availability
- For clinical datasets or third party data, please ensure that the statement adheres to our policy

All data supporting the findings of this study are available within the Article, Supplementary Information and Source Data files. The machine-learning input data and processed outputs underlying the reported analyses are provided in the Source Data. Code used for molecular featurization, preprocessing, model training, validation, SHAP analysis and virtual-library prioritization is publicly available at https://github.com/thuZHANGZhen/AMD-PAMD-lipid-ML-code.

Research involving human participants, their data, or biological material

Policy information about studies with human participants or human data. See also policy information about sex, gender (identity/presentation), and sexual orientation and race, ethnicity and racism.

Reporting on sex and gender	This study did not involve human participants, human participant data, or primary human biological materials. Established human-derived cell lines were used, as reported in the Eukaryotic cell lines section.
Reporting on race, ethnicity, or other socially relevant groupings	No human participants or participant-level data were included; therefore, race, ethnicity and other socially relevant groupings were not collected or analysed.
Population characteristics	No human study population was included. The study used established human-derived cell lines, which are described in the Eukaryotic cell lines section.
Recruitment	No human participants were recruited for this study.
Ethics oversight	This study did not involve human participants, identifiable human data or primary human biological materials. Only established cell lines obtained from commercial or institutional cell repositories were used; therefore, human-subject ethics approval and informed consent were not required.

Note that full information on the approval of the study protocol must also be provided in the manuscript.

Field-specific reporting

Please select the one below that is the best fit for your research. If you are not sure, read the appropriate sections before making your selection.

☒ Life sciences ☐ Behavioural & social sciences ☐ Ecological, evolutionary & environmental sciences

For a reference copy of the document with all sections, see nature.com/documents/nr-reporting-summary-flat.pdf

Life sciences study design

All studies must disclose on these points even when the disclosure is negative.

Sample size	No formal power calculation was performed. Sample sizes were selected based on established LNP screening and in vivo delivery studies. The 520-member HeLa-cell screen used three technical replicate wells per lipid. In vivo studies generally used three biologically independent mice per group, as specified in the figure legends.
Data exclusions	No data were excluded from the analyses.
Replication	All attempts at replication were successful. The primary 520-member HeLa-cell screen was performed using three technical replicate wells per lipid. Follow-up cell-based assays and physicochemical characterization were repeated using independent experiments or independently prepared samples as specified in the corresponding figure legends. In vivo studies generally used three biologically independent mice per group, unless otherwise specified, with consistent results across the reported measurement modalities.
Randomization	C57BL/6 mice were randomly allocated to treatment groups before administration. In vitro treatment allocation was not randomized; formulations were assigned to predefined plate positions.
Blinding	LNP formulations were coded before administration, and the investigator performing intravenous injections was unaware of formulation identity. Quantitative imaging and flow-cytometry analyses were performed using predefined analysis settings.

Reporting for specific materials, systems and methods

We require information from authors about some types of materials, experimental systems and methods used in many studies. Here, indicate whether each material, system or method listed is relevant to your study. If you are not sure if a list item applies to your research, read the appropriate section before selecting a response.

Materials & experimental systems

n/a	Involved in the study	
	☒	Antibodies
	☒	Eukaryotic cell lines
☒		Palaeontology and archaeology
	☒	Animals and other organisms
☒		Clinical data
☒		Dual use research of concern
☒		Plants

Methods

n/a	Involved in the study	
☒		ChIP-seq
	☒	Flow cytometry
☒		MRI-based neuroimaging

Antibodies

Antibodies used	Brilliant Violet 605™ anti-mouse CD31 (Cat. No. 102427, BioLegend), PE/Cyanine7 anti-mouse CD45 (Cat. No. 103113, BioLegend), and Pacific Blue™ anti-mouse F4/80 (Cat. No. 123123, BioLegend). Samples were incubated at 4°C for 20–30 min in the dark. After washing with PBS, cells were resuspended in PBS containing 0.1% Draq7™ (to exclude dead cells).
Validation	Antibodies were commercially validated by BioLegend for flow cytometry and reactivity with mouse samples. Population boundaries were defined using PBS-treated mTmG controls and the marker combinations specified in the gating strategy. No independent antibody validation was performed in this study.

Eukaryotic cell lines

Policy information about cell lines and Sex and Gender in Research

Cell line source(s)	HeLa (ATCC CCL-2), HepG2 (ATCC CRL-10741) cells and HEK293T cells (ATCC CRL-3216) were purchased from Cell Bank of the Chinese Academy of Sciences.
Authentication	Cell-line identities were documented by the supplier. No additional authentication was performed after receipt.
Mycoplasma contamination	All cell lines were regularly tested for mycoplasma contamination using MycoFluor™ (M7006) Mycoplasma Detection Kit and consistently tested negative.
Commonly misidentified lines (See ICLAC register)	The cell lines were checked against the ICLAC register. HeLa is a recognized reference cell line; no evidence of cell-line misidentification was identified for the cultures used in this study.

Animals and other research organisms

Policy information about studies involving animals; ARRIVE guidelines recommended for reporting animal research, and Sex and Gender in Research

Laboratory animals	Specific pathogen-free (SPF) C57BL/6 mice (both female and male, 6-8 weeks old) were used for screening and verification of in vivo LNPs delivery efficiency. The mice were housed in a SPF facility with a 12-hour light/dark cycle, an ambient temperature of 22 ± 2 °C, and a humidity 40–70%. They had free access to standard rodent feed and water.
Wild animals	This study did not involve wild animals.
Reporting on sex	Male and female C57BL/6 mice aged 6–8 weeks were used. Sex was recorded based on external genital morphology and is provided for each animal in the Source Data. Experiments were not designed or powered for sex-stratified analysis. Sex was not used as an experimental factor, and no sex-stratified analysis was performed because the study was not powered to detect sex-dependent effects.
Field-collected samples	This study did not involve samples collected from the field.
Ethics oversight	All animal procedures were approved by the Institutional Animal Care and Use Committee of Tsinghua Shenzhen International Graduate School, Tsinghua University (protocol no. 2025F147), and were conducted in accordance with institutional guidelines.

Note that full information on the approval of the study protocol must also be provided in the manuscript.

Plants

Seed stocks

Report on the source of all seed stocks or other plant material used. If applicable, state the seed stock centre and catalogue number. If plant specimens were collected from the field, describe the collection location, date and sampling procedures.

Novel plant genotypes

Describe the methods by which all novel plant genotypes were produced. This includes those generated by transgenic approaches, gene editing, chemical/radiation-based mutagenesis and hybridization. For transgenic lines, describe the transformation method, the number of independent lines analyzed and the generation upon which experiments were performed. For gene-edited lines, describe the editor used, the endogenous sequence targeted for editing, the targeting guide RNA sequence (if applicable) and how the editor was applied.

Authentication

Describe any authentication procedures for each seed stock used or novel genotype generated. Describe any experiments used to assess the effect of a mutation and, where applicable, how potential secondary effects (e.g. second site T-DNA insertions, mosiacism, off-target gene editing) were examined.

Flow Cytometry

Plots

Confirm that:

☒ The axis labels state the marker and fluorochrome used (e.g. CD4-FITC).

☒ The axis scales are clearly visible. Include numbers along axes only for bottom left plot of group (a 'group' is an analysis of identical markers).

☒ All plots are contour plots with outliers or pseudocolor plots.

☒ A numerical value for number of cells or percentage (with statistics) is provided.

Methodology

Sample preparation

Livers were perfused through the portal vein with PBS containing 0.1% BSA and 0.2% EDTA, followed by perfusion with digestion buffer containing 0.5 mg mL^{-1} collagenase IV and 50 µg mL^{-1} DNase I. Liver tissues were minced, incubated in serum-free DMEM containing collagenase IV at 37 °C for 3–6 min with gentle agitation and pipetting, and filtered through 40-µm cell strainers. After centrifugation at 800 × g for 4 min, red blood cells were lysed with ACK lysis buffer for 6 min. Single-cell suspensions were stained with BV605 anti-mouse CD31, PE/Cy7 anti-mouse CD45 and Pacific Blue anti-mouse F4/80 antibodies. DRAQ7 was used to exclude dead cells.

Instrument

Data acquisition was performed on a CytoFLEX flow cytometer (Beckman Coulter), and data were analyzed using FlowJo software (v10.8.1).

Software

Data collection was performed using CytExpert software, and data were analyzed using FlowJo software (v10.8.1).

Cell population abundance

No pre-enrichment or cell sorting was performed. Population frequencies were quantified as percentages of the corresponding parent viable-cell population. $CD31^{-}CD45^{-}$ cells were reported as a hepatocyte-enriched population, $CD31^{+}CD45^{-}$ cells as an endothelial/LSEC-enriched population and $CD45^{+}$ cells as immune cells. Kupffer cells were defined as $F4/80^{+}$ cells within the $CD45^{+}$ compartment.

Gating strategy

Cells were initially gated by forward- and side-scatter to exclude debris, followed by singlet selection using FSC-A versus FSC-H. Viable cells were defined as DRAQ7-negative. Viable singlets were separated into $CD31^{-}CD45^{-}$ hepatocyte-enriched, $CD31^{+}CD45^{-}$ endothelial/LSEC-enriched and $CD45^{+}$ immune-cell populations; Kupffer cells were defined as $F4/80^{+}$ cells within the $CD45^{+}$ compartment. EGFP-positive gates were defined using PBS-treated mTmG samples as negative controls and were applied consistently across treatment groups. EGFP positivity reports successful Cre mRNA expression and reporter recombination rather than nanoparticle uptake alone.

☒ Tick this box to confirm that a figure exemplifying the gating strategy is provided in the Supplementary Information.
