# Supplementary material for: Interpretable AI-guided discovery of degradable ionizable lipids with enhanced hepatic mRNA delivery in vivo
Source: Nat Commun. 2026 Oct 12;17:11561. doi: 10.1038/s41467-026-78635-1 (PMC13748640; doi:10.1038/s41467-026-78635-1)
Supplement: Supplementary file 4 [file 41467_2026_78635_MOESM6_ESM.pdf]

nature portfolio

Peer Review File

Interpretable AI-guided discovery of degradable ionizable lipids with enhanced hepatic mRNA delivery in vivo

Corresponding Author: Professor Hao Yan

This file contains all reviewer reports in order by version, followed by all author rebuttals in order by version.

Version 0:

Reviewer comments:

Reviewer #1

(Remarks to the Author)
Lipid nanoparticles (LNPs) have been widely used for mRNA delivery, yet still requiring more efficient ionizable lipids for applications. For this purpose, AI can speed up the development of new ionizable lipids and LNPs. In this manuscript, the authors established an interpretable discovery framework that combined lipid chemistry, phenotyping, machine learning, library expansion, and in vivo validation to define a learnable ionizable-lipid design space. As a result, optimal LNPs outperformed SM-102 LNPs. However, several comments should be addressed before consideration of publication.
Detailed comments:
1. From workflow of Fig. 1a, the authors conducted in vitro screening in step II. Then step V resulted in some candidates and in vitro and in vivo evaluations were further performed. We can see that only some lipids were validated by in vivo assay, and numerous lipids were tested in vitro. Can in vitro screening adequately predict in vivo performance?
2. The authors optimized representative candidate from AI, and some demonstrated hepatocyte-tropism. Can authors predict hepatocyte-targeted lipid structures by AI?
3. The authors stated that "For polyamines containing multiple reactive centers, the equivalents of Traut's reagent and acrylate were scaled proportionally to the number of primary amines". However, acrylates can also react with secondary amines with high activity. How to exclude isomerides?
4. Full names should be supplied at the first appearance of abbreviations, including Materials and Methods section.

(Remarks on code availability)

Reviewer #2

(Remarks to the Author)
This study develops a class of biodegradable ionizable lipids and combines high-throughput experimental screening with machine-learning-guided virtual screening to identify efficient LNP delivery vehicles. The authors first constructed and tested an AMD lipid library using Fluc mRNA expression in HeLa cells as the in vitro delivery readout. These experimental data were then used to train machine learning models, which were further applied to a larger virtual PAMD lipid library to prioritize potentially high-performing candidates. Through subsequent synthesis and in vivo validation, H97T101-LNP exhibited strong liver/hepatocyte-biased delivery in mice. Although this study presents notable advances in the design of biodegradable ionizable lipids and their application in LNP delivery, several conceptual and methodological issues, particularly related to the machine learning analysis, require further clarification and improvement.
1. The ML models were trained based on Fluc expression in HeLa cells, which cannot directly predict or mechanistically explain in vivo hepatocyte-biased delivery without additional evidence linking the in vitro readout to the in vivo phenotype. Accordingly, the SHAP analysis derived from this XGBoost model should be interpreted as identifying structural features associated with HeLa-cell transfection efficiency, rather than mechanistic or structural determinants of in vivo hepatocyte-biased delivery. In its current form, the manuscript does not fully support the claim of "Interpretable AI-guided discovery of hepatocyte-biased ionizable lipids for functional mRNA delivery in vivo"
2. The machine learning section still lacks several key methodological and reproducibility details.
1) The in vitro screening results were divided into three categories: non-hits, acceptable hits, and high-performing hits. However, it is unclear how these labels were used in the machine learning models. Was the ML task formulated as binary

classification or three-class classification? Which samples were assigned to each class, and what were the sample sizes for each category? These details should be provided.
2) The RDKit physicochemical descriptors and Morgan fingerprints were used, but the manuscript does not provide sufficient computational details. For example, the authors should specify the radius of the Morgan fingerprints, whether low-variance features were removed, and whether feature standardization or normalization was performed, etc.
3) In Fig. 3d, how the Head descriptor and Tail descriptor shown in Fig. 3d were generated was not clarified clearly. Were these features calculated separately for the amine head group and the degradable tail? and how these features were subsequently processed and integrated. In addition, given the potential interaction effects between the head group and tail, the authors are encouraged to include an analysis of head-tail interactions, or at least discuss whether the current models can capture such interactions.
4) It mentioned that the models were trained with cross-validation, but the specific data-splitting strategy is not sufficiently described. Moreover, because the AMD/PAMD lipid libraries have an apparent combinatorial matrix structure, random splitting may allow the training and test sets to share similar head groups or tails, leading to structural leakage and overestimation of model performance. The authors are encouraged to include methods like scaffold split or head-/tail-grouped validation to better assess model generalizability to new chemical space.
5) The authors should explain the rationale for applying different preprocessing workflows to different models. For example, why was PCA applied only to RF and LR, but not to XGBoost or LightGBM? Although model-specific preprocessing strategies may be reasonable, using different feature representations could confound the comparison of algorithm performance.

(Remarks on code availability)

Version 1:

Reviewer comments:

Reviewer #1

(Remarks to the Author)
The authors have addressed my concerns. I recommend accepting this manuscript.

(Remarks on code availability)

Reviewer #3

(Remarks to the Author)
While I reviewed the entire manuscript, my comments below are mostly focused on author's responses to Reviewer 2's comments as requested by the editor.
• General Comment
There is a tendency by the authors, in the Discussion section, to frame this study as establishing a framework for the development of hepatocyte-focused ionizable lipids. For example, the sentence in page 9, lines 27-30 still emphasizes "hepatocyte-focused framework". However, the authors appear more cautious about this claim later in the discussion presumable in response to other reviewer's comments. Importantly, only one (or two?) of the lipids demonstrated hepatocyte-enriched delivery, and the in vitro data used to develop the model were not generated using hepatocyte-based systems. Given these limitations, I suggest that the authors emphasize hepatic delivery (in the topic and body of the manuscript) rather than hepatocyte-biased delivery, as the current data do not appear sufficient to support broader conclusions regarding hepatocyte-specific delivery.
• Response to comment 1 from Reviewer 2.
While the author's response to this comment is adequate, implementing the suggestion in the General comment above would ensure they completely address this comment.
• Response to comment 2 from Reviewer 2.
In my opinion, the authors' responses to the comments concerning the methodology used for model development were satisfactory and adequately addressed the issues raised. Individual comments are addressed below.
The author's responses to comments 2.1 were adequate.
The author's responses to comments 2.2 were adequate.
The author's responses to comments 2.3 were adequate.
The author's responses to comments 2.4 were adequate.
The author's responses to comments 2.5 were adequate.

(Remarks on code availability)

Open Access This Peer Review File is licensed under a Creative Commons Attribution 4.0 International License, which permits use, sharing, adaptation, distribution and reproduction in any medium or format, as long as you give appropriate credit to the original author(s) and the source, provide a link to the Creative Commons license, and indicate if changes were made.
In cases where reviewers are anonymous, credit should be given to 'Anonymous Referee' and the source.
The images or other third party material in this Peer Review File are included in the article's Creative Commons license, unless indicated otherwise in a credit line to the material. If material is not included in the article's Creative Commons license and your intended use is not permitted by statutory regulation or exceeds the permitted use, you will need to obtain permission directly from the copyright holder.
To view a copy of this license, visit https://creativecommons.org/licenses/by/4.0/

Responses to reviewers

Reviewer #1

Lipid nanoparticles (LNPs) have been widely used for mRNA delivery, yet still requiring more efficient ionizable lipids for applications. For this purpose, AI can speed up the development of new ionizable lipids and LNPs. In this manuscript, the authors established an interpretable discovery framework that combined lipid chemistry, phenotyping, machine learning, library expansion, and in vivo validation to define a learnable ionizable-lipid design space. As a result, optimal LNPs outperformed SM-102 LNPs. However, several comments should be addressed before consideration of publication.

Response: We thank the reviewer for the positive evaluation of our integrated ionizable-lipid discovery framework and for recognizing the value of combining modular lipid chemistry, high-throughput phenotyping, machine learning, virtual-library expansion, and in vivo validation. In response to the reviewer's constructive comments, we performed additional experiments and machine-learning analyses and revised the manuscript and Supplementary Information to further strengthen the rigor, clarity, and reproducibility of the study. The responses are shown in **blue text**, and the corresponding revisions in the manuscript and Supplementary Information are highlighted **in yellow**.

Comments 1: *From workflow of Fig. 1a, the authors conducted in vitro screening in step II. Then step V resulted in some candidates and in vitro and in vivo evaluations were further performed. We can see that only some lipids were validated by in vivo assay, and numerous lipids were tested in vitro. Can in vitro screening adequately predict in vivo performance?*

Response:

We thank the reviewer for raising this important question. An *in vitro* screen conducted in a single cell model cannot, by itself, adequately predict the complete in vivo performance of an mRNA–LNP formulation. The HeLa-cell firefly luciferase (Fluc) assay measures a composite cellular phenotype involving uptake, intracellular trafficking, cytosolic release and mRNA translation. Systemic performance is additionally influenced by circulation stability, biomolecular interactions, immune clearance, tissue exposure, anatomical barriers and cell-type-specific processing. We therefore use the in vitro screen and machine-learning model as a scalable **candidate-prioritization step**, rather than as a substitute for *in vivo* evaluation.

To examine how *in vitro*-active candidates performed in vivo, we expanded the analysis to five additional candidates selected from the active region of the HeLa-cell screening landscape: A19MD10, A22MD10, A36MD09, PA23MD21 and PA93MD101. Following matched intravenous administration of Fluc mRNA, these formulations exhibited distinct whole-body, hepatic and extrahepatic functional-expression profiles (revised Fig. 5c, 5d and Supplementary Figs. S32–S34). A19MD10 and A22MD10 retained predominantly hepatic but comparatively lower expression, PA23MD21 produced appreciable hepatic expression, whereas A36MD09 and PA93MD101 showed weaker hepatic output or relatively greater extrahepatic expression. Thus, favourable *in vitro* activity did not translate uniformly into either the magnitude or anatomical distribution of functional mRNA expression *in vivo*.

These findings define both the value and limitation of the in vitro screen. It reduces the chemical search space and prioritizes candidates with cell-intrinsic functional-delivery competence, but it does not independently predict systemic expression, organ-resolved functional output or cell-type preference. We have therefore revised Fig. 1a to present a two-stage workflow comprising ***in vitro* phenotyping and ML-based candidate prioritization**, followed by **independent *in vivo* phenotyping and lead identification**. Cell-type-resolved *in vivo* analyses were also expanded in revised Fig. 5j and are discussed separately in our response to Comment 2.

Revision of the manuscript and Supplementary Information:

We revised the Title, Abstract, Introduction and Results to define the workflow as ML-based prioritization using the HeLa-cell phenotype followed by independent in vivo evaluation (Manuscript, page 1, lines 1–2 and line 21 to page 2, line 4; page 3, lines 4–23; page 7, lines 18–33 and page 8, line 1). Fig. 1a was revised accordingly, and Fig. 5c, 5d was expanded to compare whole-body and hepatic Fluc expression across additional candidates (Manuscript, pages 18–20 and 24–25). Supplementary Figs. S32–S34 provide the corresponding whole-body, hepatic and organ-resolved functional-expression data (Supplementary Information, pages 36–38).

Here, we developed a two-stage machine-learning-guided framework for the discovery of degradable ionizable lipids. We integrated a 520-member Amine Modular Degradable (AMD) lipid library, standardized high-throughput phenotyping and interpretable machine learning to prioritize candidates from a virtual space of more than 10,000 lipids. Independent in vivo evaluation subsequently identified lipids with enhanced hepatic functional output and hepatocyte-enriched expression across multiple mRNA applications. This study establishes a data-efficient workflow linking modular lipid chemistry and interpretable modelling with independent in vivo functional validation. (Manuscript, page 3, lines 4–10)

We then quantified functional translation using Fluc mRNA. At a matched dose, PA97MD101 produced ~4-fold higher hepatic Fluc output than SM102 despite broadly comparable liver accumulation (Fig. 5c–e; Figs. S32–S34). Expanded analysis of A19MD10, A22MD10, A36MD09, PA23MD21 and PA93MD101 revealed distinct whole-body, hepatic and extrahepatic functional-expression profiles. Thus, favourable HeLa-cell activity did not translate uniformly into either the magnitude or anatomical distribution of functional mRNA expression in vivo, supporting use of the model for candidate prioritization rather than prediction of systemic performance. (Manuscript, page 7, lines 18–24)

To apply a more stringent genome-level readout, we used the Cre/LoxP-based mTmG reporter system, in which successful Cre mRNA delivery and expression induce permanent tdTomato-to-EGFP conversion (Fig. 5f; Fig. S35). PA97MD101 produced stronger liver-level recombination than SM102, supported by fluorescence imaging, confocal microscopy and LoxP-specific qPCR (Fig. 5g-h; Figs. S36–S40). Flow cytometry showed distinct EGFP-activation profiles across the $CD31^{-}CD45^{-}$ hepatocyte-enriched, $CD31^{+}CD45^{-}$ endothelial/LSEC-enriched and $CD45^{+}$ immune-cell compartments (Fig. 5i-j; Figs. S41–S47). PA97MD101 showed greater functional expression in the hepatocyte-enriched population and lower expression in non-parenchymal populations than SM102. EGFP activation integrates delivery, translation and recombination and should not be interpreted as a direct measurement of nanoparticle uptake or receptor-specific targeting. (Manuscript, page 7, lines 25–33 and page 8, line 1)

Fig. 1. Two-stage AI-guided discovery and independent in vivo validation of degradable ionizable lipids. (a) Discovery workflow. The 520-member AMD library was screened using Fluc mRNA expression in HeLa cells, and the resulting *in vitro* labels were used for machine-learning-based prioritization within the 10,920-member PAMD virtual space. Selected candidates were subsequently synthesized and evaluated independently *in vivo* for organ-level and cell-population-resolved functional expression. **(b)** Synthetic and formulation strategy for AMD lipids and LNP preparation by microfluidic mixing. **(c)** *In vitro* Fluc mRNA transfection activity of the 520-member AMD library in HeLa cells. Lipids were descriptively categorized as non-hits ($<10^3$ RLU; n = 11), acceptable hits (10^3–10^5 RLU; n = 422), or high-performing hits ($>10^5$ RLU; n = 87). These categories were used for visualization only; model training used binary labels defined at 2,000 RLU. (Manuscript, page 18-19, Fig. 1 a-c)

New and revised figures:

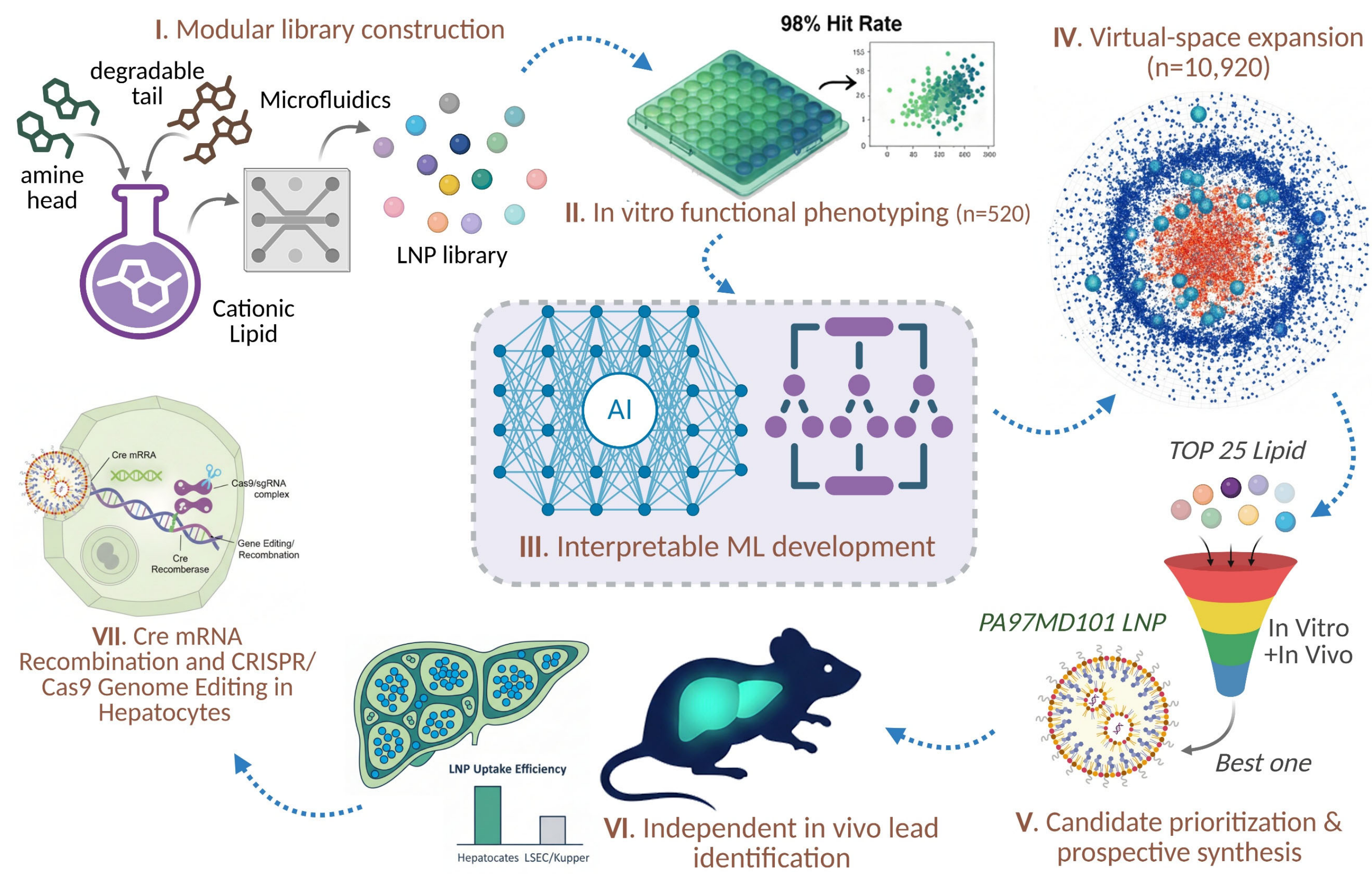

Revised Fig. 1a. Two-stage discovery workflow separating HeLa-cell phenotyping and ML-based candidate prioritization from independent in vivo evaluation. The model was not trained on organ- or cell-type-resolved outcomes.

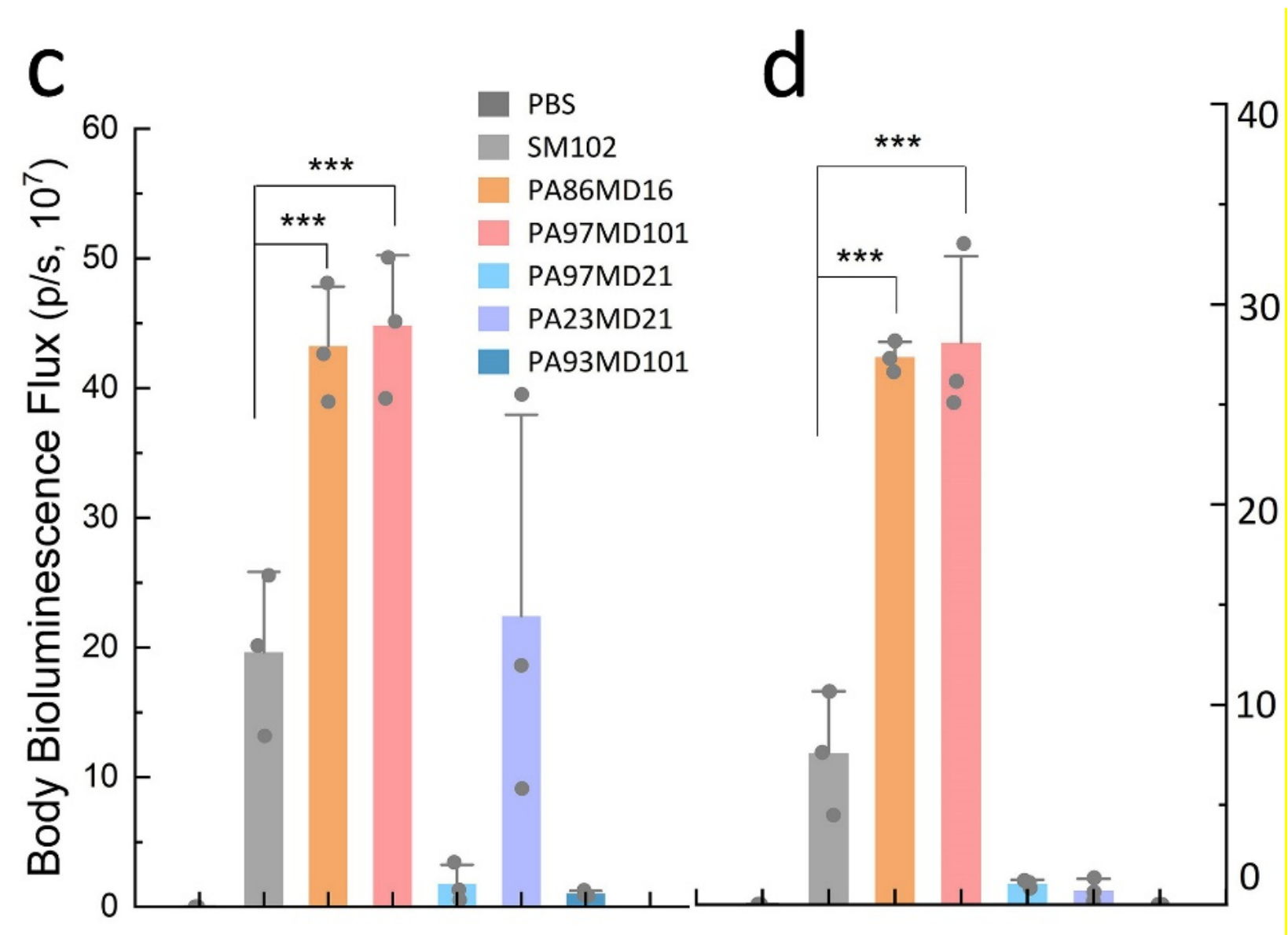

Revised Fig. 5c, d. Whole-body and ex vivo liver Fluc expression 6 h after intravenous administration of the indicated mRNA–LNPs. Data are mean ± s.d.; n = 3 biologically independent mice.

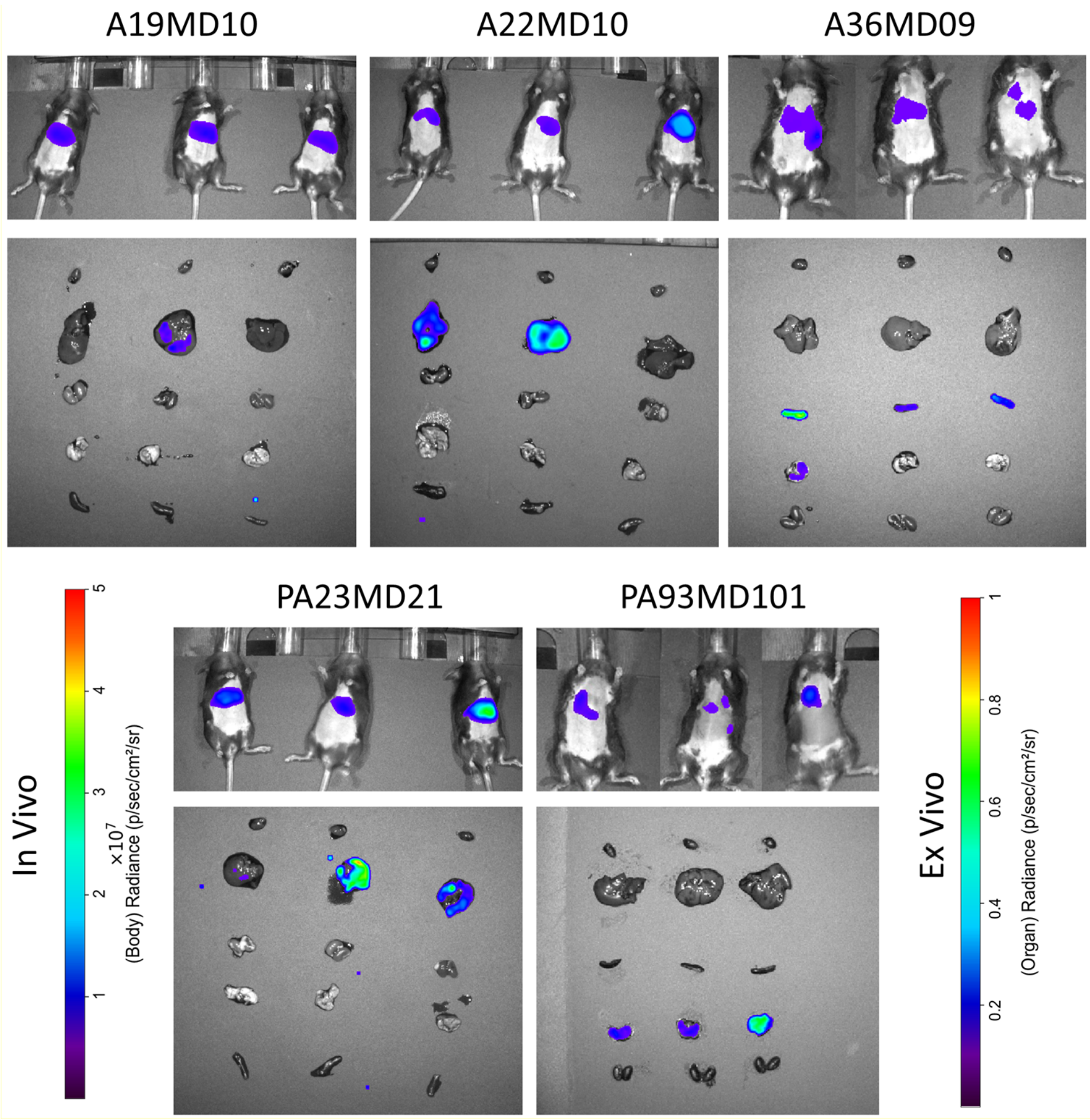

Figure S32. Representative whole-body and organ-level Fluc bioluminescence from five additional candidate LNPs 6 h after intravenous administration. Bioluminescence reports productive mRNA expression rather than nanoparticle mass distribution.

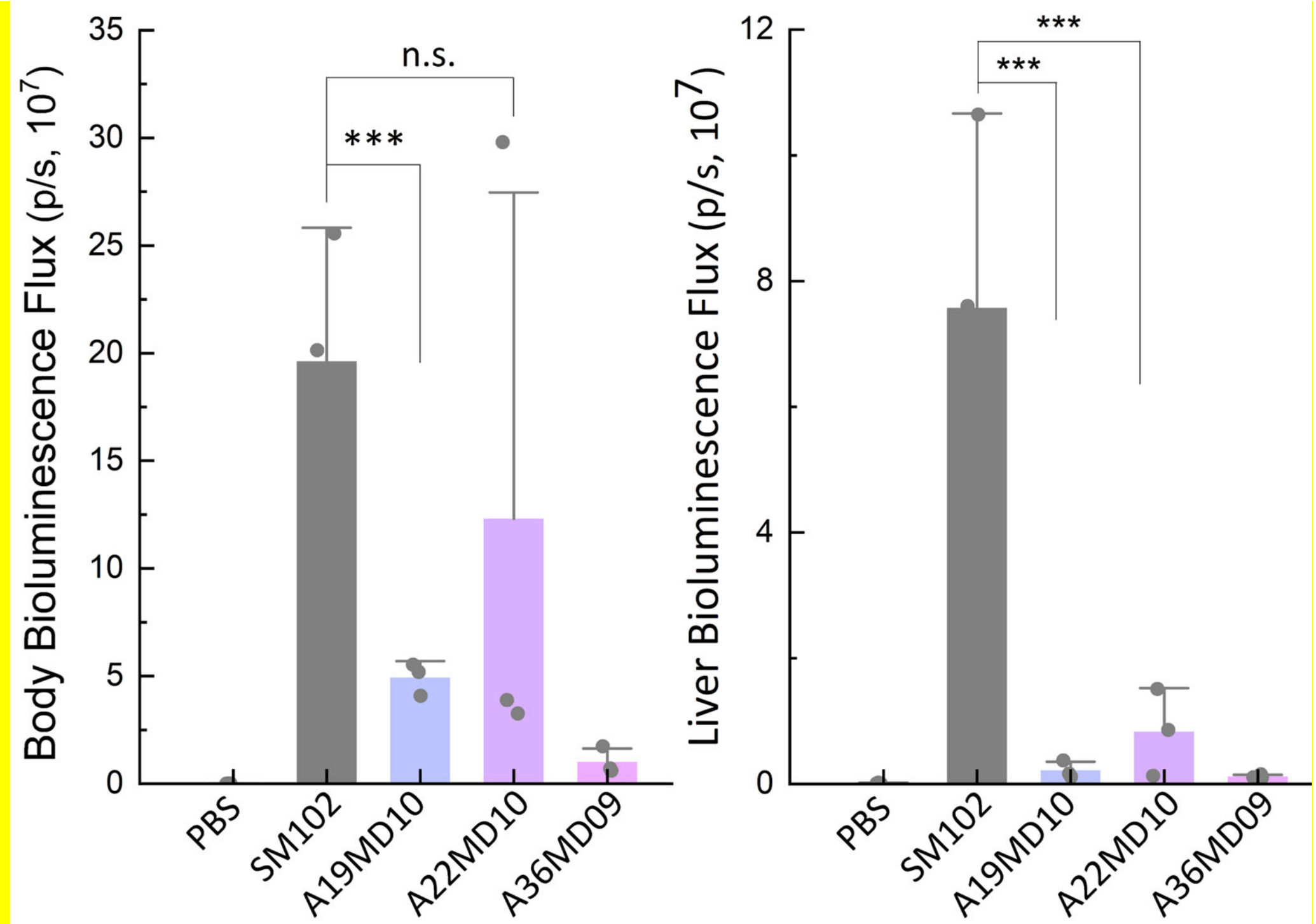

Figure S33. Whole-body and hepatic Fluc expression mediated by A19MD10, A22MD10 and A36MD09 LNPs, with PBS and SM102 as controls. Data are mean ± s.d.; n = 3 biologically independent mice; n.s., not significant; ***$P < 0.001$.

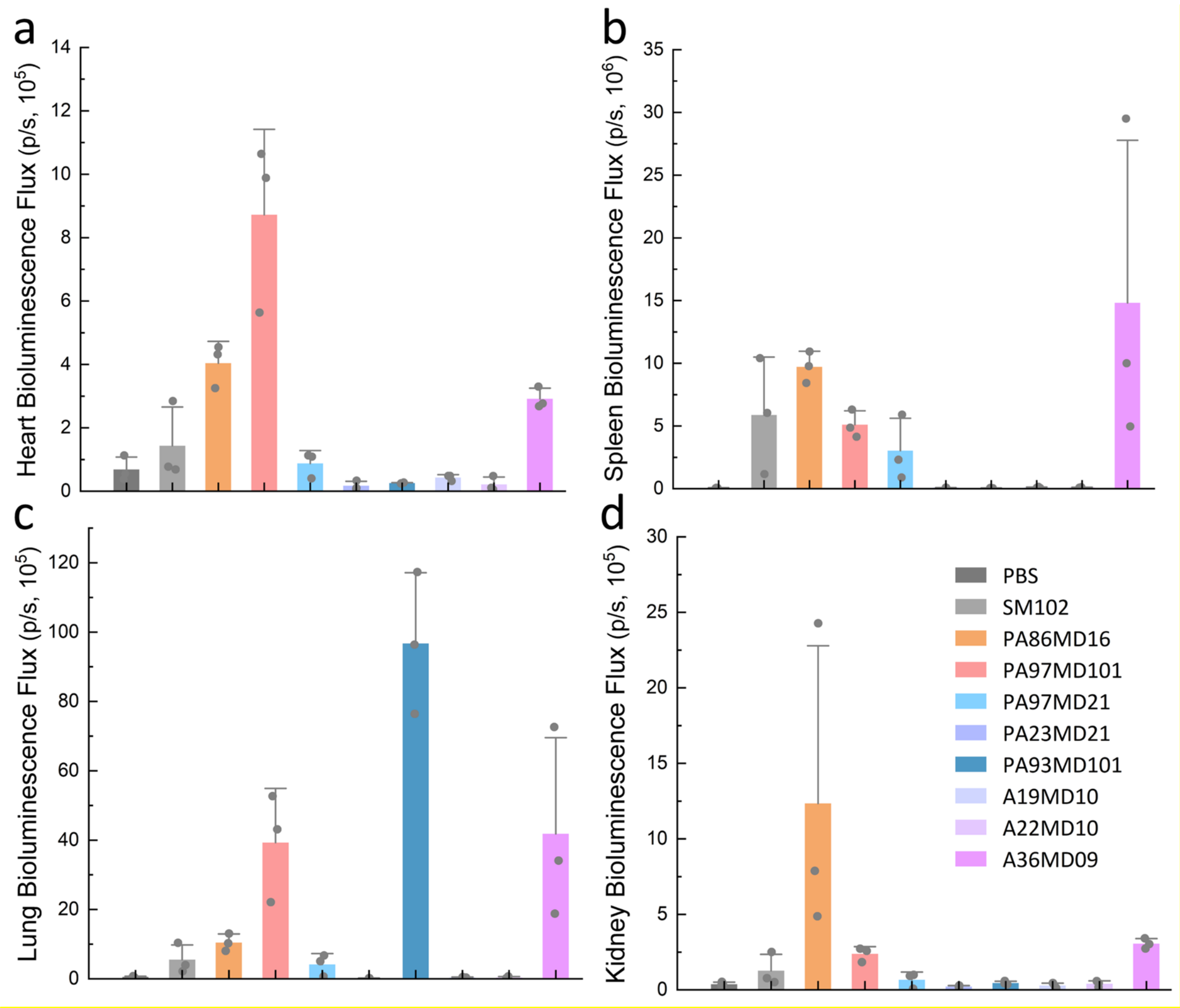

Figure S34. Organ-resolved Fluc expression in the heart, spleen, lungs and kidneys after systemic administration of the indicated candidate LNPs. Data are mean ± s.d.; n = 3 biologically independent mice.

***Comments 2**: The authors optimized representative candidate from AI, and some demonstrated hepatocyte-tropism. Can authors predict hepatocyte-targeted lipid structures by AI?*

Response:

We thank the reviewer for raising this important question. The current machine-learning model cannot, by itself, reliably predict hepatocyte-targeted lipid structures. The model was trained exclusively on firefly luciferase expression in HeLa cells and therefore predicts an *in vitro* transfection phenotype rather than organ distribution or intrahepatic cell-type preference. Hepatocyte-enriched delivery is additionally influenced by formulation properties, circulation behaviour, biomolecular interactions, liver sinusoidal transport, immune-cell clearance and cell-specific uptake and intracellular processing.

To determine intrahepatic cell-type preference experimentally, we expanded Cre mRNA delivery in mTmG reporter mice to three additional candidates, PA23MD21, A19MD10 and A22MD10, and evaluated them together with PA86MD16, PA97MD101 and SM102. Functional delivery was quantified as the percentage of EGFP-positive cells in $CD31^+CD45^-$ liver sinusoidal endothelial cells, the $CD31^-CD45^-$ hepatocyte-enriched population, and $CD45^+$ immune-cell populations (revised Fig. 5j and Supplementary Figs. S41–S47). The tested LNPs exhibited distinct intrahepatic functional-expression profiles. In particular, PA97MD101 produced greater EGFP expression in the hepatocyte-enriched population and lower expression in non-parenchymal populations than SM102, whereas other candidates showed different cell-type distributions. These results demonstrate that hepatocyte-enriched functional delivery was identified by independent *in vivo* phenotyping rather than inferred directly from the HeLa-cell machine-learning score.

The currently available cell-type-resolved dataset remains too small to train and validate a dedicated model

for hepatocyte preference with reliable generalizability. We therefore position the present model as a tool for prioritizing candidates with higher predicted *in vitro* activity, while organ distribution and intrahepatic cell-type preference are established experimentally. A future hepatocyte-targeting model will require a substantially larger dataset integrating lipid structures, formulation properties, organ-resolved expression and cell-type-specific *in vivo* functional labels.

Revision of the manuscript and Supplementary Information:

We revised the Results and Discussion to state that the model prioritizes candidates according to the HeLa-cell phenotype and does not directly predict intrahepatic cell-type preference (Manuscript, page 5, line 26 to page 6, line 8; page 7, line 18 to page 8, line 1; page 10, lines 7–15). We expanded Fig. 5j and Supplementary Figs. S45–S47 to show Cre-dependent functional expression across marker-defined liver-cell populations for additional candidate LNPs (Manuscript, pages 24–25; Supplementary Information, pages 48–50).

SHapley Additive exPlanations (SHAP) analysis identified descriptors associated with the HeLa-cell transfection phenotype. Head and Tail descriptors were calculated separately and assigned to their respective modules using feature prefixes (Fig. 3d). Complementary interpretability analyses are provided in Figs. S8–S12. Module-resolved rankings highlighted descriptors related to charge distribution, lipophilicity and topological connectivity (Fig. S13), while correlation-network analysis revealed related physicochemical clusters and partial redundancy among model inputs (Fig. S14). Together, these results suggest that in vitro activity reflects coordinated physicochemical properties rather than a single dominant feature. These associations are structural correlates of the in vitro phenotype; organ distribution and hepatocyte-enriched functional expression were evaluated independently in vivo. (Manuscript, page 5, lines 26–33)

We then quantified functional translation using Fluc mRNA. At a matched dose, PA97MD101 produced ~4-fold higher hepatic Fluc output than SM102 despite broadly comparable liver accumulation (Fig. 5c–e; Figs. S32–S34). Expanded analysis of A19MD10, A22MD10, A36MD09, PA23MD21 and PA93MD101 revealed distinct whole-body, hepatic and extrahepatic functional-expression profiles. Thus, favourable HeLa-cell activity did not translate uniformly into either the magnitude or anatomical distribution of functional mRNA expression in vivo, supporting use of the model for candidate prioritization rather than prediction of systemic performance.

To apply a more stringent genome-level readout, we used the Cre/LoxP-based mTmG reporter system, in which successful Cre mRNA delivery and expression induce permanent tdTomato-to-EGFP conversion (Fig. 5f; Fig. S35). PA97MD101 produced stronger liver-level recombination than SM102, supported by fluorescence imaging, confocal microscopy and LoxP-specific qPCR (Fig. 5g-h; Figs. S36–S40). Flow cytometry showed distinct EGFP-activation profiles across the $CD31^{-}CD45^{-}$ hepatocyte-enriched, $CD31^{+}CD45^{-}$ endothelial/LSEC-enriched and $CD45^{+}$ immune-cell compartments (Fig. 5i-j; Figs. S41–S47). PA97MD101 showed greater functional expression in the hepatocyte-enriched population and lower expression in non-parenchymal populations than SM102. EGFP activation integrates delivery, translation and recombination and should not be interpreted as a direct measurement of nanoparticle uptake or receptor-specific targeting. (Manuscript, page 7, lines 18–33)

New and revised figures:

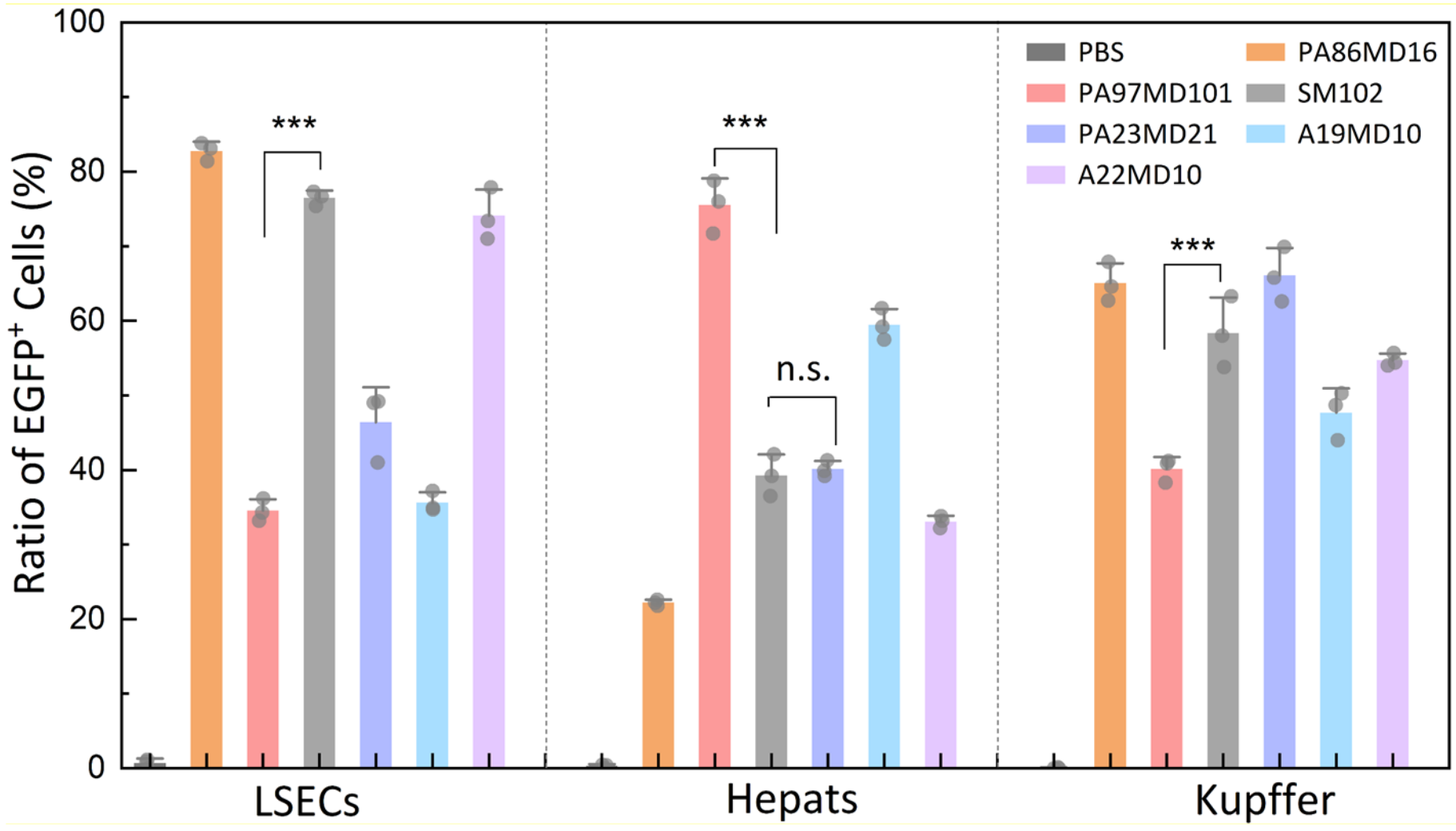

Revised Fig. 5j. EGFP activation within CD31⁻CD45⁻ hepatocyte-enriched, CD31⁺CD45⁻ endothelial/LSEC-enriched and CD45⁺ immune-cell populations after Cre mRNA–LNP administration. Data are mean ± s.d.; n = 3 biologically independent mice.

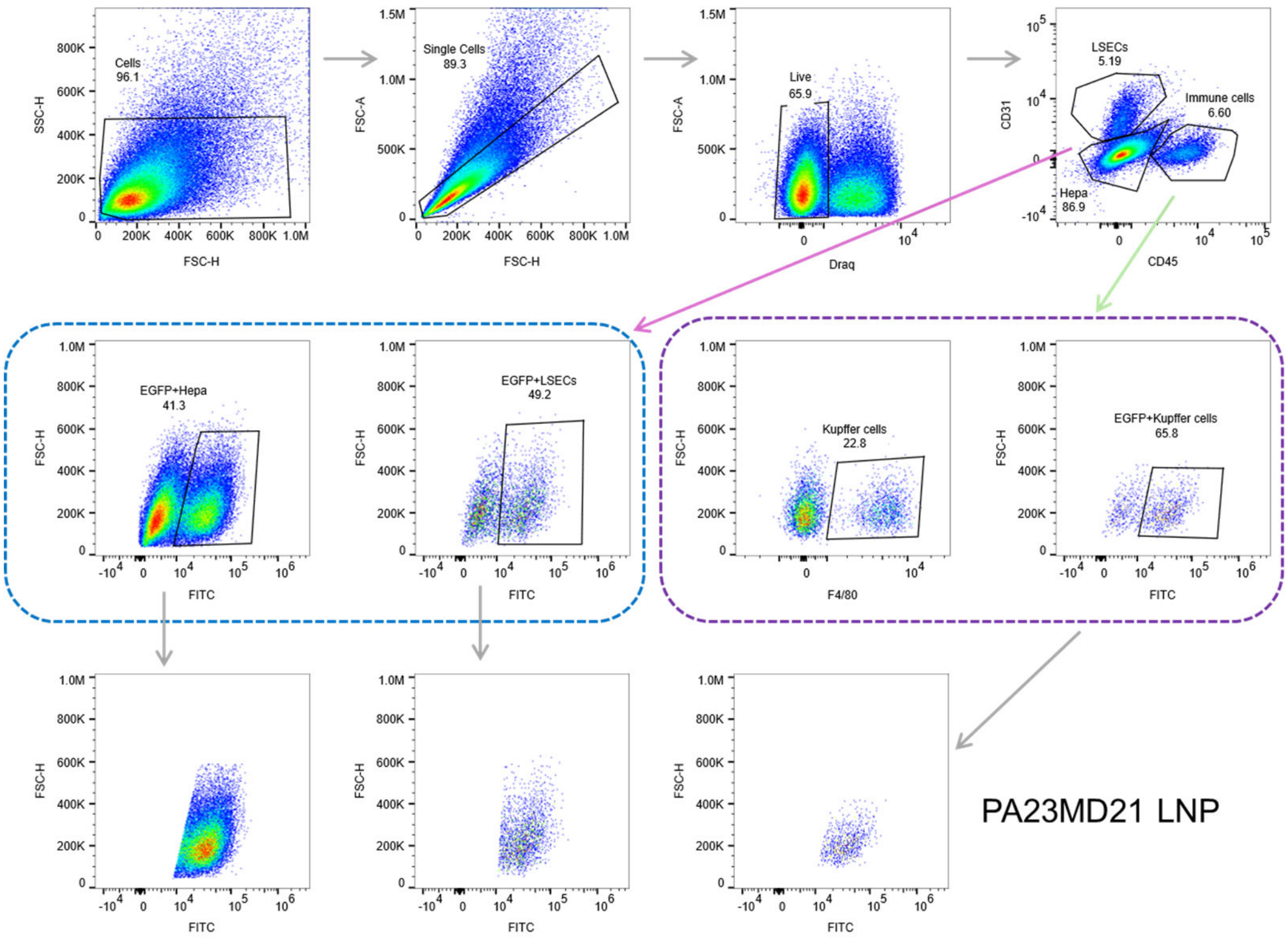

Figure S45. Representative flow-cytometry analysis of marker-defined liver-cell compartments after PA23MD21–Cre mRNA LNP administration.

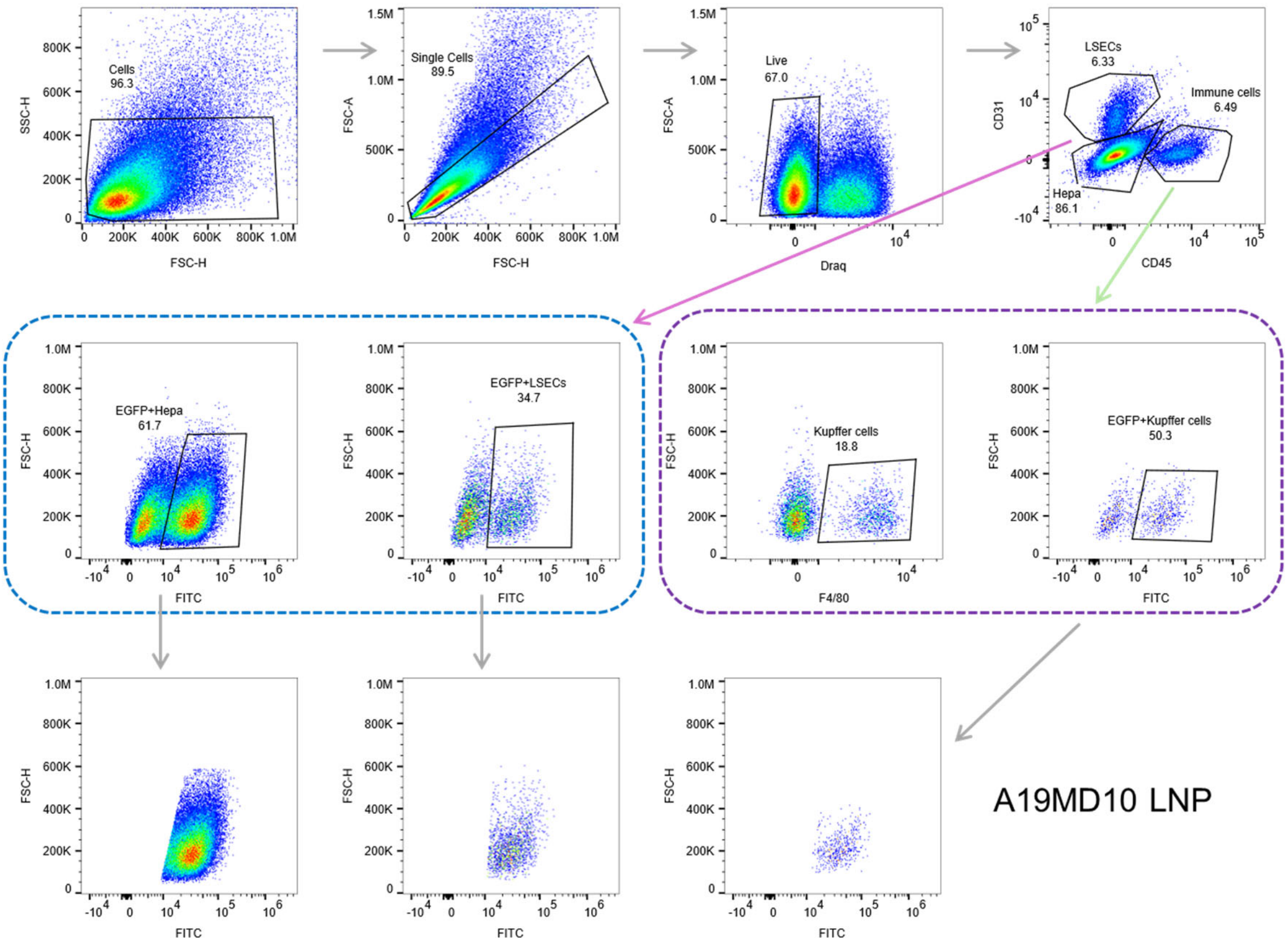

Figure S46. Representative flow-cytometry analysis of marker-defined liver-cell compartments after A19MD10–Cre mRNA LNP administration.

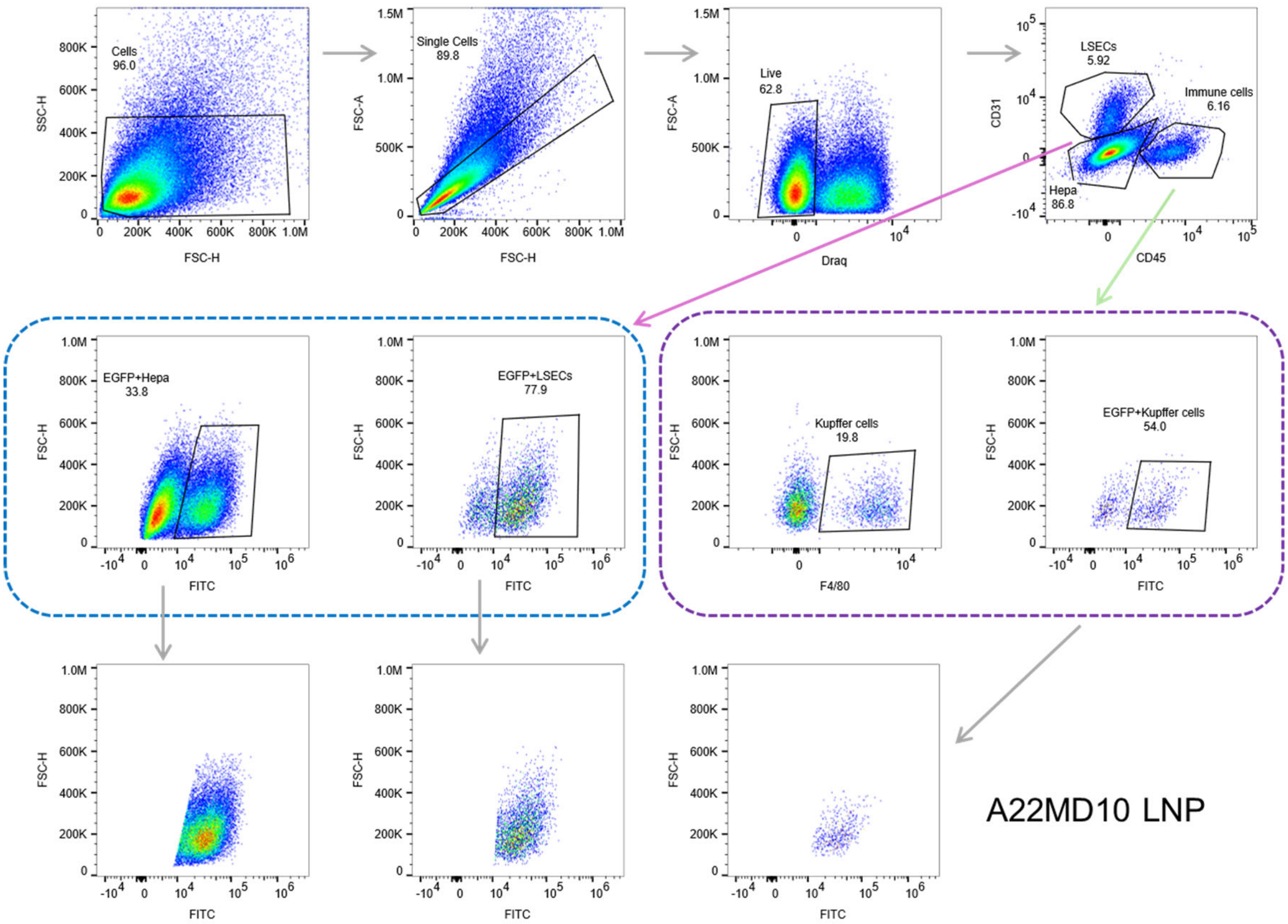

Figure S47. Representative flow-cytometry analysis of marker-defined liver-cell compartments after A22MD10–Cre mRNA LNP administration.

Comments 3: *The authors stated that "For polyamines containing multiple reactive centers, the equivalents of Traut's reagent and acrylate were scaled proportionally to the number of primary amines". However, acrylates can also react with secondary amines with high activity. How to exclude isomerides?*

Response:

We thank the reviewer for raising this important concern regarding reaction selectivity. We agree that acrylates can undergo N-Michael addition with free secondary amines and that this competing pathway must be considered for polyamine headgroups.

In our synthetic route, primary amines first react with Traut's reagent through ring opening to generate amidine-linked intermediates bearing terminal thiols (*Chem. Soc. Rev.* 2015, 44, 5495–5551.). The resulting thiols are highly nucleophilic and generally undergo rapid S-Michael addition with acrylates under mild conditions (*Chem. Mater.* 2014, 26, 724–744; Macromolecules 2018, 51, 5979–5988.). In comparison, the Traut-derived amidine nitrogens are less nucleophilic because of resonance delocalization and partial protonation. Nevertheless, polyamine headgroups may retain pre-existing free secondary amines that could potentially participate in N-Michael addition. Because chemoselectivity can depend on the local molecular environment and reaction conditions (*Nat. Chem. Biol.* 2011, 7, 876–884; *Chem. Soc. Rev.* 2015, 44, 5495–5551.), we agree that the product assignment should be supported experimentally rather than inferred solely from the proposed reaction mechanism.

We therefore purified and further characterized representative products derived from headgroups containing multiple reactive nitrogen sites, including A19MD10, A22MD10 and PA23MD10. Following column chromatography, these products were obtained in isolated yields of 86.4%, 70.3% and 83.4%, respectively. Their assigned structures were supported by complementary ^{1}H NMR, ^{13}C NMR and high-resolution mass spectrometry analyses. The observed exact masses were consistent with the intended substitution products, and no prominent additional resonances or dominant mass signals attributable to highly abundant over-alkylated products were evident in the acquired spectra.

Together, the reaction preference and spectroscopic data support the assigned products as the predominant purified species under the conditions used. We acknowledge that one-dimensional NMR and direct-infusion HRMS cannot absolutely exclude trace regioisomeric or secondary-amine-derived products. We have therefore avoided claiming complete chemoselectivity and expanded the structural characterization of representative multi-amine-derived and lead lipids in Supplementary Figs. S22–S24.

Revision of the manuscript and Supplementary Information:

We expanded the synthetic Methods to clarify the preparation, purification and structural characterization of representative multi-amine-derived and lead lipids (Manuscript, pages 10–11, particularly page 11, lines 11–12). Supplementary Figs. S22–S24 provide ^{1}H NMR, ^{13}C NMR and HRMS data for the purified representative products and explicitly state that the spectra support the assigned predominant structures but do not exclude trace regioisomeric or secondary-amine-derived species (Supplementary Information, pages 27–29, particularly page 29, lines 15–16).

Representative purified multi-amine-derived products and lead lipids were characterized by ^{1}H NMR, ^{13}C NMR and HRMS. (Manuscript, pages 11, lines 11–12)

New and revised figures:

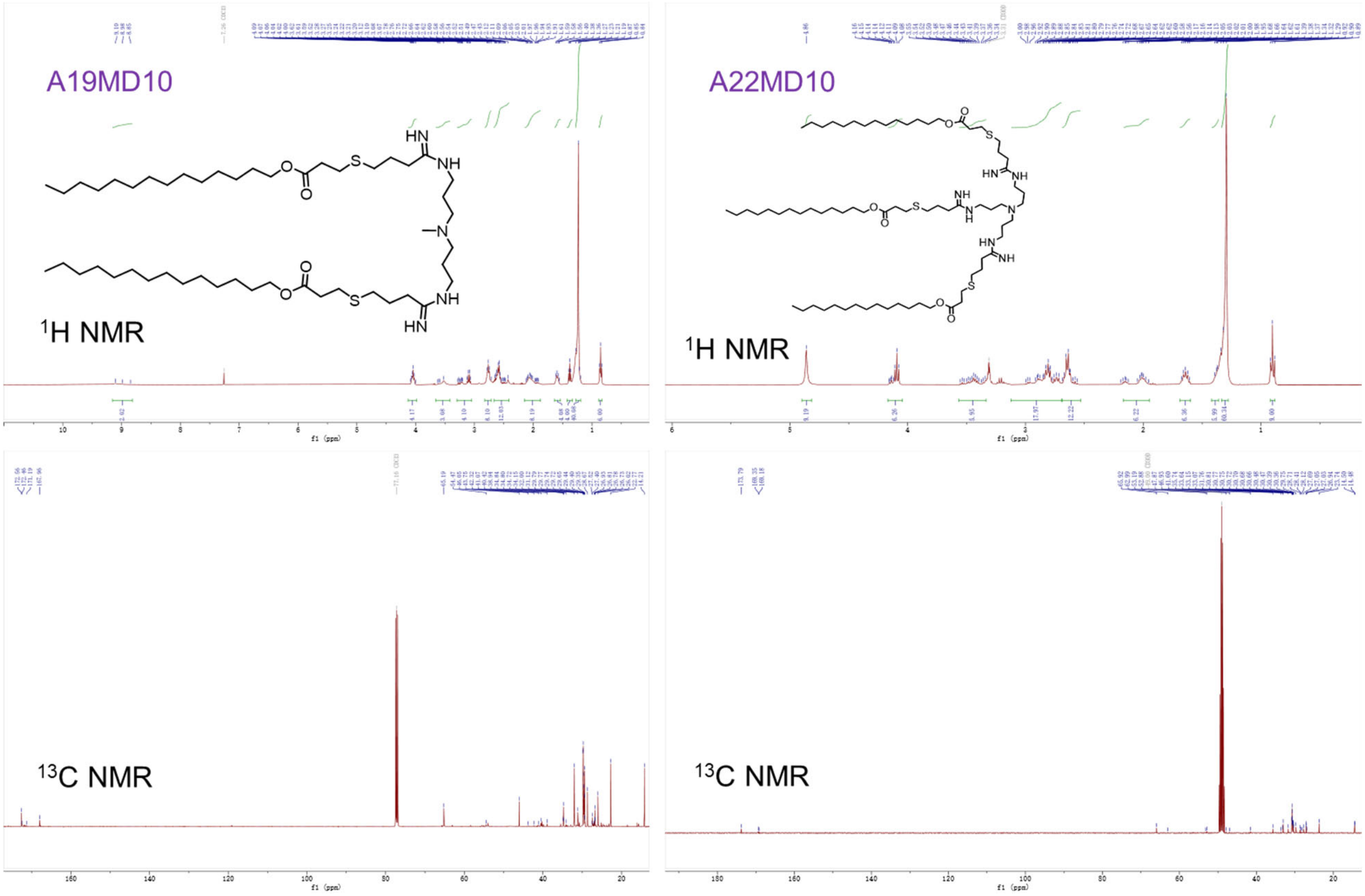

Figure S22. ^{1}H NMR, ^{13}C NMR and HRMS characterization of purified A19MD10 and A22MD10; isolated yields, 86.4% and 70.3%, respectively.

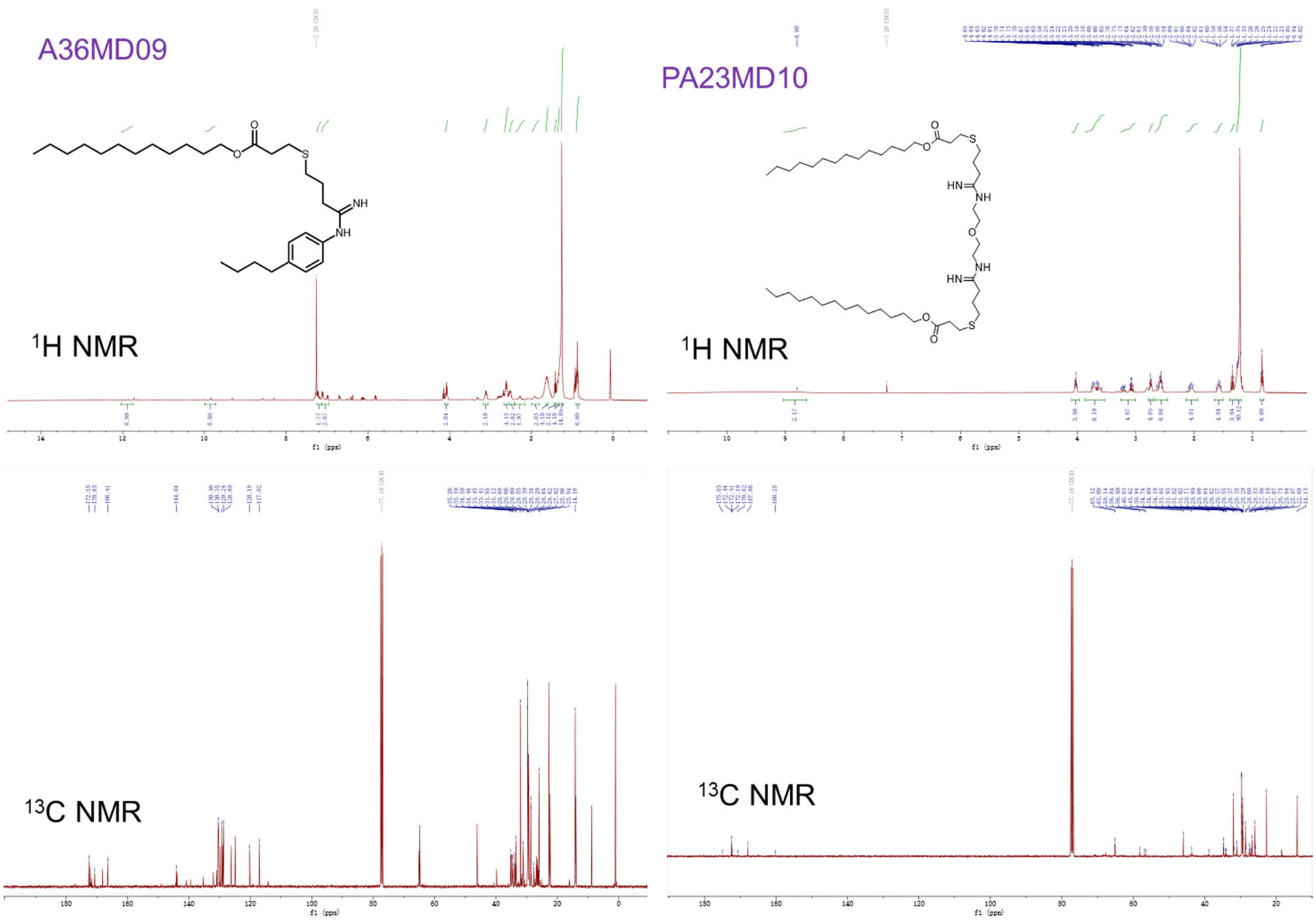

Figure S23. ^{1}H NMR, ^{13}C NMR and HRMS characterization of purified A36MD09 and PA23MD10; PA23MD10 isolated yield, 83.4%.

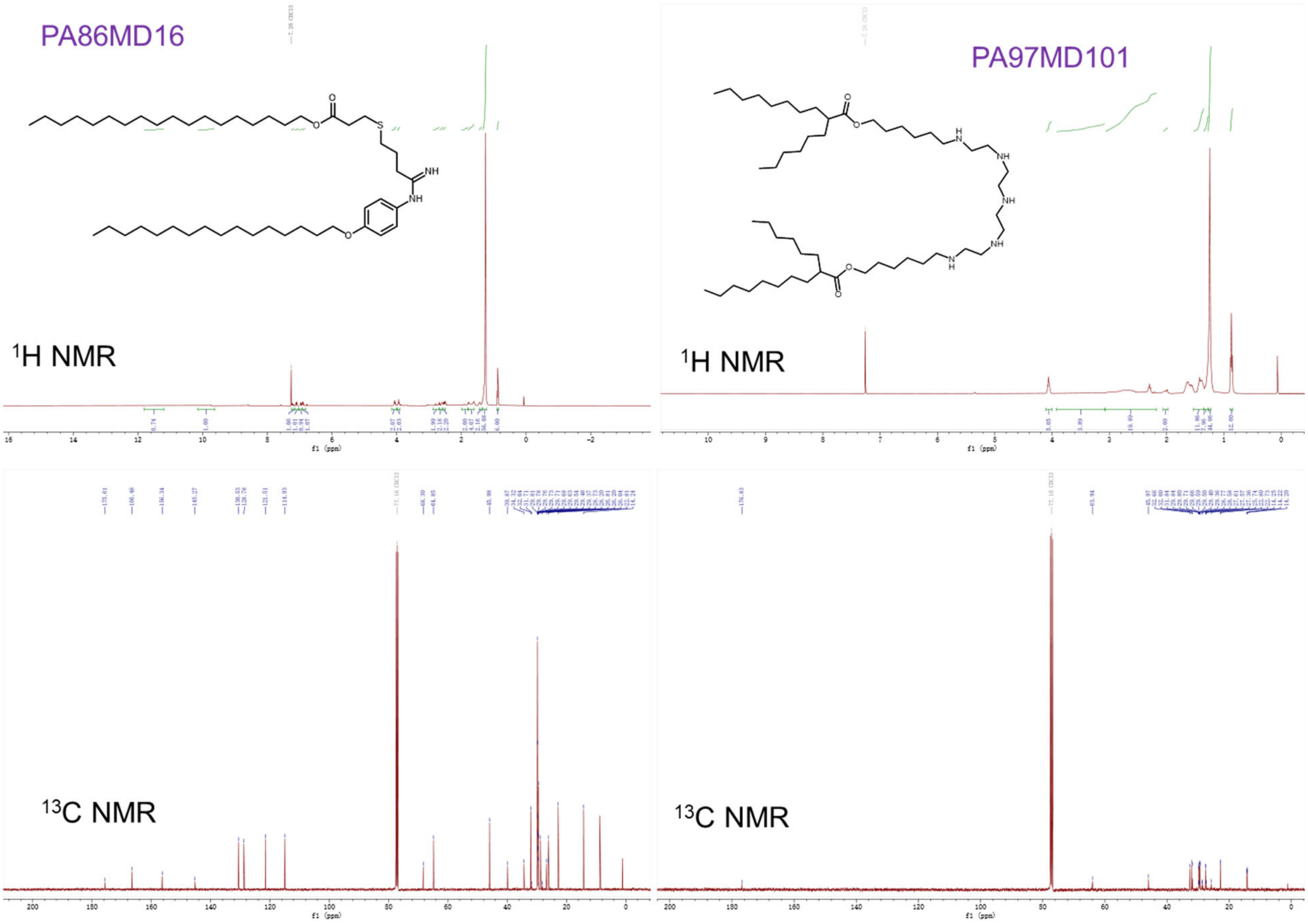

Figure S24. ^{1}H NMR, ^{13}C NMR and HRMS characterization of the purified lead lipids PA86MD16 and PA97MD101.

Comments 4: *Full names should be supplied at the first appearance of abbreviations, including Materials and Methods section.*

Response:

We have systematically reviewed the revised manuscript, Methods, figure legends and Supplementary Information and provided the full term at the first occurrence of each abbreviation. These include, among others, messenger RNA (mRNA), small interfering RNA (siRNA), single-guide RNA (sgRNA), membrane-targeted tdTomato/membrane-targeted enhanced green fluorescent protein (mTmG), enhanced green fluorescent protein (EGFP), SHapley Additive exPlanations (SHAP) and Förster resonance energy transfer (FRET). The full chemical names of abbreviated phospholipids and fluorescent lipids, including DOPS, DOPC, DOPE, NBD-PE and Rho-PE, have also been added in the Supplementary Information.

In addition, we standardized the lipid nomenclature throughout the manuscript, figures and Supplementary Information. Lipids from the experimental AMD library are now consistently designated as **A*x*MD*y***, whereas lipids from the expanded PAMD library are designated as **PA*x*MD*y***, where *x* and *y* identify the amine-head and modular degradable-tail components, respectively. All previous H*x*T*y* and inconsistent AMD-style designations have been replaced accordingly.

Revision of the manuscript and Supplementary Information:

We systematically revised the manuscript, Methods, figure legends and Supplementary Information to define abbreviations at first use and to standardize chemical, assay and lipid nomenclature throughout. Representative revisions include the definitions of mTmG and EGFP in the in vivo Results (Manuscript, page 4, lines 5–13), SHAP in the machine-learning Results (page 5, line 26), and the full chemical names of DOPS, DOPC, DOPE,

NBD-PE and Rho-PE in the Supplementary Methods (Supplementary Information, page 3, lines 19–24). AMD and PAMD lipid identifiers were also standardized throughout the manuscript and Supplementary Information.

Endosome-mimicking anionic liposomes were composed of 1,2-dioleoyl-sn-glycero-3-phospho-L-serine (DOPS) : 1,2-dioleoyl-sn-glycero-3-phosphocholine (DOPC) : 1,2-dioleoyl-sn-glycero-3-phosphoethanolamine (DOPE) : N-(7-nitrobenz-2-oxa-1,3-diazol-4-yl)-phosphoethanolamine (NBD-PE) : rhodamine-phosphoethanolamine (Rho-PE) at a molar ratio of 25:25:48:1:1. Lipids were dissolved in DCM, evaporated to form a thin film, and vacuum-dried for 2 h. Films were hydrated with PBS (pH 7.4) and sonicated for 30 min. Liposomes were concentrated to ~1 mg mL^{-1} using 30 kDa centrifugal filters. (Supplementary Information, pages 3, line 19-22)

Reviewer #2 (Remarks to the Author):

This study develops a class of biodegradable ionizable lipids and combines high-throughput experimental screening with machine-learning-guided virtual screening to identify efficient LNP delivery vehicles. The authors first constructed and tested an AMD lipid library using Fluc mRNA expression in HeLa cells as the in vitro delivery readout. These experimental data were then used to train machine learning models, which were further applied to a larger virtual PAMD lipid library to prioritize potentially high-performing candidates. Through subsequent synthesis and in vivo validation, H97T101-LNP exhibited strong liver/hepatocyte-biased delivery in mice. Although this study presents notable advances in the design of biodegradable ionizable lipids and their application in LNP delivery, several conceptual and methodological issues, particularly related to the machine learning analysis, require further clarification and improvement.

Response: We thank the reviewer for the positive assessment of our biodegradable ionizable-lipid platform and integrated experimental–computational discovery framework. We also appreciate the reviewer's constructive concerns regarding the conceptual framing and methodological rigor of the machine-learning analysis. In response, we performed extensive additional analyses and validation, clarified the prediction task and class definitions, expanded the description of molecular representations and data preprocessing, examined the respective and combined contributions of the head and tail modules, and introduced more stringent grouped and double-cold validation strategies. We have also revised the manuscript to clarify that the model is intended to prioritize candidates based on *in vitro* transfection activity, rather than to directly predict *in vivo* organ or cell-type tropism, which requires independent experimental validation. These revisions substantially strengthen the rigor, transparency and reproducibility of the study. The responses are shown in **blue text**, and the corresponding revisions in the manuscript and Supplementary Information are highlighted **in yellow**.

Comments 1: *The ML models were trained based on Fluc expression in HeLa cells, which cannot directly predict or mechanistically explain in vivo hepatocyte-biased delivery without additional evidence linking the in vitro readout to the in vivo phenotype. Accordingly, the SHAP analysis derived from this XGBoost model should be interpreted as identifying structural features associated with HeLa-cell transfection efficiency, rather than mechanistic or structural determinants of in vivo hepatocyte-biased delivery. In its current form, the manuscript does not fully support the claim of "Interpretable AI-guided discovery of hepatocyte-biased ionizable lipids for functional mRNA delivery in vivo"*

Response:

We agree that the XGBoost model, which was trained exclusively on Fluc mRNA expression in HeLa cells, predicts in vitro functional transfection activity rather than organ distribution or intrahepatic cell-type preference. Neither organ-level nor cell-type-resolved in vivo outcomes were included as training labels. Therefore, the model and its SHAP analysis cannot be interpreted as directly predicting or mechanistically explaining hepatocyte-biased delivery.

We have consequently reframed the study as a two-stage discovery workflow. In the first stage, the model prioritizes candidates from the 10,920-member PAMD virtual library according to their predicted in vitro transfection activity. The molecular features identified by SHAP, including charge distribution, lipophilicity and topological connectivity, are now described strictly as structural correlates of the HeLa-cell transfection phenotype. In the second stage, the prioritized candidates are synthesized and independently evaluated for functional expression, organ distribution and intrahepatic cell-type preference *in vivo*.

To strengthen this independent in vivo evaluation, we expanded the Cre/mTmG flow-cytometry analysis to include three additional candidates, A19MD10, A22MD10 and PA23MD21, alongside PA86MD16, PA97MD101 and SM102. Functional Cre mRNA delivery was assessed in $CD31^{-}CD45^{-}$ hepatocyte-enriched

cells, $CD31^+CD45^-$ liver sinusoidal endothelial cells and $F4/80^+$ Kupffer cells within the $CD45^+$ compartment. The candidates exhibited distinct cell-type-resolved expression profiles: A19MD10 showed relatively greater expression in the hepatocyte-enriched population, whereas A22MD10 and PA23MD21 showed more pronounced expression in LSECs and Kupffer cells, respectively. These divergent profiles reinforce that in vitro activity does not uniquely determine intrahepatic cell-type preference and that the latter must be established experimentally.

Accordingly, we have removed language implying that the model directly learned, predicted or explained hepatocyte tropism. We have revised the title to "Interpretable AI-guided discovery of degradable ionizable lipids with hepatocyte-enriched mRNA delivery in vivo" and made corresponding changes throughout the Abstract, Results, Discussion and SHAP-related descriptions. Our revised conclusion is that machine learning improves the efficiency of candidate prioritization based on an *in vitro* phenotype, whereas *in vivo* expression, organ distribution and cell-type preference require independent experimental validation. Developing a model that directly predicts hepatocyte-enriched delivery will require a substantially larger dataset containing matched lipid structures and cell-type-resolved *in vivo* functional labels.

Revision of the manuscript and Supplementary Information:

We revised the Introduction, Results and Discussion to distinguish in vitro ML-based prioritization from independent in vivo phenotyping and to describe SHAP-derived features only as correlates of the HeLa-cell transfection phenotype (Manuscript, page 3, lines 4–23; page 5, line 26 to page 6, line 8; page 10, lines 7–15). Fig. 1a and Fig. 5j were revised to reflect this two-stage framework (Manuscript, pages 18–20 and 24–25), and Supplementary Note S1 and Supplementary Figs. S41–S47 define the model scope and provide independent cell-population-resolved validation (Supplementary Information, page 10, lines 14–29; pages 44–50).

Here, we developed a two-stage machine-learning-guided framework for the discovery of degradable ionizable lipids. We integrated a 520-member Amine Modular Degradable (AMD) lipid library, standardized high-throughput phenotyping and interpretable machine learning to prioritize candidates from a virtual space of more than 10,000 lipids. Independent in vivo evaluation subsequently identified lipids with enhanced hepatic functional output and hepatocyte-enriched expression across multiple mRNA applications. This study establishes a data-efficient workflow linking modular lipid chemistry and interpretable modelling with independent in vivo functional validation. (Manuscript, page 3, lines 4–10)

SHapley Additive exPlanations (SHAP) analysis identified descriptors associated with the HeLa-cell transfection phenotype. Head and Tail descriptors were calculated separately and assigned to their respective modules using feature prefixes (Fig. 3d). Complementary interpretability analyses are provided in Figs. S8–S12. Module-resolved rankings highlighted descriptors related to charge distribution, lipophilicity and topological connectivity (Fig. S13), while correlation-network analysis revealed related physicochemical clusters and partial redundancy among model inputs (Fig. S14). Together, these results suggest that in vitro activity reflects coordinated physicochemical properties rather than a single dominant feature. These associations are structural correlates of the in vitro phenotype; organ distribution and hepatocyte-enriched functional expression were evaluated independently in vivo. (Manuscript, page 5, lines 26–33)

Several limitations should be considered. The model was trained on HeLa-cell Fluc expression and did not include organ distribution or intrahepatic cell-type preference as training labels. It should therefore be viewed as a scalable enrichment tool for cell-intrinsic functional delivery, rather than a stand-alone predictor of systemic pharmacology. Accordingly, SHAP and descriptor analyses identify features associated with the in vitro phenotype, not causal determinants of hepatocyte-enriched delivery. Performance also decreased under strict double-cold validation, highlighting uncertainty for unseen head–tail combinations. Direct prediction of

hepatocyte preference will require matched datasets integrating lipid structure, formulation properties, circulation, organ exposure and cell-type-resolved functional labels. (Manuscript, page 10, lines 7–15)

Relevant revised figures:

The independent in vivo validation relevant to this comment is presented in revised Figs. 1a and 5j and Supplementary Figs. S45–S47, as described in detail in our responses to Reviewer #1, Comments 1 and 2. To avoid duplication, these figures are not reproduced here.

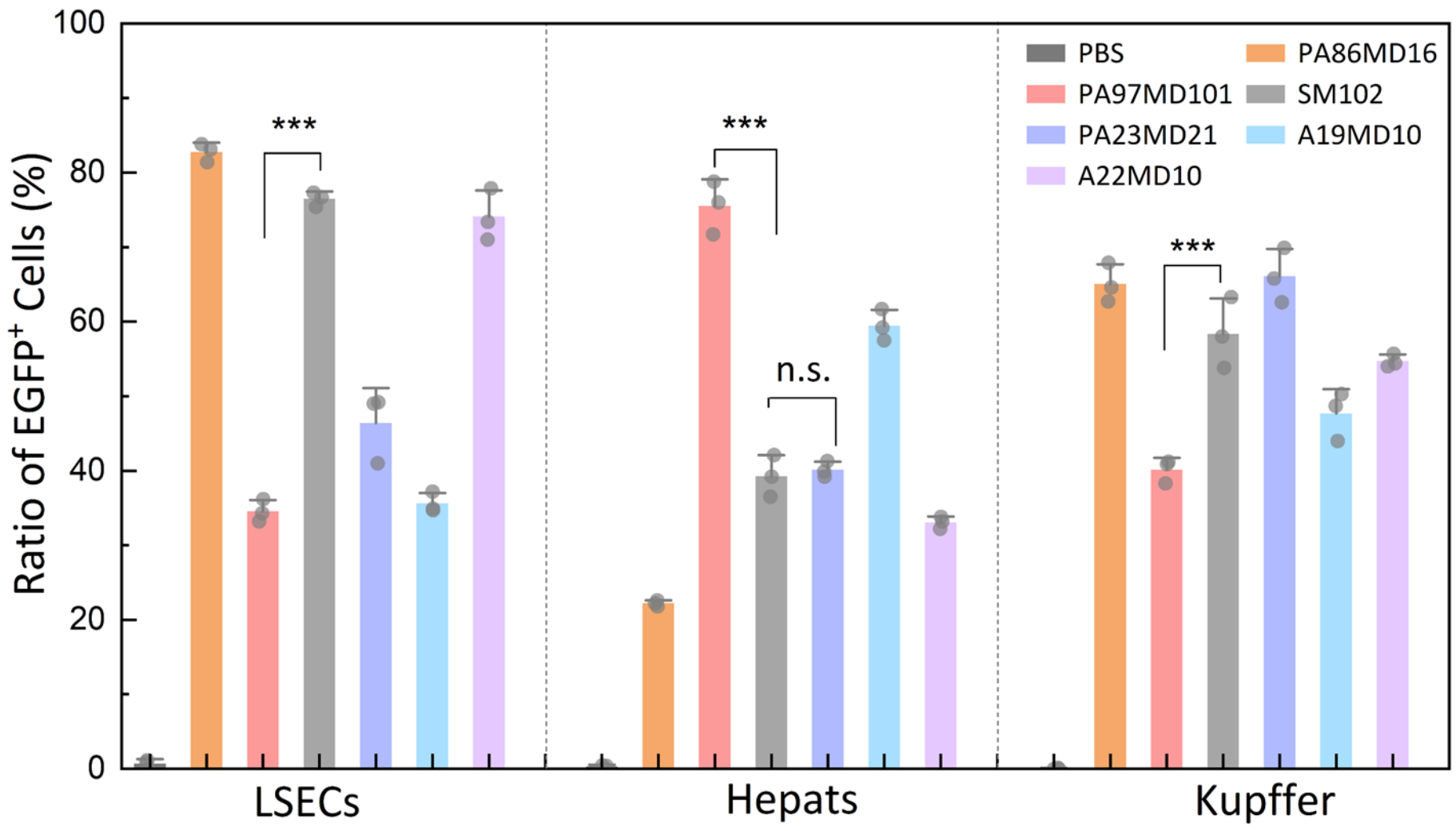

Revised Fig. 5j. EGFP activation within CD31⁻CD45⁻ hepatocyte-enriched, CD31⁺CD45⁻ endothelial/LSEC-enriched and CD45⁺ immune-cell populations after Cre mRNA–LNP administration. Data are mean ± s.d.; n = 3 biologically independent mice.

Comments 2: *The machine learning section still lacks several key methodological and reproducibility details.*

Comments 2.1: *The in vitro screening results were divided into three categories: non-hits, acceptable hits, and high-performing hits. However, it is unclear how these labels were used in the machine learning models. Was the ML task formulated as binary classification or three-class classification? Which samples were assigned to each class, and what were the sample sizes for each category? These details should be provided.*

Response:

The three activity categories presented in Fig. 1c were used only to describe and visualize the experimental screening landscape; they were not used as labels for model training. Among the 520 AMD lipids, 11 were categorized as non-hits (<10^3 RLU; 2.1%), 422 as acceptable hits (10^3–10^5 RLU; 81.2%), and 87 as high-performing hits (>10^5 RLU; 16.7%).

The machine-learning task was formulated as a binary classification problem. Each of the 520 unique head–tail combinations constituted one sample, and its label was assigned according to the mean Fluc signal measured from three technical replicates in HeLa cells. Lipids with Fluc signals <2,000 RLU were assigned to the low-activity class (class 0; n = 127, 24.4%), whereas those with signals ≥2,000 RLU were assigned to the active class (class 1; n = 393, 75.6%). More specifically, class 0 comprised all 11 non-hits and 116 acceptable hits with signals between 10^3 and 2,000 RLU, whereas class 1 comprised 306 acceptable hits with signals ≥2,000 RLU and all 87 high-performing hits. Thus, the binary labels used for model training were distinct from the three descriptive categories shown in Fig. 1c.

We have now clarified the learning task, label definitions, thresholds, sample numbers and replicate structure in the Methods, the Fig. 1c legend and Supplementary Note S1.

Revision of the manuscript and Supplementary Information:

We clarified that the three categories in Fig. 1c are descriptive, whereas model training used binary labels defined by an operational cutoff of 2,000 RLU. The Methods now report the class definitions and sample numbers (class 0, n = 127; class 1, n = 393), and the revised Fig. 1 legend reports the descriptive categories and counts (Manuscript, page 12, line 35 to page 13, line 2; page 19, lines 5–8). The same distinction and the interpretive limit of the cutoff are documented in Supplementary Note S1 (Supplementary Information, page 10, lines 14–29).

The machine-learning task was formulated as binary classification using in vitro Fluc mRNA expression measured in HeLa cells. The three activity categories were used to visualize the screening distribution, whereas model training used an operational cutoff of 2,000 RLU for candidate prioritization. Lipids with signals <2,000 RLU were assigned to the low-activity class (class 0), and those with signals ⩾2,000 RLU to the active class (class 1). The dataset comprised 520 AMD Head–Tail combinations, including 127 class-0 and 393 class-1 samples. (Manuscript, page 12, lines 33–38 and page 13, line 1-2)

New and revised figures:

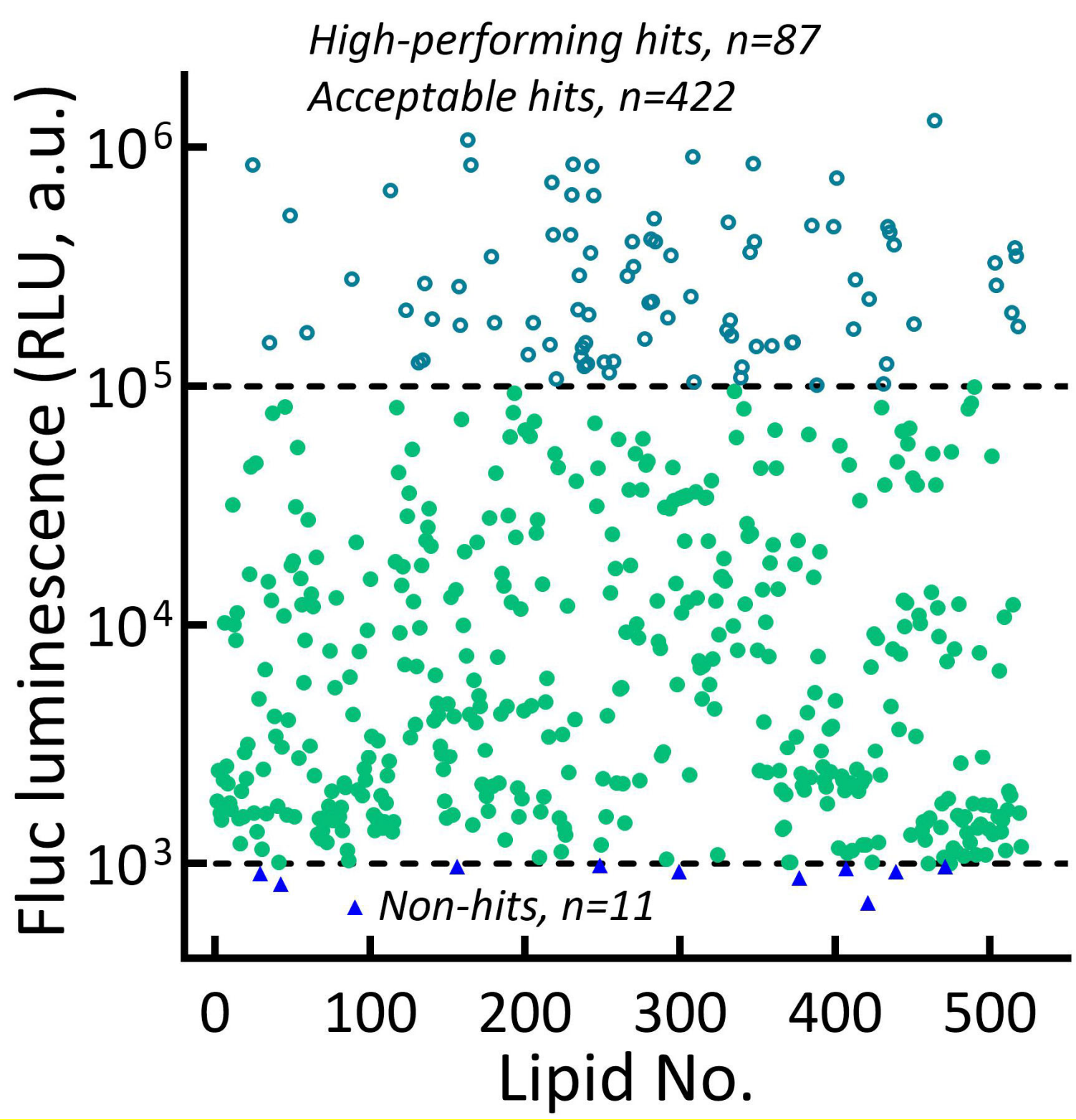

Revised Fig. 1c. HeLa-cell Fluc activity across the 520-member AMD library: non-hits (<10^3 RLU; n = 11), acceptable hits (10^3–10^5 RLU; n = 422) and high-performing hits (>10^5 RLU; n = 87). These categories are descriptive; binary model labels were defined at 2,000 RLU.

Supplementary Note S1. Scope, validation and interpretive limits of the machine-learning model. The machine-learning model in this study was designed as a candidate-enrichment model for in vitro functional mRNA transfection activity. Although the screening landscape in Fig. 1c is displayed using three descriptive activity categories, model training was formulated as a binary classification task using HeLa-cell Fluc expression, with Fluc signals <2,000 assigned to class 0 and signals >=2,000 assigned to class 1. The model was not trained on organ distribution, liver accumulation, intrahepatic cell-type preference or hepatocyte-specific functional output. Therefore, SHAP and descriptor analyses should be interpreted as identifying molecular features associated with the HeLa-cell in vitro transfection phenotype. To reduce preprocessing-

related confounding, all algorithms were compared using the same feature representation and preprocessing workflow, including RDKit descriptors, Morgan fingerprints, zero-variance feature filtering, feature standardization and training-fold-only class-imbalance handling. Head-grouped, tail-grouped and strict double-cold validation were used to evaluate generalization beyond random interpolation within the modular AMD matrix. These analyses support the use of XGBoost for candidate prioritization, but they do not establish direct prediction of hepatocyte-biased delivery. In vivo organ distribution and intrahepatic cell-type preference were therefore evaluated independently by bioluminescence imaging, Cre/LoxP reporter assays and flow cytometry. (Supplementary Information, page 10, lines 7–22)

Comments 2.2: *The RDKit physicochemical descriptors and Morgan fingerprints were used, but the manuscript does not provide sufficient computational details. For example, the authors should specify the radius of the Morgan fingerprints, whether low-variance features were removed, and whether feature standardization or normalization was performed, etc.*

Response:

We agree that the original manuscript did not provide sufficient detail regarding molecular featurization and preprocessing. We have therefore expanded the Methods and computational documentation to describe the complete workflow.

For each predefined amine-head and degradable-tail module, we generated a 2,048-bit Morgan fingerprint with a radius of 2 and calculated 208 RDKit physicochemical descriptors. The fingerprint and descriptor vectors were concatenated in a fixed order, yielding 2,256 features per module and a 4,512-dimensional input vector for each head–tail lipid combination.

All preprocessing was performed separately within each training split. After the training and held-out sets were defined, constant features were removed using VarianceThreshold (threshold = 0.0). Thus, the procedure removed zero-variance rather than arbitrarily defined low-variance features. The retained features were subsequently standardized by Z-score transformation using StandardScaler. Both the variance filter and scaling parameters were fitted exclusively on the training data and then applied unchanged to the held-out data, thereby preventing information leakage. Class-imbalance handling was likewise restricted to the training data.

Random Forest, XGBoost, LightGBM and Logistic Regression were re-evaluated using the same feature representation, matched data splits and unified preprocessing workflow. XGBoost remained the best-performing model overall and was therefore retained for virtual-library prioritization. The complete model comparison is now provided in Supplementary Table S1, and the featurization and preprocessing procedures have been documented in the revised Methods and accompanying code to improve transparency and reproducibility.

Revision of the manuscript and Supplementary Information:

We expanded the Methods to specify the 2,048-bit Morgan fingerprints (radius 2), 208 RDKit descriptors per module, fixed-order Head–Tail concatenation, 4,512-dimensional input representation, zero-variance filtering and Z-score standardization. We also clarified that all preprocessing and class-imbalance handling were fitted exclusively on training data and applied unchanged to held-out data (Manuscript, page 13, lines 3–19). The unified model comparison and preprocessing workflow are summarized in Supplementary Table S1 and Note S1 (Supplementary Information, page 10, lines 1–7 and 14–29).

The machine-learning task was formulated as binary classification using in vitro Fluc mRNA expression

measured in HeLa cells. The three activity categories were used to visualize the screening distribution, whereas model training used an operational cutoff of 2,000 RLU for candidate prioritization. Lipids with signals <2,000 RLU were assigned to the low-activity class (class 0), and those with signals ⩾2,000 RLU to the active class (class 1). The dataset comprised 520 AMD Head–Tail combinations, including 127 class-0 and 393 class-1 samples.

Random Forest, XGBoost, LightGBM and Logistic Regression were evaluated using identical molecular representations and preprocessing. For each predefined Head and Tail module, a 2,048-bit Morgan fingerprint (radius 2) and 208 physicochemical descriptors were calculated using RDKit. Fixed-order concatenation of the fingerprint and descriptor vectors generated a 4,512-dimensional representation for each Head–Tail combination. Zero-variance features were removed using VarianceThreshold(threshold = 0.0), followed by Z-score standardization with StandardScaler. All preprocessing, including variance filtering, scaling and class-imbalance handling, was fitted exclusively on training data and applied unchanged to held-out data to prevent information leakage.

Model performance was assessed using accuracy, positive-class F1, macro-F1, weighted F1, ROC-AUC and PR-AUC. Because the objective was candidate enrichment under class imbalance, macro-F1 and PR-AUC were emphasized during model comparison. Class imbalance was addressed within training folds using SMOTE-like oversampling, with class weighting evaluated as an alternative strategy. Generalization beyond random interpolation within the combinatorial AMD matrix was assessed using Head-grouped, Tail-grouped and strict double-cold validation. Identical Head modules, Tail modules, or both modules, respectively, were mutually exclusive between training and held-out sets. Based on its overall performance across the revised metrics under the unified preprocessing and validation workflow, XGBoost was selected for virtual-library prediction and candidate prioritization. (Manuscript, page 12, lines 37–38; page 13, lines 1–21)

<u>New and revised figures</u>:

Supplementary Note S1 and Methods: Expanded descriptions of fingerprint parameters, descriptor dimensions, feature concatenation, zero-variance filtering, Z-score standardization and training-data-only preprocessing.

Code: Updated scripts documenting molecular featurization, preprocessing and model evaluation.

Supplementary Table S1: Model performance under the unified preprocessing workflow. Performance of Random Forest, XGBoost, LightGBM, and Logistic Regression in the binary classification task using the unified preprocessing workflow. All models used the same feature representation and preprocessing steps, including RDKit molecular descriptors, Morgan fingerprints, zero-variance feature filtering, feature standardization, and training-fold-only SMOTE-like class balancing. Metrics include accuracy, positive-class F1, macro F1, weighted F1, ROC AUC, and PR AUC.

Model	Accuracy	Positive-class F1	Macro-F1	Weighted F1	ROC-AUC	PR-AUC
Random Forest	0.7788	0.8639	0.6371	0.7330	0.8009	0.9160
XGBoost	0.8654	0.9167	0.7833	0.8500	0.8590	0.9385
LightGBM	0.8077	0.8780	0.7118	0.7821	0.7932	0.9047
Logistic Regression	0.7981	0.8662	0.7272	0.7860	0.8045	0.9093

Comments 2.3*: In Fig. 3d, how the Head descriptor and Tail descriptor shown in Fig. 3d were generated was not clarified clearly. Were these features calculated separately for the amine head group and the degradable tail? and how these features were subsequently processed and integrated. In addition, given the potential interaction effects between the head group and tail, the authors are encouraged to include an analysis of head-tail interactions, or at least discuss whether the current models can capture such interactions.*

Response:

The Head and Tail features were calculated independently from the predefined two-dimensional structures of the amine-head and degradable-tail modules, rather than by fragmenting the final lipid products. For each module, we calculated 208 RDKit descriptors and a 2,048-bit Morgan fingerprint with a radius of 2. The Head and Tail vectors were then concatenated in a fixed order to generate a 4,512-dimensional joint representation, with Head_ and Tail_ prefixes preserving their module identities. All subsequent preprocessing was fitted using the training data only. In Fig. 3d, the non-fingerprint descriptors are grouped by these prefixes and ranked according to their mean absolute SHAP values.

Module-level analyses showed that descriptors from both modules were associated with the HeLa-cell transfection phenotype, although several highly ranked descriptors were correlated and should not be interpreted as independent mechanistic factors (Supplementary Figs. S8 and S9a, 9b). Because the combined representation allows XGBoost to learn nonlinear conditional relationships, we compared Head-only, Tail-only and combined models across matched validation splits. The combined model achieved a macro-F1 of 0.688 and PR-AUC of 0.914, compared with 0.654 and 0.909 for Head-only and 0.533 and 0.810 for Tail-only, respectively. Thus, Head features provided most of the predictive information, while Tail features contributed complementary information (Supplementary Fig. S12).

We also tested 225 explicit Head–Tail descriptor-pair interactions. All effects were weak ($|\rho| \leq 0.161$), and none remained significant after multiple-testing correction (FDR < 0.05; Supplementary Fig. S9c). Head–Tail hydrophobicity balance was nevertheless associated with activity: Head LogP − Tail LogP correlated more strongly with $\log_{10}$(RLU) ($\rho = -0.440$) than either Head or Tail LogP alone (Supplementary Figs. S10 and S11). We therefore conclude that the joint representation captures complementary information from both modules, but the current data do not support a stable interaction between any specific descriptor pair. These findings are interpreted as associations with the in vitro phenotype rather than causal determinants of in vivo delivery.

Revision of the manuscript and Supplementary Information:

We revised the machine-learning Results and Fig. 3b, 3d to explain that Head and Tail descriptors were calculated separately, retained by module-specific prefixes and integrated in a fixed-order joint representation (Manuscript, page 5, line 20 to page 6, line 8; page 22, lines 4–10). We added module-level association, descriptor-collinearity, explicit Head–Tail interaction, hydrophobicity-balance and feature-ablation analyses in Supplementary Figs. S8–S12 (Supplementary Information, pages 14–18). Supplementary Figs. S13 and S14 further show module-resolved descriptor importance and correlations among high-importance descriptors (pages 19–20).

Random Forest, XGBoost, LightGBM and Logistic Regression were compared using identical feature matrices, matched splits and training-data-only preprocessing, with macro-F1 and PR-AUC emphasized under class imbalance. XGBoost showed the best overall balance across the revised metrics and was selected for virtual-library prioritization (Table S1). Feature ablation showed that the combined Head–Tail representation performed best, with substantial predictive information retained in Head features and a more limited contribution from Tail features (Fig. 3b). Under structure-constrained validation, XGBoost retained candidate-

enrichment capability in Head- and Tail-grouped splits but showed reduced performance under strict double-cold validation, indicating uncertainty when both modules were unseen (Fig. 3c and Fig. S5). Additional baseline and class-imbalance sensitivity analyses supported this evaluation framework (Figs. S6 and S7).

SHapley Additive exPlanations (SHAP) analysis identified descriptors associated with the HeLa-cell transfection phenotype. Head and Tail descriptors were calculated separately and assigned to their respective modules using feature prefixes (Fig. 3d). Complementary interpretability analyses are provided in Figs. S8–S12. Module-resolved rankings highlighted descriptors related to charge distribution, lipophilicity and topological connectivity (Fig. S13), while correlation-network analysis revealed related physicochemical clusters and partial redundancy among model inputs (Fig. S14). Together, these results suggest that in vitro activity reflects coordinated physicochemical properties rather than a single dominant feature. These associations are structural correlates of the in vitro phenotype; organ distribution and hepatocyte-enriched functional expression were evaluated independently in vivo. (Manuscript, page 5, lines 26–33).

(a) Computational workflow for lipid discovery, including molecular featurization, SMOTE-like oversampling data balancing, and supervised model training. **(b)** Feature ablation of head-only, tail-only, and combined molecular representations under the unified ML workflow. **(c)** Model robustness evaluated under random, head-grouped, tail-grouped, and double-cold validation strategies. Blue and red dots indicate Macro F1 and PR AUC, respectively; black dots and error bars show mean ± s.d. across 20 splits. **(d)** SHAP analysis of molecular descriptors associated with the HeLa-cell transfection phenotype, shown separately for the Head and Tail modules. (Manuscript, page 22, lines 4–9).

New and revised supplementary figures:

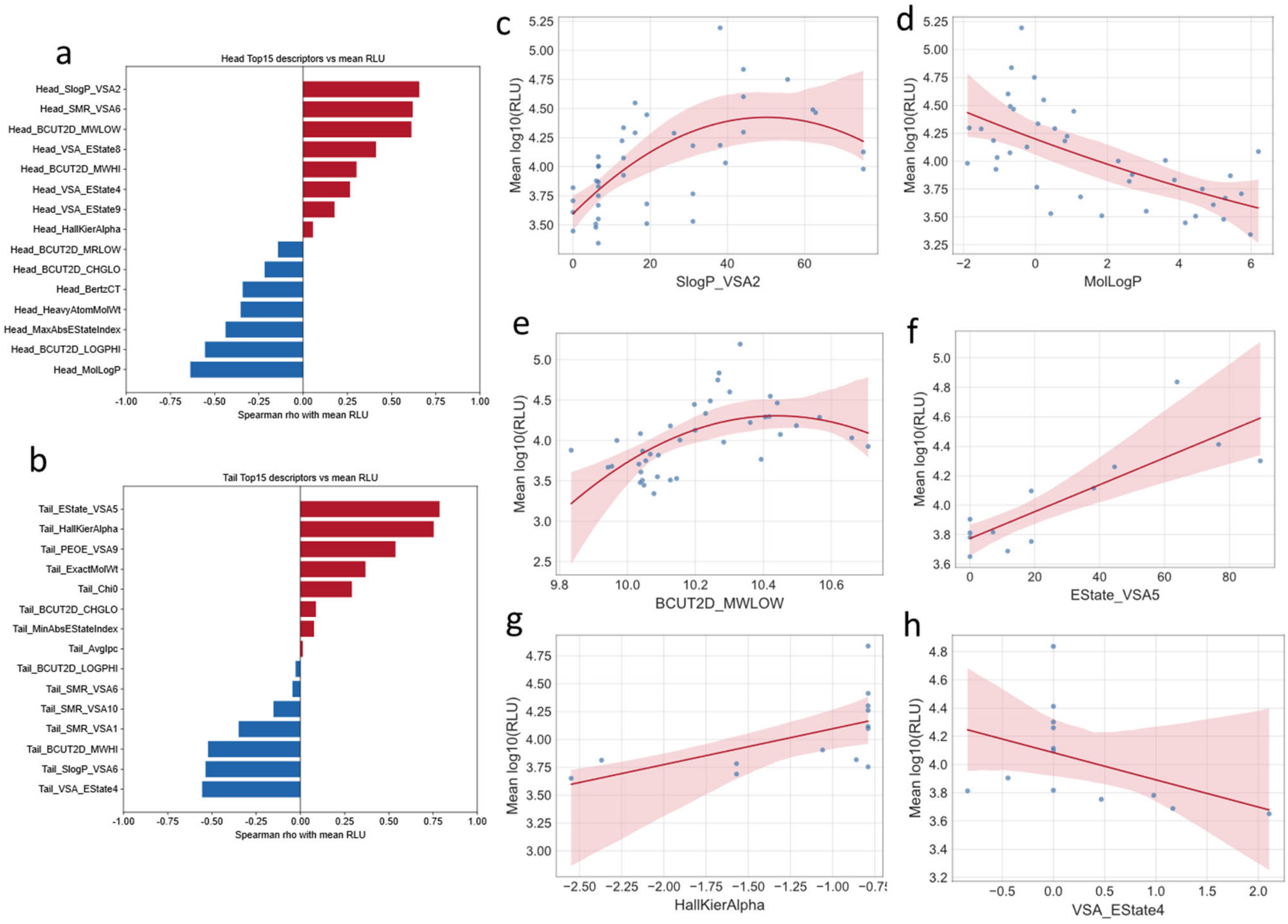

Figure S8. Module-level associations with in vitro transfection activity. Spearman correlations between selected Head or Tail descriptors and module-averaged log_{10}(RLU) across 40 amine heads and 13 degradable tails, respectively. Representative descriptor–activity relationships are also shown.

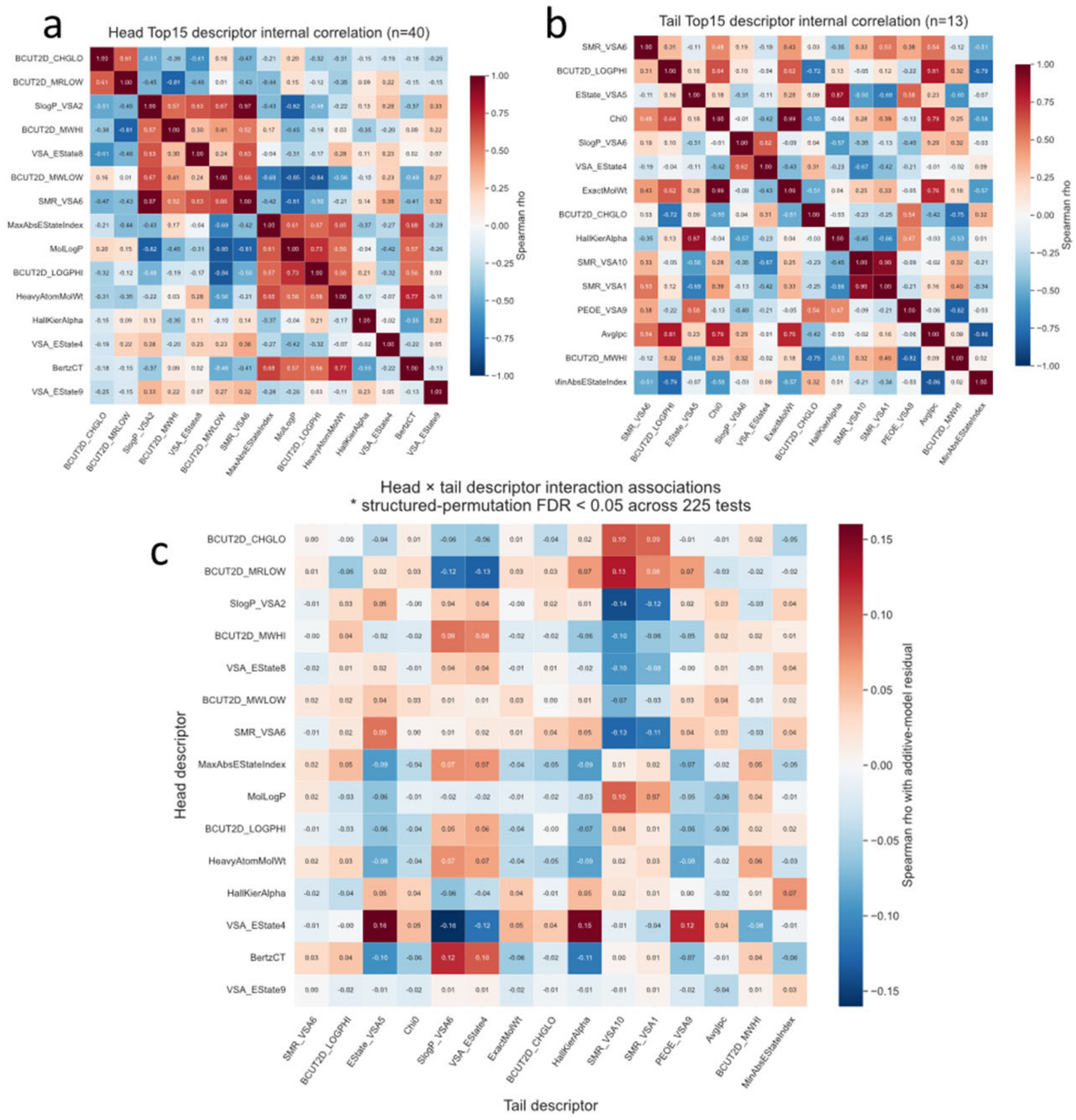

Figure S9. Descriptor collinearity and exploratory Head–Tail interaction analysis. Correlation matrices show relationships among the top-ranked Head and Tail descriptors. Analysis of 225 pairwise Head–Tail interaction terms identified no interaction that remained significant after multiple-testing correction (FDR < 0.05).

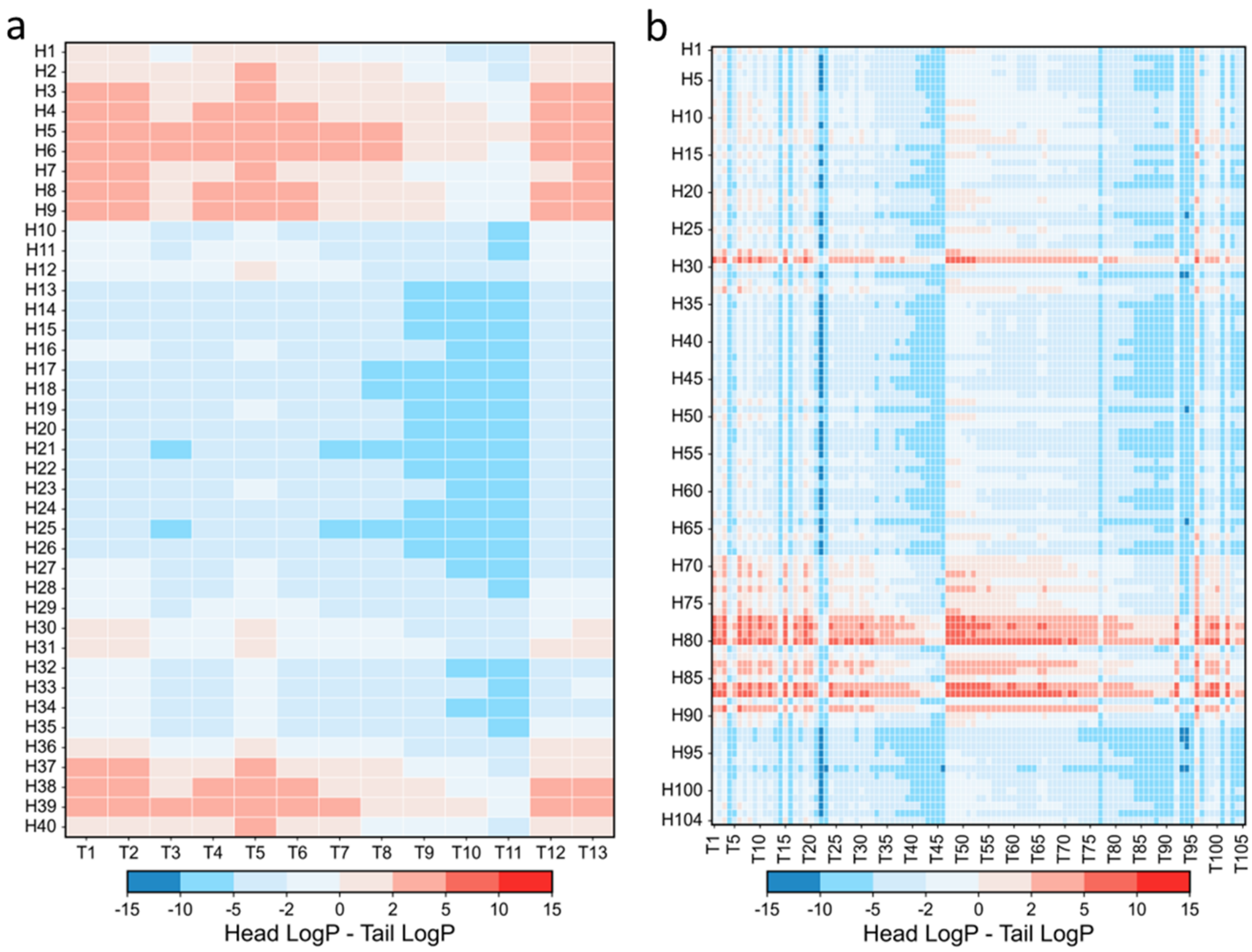

Figure S10. Head–Tail hydrophobicity balance across the lipid libraries. Heat maps show Head LogP − Tail LogP for the 40 × 13 experimental AMD library and the 104 × 105 virtual PAMD library. Red and blue indicate combinations with relatively more hydrophobic heads and tails, respectively.

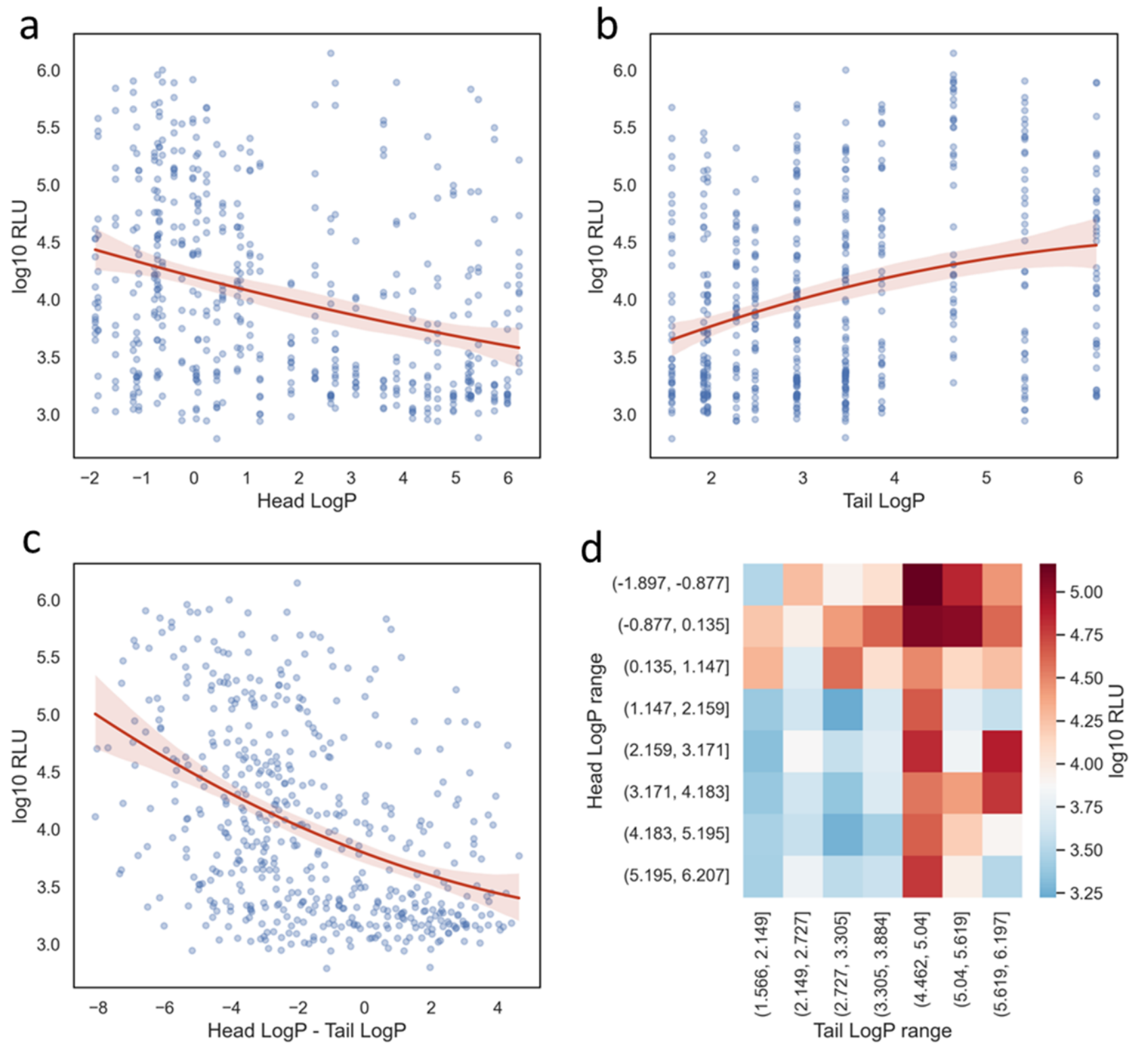

Figure S11. Association between Head–Tail hydrophobicity and in vitro activity. Relationships of Head LogP, Tail LogP and Head LogP − Tail LogP with $\log_{10}$(RLU) across the 520 experimental lipids, together with a two-dimensional Head–Tail LogP activity map.

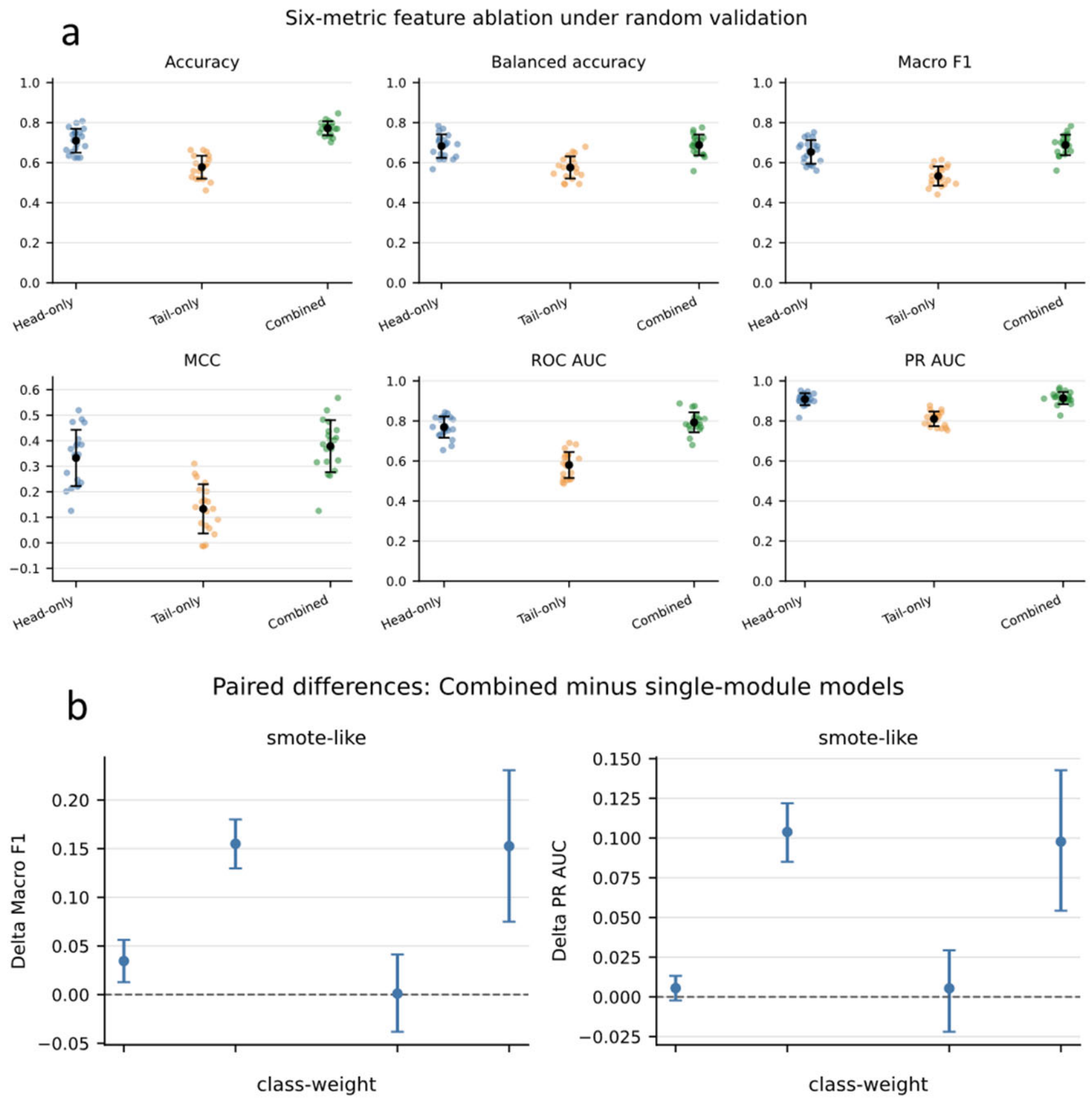

Figure S12. Ablation analysis of Head and Tail molecular representations. XGBoost performance was compared using Head-only, Tail-only and combined Head–Tail features across matched validation splits. The combined representation performed substantially better than Tail-only and modestly better than Head-only, indicating a complementary contribution from Tail features.

Comments 2.4: *It mentioned that the models were trained with cross-validation, but the specific data-splitting strategy is not sufficiently described. Moreover, because the AMD/PAMD lipid libraries have an apparent combinatorial matrix structure, random splitting may allow the training and test sets to share similar head groups or tails, leading to structural leakage and overestimation of model performance. The authors are encouraged to include methods like scaffold split or head-/tail-grouped validation to better assess model generalizability to new chemical space.*

Response:

We agree that random splitting within the combinatorial AMD matrix allows identical Head or Tail modules to occur in both training and held-out sets and may therefore overestimate generalization to unseen structural modules.

We consequently evaluated the combined Head–Tail XGBoost model using four validation strategies: (i) random validation; (ii) Head-grouped validation, in which identical Head modules were excluded from simultaneous occurrence in the training and test sets; (iii) Tail-grouped validation, applying the corresponding restriction to Tail modules; and (iv) strict double-cold validation, in which neither the Head nor the Tail module of a test lipid was present in the training set. Each strategy was evaluated across 20 repeated splits. Feature filtering, standardization and class-imbalance handling were fitted exclusively within each training split and then applied to the corresponding test set.

The mean macro-F1/PR-AUC values were 0.688/0.914 under random validation, 0.674/0.895 under Head-grouped validation, 0.652/0.909 under Tail-grouped validation and 0.594/0.829 under strict double-cold validation (Supplementary Fig. S5). Thus, the model retained candidate-enrichment capability when either module was unseen, but performance and stability decreased when both modules were absent from the training data.

These results confirm that random validation primarily measures interpolation within the AMD combinatorial matrix, whereas grouped and double-cold validation provide more stringent tests of module-level generalization. We have therefore revised the manuscript to avoid extrapolating random-split performance to unrestricted new chemical space. The model is now presented as a candidate-prioritization tool, with increasing uncertainty for combinations containing unseen modules and a continued requirement for experimental validation.

Revision of the manuscript and Supplementary Information:

We expanded the Methods to define Head-grouped, Tail-grouped and strict double-cold validation and to specify that identical modules were mutually exclusive between training and held-out sets in the corresponding analyses (Manuscript, page 13, lines 11–19). Model robustness under 20 repeated random, grouped and double-cold splits is presented in revised Fig. 3c and Supplementary Fig. S5 (Manuscript, page 22, lines 5–7; Supplementary Information, page 11, lines 2–10). The Discussion was revised to acknowledge reduced performance and increased uncertainty for unseen Head–Tail combinations (Manuscript, page 10, lines 7–15).

Random Forest, XGBoost, LightGBM and Logistic Regression were evaluated using identical molecular representations and preprocessing. For each predefined Head and Tail module, a 2,048-bit Morgan fingerprint (radius 2) and 208 physicochemical descriptors were calculated using RDKit. Fixed-order concatenation of the fingerprint and descriptor vectors generated a 4,512-dimensional representation for each Head–Tail combination. Zero-variance features were removed using VarianceThreshold(threshold = 0.0), followed by Z-score standardization with StandardScaler. All preprocessing, including variance filtering, scaling and class-imbalance handling, was fitted exclusively on training data and applied unchanged to held-out data to prevent information leakage.

Model performance was assessed using accuracy, positive-class F1, macro-F1, weighted F1, ROC-AUC and PR-AUC. Because the objective was candidate enrichment under class imbalance, macro-F1 and PR-AUC were emphasized during model comparison. Class imbalance was addressed within training folds using SMOTE-like oversampling, with class weighting evaluated as an alternative strategy. Generalization beyond random interpolation within the combinatorial AMD matrix was assessed using Head-grouped, Tail-grouped and strict double-cold validation. Identical Head modules, Tail modules, or both modules, respectively, were mutually exclusive between training and held-out sets. Based on its overall performance across the revised metrics under the unified preprocessing and validation workflow, XGBoost was selected for virtual-library prediction and candidate prioritization. (Manuscript, page 13, lines 13–21)

New and revised supplementary figures:

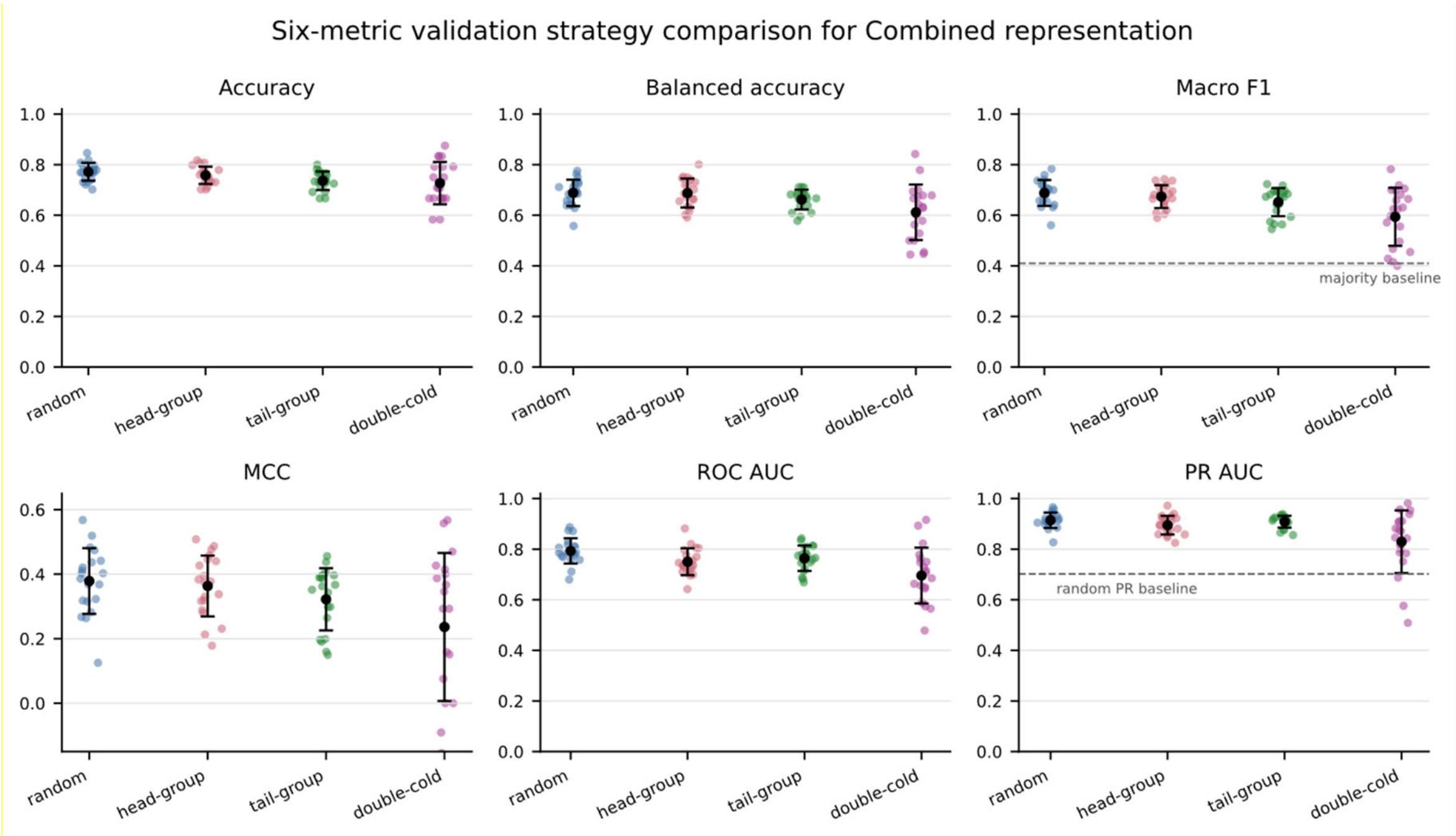

Supplementary Figure S5. Structure-constrained validation of the combined Head–Tail XGBoost model. Performance under random, Head-grouped, Tail-grouped and strict double-cold validation across 20 repeated splits. Points represent individual splits, and summary values are shown as mean ± s.d.

Comments 2.5*: The authors should explain the rationale for applying different preprocessing workflows to different models. For example, why was PCA applied only to RF and LR, but not to XGBoost or LightGBM? Although model-specific preprocessing strategies may be reasonable, using different feature representations could confound the comparison of algorithm performance.*

Response:

We thank the reviewer for identifying this potential confounder. In the original analysis, PCA was applied to Logistic Regression and Random Forest to reduce dimensionality and collinearity, whereas XGBoost and LightGBM were trained on the full feature space. We agree that comparing models using different representations does not isolate algorithm-specific performance.

We therefore removed model-specific PCA and re-evaluated Random Forest, XGBoost, LightGBM and Logistic Regression using an identical workflow. All models received the same 4,512-dimensional Head–Tail representation, matched splits, training-set-only zero-variance filtering, Z-score standardization and class-imbalance handling. Under this unified workflow, XGBoost retained the best overall balance, with an accuracy of 0.8654, macro-F1 of 0.7833, ROC-AUC of 0.8590 and PR-AUC of 0.9385 (Supplementary Table S1). Its

selection for virtual-library prioritization was therefore not dependent on the original PCA-based workflow.

To make the consequences of this reanalysis explicit in the main text, revised Fig. 3b compares Head-only, Tail-only and combined Head–Tail representations under the unified workflow. The combined representation showed the strongest overall performance, while Head features retained substantial predictive information and Tail features provided a more limited complementary contribution. We also regenerated the virtual-library rankings and ensemble-voting analysis using the unified workflow. Revised Fig. 3g shows the recurrent Head and Tail modules among the top-ranked candidates, with the principal prioritization patterns maintained. The extended module rankings are provided in Supplementary Figs. S18 and S19.

Finally, we compared training-fold-only SMOTE-like oversampling with class weighting across random, Head-grouped, Tail-grouped and strict double-cold validation. Both strategies produced similar performance patterns (Supplementary Figs. S6 and S7), indicating that the conclusions were not dependent on a single imbalance-handling strategy. The revised Methods, Supplementary Information and code now document the unified preprocessing, validation and candidate-prioritization workflow.

Revision of the manuscript and Supplementary Information:

We removed model-specific PCA and revised the Methods so that Random Forest, XGBoost, LightGBM and Logistic Regression use the same 4,512-dimensional representation, matched splits and training-data-only preprocessing (Manuscript, page 13, lines 3–19). Revised Fig. 3b shows Head-only, Tail-only and combined-representation ablation, while revised Fig. 3g and Supplementary Figs. S18–S19 show the regenerated module-prioritization patterns (Manuscript, page 22, lines 4–11; Supplementary Information, page 24, lines 2–7). Supplementary Table S1 and Figs. S6–S7 document the unified model comparison and sensitivity to class-imbalance handling (Supplementary Information, page 10, lines 1–7; page 12, lines 2–9; page 13, lines 2–8).

The machine-learning task was formulated as binary classification using in vitro Fluc mRNA expression measured in HeLa cells. The three activity categories were used to visualize the screening distribution, whereas model training used an operational cutoff of 2,000 RLU for candidate prioritization. Lipids with signals $<2{,}000$ RLU were assigned to the low-activity class (class 0), and those with signals $\geqslant 2{,}000$ RLU to the active class (class 1). The dataset comprised 520 AMD Head–Tail combinations, including 127 class-0 and 393 class-1 samples. (Manuscript, page 12-13, lines 35–35 and line 1-2)

New and revised supplementary figures:

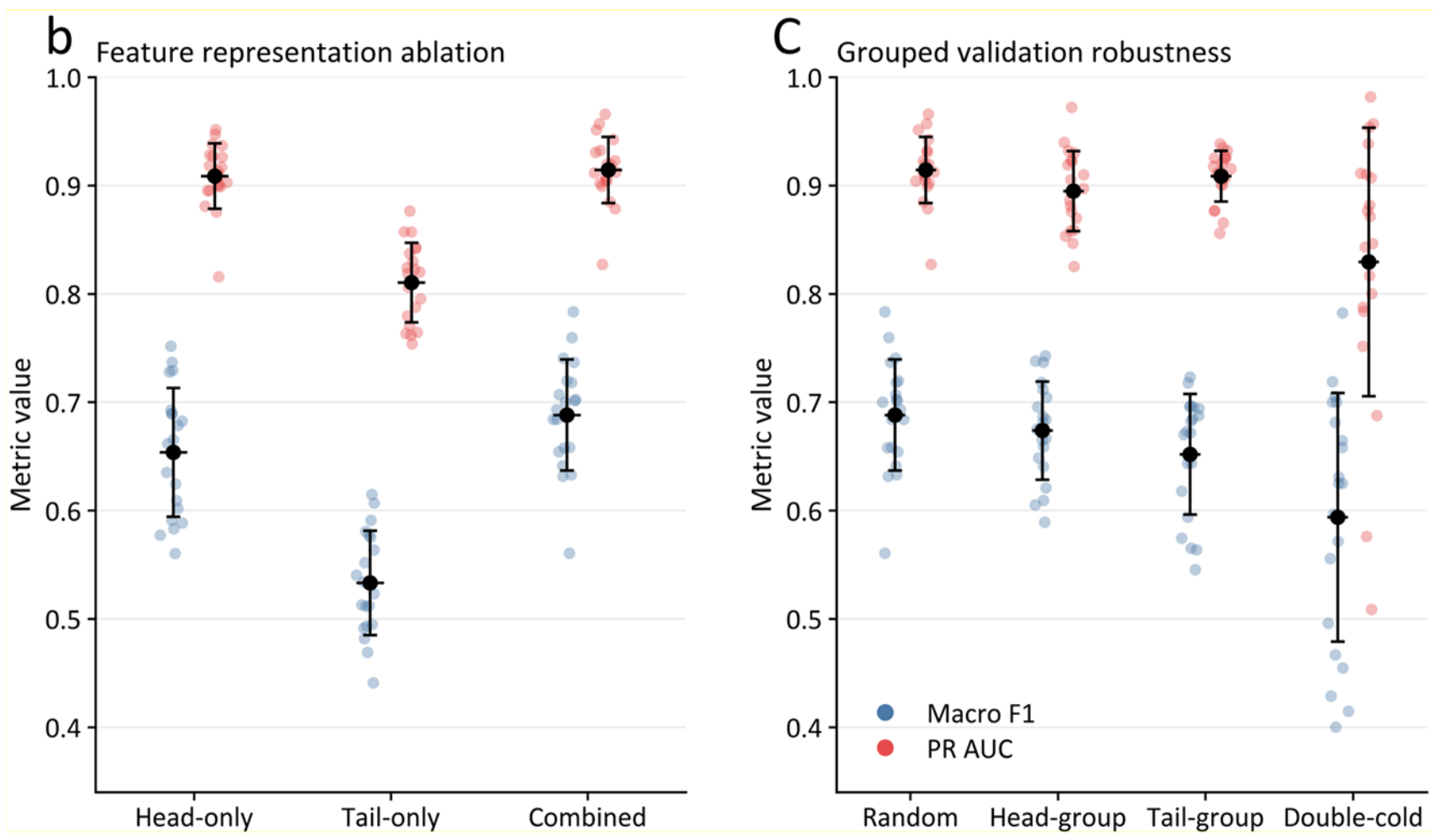

Figure S6. Macro-F1 and PR-AUC using training-fold-only oversampling or class weighting across four validation strategies and 20 repeated splits.

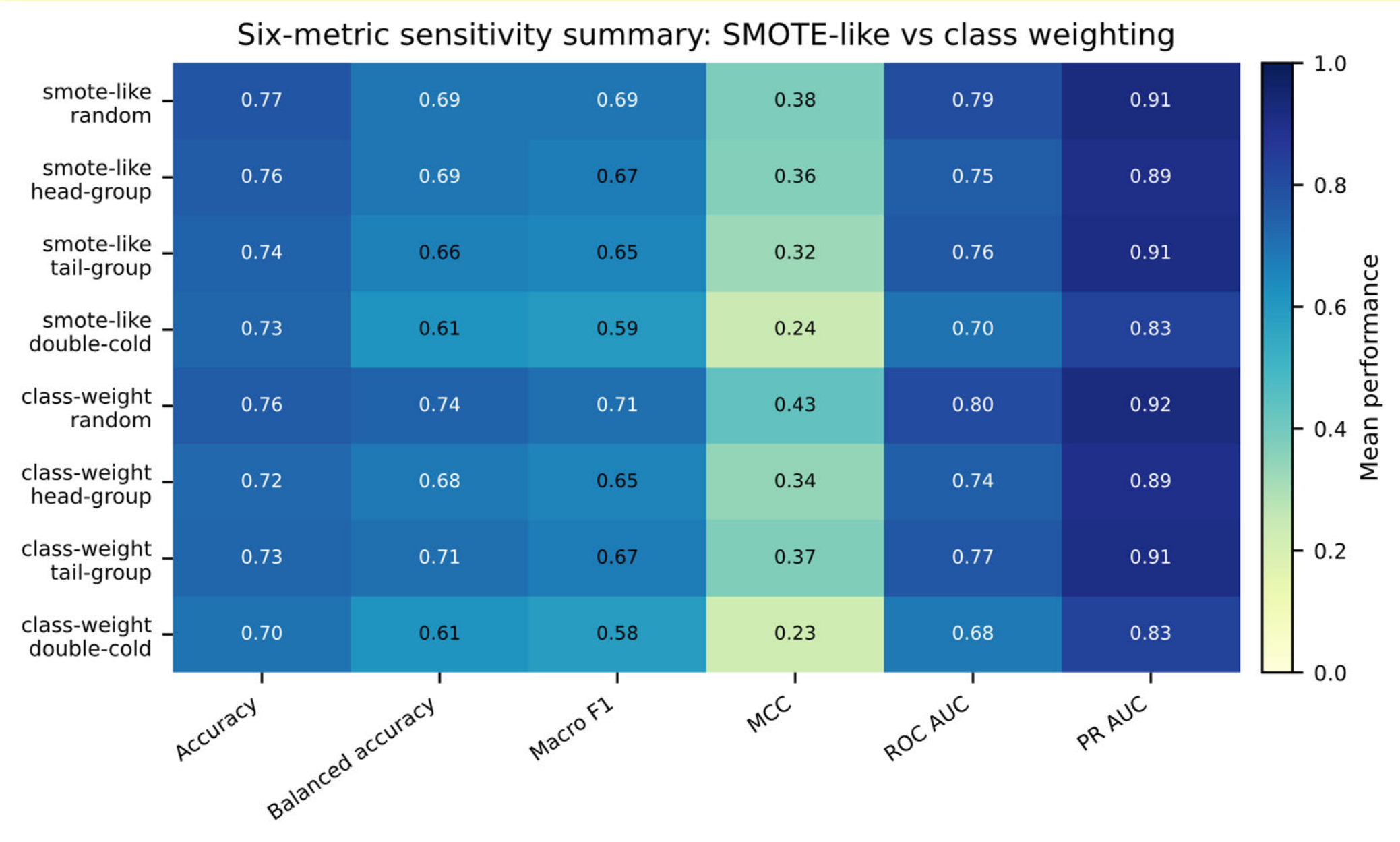

Figure S7. Six-metric summary of XGBoost performance across the indicated imbalance-handling and validation strategies.

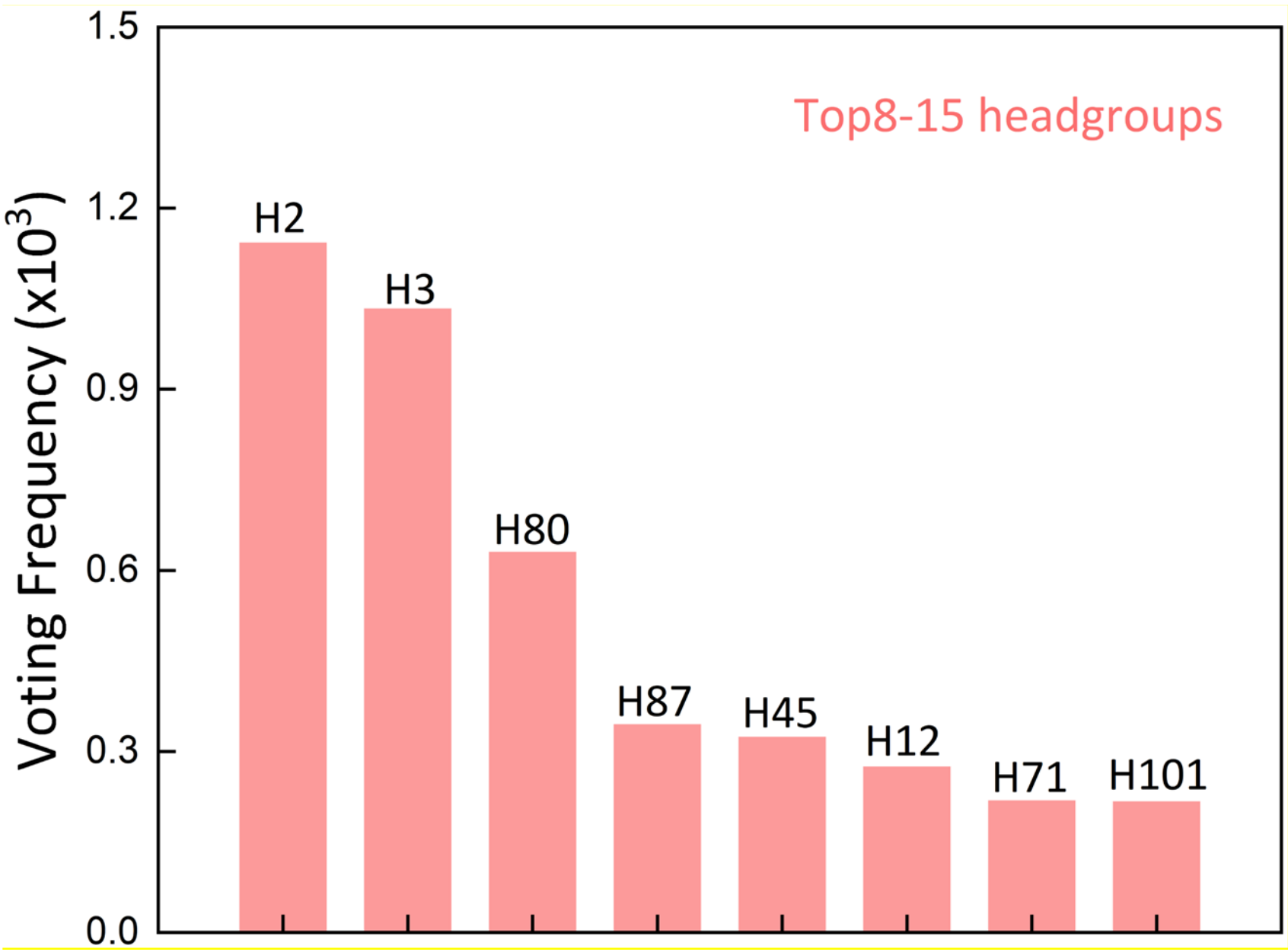

Figure S18. Frequency distribution of privileged amine heads (ranking from 8 to 15) identified through ensemble voting from the top-ranked candidates.

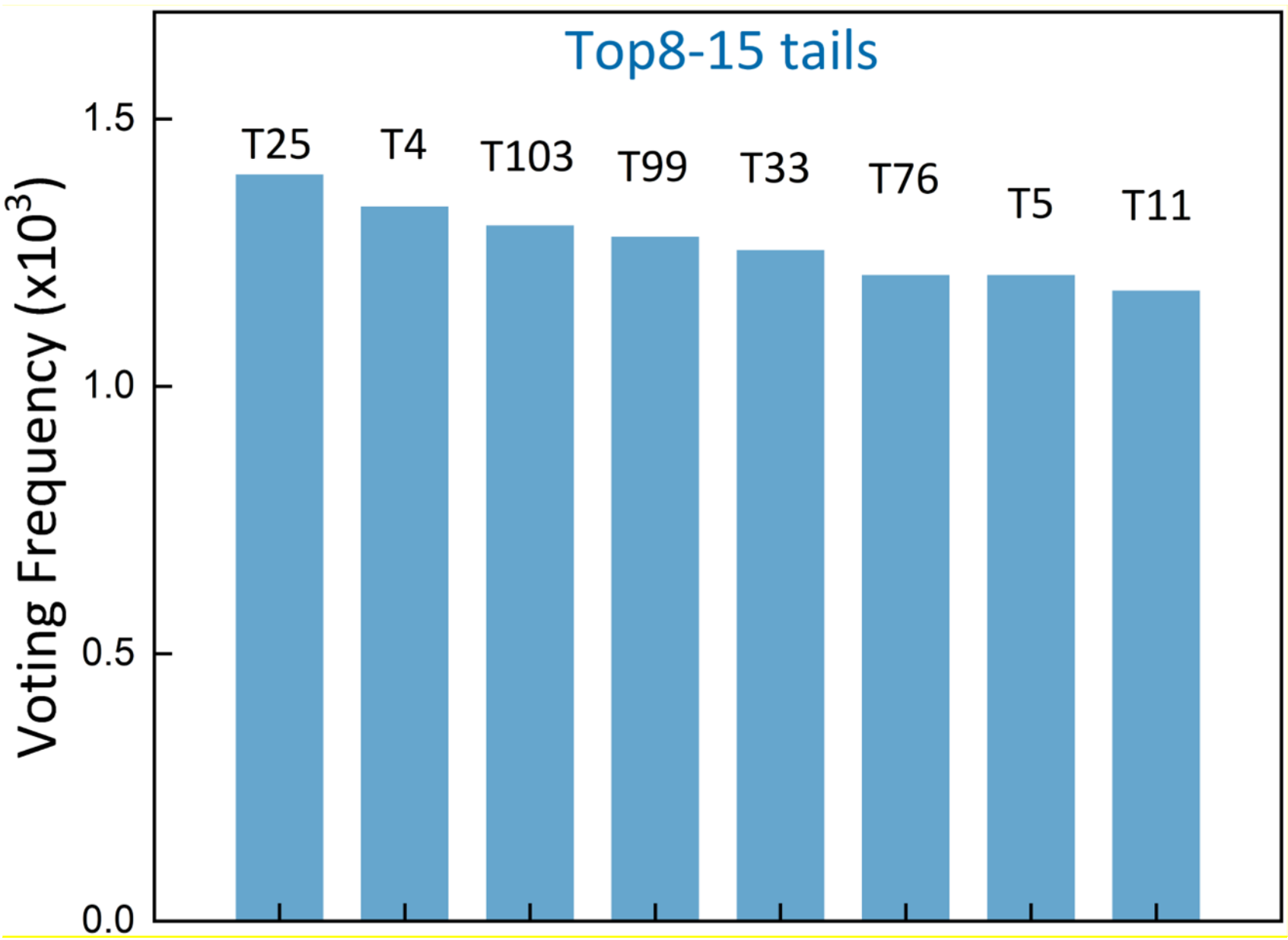

Figure S19. Frequency distribution of privileged degradable tails (ranking from 8 to 15) identified through ensemble voting from the top-ranked candidates.

Responses to reviewers

We thank the Editor and Reviewers for their constructive comments and careful evaluation of our manuscript. Revisions made in response to the reviewers are highlighted in yellow in the marked manuscript, whereas changes addressing the editorial requests are shown in red. Detailed responses to the editorial requests are provided in the completed Author Checklist.

Reviewer #1

The authors have addressed my concerns. I recommend accepting this manuscript.

Response:

We sincerely thank the reviewer for their careful evaluation of our manuscript and recommendation for acceptance. We greatly appreciate the reviewer's time and constructive feedback throughout the review process.

Reviewer #3

General Comment.

There is a tendency by the authors, in the Discussion section, to frame this study as establishing a framework for the development of hepatocyte-focused ionizable lipids. For example, the sentence in page 9, lines 27-30 still emphasizes "hepatocyte-focused framework". However, the authors appear more cautious about this claim later in the discussion presumable in response to other reviewer's comments. Importantly, only one (or two?) of the lipids demonstrated hepatocyte-enriched delivery, and the in vitro data used to develop the model were not generated using hepatocyte-based systems. Given these limitations, I suggest that the authors emphasize hepatic delivery (in the topic and body of the manuscript) rather than hepatocyte-biased delivery, as the current data do not appear sufficient to support broader conclusions regarding hepatocyte-specific delivery.

Response:

We thank the reviewer for this important comment and agree that the overall scope of the study should emphasize hepatic delivery rather than imply a general capability to design hepatocyte-biased ionizable lipids. We have therefore revised the title and the relevant text throughout the manuscript and Supplementary Information to make this distinction explicit.

Specifically, the machine-learning model in this study was trained on HeLa-cell Fluc expression and was used to prioritize ionizable-lipid candidates on the basis of predicted *in vitro* transfection activity. It was not trained on organ distribution, hepatic accumulation, or intrahepatic cell-type preference. These properties were evaluated independently in subsequent *in vivo* experiments. Accordingly, hepatocyte-enriched functional expression is now described only as an experimentally observed property of PA97MD101 under the tested conditions, based on the mTmG reporter and flow-cytometry analyses, and is not generalized to the model, the screening framework, or the broader lipid library.

To reflect this revised scope, we have changed the title to: **"Interpretable AI-guided discovery of degradable ionizable lipids with enhanced hepatic mRNA delivery in vivo."** We have also revised the Abstract, Introduction, Results, Discussion, figure legends and Supplementary Information to consistently separate machine-learning-based candidate prioritization from independent *in vivo* evaluation. The Discussion now explicitly states that the hepatic performance and intrahepatic expression profile of PA97MD101 were established experimentally and do not demonstrate a

general ability to design hepatocyte-selective lipids. We further clarify that direct prediction of tissue or cell-type preference would require training datasets containing the corresponding cell-type-resolved *in vivo* functional measurements.

In the Supplementary Information, the descriptions of the virtual-screening results have likewise been revised to refer to candidates with higher predicted *in vitro* transfection activity rather than hepatocyte-specific delivery. The physicochemical characterization of the prioritized validation library is presented as experimental characterization rather than an outcome directly predicted by the model, and the organ-level biodistribution analysis is now explicitly described as liver-dominant fluorescence that does not establish hepatocyte-specific delivery or receptor-mediated targeting.

Together, these revisions ensure that hepatic delivery is the overall focus of the study, whereas hepatocyte-enriched functional expression is presented only as a candidate-specific property of PA97MD101 established through independent *in vivo* experiments. We believe that this revised framing more accurately reflects both the predictive scope of the model and the experimental evidence in the manuscript.

Revision of the manuscript

1. Title

The title has been revised from the previous hepatocyte-focused wording to:

"Interpretable AI-guided discovery of degradable ionizable lipids with enhanced hepatic mRNA delivery in vivo."

2. Abstract

We now define the scope of the workflow as candidate prioritization based on *in vitro* transfection activity followed by independent evaluation of hepatic delivery *in vivo*:

"Starting from a 520-member library, we train an XGBoost model on firefly luciferase (Fluc) expression in HeLa cells and use physicochemical descriptors and molecular fingerprints to prospectively prioritize candidates with higher predicted *in vitro* transfection activity across a 10,920-member virtual space. *In vivo* phenotyping subsequently identifies PA97MD101 as a lead ionizable lipid."

The Abstract now concludes:

"These findings support further evaluation of PA97MD101 for functional hepatic RNA delivery."

3. Introduction and Results

The study is now consistently framed as a two-stage workflow:

"A key challenge is therefore to establish a data-efficient workflow that prioritizes ionizable lipids using *in vitro* transfection data and subsequently evaluates their hepatic functional delivery *in vivo*."

The relevant Results section has also been retitled:

"PA97MD101 enhances hepatic mRNA delivery in vivo."

4. Discussion

We have removed the previous "hepatocyte-focused framework" language and now state:

"Here we establish an interpretable workflow for the prospective prioritization of degradable ionizable lipids using *in vitro* transfection data, followed by independent evaluation of hepatic mRNA delivery *in vivo*."

We further clarify the candidate-specific nature of the intrahepatic phenotype:

"The model supports candidate prioritization based on HeLa-cell transfection activity, while subsequent experiments identify PA97MD101 as a lead lipid for hepatic mRNA delivery. Its intrahepatic expression profile represents an experimentally observed property under the tested conditions and does not establish a general ability to design hepatocyte-selective lipids."

5. Figure legends

The Fig. 1 and Fig. 5 legends now refer to the $CD31^{-}CD45^{-}$ compartment as a **hepatocyte-enriched population** and describe the PA97MD101 phenotype as **hepatocyte-enriched functional expression**, rather than implying hepatocyte-specific targeting.

Revision of the Supplementary Information

The Supplementary Information has been revised in parallel with the main manuscript to ensure consistent interpretation of the machine-learning and in vivo results.

First, the virtual-screening sections now describe ML-selected candidates in terms of **higher predicted *in vitro* transfection activity**, rather than hepatocyte-specific delivery (Supplementary Figs. 13–15).

Second, the physicochemical characterization of the focused validation library is now presented as experimental characterization of the **ML-prioritized validation library**, without implying that nanoparticle stability or colloidal behavior was directly predicted by the model (Supplementary Fig. 19).

Third, the organ-level biodistribution analysis is now described as **liver-dominant fluorescence under the tested conditions**, and the legend explicitly states that these measurements do not establish hepatocyte-specific delivery or receptor-mediated targeting (Supplementary Fig. 29).

During our final consistency check, we also identified and corrected several minor textual and labeling inconsistencies in **Supplementary Figs. 2, 19 and 23**, including the description of representative lipid candidates, panel annotations and terminology used for the ML-prioritized candidates and fluorescence channels. These corrections improve clarity and consistency and do not affect the underlying data, analyses or conclusions.

Response to comment 1 from Reviewer 2.

While the author's response to this comment is adequate, implementing the suggestion in the Generalcomment above would ensure they completely address this comment.

Response:

We thank the reviewer for confirming that our previous response to Reviewer 2's Comment 1 was adequate and for emphasizing the importance of applying the clarified scope consistently throughout the manuscript. In accordance with the General Comment above, we have revised the title and the relevant sections of the manuscript and Supplementary Information to emphasize hepatic delivery and to remove or qualify statements

that could imply a general ability of the model to predict hepatocyte-biased delivery.

We now explicitly distinguish the overall screening framework from the hepatocyte-enriched functional expression observed for PA97MD101 in the mTmG reporter and flow-cytometry experiments. This phenotype is presented as a candidate-specific experimental observation under the tested conditions and is not generalized to the model or the broader lipid library.

We believe these revisions fully align the manuscript with the scope and limitations identified by the reviewer.

Response to comment 2 from Reviewer 2.

In my opinion, the authors' responses to the comments concerning the methodology used for model development were satisfactory and adequately addressed the issues raised. Individual comments are addressed below.

The author's responses to comments 2.1 were adequate.

The author's responses to comments 2.2 were adequate.

The author's responses to comments 2.3 were adequate.

The author's responses to comments 2.4 were adequate.

The author's responses to comments 2.5 were adequate.

Response:

We sincerely thank the reviewer for carefully evaluating our responses to Reviewer 2's Comments 2.1–2.5 and for confirming that the concerns regarding model development have been satisfactorily addressed. We appreciate the reviewer's constructive assessment, which has helped us improve the clarity, reproducibility and interpretation of the machine-learning methodology.
